# Quantitative evaluation of 3D dosimetry for stereotactic volumetric‐modulated arc delivery using COMPASS

**DOI:** 10.1120/jacmp.v16i1.5128

**Published:** 2014-01-07

**Authors:** Subramani Vikraman, Durai Manigandan, Karukkupalayam Palaniappan Karrthick, Raju Sambasivaselli, Vellaingiri Senniandavar, Mahendran Ramu, Thiyagarajan Rajesh, Muller Lutz, Manavalan Muthukumaran, Nithyanantham Karthikeyan, Kataria Tejinder

**Affiliations:** ^1^ Division of Radiation Oncology Medanta The Medicity Hospital Gurgaon Haryana India; ^2^ Department of Radiation Oncology Sri Sidhivinayak cancer Hospital Miraj MH India; ^3^ International Competence Centre IBA Dosimetry GmbH Schwarzenbruck Germany; ^4^ Department of Radiation Oncology Apollo Specialty Hospital Chennai Tamil Nadu India; ^5^ Department of Radiation Oncology St John's Medical College and Hospital Bangalore Karnataka India; ^6^ Research and Development Bharathiar University Coimbatore Tamil Nadu India

**Keywords:** VMAT, COMPASS, 3D dosimetry CCA COMPASS‐calculated, CME COMPASS‐measured

## Abstract

The purpose of this study was to evaluate quantitatively the patient‐specific 3D dosimetry tool COMPASS with 2D array MatriXX detector for stereotactic volumetric‐modulated arc delivery. Twenty‐five patients CT images and RT structures from different sites (brain, head & neck, thorax, abdomen, and spine) were taken from CyberKnife Multiplan planning system for this study. All these patients underwent radical stereotactic treatment in CyberKnife. For each patient, linac based volumetric‐modulated arc therapy (VMAT) stereotactic plans were generated in Monaco TPS v3.1 using Elekta Beam Modulator MLC. Dose prescription was in the range of 5–20 Gy per fraction. Target prescription and critical organ constraints were tried to match the delivered treatment plans. Each plan quality was analyzed using conformity index (CI), conformity number (CN), gradient Index (GI), target coverage (TC), and dose to 95% of volume ($D_{95}$). Monaco Monte Carlo (MC)‐calculated treatment plan delivery accuracy was quantitatively evaluated with COMPASS‐calculated (CCA) dose and COMPASS indirectly measured (CME) dose based on dose‐volume histogram metrics. In order to ascertain the potential of COMPASS 3D dosimetry for stereotactic plan delivery, 2D fluence verification was performed with MatriXX using MultiCube phantom. Routine quality assurance of absolute point dose verification was performed to check the overall delivery accuracy. Quantitative analyses of dose delivery verification were compared with pass and fail criteria of 3 mm and 3% distance to agreement and dose differences. Gamma passing rate was compared with 2D fluence verification from MatriXX with MultiCube. Comparison of COMPASS reconstructed dose from measured fluence and COMPASS computed dose has shown a very good agreement with TPS calculated dose. Each plan was evaluated based on dose volume parameters for target volumes such as dose at 95% of volume ($D_{95}$) and average dose. For critical organs dose at 20% of volume ($D_{20}$), dose at 50% of volume ($D_{50}$), and maximum point doses were evaluated. Comparison was carried out using gamma analysis with passing criteria of 3 mm and 3%. Mean deviation of 1.9%±1% was observed for dose at 95% of volume ($D_{95}$) of target volumes, whereas much less difference was noticed for critical organs. However, significant dose difference was noticed in two cases due to the smaller tumor size. Evaluation of this study revealed that the COMPASS 3D dosimetry is efficient and easy to use for patient‐specific QA of VMAT stereotactic delivery. 3D dosimetric QA with COMPASS provides additional degrees of freedom to check the high‐dose modulated stereotactic delivery with very high precision on patient CT images.

PACS numbers: 87.55.Qr, 87.56.Fc

## I. INTRODUCTION

The novel 3D dosimetric quality assurance system COMPASS (Ion Beam Applications (IBA) Dosimetry GMBH, Schwarzenbruck, Germany) allows an independent verification of TPS and faster dose volume‐based correlation with measured data for volumetric‐modulated arc therapy, including tissue inhomogeneity correction.^(1)^ The rapid increase of clinical usage of stereotactic VMAT (Elekta Ltd., Stockholm, Sweden) or RapidArc (Varian Medical Systems, Palo Alto, CA) needs an extensive dosimetric verification to ensure correct treatment delivery applying an easy and fast paradigm. The complex VMAT is delivered by changing gantry speed, MLC aperture, and dose rates in single or multiple coplanar or noncoplanar arcs. VMAT will be the treatment of choice for a substantial number of tumor sites. It delivers highly conformal dose to the target while sparing critical organs, and allows shorter treatment time.^(2)^ The main advantage of VMAT is undoubtedly the treatment time reduction, yet with similar plan quality from treatment planning point of view.^(3)^ Monaco (Elekta Ltd.) Monte Carlo‐calculated stereotactic VMAT plans offer shorter treatment time and highly effective organ at risk sparing (without compromising the plan quality) and better conformity, thereby improving patient comfort and clinical throughput. A prolonged fractional delivery theoretically could increase the interfractional radiation damage repair within the tumor cell, which would impair the treatment efficacy. Therefore, shorter treatment time and effective organ sparing with better conformity are paramount in stereotactic VMAT delivery. Many authors have studied the treatment plan efficiency of stereotactic VMAT and it is apparent that the Monaco planning system generates efficient plans for stereotactic VMAT by the use of biological cost functions and proven Monte Carlo algorithm.^(4)^ Three‐dimensional dosimetry based, for example, on linac log files, gel dosimetry or portal dosimetry, can assess the accuracy of delivered dose, but it will not provide an independent cross check of the beam model in the TPS. Gel dosimetry has the potential for volumetric measurements with good spatial resolution and isotropic response, but gels are complex in preparation and the handling can be considered as an experimental procedure.^(5)^ Three‐dimensional dosimetry using back‐projection of portal dosimetry for VMAT has proven to be a powerful tool for dosimetric verification of complex radiotherapy, but the various studies report the over sensitivity and response dependence (ghosting, image lag, aging) of EPID.^(3)^ To adapt clinically the EPID dosimetry for rotational therapy one needs to take into account the gantry angle resolved dosimetric information, calculation of phantom or patient transmission, and detector sag as a function of gantry angle.^(3)^ In addition, the presence of noncoplanar VMAT fields may cause clearance problems for EPID dosimetry.^(2)^ Quantitative analysis of patient specific QA measurements with high spatial accuracy is of particular interest given the dynamic delivery of VMAT. EBT3 film could be the detector of choice for stereotactic delivery due to its high spatial accuracy, which eliminates the lack of electronic equilibrium in small radiation fields. The uncertainty associated with GAFCHROMIC EBT3 film dosimetry, however, (involving calibration, scanning, and handling of the film) requires a stringent procedure. Therefore, it cannot be considered to be a fast and reliable method of dosimetry, compared to the novel 3D dosimetry tool. The complexity in delivery of VMAT and hypofractionated dose raises demand for fast and precise 3D dosimetry to verify the patient‐specific treatment plan delivery accuracy prior to treatment. In this study we have evaluated the use of 3D dosimetry for VMAT stereotactic dose delivery. To highlight the potential of 3D dosimetry in stereotactic dose delivery, the 2D dose maps measured by MatriXX (IBA Dosimetry) with MultiCube were analyzed as well. To assess the overall delivery accuracy, absolute dose measurements were performed using Exradin A16 chamber (Standard Imaging Inc., Middleton, WI) in a stereotactic dose verification phantom.

## II. MATERIALS AND METHODS

### A. Monaco stereotactic VMAT planning

For all patients, stereotactic VMAT plans were generated using CMS Monaco TPS v 3.01 with 6 MV for Elekta Synergy S Beam Modulator linac, which has 4 mm leaf width at isocenter and a maximum field size of $16\times 21\;{\text{cm}}^{2}$. Full arc sequences of 360° and an arc interval of 30° was used for all the treatment plans. Partial arcs were selected for tumors located unilaterally in order to avoid beam entry through critical organs. A calculation grid resolution of 2 mm was used as recommended for stereotactic delivery.^(6)^ Secondary dose calculation algorithm was selected as Monte Carlo and calculations were performed to medium (not $H_{2}O$). The beamlet width was chosen as 2 mm. Each plan was generated using the semibiological cost functions. Monaco contains three biological cost functions (EUD, parallel, serial) and five physical (max dose, overdose/underdose volume constraint, quadratic over/underdose) cost functions, two of which are dose‐volume histogram–based cost functions. We have used the combination of biological and physical cost functions. Monaco comprises two different algorithms: finite‐size pencil beam (fsPB) and Monte Carlo. The finite‐size pencil beam algorithm was used in the first stage of optimization process, but it is not recommended to be used for final dose calculation by the vendor for the version Monaco 3.01. The Optimization processes in Monaco are divided into two steps; the way they are involved in the process varies depending on the treatment delivery mode. In stage one, the ideal fluence distribution is optimized to achieve the prescription goals, and in stage two the ideal fluence is translated into deliverable segments with relative shapes and weights. Monaco calculates the static sectors from the total arc length and the angular increments to simulate the static IMRT planning process. Monaco uses the sweep sequencer that significantly improves the delivery efficiency and minimizes the loss of dose delivery quality. This sweep sequencer moves the leaves from start position to their end position in a continuous and unidirectional manner.^(7)^ We have used a Monte Carlo variance of 3% per control point. Each plan was generated in Monaco with the aim of meeting given critical organ constraints, better conformity with very steep dose gradients, and good target coverage. We have tried to achieve that a minimum of 95% of target volume receives 100% of prescription dose and a conformity index as close as possible to unity.

### B. COMPASS and ${\text{MatriXX}}^{\text{Evolution}}$

**Figure 1:**
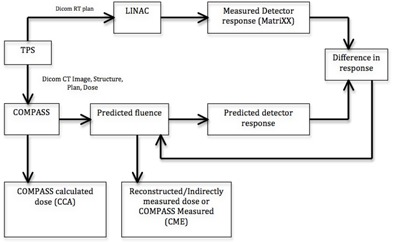
Schematic diagram for COMPASS reconstructed dose.

COMPASS is a novel 3D dosimetry QA system, which consists of 3D anatomy‐based dose verification software. It works in conjunction with ${\text{MatriXX}}^{\text{Evolution}}$ ionization chamber based array detector from IBA. COMPASS uses an independent dose calculation algorithm based on collapsed cone convolution. This allows pretreatment dose verification on CT images, including dose‐volume histogram. Similar to TPS, this COMPASS dose engine requires modeling of a virtual linac. To commission the beam model, the user needs to enter the linac specifications, depth dose curves, beam profiles, and output factors into COMPASS. Using the DICOM RT plan from TPS, COMPASS predicts the response independently by calculating the dose using a collapsed cone convolution algorithm. TPS‐generated treatment plans can be compared, evaluated, and verified against the COMPASS ‘computed’ dose from COMPASS using the commissioned dose engine (collapsed cone convolution) and fluence taken from the imported plan. These ‘ideal’ fluences can be adapted to the real delivery by means of a perturbative correction. This ‘indirectly measured’ dose is based on the measured signals with MatriXX array detector in COMPASS. The schematic diagram in Fig. 1 explains the workflow of indirectly measured or reconstructed dose. The collapsed cone dose engine in COMPASS calculates the dose by means of convolution–superposition method, which has been developed by Ahnesjö.^(6)^ Dose calculation consists of a two‐step process: first the total energy release in matter (TERMA) is calculated; in the second step, the TERMA is convolved with point spread kernels in (usually anisotropically distributed) directions which represent the whole surrounding cone in space. The collapsed cone algorithm is superior to the pencil beam algorithm as it takes into account the lateral energy transport and the influence of inhomogeneity.

### C. ${\text{MatriXX}}^{\text{Evolution}}$ detector

The ${\text{MatriXX}}^{\text{Evolution}}$ detector was used in conjunction with COMPASS verification software for 3D dosimetry. This detector consists of 1020 vented plane parallel ionization chambers with diameter of 4.5 mm and 5 mm height. The chamber volume is 0.08 cc and the distance between two chambers is 7.62 mm from center to center. To investigate the potential of 3D dosimetry particularly for stereotactic dose deliveries, 2D fluence verification results were analyzed in terms of number of passing pixels using gamma method, which combines criteria for dose difference and distance to agreement.^(8)^ The resolution of the detectors raises the question of correct choice of the detector for stereotactic treatments due to its volume effect and the issue with lateral electronic equilibrium associated with small fields used in stereotaxy. To see the feasibility and resolution, the dose distribution was compared with radiochromic (GAFCHROMIC EBT) film dosimetry by Korevaar et al.^(9)^ It is obvious that film has a very high spatial resolution compared to the ${\text{MatriXX}}^{\text{Evolution}}$ array detector. Anyway, due to the uncertainties an inconvenience associated with film dosimetry, there is a strong demand to go for array‐based detectors for fast and accurate dosimetry. Moreover, compared to the novel 3D dosimetry, film and conventional MatriXX QA provides only a planar dose distribution.^9^, ^10^, ^11^, ^12^, ^13^

### D. TPS‐calculated vs. COMPASS‐computed

All these 25 patients CT images, RT Structures, RT Plans, and RT doses were exported to COMPASS system through DICOM network. Like in the imported DICOM RT plans from the TPS, COMPASS computation was performed for all patients with a calculation grid resolution of 2 mm. Each plan was analyzed quantitatively based on dose‐volume histogram metrics, and quality was analyzed by gamma evaluation method (Low et al.^(8)^).

### E. TPS‐calculated vs. COMPASS‐reconstructed dose

COMPASS calculates the reconstructed dose on CT images from the delivered fluence using a resolution of 2 mm. COMPASS calculates the ideal fluence from the RT Plan input and the commissioned beam model, and this ideal fluence is used in the dose calculation functionality based solely on plan input (the ‘computed’ dose). The native resolution of the MatriXX chamber array, however, is 7.62 mm. Therefore, this resolution is not sufficient to directly calculate the real delivered fluence with an appropriate accuracy for accurate plan verification. Therefore, the algorithm in COMPASS uses a combination of ‘ideal’ and ‘measured’ fluences to determine the ‘real’ delivered fluence.

This algorithm first determines the signal expected in MatriXX by using a hard‐coded Monte Carlo phase space table, which takes into account the spatial and energetic response function of the detector. This signal, the ‘response’, is then compared with the measured response. The response difference is then used to adapt the ‘ideal’ fluence to the response difference. This perturbative correction yields a very good approximation of the really delivered fluence, yet maintaining, to a large extend, the higher resolution of the calculation grid compared to MatriXX native resolution. This method is very appropriate to find MLC positional errors, like MLC movement influenced by gravity in dynamic rotational plans. Such deviations can be traced very accurately by the response correction method in COMPASS. The COMPASS workflow and algorithm is explained in detail by Boggula et al.^1^, ^5^ and Godart et al.^(14)^ The ‘reconstructed dose’ is then calculated from the measured fluence using the COMPASS CC superposition algorithm. This ‘reconstructed’ dose has been computed for all of the patient plans and compared with the dose generated by the TPS.

### F. COMPASS measurement setup

**Figure 2:**
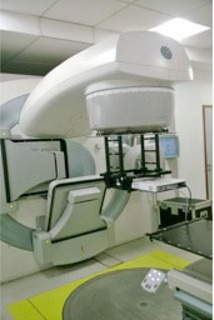
MatriXX with gantry mount.

COMPASS software version 2.03 was used for measurements using ${\text{MatriXX}}^{\text{Evolution}}$ detector. As shown in Fig. 2, the MatriXX detector was mounted on the gantry using a specially designed holder for Elekta linac. This holder allows lateral and longitudinal adjustments by means of micrometer screws. The major rotational correction was done by aligning the central axis cross hairs with the detector before fixing the buildup. Either 5 cm or 2 cm buildup on top of the detector can be used, and in this study we have applied 2 cm of buildup. Source‐to‐detector distance in the gantry mount is 100 cm. A gravity‐based angle sensor fixed at the gantry was used and connected through the RS232 interface. This inclinometer prompts the respective gantry angles to COMPASS software for each frame acquired during measurements. The gantry angle sensor must be calibrated at 0° and 90° gantry position before measurement takes place. MatriXX was connected to the computer running the COMPASS verification software through Ethernet connection. The RT Plan was loaded for each patient and the modeled linac was selected before measurement. The detector must be commissioned for each change in the measurement setup to account for the setup uncertainties and output errors. A preirradiation of 1000 MU was given as warm‐up. Small, medium, and large square field sizes were exposed to check the detector offset and rotational errors within user‐defined tolerances. Absolute dose calibration was done by entering the known dose for reference field size at reference depth and measuring the number of ADC counts for 100 MU. This commissioned detector setup is used for every patient's measurements.

All the 25 TPS‐generated treatment plans were scheduled in record and verification system MOSAIQ version 2.01 and taken in quality assurance delivery mode. Measured fluence was saved and the reconstructed dose was calculated off‐line.

### G. 2D fluence verification with MatriXX in MultiCube

The MatriXX detector was mounted in the MultiCube phantom, which was positioned on the patient couch and connected with OmniPro IMRT verification software (Iba Dosimetry GmbH). Couch and gantry angle were reset to zero as routine QA for IMRT. Absolute dose calibration was performed for known dose for $10.4\times 10.4\;{\text{cm}}^{2}$ field size. Source‐to‐detector distance (sensitive plane of the detector) was kept at 100 cm and source‐to‐surface distance (MultiCube phantom) as 89 cm. Each measurement was saved in .opab format and analyzed off‐line. A QA plan was generated for every patient in Monaco planning system by calculating the dose to the scanned MultiCube phantom with MatriXX detector. The isocentric coronal dose planes were exported to OmniPro IMRT system through the network. To verify the absolute dose at central axis, the isocentric dose was noted for each plan and compared with measured absolute dose from MatriXX measurements. The coronal dose plane was normalized to 100% during import into OmniPro IMRT software. The dataset 1 in the verification software represents the measured dose plane and dataset 2 comes from the TPS dose plane. The TPS dose plane was resampled to MatriXX native grid resolution of 7.62 mm in order to get the correct reference data to be compared with the MatriXX measurements. Subsequently the resolution was interpolated to 1 mm for both measured and TPS dose planes in order to facilitate data analysis. The dose distributions were normalized to 100% at either to the maximum or an appropriate cursor position before comparing the planar dose distribution (without touching the absolute calibration). The number of passing pixels was analyzed for each patient using 2D gamma evaluation criteria of 3 mm and 3% as DTA and dose difference.^9^, ^10^, ^11^, ^12^, ^13^

### H. Absolute point dose verification

**Figure 3:**
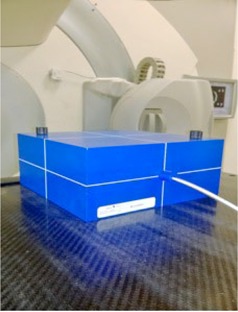
Stereotactic dose verification phantom (SDVP).

Using the Standard Imaging Slimline A16 microchamber of 0.007 cc collecting volume and SuperMAX 1000 plus electrometer, the absolute dose was measured in a stereotactic dose verification phantom, as shown in Fig. 3. The QA plans were generated for the scanned stereotactic dose verification phantom with the pinpoint chamber inserted. Source‐to‐detector distance was kept at 100 cm and source‐to‐surface distance as 95 cm. The overall delivery accuracy was verified by comparing the measured absolute point dose with TPS calculated and MatriXX measured point doses. The difference was calculated by keeping the measured dose from pinpoint chamber as reference value.

### I. Treatment plan analysis

All the 25 plans were generated in Monaco TPS, and to score the plan quality the dosimetry indices of CN, CI, GI, TC, $D_{95}$, and critical organ doses were evaluated. Conformity number is the ratio between target coverage and conformity index. Conformity index was calculated from the ratio of total volume receiving the 100% prescription dose to target volume receiving prescription dose. The target coverage was calculated from the ratio of target volume receiving the 100% prescription dose to total target volume. The gradient index was calculated from the ratio of volume of 50% prescription isodose to volume of 100% prescription isodose. The goal was that all the plans should achieve at least 95% of target volume receiving 100% of prescription dose. The analysis was varied for different sites, based on the dose‐volume histogram metrics.^15^, ^16^, ^17^

### J. Dosimetric comparison

**Figure 4:**
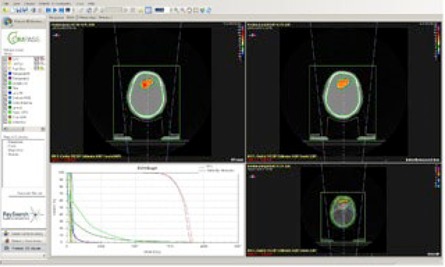
Comparison between TPS‐calculated vs. COMPASS measured.

Three‐dimensional anatomy‐based dosimetric comparison was done using the dose‐volume histogram function available in COMPASS verification software. For all the 25 patients the following comparisons were made. First, the difference between TPS calculated and CME dose distribution was analyzed in 3D. As a second evaluation, the doses calculated in the TPS and from CCA dose with collapsed cone algorithm were compared. Figure 4 shows the comparison between the TPS calculated and CME. In order to check the feasibility of 3D dosimetry and highlight the potential of anatomy‐based 3D dosimetry in particular for stereotactic hypofractionated dose delivery, the 2D fluence was verified by MatriXX /MultiCube dosimetry as the third type of measurement. In order to find the overall delivery accuracy, the absolute point dose was compared with TPS and MatriXX measurement.

### K. Gamma evaluation

The quantitative and qualitative analyses were performed based on the proven gamma evaluation method by Low et al.^8^, ^18^ The quantitative analysis in this study was based on the gamma pass–fail criteria of 3 mm for distance to agreement and 3% for dose difference.^8^, ^18^

## III. RESULTS

Treatment plans were evaluated by dose‐volume histograms and different dosimetric indices. The results of treatment plan analysis are shown in Table 1 for 25 patients. The averages of five patient results were taken. Each plan has achieved coverage of 95% of tumor volume receiving 100% of prescribed dose. All the data are presented in percentage of mean dose and standard deviation. The conformity number was calculated from the ratio of target coverage and conformity index in order to find the dose gradient. For each case, mean dose and standard deviation of $D_{95}$ was observed as 100%±0.02‐0.05 and the target mean dose as >112% was greater than unity. The closer the CI is to unity, the better is the conformity. The observed value was greater than 1.15 in all cases. The CN is calculated from the ratio of target coverage to conformity index, and avoids the misinterpretation caused by different target coverage. Conformity numbers were smaller than unity and closer to unity (which represents better conformity and target coverage). The gradient index is defined by the ratio of V50 to V100 and shows the dose falloff in normal structures. Values observed were less than 4.95±1.50SD. The given critical organ doses were achieved in each plan. Table 2 shows the each PTV volumes in cc for 25 cases.

**Table 1:** Monaco treatment plan analysis

*Site*	*D95 (%)*	*Target Mean Dose (%)*	CI(Mean±SD)	CN(Mean±SD)	GI(Mean±SD)
Brain	100±0.03	112.3±6.02	1.29±0.20	0.748±0.12	4.95±1.50
Head & Neck	99.98±0.043	114.77±6.8	1.20±0.09	0.788±0.06	4.24±0.58
Thorax	100±0.020	113.61±5.1	1.26±0.31	0.783±0.15	4.28±1.61
Abdomen	99.98±0.038	112.22±2.55	1.15±0.04	0.820±0.03	4.29±0.31
Spine	100.02±0.059	116.88±3.36	1.21±0.06	0.782±0.04	4.89±1.42

COMPASS offers various statistical analysis tools such as average dose, dose at volume, volume at dose, volume at gamma, and average gamma. TPS‐calculated plans were compared with CME and CCA data based on the following dosimetric indices derived from COMPASS dose‐volume histogram: for target volumes, dose to 95% target volume, average dose, and average gamma to target volume were compared in all cases.

Tables 3 and 4 show the comparison between CME versus TPS‐calculated (Table 3) and CCA versus TPS‐calculated (Table 4) values for target volumes. The dosimetric metrics of $D_{95}$, average target dose, and average gamma were taken to analyze the dose distribution agreement inside the target volume between the TPS versus CME and calculated values. $D_{95}$ were noted for each site and case, and the average difference and standard deviation was calculated. Similarly, average target dose and average gamma were analyzed. In Table 3, for all 25 cases CME dose to 95% volume shows a maximum deviation of 2.62%, compared with TPS‐calculated dose. The maximum standard deviation of 1.72% was observed in head and neck cases. For brain cases and CME versus TPS doses, an average deviation of 0.048%±0.696%,0.54%±0.747%,0.37%±0.086% was observed for $D_{95}$, average target dose, and average gamma, respectively. Maximum standard deviations of 1.720% and 1.373% were observed for $D_{95}$ and average target dose, respectively, compare to for other sites in CME versus TPS‐calculated.

**Table 2:** Size of each target volumes

	*Brain PTV vol. (cc)*	*Head & Neck PTV vol. (cc)*	*Thorax PTV vol. (cc)*	*Spine PTV vol. (cc)*	*Abdomen PTV vol. (cc)*
Case 1	29.87	54.65	113.159	76.46	82.68
Case 2	13.98	18.201	134.576	30.79	14.01
Case 3	15.73	38.024	102.418	64.46	46.36
Case 4	88.46	39.448	196.418	26.21	88.03
Case 5	31.71	55.74	16.691	69.23	15.07

**Table 3:** Comparison of COMPASS measured vs. TPS‐calculated values for target volume (% dose difference)

		$D_{95}$ (%)	*Average Target Dose (%)*	*Average Gamma*
*Brain*	Case1	‐0.13	‐0.2	0.28
	Case2	0.58	1.41	0.47
	Case3	0.92	1.01	0.45
	Case4	‐0.8	‐0.27	0.31
	Case5	‐0.33	0.76	0.33
	Average	0.048	0.54	0.37
	STD	0.696	0.747	0.086
*Head & neck*	Case1	1.66	0.36	0.25
	Case2	‐1.31	‐0.61	0.3
	Case3	1.99	0.54	0.29
	Case4	‐1.44	‐1.92	0.34
	Case5	1.61	1.75	0.38
	Average	0.502	0.024	0.31
	STD	1.720	1.373	0.010
*Thorax*	Case1	0.52	‐0.27	0.24
	Case2	0.66	0.28	0.25
	Case3	0.2	‐0.27	0.3
	Case4	0.78	0.29	0.27
	Case5	0.85	0.3	0.28
	Average	0.60	0.07	0.27
	STD	0.257	0.307	0.0239
*Abdomen*	Case1	‐2.62	‐1.23	0.41
	Case2	‐0.19	‐1.39	0.41
	Case3	‐1.44	‐1.47	0.42
	Case4	‐0.18	‐0.31	0.26
	Case5	0.55	‐1.11	0.47
	Average	‐0.78	‐1.10	0.39
	STD	1.255	0.464	0.079
*Spine*	Case1	1.45	1.07	0.45
	Case2	‐1.26	‐0.99	0.29
	Case3	‐0.48	‐1.06	0.35
	Case4	‐0.29	‐0.65	0.33
	Case5	0.33	‐0.88	0.45
	Average	‐0.05	‐0.50	0.37
	STD	1.013	0.892	0.0727

**Table 4:** Comparison of COMPASS‐calculated vs. TPS‐calculated for target volumes

		$D_{95}$ *(%)*	*Average Dose (%)*	*Average Gamma*
*Brain*	Case1	0.07	0.15	0.15
	Case2	0.06	0.17	0.16
	Case3	‐0.3	‐0.22	0.18
	Case4	0.03	0.03	0.15
	Case5	0.24	‐0.1	0.17
	Average	0.02	0.006	0.162
	STD	0.197	0.166	0.0130
*Head & Neck*	Case1	0.13	‐0.41	0.2
	Case2	0.35	‐0.29	0.24
	Case3	‐1.61	‐0.87	0.29
	Case4	0.7	0.72	0.24
	Case5	0.33	0.36	0.2
	Average	‐0.02	‐0.098	0.234
	STD	0.912	0.634	0.037
*Thorax*	Case1	0.48	‐0.64	0.32
	Case2	0.19	‐0.24	0.26
	Case3	0.28	‐0.48	0.28
	Case4	0.93	0.56	0.38
	Case5	0.56	0.38	0.87
	Average	0.488	‐0.084	0.422
	STD	0.288	0.529	0.255
*Abdomen*	Case1	0.28	0.66	0.37
	Case2	0.94	0.52	0.56
	Case3	1.41	1.26	0.3
	Case4	1.24	1.45	0.31
	Case5	0.88	0.76	0.87
	Average	0.95	0.93	0.482
	STD	0.433	0.403	0.241
*Spine*	Case1	‐0.25	‐0.89	0.29
	Case2	‐1.19	‐0.87	0.22
	Case3	‐0.52	‐1.37	0.32
	Case4	‐0.88	‐2.82	0.31
	Case5	‐0.79	‐2.51	0.25
	Average	‐0.726	‐1.692	0.278
	STD	0.358	0.917	0.042

Similar maximum standard deviations were observed for the CCA versus TPS‐calculated values for head and neck cases. But still the individual deviations observed are well within clinically acceptable limits compared to TPS‐calculated values.

Table 5 shows the comparison between TPS‐calculated versus CME and CCA doses for critical organs at risk. Dose to 2% of volume was taken as maximum dose for serial organs like brain stem, optic chiasm, left and right optic nerves, spinal cord/thecal sac, and esophagus. Per the institutional protocol applied in our institution, the dose to 50% volume was analyzed for right and left parotid, mandible, oral cavity, right and left lung, duodenum, right and left kidney, stomach, and liver. In addition, dose to 20% volume in right and left lung were analyzed. In the low‐dose region (less than 4 Gy), maximum deviation of 30% between TPS‐calculated versus CME and CCA for organs like eyes was observed. Critical organ doses for heart and esophagus were observed only in one case out of five patients. Dose to 50% volumes has shown larger differences in many critical organs at risk compared to the situation for higher doses to smaller volumes

**Table 5:** COMPASS measured and COMPASS‐calculated vs. TPS‐calculated doses for organ‐at‐risk volumes

*Site*	*TPS vs. CME (%)*	*TPS vs. CCA (%)*
*Brain*		
Brain stem 2%	‐2.46±2.76	0.38±1.20
Optic Chiasm 2%	‐3.74±3.86	‐0.91±0.89
Right Optic Nerve 2%	‐1.53±0.88	‐2.00±0.50
Left optic Nerve 2%	‐1.41±2.27	‐1.70±0.29
Right Eye 100%	NS	NS
Left Eye 100%	NS	NS
*Head & Neck*		
Right parotid 50%	‐0.78±2.98	1.26±1.21
Left parotid 50%	‐1.12±3.82	2.52±1.53
Spinal cord 2%	0.116±3.02	2.82±3.02
Oral cavity 50%	‐1.55±1.82	0.28±2.12
Mandible 50%	‐2.63±3.18	‐1.50±1.20
*Thorax*		
Spinal cord 2%	2.58±2.02	3.01±3.64
Left Lung 20%	1.50±0.858	3.64±2.23
Left lung 50%	6.74±8.44	7.39±2.10
Right Lung 20%	1.84±0.763	2.84±2.75
Right Lung 50%	4.15±5.71	4.67±7.21
Ribs 2 %	‐0.84±1.86	‐1.26±0.39
*Abdomen*		
Duodenum 5%	0.93±4.7	4.45±3.33
Duodenum 50%	3.94±9.53	4.15±4.37
Stomach 5%	0.79±1.09	4.66±1.87
Stomach 50%	0.59±2.58	1.90±2.79
Right Kidney 5%	1.04±0.52	2.88±1.73
Right Kidney 50%	‐0.43±0.93	1.70±1.08
Left kidney 5%	0.57±1.07	5.24±1.49
Left Kidney 50%	‐0.44±3.45	11.13±6.02
*Spine*		
Thecal Sac 2%	2.21±2.60	0.30±1.70
Heart 20%	1.05	1.07
Esophagus 2%	3.09	1.82
Liver 30%	1.28±0.85	1.56±2.12
Liver 50%	13.4±8.05	14.54±13.12
Right Kidney 5%	2.41±1.81	1.33±1.16
Right Kidney 50%	3.18±19.3	4.0±22.52
Left Kidney 5%	1.46±1.61	0.92±1.69
Left Kidney 50%	14.34±16.9	30.26±17.52

NS = not significant.

Absolute point dose verification was performed using A16 chamber and MatriXX detector. For each plan, point dose was calculated separately for each detector on scanned plastic phantoms using Monaco TPS. The calculated treatment plans were delivered on A16 detector. The measured absolute doses are in very good agreement with the TPS calculated doses; maximum mean deviation of 1.80%±2.71%. Measurements with MatriXX detector with MultiCube phantom were shown larger deviation compare to A16 chamber. All measurements using the MatriXX detector show higher discrepancies (≥3%), except for the abdomen cases.

As a cross check of the 3D dosimetric comparison, routine patient specific QA of 2D dose verification was performed and the results were in accordance within clinically acceptable limits.

For all sites, the 2D gamma passing rate has shown an excellent agreement between the measured and TPS‐calculated dose distribution. The lowest passing rate of 98.798%±1.039% was observed for the head and neck cases.

## IV. DISCUSSION

It has been well recognized that stereotactic VMAT plan delivery requires a very comprehensive and stringent pretreatment quality assurance program, as both high modulation and hypofractionation are associated with higher risk to the patient.

There have been many studies published about the use of COMPASS in combination with MatriXX array detector for patient specific quality assurance. However, despite the advantages of 3D anatomy‐based dosimetric analysis, it is essential to assess the potential of MatriXX and COMPASS to verify the stereotactic dose delivery, as hypofractionation, small segment shapes, and VMAT are a challenge for the relatively low resolution array detector. The patient specific QA based on 2D array detectors is a clinically proven method for IMRT and VMAT using larger fields, but their geometrical resolution is very limited compared to film and EPID dosimetry.

The issue of limited detector resolution is handled in COMPASS in a very innovative way: by introducing a perturbative correction based on the difference between measured and predicted responses of the detector. The fluence is not entirely taken from the measurement of the detector, as the resolution would be limited to the native detector resolution. To overcome this detector resolution limitation, COMPASS uses a Monte Carlo‐generated response function (both spatial and spectral response) for each individual ion chamber. Together with the ‘ideal’ fluence derived from plan and linac head model (and computed within a 2 mm grid), this can be used to predict exactly the expected signal in each pixel for ‘perfect’ delivery. The difference between ‘ideal’ and ‘real’ signal is the input used for the determination of the real fluence. This fluence has a zeroth order term (the ‘ideal’ fluence calculated in 2 mm grid), first order term (scaling correction of this term, again in 2 mm resolution), and higher order residual term (e.g., differences caused by malfunction of a single leaf or group of leaves), the latter obviously in the native MatriXX resolution (7.62 mm or 10 mm projected to the isocenter, respectively, depending on the gantry mount used). The measurement‐based correction method was extensively discussed in the COMPASS ‘white paper’ by Narloch,^(19)^ and readers are requested to refer to this paper for in‐depth understanding of the measurement based correction method. As the results from conventional QA methods using film, EPID, and 2D arrays are difficult to correlate to a predicted patient outcome, COMPASS takes the fluence determined according to the above‐mentioned procedure, together with the patient's planning CT, and reconstructs the three‐dimensional dose distribution in the patient using a collapsed cone superposition algorithm. This gives a much deeper insight into the expected treatment outcome by allowing an interpretation based on patient's segmentation and DVHs.

Moreover, accurate and meaningful pretreatment delivery verification is of utmost importance in stereotactic hypofractionated dose delivery, as it has a higher potential of harming patients. The potential of predicting the hypofractionated doses for stereotactic fields by COMPASS is an appropriate tool to satisfy the demand for comprehensive and sensitive 3D dosimetry and allows a quantitative analysis for such VMAT deliveries.

Boggula et al.^(1)^ using the Ergo++ treatment planning (Elekta) system did similar work for intensity‐modulated arc therapy. The optimization in ERGO++ uses an arc modulation optimizer algorithm (AMOA). AMOA is an aperture‐based optimization algorithm that calculates the monitor units per control point. PBS algorithms are still implemented in many TPS, but due to the inherent limitation of the PB algorithm in taking into account the inhomogeneity and calculating the lateral energy transport correctly, these are currently no more clinically acceptable, as more precise dose calculation algorithms like collapsed cone convolution–superposition and Monte Carlo are now commercially available. Therefore, this study aims at evaluating quantitatively the stereotactic volumetric‐modulated arc therapy calculated with Monte Carlo algorithm against COMPASS, which applies the collapsed cone convolution algorithm proposed by Ahnesjo.^(6)^ In the same paper by Boggula, the 2D array detector (off‐line) and 2D transmission detector (online) for Monte Carlo calculated IMRT prostate cases were evaluated, but this study was limited to five prostate cases with conventional IMRT technique. The 2D transmission detector, which was evaluated by Boggula and colleagues, is not yet clinically released. Therefore, the COMPASS verification software in combination with 2D array detector MatriXX is the state‐of‐the art tool to verify stereotactic VMAT plans, yielding a quantitative, 3D anatomy‐based dosimetry. In order to explore the potential of this combination for the delivery verification of modulated stereotactic Monte Carlo‐calculated VMAT plans, in this study such plans for five major clinical sites (brain, head and neck, thorax, abdomen, and spine) were analyzed. The potential of highlighting the delivery errors and showing the errors on patient CT images were evaluated quantitatively using various dose‐volume–based histogram metrics. The proven and clinically accepted method of gamma evaluation was adopted in this study to benchmark the delivery accuracy of stereotactic Monte Carlo‐calculated VMAT plans. This study was carefully designed to experimentally verify the potential of 3D dosimetry in highlighting the delivery errors at different localizations with real clinical intricacies associated to stereotactic VMAT plan delivery. Though the conventional point dose QA and planar dosimetry can give clinically acceptable passing rates, this integral metrics might not be sensitive to small‐volume errors for special ROIs. The evaluation based on 3D anatomy and dose‐volume histogram metrics, which can achieve a real correlation to the expected patient outcome, can be seen as of paramount importance in the best practice of stereotactic VMAT delivery.

However, all the routine patient‐specific QA methods might show clinically acceptable passing rates but insufficient outcome when analyzing the data on a DVH‐based metrics.^(13)^ But it will not interpret the dosimetric errors on patient's anatomy. Some commercially available solutions for plan QA do have 3D functionality, but the calculation uses a perturbation of the original TPS dose distribution.

Only COMPASS 3D dosimetry uses a sufficiently accurate algorithm for the dose determination which is independent from the TPS. Therefore, it can identify also weaknesses in the TPS commissioning and algorithm or errors due to wrong parameter settings in the calculation (e.g., low number of seeds in a Monte Carlo calculation, mismatch between dose‐to‐water and dose‐to‐material). It can directly show the dosimetric errors on patient's anatomy in order to avoid any random, systematic or ‘catastrophic’ errors. Such a correlation between the TPS and measured dosimetric errors are of utmost importance in the delivery of stereotactic VMAT plans. The conventional QA process, using 2D gamma passing rates, is not sufficient here as it can give both false negatives (relevant error not detected) and false positives (bad passing rate for an acceptable treatment). The 3D DVH‐based analysis gives the user the option to track where the problem arises and what are the possible consequences.

Table 1 shows the dosimetric indices of treatment plan analysis. In all 25 cases, 95% of tumor volume was planned to deliver 100% of prescription doses, and the mean dose observed was more than 112% of prescription dose in all sites. Per our institutional protocol, we require less than 1.5 conformity index for clinical cases. We have observed a conformity index minimum of 1.2 and a maximum of 1.29±0.20SD, a conformity index of less than 1.3 was noticed in all cases. Zhang et al.^(16)^ have shown that poor target coverage may yield a better conformity index. Therefore, the inclusion of target coverage in the conformity number compensates this effect. The conformity numbers were calculated from the ratio of target coverage to conformity index and a maximum of 0.820±0.03SD was noticed. The value that is closest to unity corresponds to best conformity and coverage.

The rapid dose falloff surrounding the tumor was calculated from the gradient, defined by the ratio of V50 to V100. The maximum value for this gradient index was observed as 4.95±1.50. From our observed results and published articles on plan quality and efficacy for Monte Carlo‐calculated stereotactic VMAT plans, one can conclude that Monaco TPS gives the best possible treatment plans for stereotactic delivery.

Tables 3 and 4 show the list of comparison between the TPS‐calculated versus CME and TPS‐calculated versus CCA results based on various dose volume metrics. For target volume $D_{95}$, average target dose and average gamma were compared with values from TPS. The comparison of dose to 95% volume between TPS versus CME and CCA was showing the quality of the dose distribution delivered inside the target volume. In addition, the results for average target dose and average gamma prove that this 3D dosimetric verification is very robust. The observed results confirm that, inside the target volume, 100% of the prescription dose was delivered to 95% of target volume with a low standard deviation of <2%; maximum deviation was observed for head neck cases, where the combination of inherent complexity of the shape with very inhomogeneous tissue density results in the biggest challenges for planning and delivery. Table 4 shows the results of comparison between TPS versus CCA dose for target volumes for similar parameter of $D_{95}$, average target dose, and average gamma. The results show some deviations (up to ~2%). Those differences cannot be attributed to intrinsic calculation accuracy differences between Monte Carlo and collapsed cone algorithms, as they are not present in the CME outcome. Most probably they can be attributed to weaknesses in the commissioning of COMPASS, as the analysis of the measured beam tends to compensate such setup errors (the measured values do not depend on parameters like leaf transmission, source shape, and dosimetric leaf gap). Our results show that one can use CCA dose verification as a tool for secondary dose calculation verification in stereotactic delivery with sufficient accuracy.

For critical organs, Table 5 shows a comparison between the TPS versus CME and CCA. Larger deviations were observed for lower dose to higher volumes. It can be seen that maximum doses to 2% volumes are matching very well compared to TPS‐calculated values. Larger deviations, however, are observed for doses to 50% volume in accounting scattered dose calculation and low dose spillages. Doses less than 4 Gy are not showing any relevant deviation compared to TPS versus CME and CCA. If the critical organs are located very close to the tumor volume and minimum dose to critical organs is more than 5 Gy, CME and CCA have shown significant deviations.

In all 25 cases, the maximum doses were matched well within acceptable limit of 3%. It is important to note that if the total doses are less than 3–5 Gy and volume is relatively large, then small differences in doses can show huge relative differences, which is not clinically relevant. In such situations, the comparison may interpret incorrectly to correlate with clinical consequences. Table 5 shows in spine cases the difference for 50% of liver was showing around 13.4%±8.05% and 50% of left kidney was showing 14.34%±16.9%; whereas, dose to 30% volume of liver was matching well with TPS‐calculated values of <2%.

Table 6 lists the results for absolute dose verification using the detector A16. It shows an excellent agreement between the TPS‐calculated and ion chamber measured values. For this particular measurement in stereotactic fields, the size of the detector and the resulting volumeintegration effect is very important. It can be seen that measuring the absolute dose using the MatriXX detector in these cases may compromise the accuracy level. Since the design of detector arrangements in MatriXX array, where there is no detector at central axis, and averaging dose always from the four detectors in quadrants could cause the dose deviation effectively in absolute dose comparison. Therefore, for small segments shapes, the A16 chamber gave the better result comparing to MatriXX detector in the MultiCube phantom. Absolute dose and fluence verification measurement using MatriXX/MultiCube will reduce the verification time significantly, but point dose verification in this case shows up to 3% of variations compared to the small volume detector. Partial volume irradiation leads to lateral electronic disequilibrium where the response within the detector is affected due to volume averaging effect in particular high‐dose gradient region.^(20)^ It could be verified using the other array detectors to see the collection efficiency for stereotactic fields where the detector is located at central axis. Though the MatriXX detector is widely accepted to verify fluence and point doses for IMRT delivery, it is mandatory to validate the collection efficiency for modulated stereotactic fields where the narrow and elongated fields are more. Partially irradiating the detector multiple times is mainly underdosing the absolute point dose accuracy at central axis. This is not the scenario when we use COMPASS software for 3D dosimetry verification, since COMPASS uses the Monte Carlo‐derived response function for individual detector and applies the response correction method, which overcomes the limitations of 2D measurements.

**Table 6:** Absolute point dose verification

*Sites*	*MatriXX Measured vs. TPS (%)*	*A16 Chamber vs. TPS (%)*
Brain	3.2±3.0	‐0.8±1.2
Head & Neck	3.6±2.5	1.8±2.7
Thorax	2.9±2.6	‐0.7±1.6
Abdomen	0.7±0.9	0.1±0.6
Spine	3.5±3.0	1.3±0.8

Table 7 shows 2D fluence verification with 99% of pixels passing with gamma criteria of 3% /3 mm for all sites. On the other hand, the 3D dosimetry results show a deviation for target coverage, as well as for critical organ doses. This fact — the gamma passing rates from 2D fluence verification do not show any deviation (false negative outcome of the verification) — clearly underlines the need of correlating dosimetric data with patient anatomy. The 2D verification alone could in the worst case leave critical errors unidentified (false negatives), or identify acceptable plan deliveries as having excess discrepancies (false positives) which, though being obviously not dangerous for the patient, could create unnecessary delays in the workflow.

Even if a plan is correctly rejected in the 2D gamma metrics, it is not clear where this discrepancy will affect the patient and how serious the possible consequences could be. In the shown case (with 99% gamma passing rate), the results from 3D dosimetry, however, reveal a deviation to target volume coverage, as well as a deviation for critical organs at risk.

It is obvious that the dose volume‐based analysis in the COMPASS software, which shows the interpretation of delivery errors within the patient anatomy, allows a quantitative assessment for the three critical situations: false negative, false positive, and correct positive — where it can help in understanding the potential harm to the patient and hence taking correct decisions for further procedure.

**Table 7:** 2D Fluence verification 3 mm/3%

*Sites*	*Number of Pixels Passed (%)*
Brain	99.7±0.3
Head & Neck	98.8±1.0
Thorax	99.3±0.5
Abdomen	99.3±0.3
Spine	99.7±0.2

## V. CONCLUSIONS

The quantitative analysis of stereotactic VMAT plans using 3D dosimetry has shown its potential of identifying the delivery errors in a complex hypofractionated treatment modality. COMPASS 3D dosimetry is an efficient tool for pretreatment patient‐specific QA, as there is no need to place a phantom on the patient couch or for the creation of a hybrid QA plan. The result obtained in this study show that COMPASS can be used as a sensitive and meaningful quality assurance tool for stereotactic dose verification. It avoids the shortcomings of ‘classical’ 2D verification schemes and can be used regardless of the limited detector resolution also for small field sizes.

## Supporting information

Supplementary Material

## References

[1] Boggula R , Lorenz F , Mueller L , et al. Experimental validation of a commercial 3D dose verification system for intensity‐modulated arc therapies. Phys Med Biol. 2010;55(19):5619–33. 20826904 10.1088/0031-9155/55/19/001

[2] Fiandra C , Fusella M , Giglioli FR , et al. Comparison of Gafchromic EBT2 and EBT3 for patient‐specific quality assurance: cranial stereotactic radiosurgery using volumetric modulated arc therapy with multiple noncoplanar arcs. Med Phys. 2013;40(8):082105. 23927342 10.1118/1.4816300

[3] Mans A , Remeijer P , Olaciregui‐Ruiz I , et al. 3D dosimetric verification of volumetric‐modulated arc therapy by portal dosimetry. Radiother Oncol. 2010;94(2):181–87. 20089323 10.1016/j.radonc.2009.12.020

[4] Diot Q , Kavanagh B , Timmerman R , Mifflen M . Biological‐based optimization and volumetric modulated arc therapy delivery for stereotactic body radiation therapy. Med Phys. 2012;39(1):237–45. 22225293 10.1118/1.3668059

[5] Boggula R , Jahnke L , Wertz H , Lohr F , Wenz F . Patient‐specific 3D pretreatment and potential 3D online dose verification of Monte Carlo calculated IMRT prostate treatment plans. Int J Radiat Oncol Biol Phys. 2011;81(4):1168–75. 21093168 10.1016/j.ijrobp.2010.09.010

[6] Ahnesjo A . Collapsed cone convolution of radiant energy for photon dose calculation in heterogeneous media. Med Phys. 1989;16(4):577–92. 2770632 10.1118/1.596360

[7] CMS . Monaco training guide, version 2.0.31. Maryland Heights, MO: CMS Inc.; n.d.

[8] Low DA , Harms WB , Mutic S , Purdy JA . A technique for the quantitative evaluation of dose distributions. Med Phys. 1998;25(5):656–61. 9608475 10.1118/1.598248

[9] Korevaar EW , Wauben DJ , van der Hulst PC , Langendijk JA , Van't Veld AA . Clinical introduction of a linac head‐mounted 2D detector array based quality assurance system in head and neck IMRT. Radiother Oncol. 2011;100(3):446–52. 21963288 10.1016/j.radonc.2011.09.007

[10] Herzen J , Todorovic M , Cremers F , et al. Dosimetric evaluation of a 2D pixel ionization chamber for implementation in clinical routine. Phys Med Biol. 2007;52(4):1197–208. 17264380 10.1088/0031-9155/52/4/023

[11] Saminathan S , Manickam R , Chandraraj V , Supe SS . Dosimetric study of 2D ion chamber array matrix for the modern radiotherapy treatment verification. J Appl Clin Med Phys. 2010;11(2):3076. 20592695 10.1120/jacmp.v11i2.3076 PMC5719948

[12] Wagner D and Vorwerk H . Two years experience with quality assurance protocol for patient related Rapid Arc treatment plan verification using a two dimensional ionization chamber array. Radiat Oncol, 2011;6:21. 21342509 10.1186/1748-717X-6-21 PMC3049120

[13] Nelms BE , Zhen H , Tome WA . Per‐beam, planar IMRT QA passing rates do not predict clinically relevant patient dose errors. Med Phys. 2011;38(2):1037–44. 21452741 10.1118/1.3544657 PMC3188652

[14] Godart J , Korevaar EW , Visser R , Wauben DJ , Van't Veld AA . Reconstruction of high‐resolution 3D dose from matrix measurements: error detection capability of the COMPASS correction kernel method. Phys Med Biol. 2011;56(15):5029–43. 21772084 10.1088/0031-9155/56/15/023

[15] Wagner TH , Bova FJ , Friedman WA , Buatti JM , Bouchet LG , Meeks SL . A simple and reliable index for scoring rival stereotactic radiosurgery plans. Int J Radiat Oncol Biol Phys. 2003;57(4):1141–49. 14575847 10.1016/s0360-3016(03)01563-3

[16] Zhang GG , Ku L , Dilling TJ , et al. Volumetric modulated arc planning for lung stereotactic body radiotherapy using conventional and unflattened photon beams: a dosimetric comparison with 3D technique. Radiat Oncol. 2011;6:152. 22070866 10.1186/1748-717X-6-152 PMC3354344

[17] Paddick I and Lippitz B . A simple dose gradient measurement tool to complement the conformity index. J Neurosurg. 2006;105 (Suppl):194–201. 10.3171/sup.2006.105.7.194 18503356

[18] Low DA and Dempsey JF . Evaluation of the gamma dose distribution comparison method. Med Phys. 2003;30(9):2455–64. 14528967 10.1118/1.1598711

[19] Narloch N . On the clinically relevant detector resolution and error detection capability of COMPASS 3D plan verification [White Paper]. Schwarzenbruck, Germany: IBA Dosimetry GmbH; 2012.

[20] Fraser D , Parker W , Seuntjens J . Characterization of cylindrical ionization chambers for patient specific IMRT QA. J Appl Clin Med Phys. 2009;10(4):241–51. 10.1120/jacmp.v10i4.2923 PMC5720562 19918222

