# Supplementary material for: Quantitative evaluation of 3D dosimetry for stereotactic volumetric‐modulated arc delivery using COMPASS
Source: J Appl Clin Med Phys. 2014 Jan 7;16(1):192–207. doi: 10.1120/jacmp.v16i1.5128 (PMC5689974; doi:10.1120/jacmp.v16i1.5128)
Supplement: Supplementary file 1 — Supplementary Material [file ACM2-16-192-s001.pdf]

Dosimetry

OmniPro I'mRT System Version 1.7a

User's Guide

P-07-002-510-001 06

Notice

This User's Guide is an integral part of OmniPro I'mRT System and should always be kept at hand. Observance of the manual instructions is required for proper performance and correct operation of OmniPro I'mRT System.

OmniPro I'mRT System and its accessories must not be used for any other purpose than described in the accompanying documentation (intended use). Violation will result in loss of warranty.

IBA Dosimetry does not accept liability for injury to personnel or damage to equipment that may result from misuse of this equipment, failure to observe the hazard notices contained in this manual, or failure to observe local health and safety regulations.

IBA Dosimetry shall under no circumstances be liable for incidental or coincidental damage arising from use of the equipment described in this document.

No part of the accompanying documentation may be translated or reproduced without written permission of IBA Dosimetry, unless reproduction is carried out for the sole purpose to be used by several people in the same department.

The user must treat the accompanying documentation like any other copyrighted material. Especially, if part of the accompanying documentation is provided in electronic form, these files shall not be modified in any way. IBA Dosimetry and its suppliers retain title and all ownership rights to the accompanying documentation (either in electronic or printed form).

IBA Dosimetry GmbH

Bahnhofstrasse 5

DE-90592 Schwarzenbruck

Germany

Phone: +49 9128 607-0

Fax: +49 9128 607-10

www.iba-dosimetry.com

Last update: 2009-10-05

Table of Contents

1. Introduction 1
1.1. Intended use 1
1.2. Product Description 1
1.2.1. OmniPro I'mRT System 1
1.2.2. Supported Software 1
1.2.3. Supported Equipment 2
1.3. Product Documentation 6
1.3.1. OmniPro I'mRT System User's Guide 6
1.3.2. On-line Help 7
2. Health and Safety Information 9
2.1. OmniPro I'mRT Software 9
2.1.1. Responsibility 9
2.1.2. Data Sampling and Handling 10
2.2. MatriXX 12
2.2.1. Operator 12
2.2.2. Accessories 13
2.2.3. Environmental Requirements 13
2.2.4. Storage 13
2.2.5. Handling 14
2.2.6. Electrical Installations 14
2.2.7. Rating Label 14
2.2.8. EMC Precautions 14
2.2.9. Naming in the manual 18
2.3. Regulatory Requirements 19
2.3.1. OmniPro I'mRT 19
2.3.2. I'mRT MatriXX and MatriXX Evolution 19
2.3.3. Other Devices Supported by OmniPro I'mRT Software 19
2.3.4. Quality Management System 19
2.3.5. CE Marking 19
3. General Workflow for QA with MatriXX 21
3.1. Basic Preparations 21
3.1.1. Scanning of MatriXX in CT scanner 21
3.1.2. Calibration of the MatriXX Measurement System 22
3.2. Realization of the Measurement 22
3.3. Analysis 22
4. Hardware description 23
4.1. I'mRT MatriXX and MatriXX Evolution 23
4.1.1. Intended Use 23
4.1.2. Connection of I'mRT MatriXX and MatriXX Evolution 23
4.1.3. Technical Specifications 23
4.2. I'mRT MatriXX - Accessories 27

4.2.1. XY Tables 27
4.2.2. Build-up Plates....28
4.2.3. Backscatter Holder and Plates....28
4.2.4. Gantry Adapters....29
4.3. I'mRT QA / BIS and BIS 710 31
4.4. Vidar Scanner Installation 31
4.5. Kodak LS (Lumiscan) Scanner Installation 34
5. Installation....35
5.1. Installation of OmniPro I'mRT Software 35
5.1.1. Computer Requirements 35
5.1.2. OmniPro I'mRT Installation Utility 36
5.1.3. Test of the Installation....40
5.1.4. Registration of the Software....41
5.1.5. Removing OmniPro I'mRT 42
5.1.6. Reinstallation of OmniPro I'mRT....42
5.2. Computer settings for MatriXX 43
5.2.1. TCP/IP and IP Addresses....43
5.2.2. Direct Connection to a PC....44
5.2.3. Connection to a Local Network 52
5.2.4. MatriXX Firmware 56
6. Getting started in OmniPro I'mRT software 57
6.1. Outline of OmniPro I'mRT 57
6.1.1. Windows Environment 57
6.1.2. Screen Structure....57
6.1.3. Main Menu Bar....57
6.1.4. Main Toolbar....58
6.1.5. I'mRT QA / BIS Toolbar 59
6.1.6. I'mRT MatriXX Toolbar....59
6.1.7. Status Bar 59
6.1.8. Panes....59
6.1.9. Panes, Single Data Set....60
6.1.10. Panes during Plan Verification....60
6.1.11. Data Types....61
6.1.12. Open Data....62
6.2. Configuration of OmniPro I'mRT Software....63
6.2.1. Specify Units and Parameters....63
7. Setup of MatriXX for measurements....71
7.1. Setting up I'mRT MatriXX....71
7.1.1. Mounting I'mRT MatriXX....72
7.2. Setting up MatriXX Evolution 84
7.2.1. Setup with MULTICube....84
7.2.2. Setup with Gantry Holder....85
7.3. Preparation for Measurements for I'mRT MatriXX and MatriXX Evolution 86
7.3.1. Connections....86
7.3.2. Pre-irradiation 86
7.3.3. Warm-up Time 86
7.3.4. Gantry Angle Sensor (available with MatriXX Evolution)....87
7.4. I'mRT MatriXX and MatriXX Evolution Measurements....98

7.4.1. General 98
7.4.2. Calibration....98
7.4.3. Uniformity Correction Matrix for MatriXX....99
7.4.4. Add Correction Factors....100
7.4.5. Measurement Setup....110
7.4.6. Measurement....118
7.5. VMAT/RapidArc – Parameter setup....120
7.5.1. Hardware Setup....120
7.5.2. Setup Measurement Parameters....120
7.6. TomoTherapy - Specific Workflow....122
7.6.1. Preparation....122
7.6.2. Tomo specific setup....126
7.7. I’mRT Phantom Measurements....130
7.7.1. Dose Planning....130
7.7.2. Absolute Dose Measurement in I’mRT Phantom....130
7.7.3. Analysis....131
7.7.4. Dose Measurements Using Films in the I’mRT Phantom....132
7.8. Setup for I’mRT QA/BIS Measurements....135
7.8.1. General....135
7.8.2. Calibration....135
7.8.3. Measurement Setup....136
7.8.4. Measurement....142
7.9. Setup for Film Control Panel....144
7.9.1. Views and Panels....144
7.9.2. Menus....145
7.9.3. Scanning....145
7.9.4. Scanner Artifacts Correction....151
7.9.5. Scanner Calibration....153
7.9.6. Film Calibration....160
7.9.7. Display and Modification of Images....169
8. Data Import....177
8.1. Import of Dose Planes and Dose Cubes....178
8.1.1. DICOM Import....178
8.1.2. CMS Import....181
8.1.3. RTOG Import....182
8.1.4. CadPlan Import....184
8.1.5. Plato Import....186
8.1.6. Send to I'mRT Workspace....187
8.2. Fluence Import....187
8.2.1. CadPlan, Pinnacle, and CMS....188
8.2.2. DICOM Compensator Fluence....189
8.2.3. KonRad Fluence Import....191
8.2.4. DICOM RT Image Import....194
8.2.5. Enter Location....196
8.3. BrainLAB import....197
8.3.1. Patient Coordinate System....197
8.3.2. Individual Beams (“Fluence Maps”):....198
8.3.3. File Format....199
8.3.4. User Interface....199
8.3.5. Error Messages....199

8.4. Generic ASCII Import 200
8.4.1. Coordinate System 200
8.4.2. File Format....200
8.4.3. Example Files 206
8.4.4. File Fulfilling the Minimum Requirements 208
8.4.5. User Interface for ASCII and BrainLAB Import....209
8.4.6. Header Information for OmniPro I'mRT ASCII Files....210
8.4.7. Header Information for BrainLAB Files 211
8.4.8. Error Messages....211
8.5. Tomo Therapy ASCII import....213
9. Data Analysis 215
9.1. Convert Grid 215
9.2. Modifying Data....218
9.2.1. Add Constant Value....218
9.2.2. Rescale....218
9.2.3. CAX Correction....219
9.2.4. Move 220
9.2.5. Turn Array....221
9.2.6. Smooth....221
9.2.7. Flip Image 222
9.2.8. View ROI....223
9.2.9. Cut ROI....223
9.2.10. Change Plane 223
9.2.11. Filter....223
9.3. Macros 225
9.3.1. Define a Macro....226
9.3.2. Apply a Macro....227
9.4. Analyzing One Single Data Set....228
9.4.1. Isodoses....228
9.4.2. Profile....230
9.4.3. Build a 3D Cube....232
9.4.4. Play a Movie 232
9.4.5. Multiple Row Analysis....233
9.4.6. Time Based Analysis 235
9.4.7. Merge Fields....236
9.5. Plan Verification 2D 238
9.5.1. Compare Profiles 239
9.5.2. Compare Isodoses....239
9.5.3. Synchronized Rescaling....240
9.5.4. Histogram....241
9.5.5. Palette....243
9.5.6. Calculations 244
9.6. Plan Verification 3D 249
9.6.1. Difference to 2D....249
10. Data Output 251
10.1. Print Data....251
10.1.1. Preview and Print a Selected Pane....251
10.1.2. Preview and Print All Panes in the Window 252
10.1.3. Print Setup 254
10.2. Copy Data....254

10.3. Export Data ... 254
10.3.1. Export to ASCII ... 254
10.3.2. Export to BRI ... 257
10.3.3. Export to RT Image ... 258
10.3.4. Export to TomoTherapy ... 259
10.3.5. Export to OmniPro Accept ... 261
10.3.6. Export of Data in Graphic Format ... 261
10.4. Save Data ... 263
10.4.1. Save Measurement ... 263
10.4.2. Save Workspace ... 263
10.4.3. Save Your Options ... 263
10.4.4. Save Measurement Parameters ... 264
11. iViewGT Browser ... 265
11.1. Prerequisites ... 265
11.2. Browser ... 265
11.2.1. Installation ... 265
11.2.2. Login ... 266
11.2.3. iViewGT Browser Dialog ... 266
11.2.4. Close and Log Out ... 268
12. Maintenance ... 271
12.1. OmniPro I'mRT Software ... 271
12.1.1. ODBC Data Source ... 271
12.2. MatriXX ... 272
12.2.1. General ... 272
12.2.2. Cleaning ... 272
12.2.3. Repair ... 272
12.2.4. Recalibration ... 272
13. Troubleshooting ... 273
13.1. Hardware ... 273
13.1.1. MatriXX ... 273
13.1.2. Scanners, I'mRT Phantom, I'mRT QA, BIS 710 ... 274
13.2. Software ... 275
13.2.1. Installation ... 275
13.2.2. Database and Equipment Setup ... 275
13.2.3. Display of Data ... 276
14. Appendix ... 279
14.1. Algorithms ... 279
14.1.1. 1D Data ... 279
14.1.2. 2D Data ... 288
14.2. References ... 291
14.3. Glossary of Terms ... 293
14.4. Complaint Reports ... 297

1.Introduction

1.1. Intended use

The intended use of the OmniPro I'mRT system is to:

- verify the treatment plan and delivered dose of intensity modulated or static beams, prior to treatment
- verify the intensity maps during IMRT delivery, prior to treatment
- verify the absolute dose in given points for IMRT fields

1.2. Product Description

1.2.1. OmniPro I'mRT System

OmniPro I'mRT System consists of the OmniPro I'mRT software and various hardware supported by OmniPro I'mRT.

OmniPro I'mRT system can be configured in different ways, supporting (either in combination or stand alone):

- I'mRT MatriXX
- MatriXX Evolution
- I'mRT Phantom, utilizing film or single detectors
- 2D film digitizers, such as the Vidar scanner family
- I'mRT QA (BIS 2G)
- BIS 710
- OmniPro I'mRT only

NOTICE

IMPORTANT NOTICE

OBSOLETE PRODUCTS

The following products are not available for purchasing any longer, but are supported by the OmniPro I'mRT software:

- I'mRT QA (BIS 2G)
- BIS 710

1.2.2. Supported Software

Dose or fluence distributions calculated by a Treatment Planning system can be imported to OmniPro I'mRT, and compared with measured data.

Examples of data to be compared:

- Absolute dose in single points
- Intensity Maps
- Relative dose

There are tools to analyze both static and dynamic beams.

See also *8 Data Import*.

1.2.3. Supported Equipment

1.2.3.1. I'mRT MatriXX

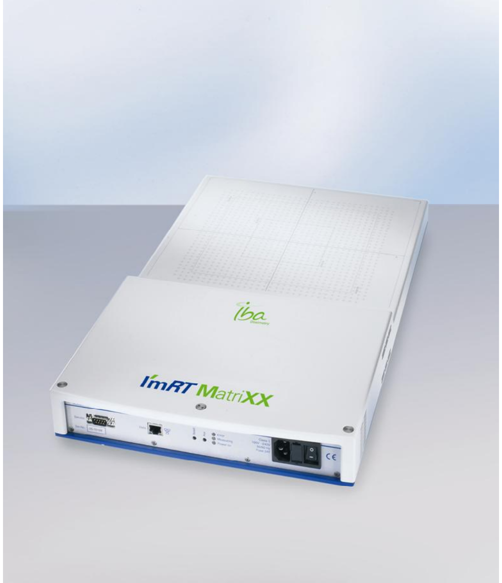

I'MRT MatriXX consists of a 1020 vented ion chambers array detector, arranged in 32 × 32 grid. When irradiated, the air in the chambers is ionized. The released charge is separated by means of an electrical field between the bottom and the top electrodes. The current, which is proportional to the dose rate, is measured and digitalized by a non-multiplexed 1020 channels current sensitive analog to digital converter.

The measured data are transmitted to a PC via a standard Ethernet interface, available on most PCs.

1.2.3.1.1. Product Development

I'mRT MatriXX is developed by IBA Dosimetry in collaboration with INFN and University of Torino, Italy.

The device is manufactured by

IBA Dosimetry GmbH

Bahnhofstrasse 5

DE-90592 Schwarzenbruck

Germany

Phone: +49 9128 607-0

Fax: +49 9128 607-10

www.iba-dosimetry.com

1.2.3.2. MatriXX Evolution

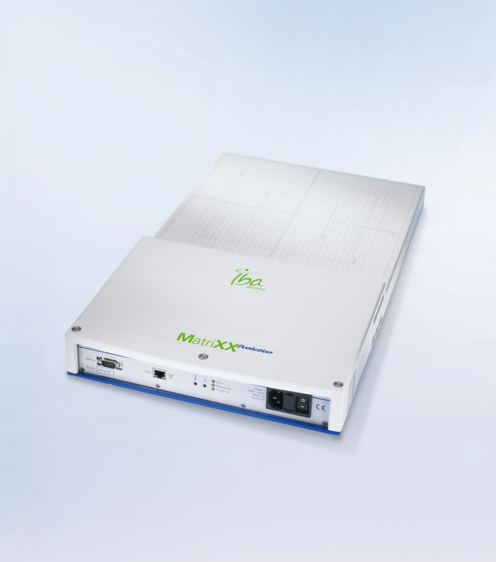

MatriXX Evolution is an optimized 2D digital verification system for rotational therapy techniques. It operates with the OmniPro I'mRT software for complete plan verification and QA of ImRT, IGRT, and rotational treatments.

1.2.3.3. MULTICube

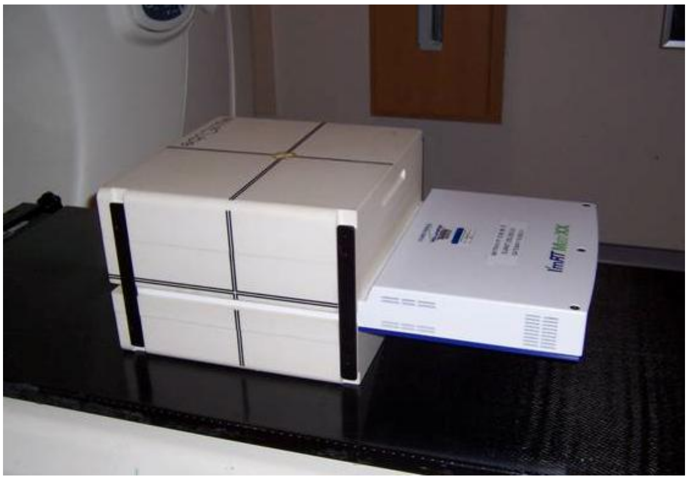

The Plastic Water ® phantom MULTICube is used together with I’mRT MatriXX or MatriXX Evolution for Rotational Dosimetry application.

The phantom is described in the user documentation delivered with the MULTICube.

Plastic Water® is a trademark of CIRS, USA.

1.2.3.4. I'mRT Phantom

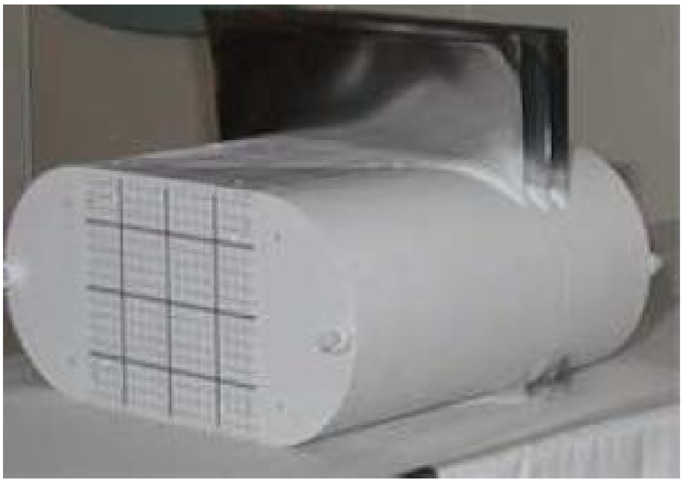

The I'mRT Phantom resembles the human body in shape and radiation properties. The cube, which can be used separately, resembles the human head.

After CT scanning of the phantom, a treatment plan is calculated, and the dose distribution data is imported into OmniPro I'mRT software. The I'mRT Phantom is then treated according to this plan, and the dose distribution or the absolute dose in singular points is measured, using films and ionization chambers respectively. The OmniPro I'mRT software will compare the measurement values with the calculated dose distributions.

Safety information, technical information, and instruction for use for I'mRT Phantom are found in I'mRT Phantom User's Guide.

1.2.3.5. Film Scanners

OmniPro I'mRT software supports the following film digitizers and scanners:

- Vidar®Systems Corporation

  VXR-16™

  VXR-16 DosimetryPRO™

  DosimetryPRO Advantage™

  DosimetryPRO Advantage (Red)™

  USB or SCSI interfaces can be used.

- Kodak (former Lumisys Inc.), e.g.:

  LS50, LS75, or LS85.

  DCB or SCSI interfaces can be used.

- External scanners, e.g. Twain scanners, for .tif and .dcm file import.

1.2.3.6. I’mRT QA

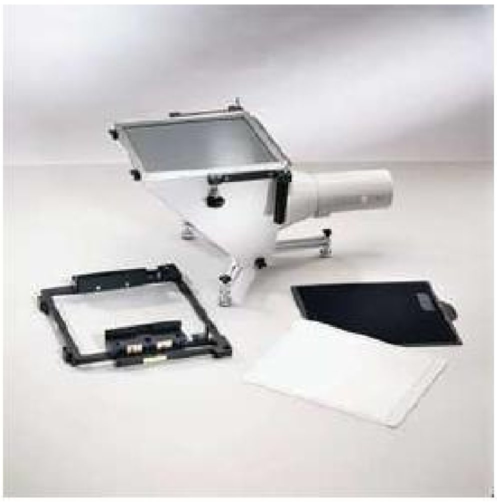

I'mRT QA is used to measure intensity maps in 2D with high spatial resolution, both in static and in dynamic fields. Thanks to a movie mode, dynamic fields can be analyzed with a time resolution down to 120 ms.

Safety information, technical information, and instruction for use for I’mRT QA are found in I’mRT QA User’s Guide.

1.2.3.7. BIS 710

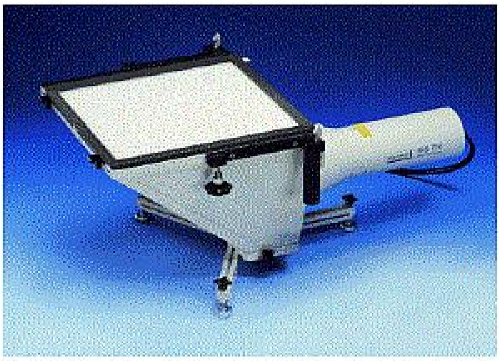

BIS 710 is used to measure intensity maps in 2D, both in static and in dynamic fields. The time resolution is 240 ms. BIS 710 is the predecessor to I'mRT QA.

Safety information, technical information, and instruction for use for BIS 710 are found in BIS 710 User’s Guide.

1.3. Product Documentation

1.3.1. OmniPro I'mRT System User's Guide

OmniPro I'mRT System User's Guide is available as a printed paper copy, and on-line as a .pdf file.

1.3.1.1. OmniPro I'mRT

OmniPro I'mRT System User's Guide contains health and safety information, installation, setup, and measurement instructions, and descriptions of the OmniPro I'mRT software functions for visualization, analysis, handling, and storing of measurement data.

1.3.1.2. I'mRT MatriXX and MatriXX Evolution

OmniPro I'mRT System User's Guide contains product information, health and safety information, instructions for installation, and instructions for setup and preparation of measurements for MatriXX.

1.3.1.3. Other Devices Supported by OmniPro I'mRT

OmniPro I'mRT System User's Guide contains installation, setup, and measurement instructions, related to OmniPro I'mRT software. For general information, refer to the product specific manuals (see *References*).

1.3.1.4. Intended User

This manual is intended for personnel with the following expertise:

Area	Expertise
Installation	Experts
Calibration	Experts
Configuration	Experts
Start-up, operation and shutdown	Experts<br>Trained personnel
Maintenance	Experts
Troubleshooting	Experts

Trained personnel (DIN EN 60204-1):

Any person who has been trained by experts in the tasks they need to perform, the risks associated with improper use, and the necessary safety equipment and safety precautions.

Experts (DIN EN 60204-1):

Any person who has the necessary specialist training and experience and knowledge of the relevant standards needed to appraise their work and to recognize the possible dangers.

1.3.1.5. Typographical conventions

Dialog captions and dialog text are indicated by bold font.
Examples: **Analysis Options**, **Field size**.

Menus and menu items are indicated by italic, bold font.
Examples: ***Equipment***, ***Radiation Devices***.
Chapter and section headings in this User's Guide are indicated by italic font.
Example: *Health and Safety Information*.

NOTICE

IMPORTANT NOTICE

Throughout this User's Guide, hazardous situations or operations are identified by DANGER, WARNING and CAUTION notices, where:

Sign	Meaning
⚠ DANGER	DANGER indicates a hazardous situation which, if not avoided, will result in death or serious injury of the operator or patient.
⚠ WARNING	WARNING indicates a hazardous situation which, if not avoided, could result in death or serious injury of the operator or patient.
⚠ CAUTION	CAUTION, used with a safety alert symbol, indicates a hazardous situation which, if not avoided, could result in minor or moderate injury of the operator or patient.
CAUTION	CAUTION, without the safety alert symbol, is used to address issues related to possible hardware damage.
NOTICE	IMPORTANT NOTICES are used to address operational issues not related to personal injury or hardware damages.

1.3.1.6. Images

NOTICE

IMPORTANT NOTICE

VALUES AND SELECTIONS DISPLAYED IN DIALOG IMAGES

Values and selections displayed in dialog images are examples. Values and selections must always be entered according to current measurement conditions.

1.3.2. On-line Help

Select ***Contents*** in the ***Help*** menu in OmniPro I'mRT to open the Online Help, or press <F1> to display context specific help.

NOTICE

IMPORTANT NOTICE

VALUES AND SELECTIONS DISPLAYED IN DIALOG IMAGES

Values and selections displayed in dialog images are examples. Values and selections must always be entered according to current measurement conditions.

2. Health and Safety Information

NOTICE

IMPORTANT NOTICE

ALL PERSONNEL MUST READ THIS CHAPTER

All personnel must read this chapter, and be fully aware of its contents before commencing installation work, and before operating or servicing OmniPro I'mRT System.

If the system is used in a way not specified by IBA Dosimetry, the protection provided by the equipment may be reduced.

The purpose of this chapter is to identify the hazards associated with the system, and especially with the OmniPro I'mRT software, I'mRT MatriXX, and MatriXX Evolution. Hazards connected to other hardware supported by OmniPro I'mRT are found in the respective operation manuals.

All personnel must read the Health and Safety chapters in the respective Operation Manuals, and be fully aware of their contents before operating, installing, or servicing the respective device.

⚠ WARNING

WARNING

GENERAL CONDITIONS

IBA Dosimetry accepts responsibility for the safety, reliability and performance of the OmniPro I'mRT System under the following conditions only:

- Assembly operations, extensions, or repairs are carried out by personnel authorized by IBA Dosimetry.
- The electrical installations of the relevant rooms comply with IEC recommendations.
- The OmniPro I'mRT System is used in accordance with this User's Guide, and with the respective Operation Manual(s).

2.1. OmniPro I'mRT Software

2.1.1. Responsibility

⚠ WARNING

WARNING

KNOWN ERRORS

Each user must be aware of the system restrictions and known errors specified in the ReadMe.txt file on the installation CD before using OmniPro I'mRT.

⚠ WARNING

WARNING

DATA EVALUATION RESPONSIBILITY

The person managing the OmniPro I'mRT system bears the full responsibility for critically evaluating every measurement result and/or manipulated measurements, before using data for verifying or modifying treatment plans, or using data for adjusting, checking, or servicing a radiation device.

⚠ WARNING

WARNING

GENERAL RESPONSIBILITY

OmniPro I'mRT is intended to be used by the physicist or engineer responsible for the quality control of radiation therapy equipment and of treatment delivery.

The user of OmniPro I'mRT must have experience in radiation physics and radiotherapy, and must be familiar with the terminology used in the area of dosimetry.

2.1.2. Data Sampling and Handling

⚠ WARNING

WARNING

FILM MEASUREMENT: SENSITIVITY MAY VARY FROM FILM TO FILM

The sensitivity of radiographic films is strongly dependent on parameters like type, age, batch, or storage conditions. Perform a film calibration when using films where one or more of the above parameters differ from the film used for the current film calibration.

⚠ WARNING

WARNING

FILM MEASUREMENT: OD VALUES OUTSIDE FILM SCANNER CALIBRATION RANGE

If a film is calibrated or measured outside the film scanner calibration range, the measurement values may not be correct. Never calibrate or measure a film outside the OD range for which the film scanner has been calibrated.

⚠ WARNING

WARNING

FILM MEASUREMENT: DOSE OUTSIDE THE FILM CALIBRATION RANGE

If a film is measured outside the film calibration range, the measurement values may not be correct. Never measure a film outside the dose range for which the film has been calibrated.

⚠ WARNING

WARNING

FILM MEASUREMENT: FILM BECOMES SATURATED

Depending on the choice of film, the saturation dose of the film may be lower than the maximum dose to be verified. Scale down the treatment plan or select an appropriate film.

⚠ WARNING

WARNING

FILM MEASUREMENT: MARKERS ON FILM

Markers for identification, orientation, or positioning may overlap or interfere with optical density, due to radiation. Mark your films outside the region where you want to determine the dose.

⚠ WARNING

WARNING

FILM MEASUREMENT: FILM AND FILM-ENVELOPE PRODUCE AN OFFSET

When inserting films in a body phantom, other film planes or slices are shifted, due to the thickness of the films and their envelopes.

When defining the offset information in the film setup, take into account the additional offset produced by the other films.

⚠ WARNING

WARNING

I'MRT QA/BIS MEASUREMENT: I'MRT QA/BIS CORRECTION IMAGE

The specification of the correction image file and the CCD area are mandatory. If you specify an incorrect or non-existing file and/or path, the measured image will not be corrected. If you specify an incorrect CCD area, the measured image will be incorrect. It will be very noisy and shifted, and may not appear fully on the screen.

⚠ WARNING

WARNING

I'MRT MATRIXX MEASUREMENT: I'MRT MATRIXX UNIFORMITY CORRECTION MATRIX

The application of the Uniformity Correction Matrix is mandatory. If the Uniformity Correction Matrix is not activated, the measured image will not be corrected.

⚠ WARNING

WARNING

SYMMETRY/FLATNESS: PARAMETERS MEASURED UNDER NON-REFERENCE CONDITIONS

Dosimetry protocols define the reference conditions for the determination of symmetry or flatness. Definition of water as medium, a certain depth, and a certain field is required, and also the type of detector to be used may be specified.

If measuring under different conditions (e.g. when using I'mRT QA/BIS in air), these parameters may differ from those measured under reference conditions.

⚠ WARNING

WARNING

ASCII EXPORT: LIMITED NUMBER OF DATA ROWS AND/OR COLUMNS

Some Windows applications support fewer rows and/or columns than the number of columns exported by OmniPro I'mRT (e.g. Microsoft EXCEL supports 256 × 65536).

If importing OmniPro I'mRT ASCII data to those applications, rows and/or columns exceeding the limit will not be imported.

If necessary, reduce the number of data by selecting a small region of interest.

⚠ WARNING

WARNING

TWAIN SCANNERS: 8-BIT GREYSCALE NOT SUFFICIENT

Using an 8 bit grey-scale scanner will give insufficient dose resolution, which will result in incorrect interpretation of data.

16 bit grey-scale is required.

⚠ WARNING

WARNING

DOSE EXPOSURE CHECKING: DOUBLE EXPOSURE

Double exposure will result in incorrect plan verification. When checking dose exposure for parts of a field or treatment, use a new film for each irradiation.

⚠ WARNING

WARNING

I'MRT MATRIXX: CALCULATION GRID

For correct comparison of treatment planning system (TPS) data and MatriXX data, the TPS grid resolution must be adapted to the MatriXX grid resolution. See *9.1 Convert Grid.*

2.2. MatriXX

2.2.1. Operator

MatriXX must only be used by people who

- are aware of, and understand, the limitations of the device in measurement of radiation output.
- have knowledge about safety procedures to be observed when working with radiation sources such as Cobalt-60 machines or linear accelerators.
- are aware of safety precautions required to avoid possible injury when using electrical/electronic equipment.

Before using I'mRT MatriXX or MatriXX Evolution for measurements, the operator must verify the general functionality, safety, and duly condition of the device and the dosimetry system. The device must not be used if any blemish is noticed, and the manufacturer should be notified.

2.2.2. Accessories

CAUTION

ACCESSORIES AND SPARE PARTS

No other accessories and spare parts than those provided or approved by the manufacturer must be used, otherwise operator safety, specified measuring accuracy, and interference free operation cannot be guaranteed. Violation of this prescription will result in loss of warranty.
IBA Dosimetry cannot be held liable for any damages resulting from the use of accessories or consumables that are not provided or approved by the manufacturer.

2.2.3. Environmental Requirements

MatriXX are sensitive measuring systems and must be stored and used in a clean, dry, preferably air-conditioned area, at room temperature. Protect them from mechanical and thermal stress, and unnecessary moisture. Avoid exposure to solvents and aggressive vapors.

2.2.3.1. Working and Storage Conditions

Temperature	+15 - +35°C
Pressure	500 - 1100 hPa
Relative humidity	30% - 70% without condensation

IMPORTANT NOTICE

THE DEVICE MUST NOT BE USED UNLESS IT IS COMPLETELY DRY

If moisture has developed as a result of temperature changes, I'mRT MatriXX or MatriXX Evolution must not be used unless it has been completely dried.

2.2.4. Storage

When the system is not in use, unplug the power supply from the mains connector.

Store MatriXX in a radiation protected area. Do not place the system near equipment generating a strong magnetic field.

WARNING

WARNING

DO NOT STORE THE MATRIXX ADJACENT TO OR STACKED WITH OTHER EQUIPMENT

MatriXX should not be stored adjacent to, or stacked with other equipment. If adjacent or stacked storing is necessary, the device should be observed to verify normal operation in the configuration in which it will be used.

CAUTION

CAUTION

DO NOT STORE THE DEVICE IN THE ACCELERATOR ROOM

Due to the sensitivity of the electronics against radiation, never store MatriXX in the accelerator room. This would considerably shorten their lifetime. Move the device into the accelerator room only if you intend to make measurements.

2.2.5. Handling

Handle MatriXX with care – avoid impact, excessive weight on the surface, and strong vibrations. Maximum load of build-up is 25 g/cm^2.

Never irradiate other parts than the marked sensor area.

Do not handle the devices with wet hands.

If a handling incident occurs, always perform a check before using the device again. If the device has been covered with water, un-power the device, and wait approx. 1 minute before touching it.

2.2.6. Electrical Installations

The electrical installations in the rooms where I'mRT MatriXX or MatriXX Evolution with connected equipment is used must comply with IEC recommendations. Always use a power cord with a grounding pin.

2.2.7. Rating Label

The power entry module of the device contains the rating label below:

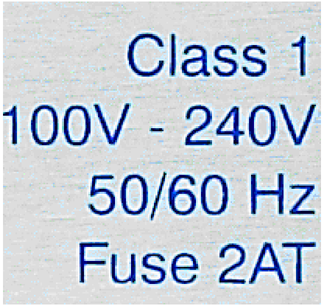

⚠ WARNING

WARNING

HAZARDOUS VOLTAGE

Pay attention to the voltage ratings on the rating label. This determines the safety hazards for the power supply. Do not open covers.

2.2.8. EMC Precautions

⚠ WARNING

WARNING

EMC PRECAUTIONS

MatriXX need special precautions regarding EMC and must be installed and put into service according to the EMC information provided in this manual.

⚠ WARNING

WARNING

RF COMMUNICATION EQUIPMENT

Portable and mobile RF communication equipment can affect the performance of MatriXX.

2.2.8.1. Guidance and Manufacturers Declaration – Electromagnetic Emissions

MatriXX are intended for use in the electromagnetic environment specified below.

The customer or the user of I'mRT MatriXX or MatriXX Evolution should assure that it is used in such an environment.

Emissions test	Compliance	Electromagnetic environment – guidance
RF emissions (CISPR 11)	Group 1	MatriXX use RF energy only for their internal function. Therefore, its RF emissions are very low, and are not likely to cause any interference in nearby electronic equipment.<br><br>MatriXX are suitable for use in all establishments other than domestic and those directly connected to the public low-voltage supply network that supply buildings used for domestic purposes.
RF emissions (CISPR 11)	Class A	
Harmonic emissions (IEC 61000-3-2)	Not applicable	
Voltage fluctuations/flicker emissions (IEC 61000-3-3)	Not applicable	

2.2.8.2. Guidance and manufacturer’s declaration – electromagnetic immunity

The I’mRT MatriXX is intended for use in the electromagnetic environment specified below.

The customer or the user of the I’mRT MatriXX should assure that it is used in such an environment.

Immunity test	IEC 60601 test level	Compliance level	Electromagnetic environment – guidance
Electrostatic discharge (ESD)<br>IEC 61000-4-2	±6 kV contact<br>±8 kV air	±6 kV<br>±8 kV	Floors should be wood, concrete, or ceramic tile. If floors are covered with synthetic material, the relative humidity should be at least 30%.
Electrical fast transient/burst<br>IEC 61000-4-4	±2 kV for power supply lines<br>±1 kV for input/output lines	±2 kV<br>±1 kV	Mains power quality should that of a typical commercial or hospital environment.
Surge IEC 61000-4-5	±1 kV differential mode<br>±2 kV common mode	±1 kV<br>±2 kV	Mains power quality should that of a typical commercial or hospital environment.
Voltage dips, short interruptions and voltage variations on power supply input lines IEC 61000-4-11	<5% U_T<br>(>95% dip in U_T) for 0,5 cycle<br>40% U_T<br>(60% dip in U_T) for 5 cycles<br>70% U_T<br>(30% dip in U_T) for 25 cycles<br><5% U_T<br>(>95% dip in U_T) for 5 sec	<5% U_T<br>40% U_T<br>70% U_T<br><5% U_T	Mains power quality should be that of a typical commercial or hospital environment.<br>If the user of I’mRT MatriXX or MatriXX Evolution requires continued operation during power mains interruptions, it is recommended that MatriXX be powered from an uninterruptible power supply or a battery.
NOTE U_T is the a.c. mains voltage prior to application of the test level.			
Power frequency (50/60 Hz) magnetic field IEC 61000-4-8	3 A/m	3 A/m	Power frequency magnetic fields should be at levels characteristic of a typical location in a typical commercial or hospital environment.

Immunity test	IEC 60601 test level	Compliance level	Electromagnetic environment – guidance
Conducted RF<br>IEC 61000-4-6<br><br>Radiated RF<br>IEC 61000-4-3	3 Vrms<br>150 kHz to<br>80 MHz<br><br>3 V/m<br>80 MHz to<br>2,5 GHz	3 V<br><br><br><br>3 V/m	Portable and mobile RF communications equipment should be used no closer to any part of I'mRT MatriXX or MatriXX Evolution, including cables, than the recommended separation distance calculated from the equation applicable to the frequency of the transmitter.<br>Recommended separation distance:<br>$d = \left[\frac{3.5}{V_1}\right]\sqrt{P}$<br>80 MHz to 800 MHz:<br>$d = \left[\frac{3.5}{E_1}\right]\sqrt{P}$<br>800 MHz to 2,5 GHz:<br>$d = \left[\frac{7}{E_1}\right]\sqrt{P}$<br>where P is the maximum output power rating of the transmitter in watts (W) according to the transmitter manufacturer and d is the recommended separation distance in meters (m).<br>Field strengths from fixed RF transmitters, as determined by an electromagnetic site survey a, should be less than the compliance level in each frequency range b.<br>Interference may occur in the vicinity of equipment marked with the following symbol:<br>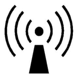

NOTE 1 At 80 MHz and 800 MHz, the higher frequency range applies.

NOTE 2 These guidelines may not apply in all situations. Electromagnetic propagation is affected by absorption and reflection from structures, objects and people.

Field strengths from fixed transmitters, such as base stations for radio (cellular or cordless) telephones and land mobile radios, amateur radio, AM and FM radio broadcast and TV broadcast cannot be predicted theoretically with accuracy. To assess the electromagnetic environment due to fixed RF transmitters, an electromagnetic site survey should be considered. If the measured field strength in the location in which MatriXX are used exceeds the applicable RF compliance level above, MatriXX should be observed to verify normal operation. If abnormal performance is observed, additional measures may be necessary, such as reorienting or relocating the MatriXX devices. In the frequency range 150 kHz to 80 MHz, field strengths should be less than 3 V/m.

2.2.8.3. Recommended separation distances between portable and mobile RF communications equipment and I’mRT MatriXX

I’mRT MatriXX is intended for use in an electromagnetic environment in which radiated RF disturbances are controlled. The customer or the user of I’mRT MatriXX can help prevent electromagnetic interference by maintaining a minimum distance between portable and mobile RF communications equipment (transmitters) and I’mRT MatriXX as recommended below, according to the maximum output power of the communications equipment.

**Rated maximum output power of transmitter (W)**	**Separation distance according to frequency of transmitter (m)**		
	150 kHz to 80 MHz $d = \left[\frac{3.5}{3V}\right]\sqrt{P}$	80 MHz to 800 MHz $d = \left[\frac{3.5}{3V/m}\right]\sqrt{P}$	800 MHz to 2,5 GHz $d = \left[\frac{7}{3V/m}\right]\sqrt{P}$
0.01	0.12	0.12	0.25
0.1	0.37	0.37	0.74
1	1.2	1.2	2.4
10	3.7	3.7	7.4
100	12	12	24

For transmitters rated at a maximum output power not listed above, the recommended separation distance d in meters (m) can be estimated using the equation applicable to the frequency of the transmitter, where P is the maximum output power rating of the transmitter in watts (W) according to the transmitter manufacturer.

NOTE 1 At 80 MHz and 800 MHz, the separation distance for the higher frequency range applies.

NOTE 2 These guidelines may not apply in all situations. Electromagnetic propagation is affected by absorption and reflection from structures, objects and people.

2.2.9. Naming in the manual

If further chapters are related to both, the I’mRT MatriXX and MatriXX Evolution only “MatriXX” will be appear in the context.

2.3. Regulatory Requirements

2.3.1. OmniPro I'mRT

OmniPro I'mRT software fulfils the requirements of the Medical Device Directive 93/42/EEC. It is a medical device class IIb according to annex IX, classification rule 10.

2.3.2. I'mRT MatriXX and MatriXX Evolution

I'mRT MatriXX and MatriXX Evolution fulfill the requirements of the Medical Device Directive 93/42/EEC. They are medical devices class I according to annex IX, classification rule 12.

Protection Class IP 21.

2.3.3. Other Devices Supported by OmniPro I'mRT Software

See the respective Operation Manuals of the devices supported by OmniPro I'mRT.

2.3.4. Quality Management System

The quality management system at IBA Dosimetry GmbH and IBA Dosimetry AB is certified according to EN ISO 13485:2003.

2.3.5. CE Marking

CE 0413 CE 0124

3. General Workflow for QA with MatriXX

3.1. Basic Preparations

3.1.1. Scanning of MatriXX in CT scanner

For calculating the dose for verification plans in the TPS a CT scan of the MatriXX is necessary. The verification plan will be calculated on the MatriXX CTs. The TPS plane where the effective point of measurement of the chamber row is located will be exported. A calculated TPS dose plane and a 2D measurement at the same position are available for verification in the OmniPro I'mRT software.

During the CT scan the position of MatriXX at the linear accelerator should be exactly like during the subsequent measurement of the verification plans. In order to be able to measure the scattering, MatriXX is positioned either in the MULTICube or on a 5 cm thick layer of water equivalent material (RW3-plates). Also, as build-up material a 5 cm thick layer of RW3-plates is positioned on the MatriXX measurement area. In this way the scattering resembles that of the human body.

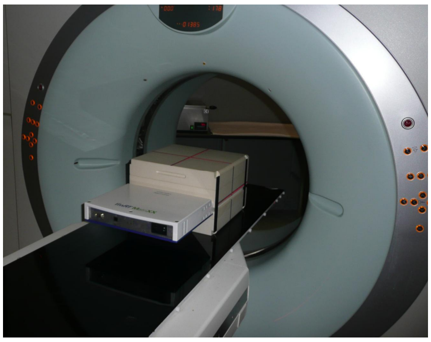

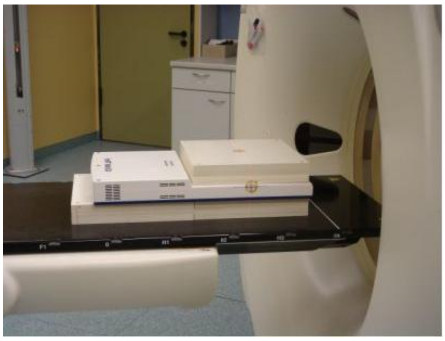

If the gantry holder is used the same build-up and backscatter should be mounted for the verification measurement as used in the CT.

To use the CT data in the TPS system, the origin has to be precisely defined. MatriXX is positioned in such a way in the CT scanner that the central point of the measurement level is positioned exactly at the CT central axis. Thus point zero of the measurement system always coincides with point zero of the CT data set (DICOM origin). In the plan system the iso-centre can be positioned precisely in the DICOM origin.

NOTICE

IMPORTANT NOTICE

DIFFERENT CT SCANS FOR DIFFERENT MATRIXX'S

For every MatriXX a CT is necessary. Otherwise deviations in dose calculation and measurements are possible. Ensure that the correct CT scan is used.

3.1.2. Calibration of the MatriXX Measurement System

The calibration of the MatriXX should be carried out directly after the calibration of the linac. MatriXX has built-in pressure and temperature sensors. Therefore the input of pressure and temperature is not necessary during the calibration, nor during the subsequent measurements.

The measurement setup follows directly from the verification measurements and therefore directly from the setup during the CT scan.

The precise thickness of the water-equivalent layer over the MatriXX measurement level is known and therefore also the exact number of monitor units required for a certain dose (for more details please see chapter *7.4.4.1 Creating a kUser Factor*).

3.2. Realization of the Measurement

The MatriXX setup is exactly like the setup used for the CT scan.

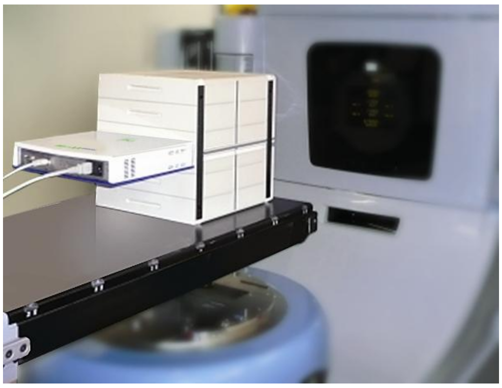

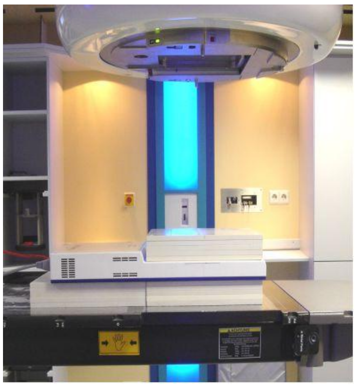

For the exact orientation of the measurement system, the gantry and collimator angles of the linear accelerator must be set at precisely 0°. The vertical orientation of MatriXX is carried out with assistance from the laser and the sideways positioned markers on MatriXX that indicate the exact position of the measurement level (for more details please see chapter *7 Setup of MatriXX*).

After connecting the MatriXX with the computer, setting the measuring parameters in the software and measuring the background the first measurement can be started. Either all fields of the treatment plan can be irradiated in a queue as one measurement or one field after each other in single measurements to verify each single field. The raw data should be collected beforehand as copy in a separate order.

3.3. Analysis

The most common method to analyze the pre-treatment verification is using the Gamma Index Method. The Gamma Index takes the dose deviation (Delta Dose) and the dose distribution (Distance-To-Agreement) into account. For more information please see chapter *14.1.2 2D Data*. A resulting matrix is formed that gives a value of the Gamma Index for every tested measurement point. If this value is smaller than or equal to 1, the criteria values are not exceeded. However, if this value is higher than 1 the measurement result lies outside the tolerance range. Since the pixels with Gamma Index > 1 are colored red in OmniPro I'mRT, it is easy to determine if there are areas outside the tolerance range, and how large these areas are. Furthermore, the area outside the tolerance range can be allocated to the patient's anatomy.

4. Hardware description

4.1. I'mRT MatriXX and MatriXX Evolution

4.1.1. Intended Use

4.1.1.1. I'mRT MatriXX

I'mRT MatriXX is intended for pre-treatment verification of 2D dose distribution, and for routine QA of high energy photon and electron beams radiation, produced by treatment units: Linacs and ^{60}Co.

Data obtained with the MatriXX is intended to be used for QA procedures only.

Data is not intended to be transferred to a treatment planning system.

4.1.1.2. MatriXX Evolution

MatriXX Evolution is intended for 2D digital verification in rotational therapy techniques.

Data obtained with MatriXX Evolution is intended to be used for complete plan verification and QA of ImRT, IGRT, and rotational treatments.

Data is not intended to be transferred to a treatment planning system.

4.1.2. Connection of I'mRT MatriXX and MatriXX Evolution

4.1.2.1. Ethernet Connection

To connect MatriXX, a standard Ethernet interface must be available on the computer. MatriXX can also be connected via a network.

See chapters *5.1 I'mRT MatriXX and MatriXX Evolution – Installation*, *7 I'mRT MatriXX – Setting up for Measurements*, and *7.2 MatriXX Evolution – Setting up for Measurements* for further description of the setup.

4.1.3. Technical Specifications

4.1.3.1. General

Item	Characteristic
Warm-up time	15 – 60 min (see *7.3 Pre-irradiation and warm-up*)
Cooling	Forced air cooling via two fans
Uniformity correction	Via an individual factory calibration matrix, determined with ^{60}Co radiation, stored in the device

4.1.3.2. Detector

Item	Characteristics
Sensor type	Vented parallel ion chamber
Number of sensors	1020, arranged in a 32 × 32 grid
Active measurement area	24,4 cm × 24,4 cm
Chamber diameter	4.5 mm
Chamber height	5 mm
Chamber volume	0.08 ccm
Distance between chambers	7.619 mm
Nominal sensitivity	2.4 nC/Gy
Maximum dose rate	20 Gy/min
Minimum dose rate	0.02 Gy/min
Bias voltage	500 ±30 V
Effective point of measurement	3 mm below of surface, indicated by an engraved marker on the side of the housing
Absorber material on top	ABS (density 1.06 g/cm^3)
Water equivalent depth	3.2 mm

4.1.3.3. Electronics

Item	Characteristics
Minimum sampling period	20 ms
Non-linearity	±0.5%
Data link	10/100 baseT (Ethernet)
Communication protocol	TCP/IP
Mains voltage range	100 – 240 VAC ±10%
Max. power consumption	15 W
Electrical safety standard	IEC/EN 60601-1
EMC standard	IEC/EN 60601-2:2001

4.1.3.4. Mechanics

Item	Characteristics
Outer dimensions	560 × 60 × 320 mm (l × h × w)
Weight	10.5 kg

4.1.3.5. Operational Conditions

Condition	Range
Ambient temperature	+15° - +35 °C
Relative humidity range	500 – 110 hPa
Atmospheric pressure	30% - 70% without condensation

4.1.3.6. Connectors and LEDs

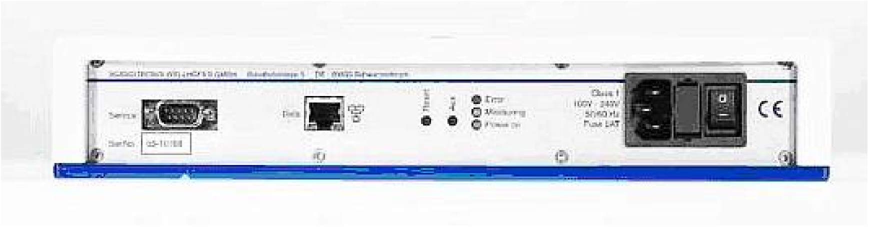

Connector/LED	Label	Function
Ethernet	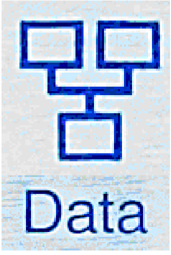	Connection to a PC or a network.
Reset button	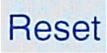	For reset of the device.
Auxiliary button	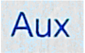	For reset to the Ethernet settings.
Green LED	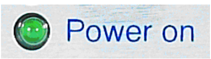	Illuminated if the MatriXX is properly connected to mains power, and switched on.

Connector/LED	Label	Function
Yellow LED	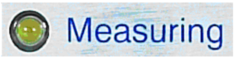	Illuminated if a measurement is running.
Red LED	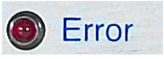	Error indication.
Power entry module	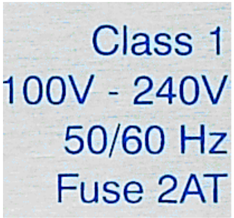	Connection to mains power.
Service connector, I'mRT MatriXX	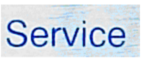	To be used by service personnel only.
Service connector, MatriXX Evolution	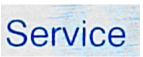	Connector for Gantry Angle Sensor.<br>To be used for other purposes by service personnel only.

4.1.3.7. Power Supply

The power entry module consists of:

- an appliance inlet according to IEC/EN 60320-1/C14 protection class 1, cold condition
- a two pole fuse holder, one for the active and one for the neutral line, according to IEC/EN 60137-6 for fuse links 5 × 20 mm

4.1.3.8. Data Cable

For communication with the PC a good quality shielded Cat5e patch cable shall be used, in order to ensure the emission and immunity levels of the EMC test. The cable length shall be ≤ 40 m.

Two data cables are delivered with MatriXX: One shielded cross-over Ethernet cable for direct connection to the PC, 2 m, and one straight cable for LAN connection, 30 m. A junction box is also included. If necessary, the cross-over cable can be extended with the straight 30 m cable via the junction box.

4.2. I'mRT MatriXX - Accessories

For mounting of the MatriXX on the gantry, adapters for Siemens, Varian, and Elekta gantries are available.

XY tables on a support frame, build up plates, and backscatter plates with a holder are also available.

4.2.1. XY Tables

The XY table is used for adjusting the MatriXX position to the crosshairs in the light field of the accelerator. It is mounted on the support frame for the MatriXX and the backscatter holder.

XY tables are available in a standard and an advanced version.

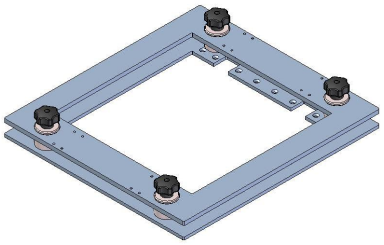	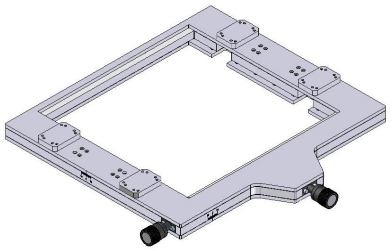
XY table, standard version	*XY table, advanced version*

4.2.1.1. Specifications

4.2.1.1.1. Support Frame

Item	Characteristics
Material	Aluminum, Delrin (POM), and steel
Weight	2.5 kg

4.2.1.1.2. XY Table, Standard Version

Item	Characteristics
Material	Aluminum, Delrin (POM), and steel
Weight	3.5 kg

4.2.1.1.3. XY Table, Advanced Version

Item	Characteristics
Material	Aluminum, brass, and steel
Weight	4.9 kg

4.2.2. Build-up Plates

Plates for build up in steps of 1, 2, 5, and 5 mm are available from IBA Dosimetry. The material is water-equivalent polystyrene, RW3.

Plates made of RW3 and PMMA from other manufacturers can be used, provided that the size fits into the support frame.

4.2.2.1. Specifications

Item	Characteristics
Material	RW3 (water-equivalent polystyrene)
Size	300 × 300 mm ± 0.1 mm
Thickness	10 mm, 4 plates<br>5 mm, 1 plate<br>2 mm, 2 plates<br>1 mm, 1 plate<br>Total: 5 cm
Weight	8 plates: 5 kg

4.2.3. Backscatter Holder and Plates

Backscatter plates for build up in steps of 1, 2, 5, and 10 mm are available from IBA Dosimetry. The material is water-equivalent polystyrene, RW3.

The plates must be mounted in a backscatter holder, and attached to a support frame (see *7.1.1.3 Mounting Equipment into the Support Frame*).

4.2.3.1. Specifications

4.2.3.1.1. Backscatter Holder

Item	Characteristics
Material	10 mm water-equivalent polystyrene (RW3), PVC, and steel
Weight	1.9 kg

4.2.3.1.2. Backscatter Plates

Item	Characteristics
Material	Water-equivalent polystyrene (RW3)
Thickness	10 mm, 8 plates<br>5 mm, 1 plate<br>2 mm, 2 plates<br>1 mm, 1 plate<br>Total: 9 cm
Size	300 × 300 mm ± 0,1 mm
Weight	12 plates: 8.6 kg

4.2.4. Gantry Adapters

The gantry adapters are available in two versions, one for isocenter mounting for SDD 100 cm, and one for SDD 76.2 cm.

4.2.4.1. Varian Adapters

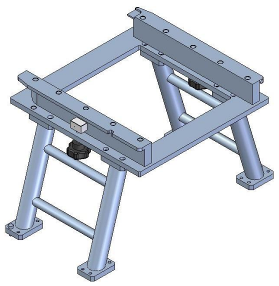

Varian adapter for SDD 100 cm

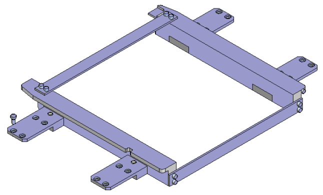

Varian adapter for SDD 76.2 cm

Total weight of the support frame with the MatriXX with 50 mm build up, backscatter holder with 10 cm backscatter plates, and the gantry adapter:

- With the adapter for SDD 100 cm 37.9 kg
- With the adapter for SDD 76.2 cm 35.4 kg

4.2.4.2. Siemens Adapters

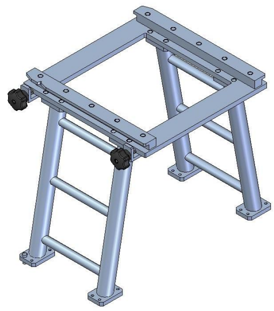	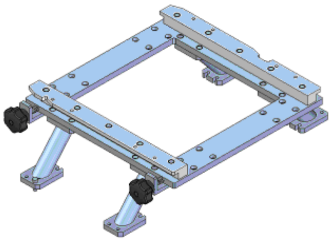
Siemens adapter for SDD 100 cm	*Siemens adapter for SDD 76.2 cm*

Total weight of the support frame with the MatriXX with 50 mm build up, backscatter holder with 10 cm back scatter plates, and the gantry adapter:

- With the adapter for SDD 100 cm 38 kg
- With the adapter for SDD 76.2 cm 37 kg

4.2.4.3. Elekta Adapters

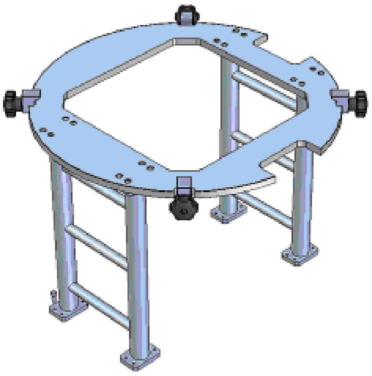	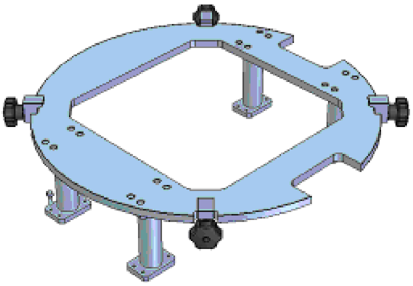
Elekta adapter for SDD 100 cm	*Elekta adapter for SDD 76.2 cm*

Total weight of the support frame with the MatriXX with 5 cm build up, backscatter holder with 10 cm backscatter plates, and the gantry adapter:

- With the adapter for SDD 100 cm 39.6 kg
- With the adapter for SDD 76.2 cm 38.3 kg

4.3. I'mRT QA / BIS and BIS 710

4.3.1.1. Frame Grabber Installation

The frame grabber board is a PCI board, which must be installed in the computer that will be used for the I'mRT QA measurements. Refer to the I'mRT QA/BIS2G or BIS 710 manuals for further descriptions of the setup.

4.4. Vidar Scanner Installation

Vidar scanners can be connected with an SCSI adapter, or (scanners with serial number 310000 or higher) a USB interface.

NOTICE

IMPORTANT NOTICE

WINDOWS VISTA

Windows Vista supports the Vidar DosimetryPRO Advantage (Red) scanner, but no other Vidar scanners.

4.4.1.1. SCSI

4.4.1.1.1. Installation of the Adapter Board

1. Install the SCSI adapter board, according to the adapter manual. Recommended adapters are Adaptec AHA-2940AU® and Adaptec AHA-2930CU®.
2. Switch off the computer and scanner.
3. Connect the SCSI cable between the PC and the scanner.
4. Set the ID on the scanner to 5.
5. Connect an external SCSI terminator, or set the switch according to the scanner manual.
6. Check the mains voltage setting.
7. Connect the power cord.

4.4.1.1.2. Booting

NOTICE

IMPORTANT NOTICE

SCANNER ID

Some computers do not start if the scanner SCSI ID value is less than 4. Switch off the computer and the scanner. Change the scanner ID.

Switch the scanner on, and then the PC.

NOTICE

IMPORTANT NOTICE

WAIT BETWEEN BOOTINGS

Allow 5 minutes for the scanner boot procedure to be completed, before booting the PC.

During the booting, check that the following messages appear:

- Adaptec AHA......
- Press <<<Ctrl+A.....>>>
- VIDAR VXR…

This means that the PC has recognized the scanner.

4.4.1.1.3. Installation of SCSI Adapter and Scanner Drivers

The plug-and-play function recognizes the SCSI adapter, and starts the Add New Hardware wizard.

Select Search for the best driver for your device.

Find the Adaptec SCSI adapter in the list of installed hardware.

Restart the computer.

Select ***Settings/ControlPanel/System/Hardware/Device manager - SCSI adapters*** from the Windows ***Start*** menu. Check that the Adaptec adapter is present. If there is a yellow exclamation mark on the icon, there is a problem with the drivers. Select **Properties** and change driver.

Insert the Vidar Drivers and Toolkit Installation CD, and install the STI drivers and the Vidar Toolkit. (The CD is supplied with the scanner.)

The STI driver and Vidar Toolkit for Windows® 2000 and XP are also available at the Vidar Systems' website:

www.filmdigitizer.com/html/drivers.html.

The plug-and-play function recognizes the VXR scanner, and tries to install a driver for that device. It is found on the Vidar installation CD. Step through the pages in the ***Found New Hardware*** wizard, selecting the default alternatives. Finally, select ***Finish***.

4.4.1.2. USB

The computer must have a USB 2.0 port. A USB 2.0 peripheral connected to a 1.1 port will not recognize the Vidar scanner.

A USB 2.0 adapter can be installed. The Adaptec™Model USB2Connect, part number AUA-2000 is recommended.

The scanner must be connected to the computer with a USB 2.0 cable.

4.4.1.3. Installation

4.4.1.3.1. Installation Procedure for all Vidar Scanners Except Vidar DosimetryPRO Advantage (Red) Scanner

Vidar drivers and Toolkit Installation CD will be needed.

Ensure that the scanner is turned off, and is NOT connected to the PC. Wait until you are prompted to connect the digitizer.

Turn on the PC.

Insert the Vidar Drivers and Toolkit Installation CD. The installer should launch automatically.

If the installer does not install automatically, double-click the appropriate CD drive icon under My Computer in Windows Explorer.

In the first screen, click Install Software.

4.4.1.3.2. Installation Procedure for Vidar DosimetryPRO Advantage (Red) Scanner

1. Install the driver and the toolkit.
2. Vista: Copy the file vscsi32.dll from C:/user_name/Documents/Vidar/Toolkit to C:/Program Files/IBADosimetry/OmniPro I'mRT 1.7
3. XP: Copy the file vscsi32.dll from C:/Documents and Settings/user_name/My Documents/Vidar/Toolkit to C:/Program Files/IBADosimetry/OmniPro I'mRT 1.7

4.4.1.3.3. Operation System, Driver, and Toolkit Compatibility

Operation system	Driver	Toolkit version	Run
XP	XP Driver	5.0.8.0	OK
Vista	Vista Driver 4.0.6.4	5.0.8.0	OK
XP	Vista Driver 4.0.6.4		OK
Vista	XP Driver		Not running

4.5. Kodak LS (Lumiscan) Scanner Installation

NOTICE

IMPORTANT NOTICE

WINDOWS VISTA AND XP

Windows Vista and XP do not support Kodak LS scanners.

4.5.1.1. Free Resources

The default resources used by the control board are:

- IRQ 5
- I/O 0x100 – 0x11F
- Memory 0xD0000 – 0xD7FFF

If the above resources are not free, find free resources on the computer, and change the switches according to the new resources. See the scanner manual for switch setting and possible choices.

NOTICE

IMPORTANT NOTICE

ISA SLOT

There must be a free ISA slot on the PC.

4.5.1.2. Installation of LumiScan Driver

NOTICE

IMPORTANT NOTICE

WINDOWS VISTA AND XP

There are no drivers for the LumiScan scanner available that work under Windows XP or Vista.

4.5.1.2.1. Windows 2000

Please check the Readme file of the LumiScan DeskTop (LSDT) Software version 2.50 or later.

4.5.1.2.2. Connecting the Scanner

Turn off the PC. Connect the scanner control cable. Turn on the PC.

5. Installation

5.1. Installation of OmniPro I'mRT Software

5.1.1. Computer Requirements

OmniPro I'mRT software work under Windows® XP Professional Service Pack 2, Windows 2000 Service Pack 4, and Windows Vista Ultimate Edition, Service Pack 1.

.NET Framework 2.0 must be installed (Internet Explorer 5.01 or higher is required for installation).

The validation of the software is done with the US versions of the operating systems. Troubleshooting or support in case of problems related to local language operating systems is therefore very limited.

NOTICE

IMPORTANT NOTICE

SOFTWARE CONTROLLING MATRIXX

The software controlling the MatriXX is included as a part of Windows application softwares developed by IBA Dosimetry, and can be directly accessed from the application's main window.

NOTICE

IMPORTANT NOTICE

UNDER WINDOWS VISTA, THE FOLLOWING EQUIPMENT IS NOT SUPPORTED:

- Vidar scanners older than DosimetryPRO Advantage (Red)
- Kodak LS scanners
- I'mRT QA
- BIS 710

NOTICE

IMPORTANT NOTICE

KODAK LS SCANNERS

Kodak LS scanners require Windows 2000; they do not work under Windows XP or Vista.

To use OmniPro I'mRT, with all its options, we recommend that your computer fulfills the following requirements:

- PC with a 2 GHz Pentium processor
- Microsoft Windows 2000 SP4, XP Professional SP2, or Vista Ultimate SP1 (32-bit)
- 2 GB of RAM memory (recommended for real-time intensity mode)
- 160 GB hard disk; 40 GB recommended for data archiving
- Ethernet RJ-45 connection (for MatriXX)
- Graphics card supporting 32 bit colors and 1024 × 768 pixels
- CD-drive

- SCSI-card (for Vidar scanners)
- Interface for film scanners (SCSI, PCI, or USB v. 2.0)
- PCI slot for Frame Grabber (for I'mRT QA/BIS)
- Microsoft Data Access Components (MDAC 2.5)

The latest type of computer is always recommended, especially if you are going to use large data sets or work with film scanners.

5.1.2. OmniPro I'mRT Installation Utility

The OmniPro I'mRT CD-ROM contains a setup utility that will install the necessary files and create a program group and icons.

NOTICE

IMPORTANT NOTICE

START BY INSTALLING THE SCANNER

If you have a film scanner, the scanner must be installed prior to the installation of I'mRT software, otherwise the vscsi32.dll file might be overwritten.

NOTICE

IMPORTANT NOTICE

LOG IN AS ADMINISTRATOR

When installing, log in as Administrator. Otherwise the installation will fail.

Follow these steps to install the software:

Insert the CD-ROM in the CD-drive.

After a few seconds the installation program starts. If not, go to the Windows Start menu. Select ***Settings-Control Panel-Add/Remove Programs***. Click ***Add new program***. Click ***CD*** or ***Disk***. Follow the instructions on the screen.

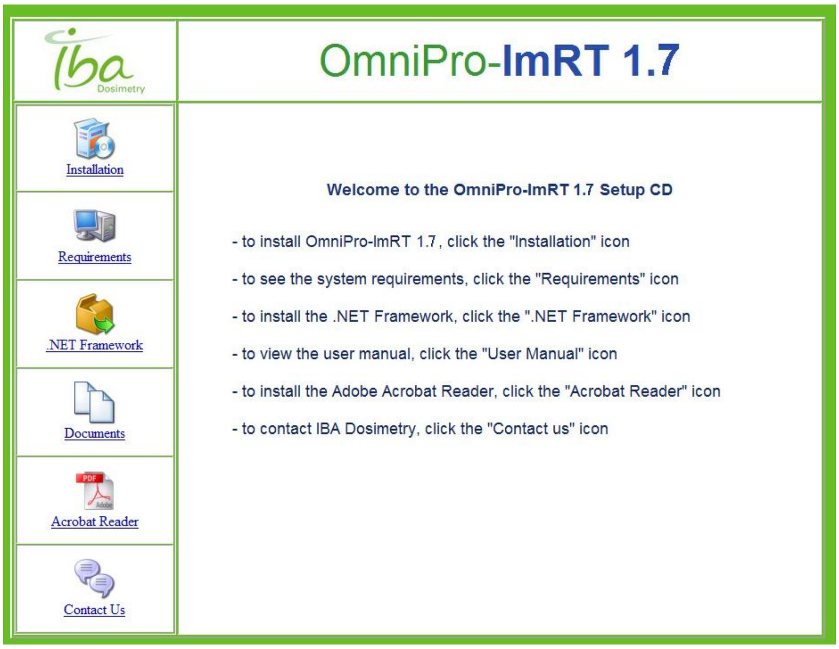

Select what you want to do:

- Installation: Allows you to start the installation or the un-installation of OmniPro I'mRT, and to read the OmniPro I'mRT Read me file.
- Requirements: Displays the requirements for the computer you want to use together with OmniPro I'mRT.
- Documents: Allows you to read a pdf file of the User's Guide. (Acrobat Reader is required). Please note that this pdf file contains the same information as the printed User's Guide.
- Acrobat Reader: Allows you to install the Acrobat Reader.

5.1.2.1. OmniPro I'mRT Installation Procedure

CAUTION

CAUTION

INSTALLED FILES

During the installation of this software some important files are placed in the same directory as the application. Since the application needs these files to be able to run properly, please do not move the OmniPro I'mRT application from the original directory.

Select **Install OmniPro I'mRT**. The Installation wizard starts. Follow the instructions on the screen.

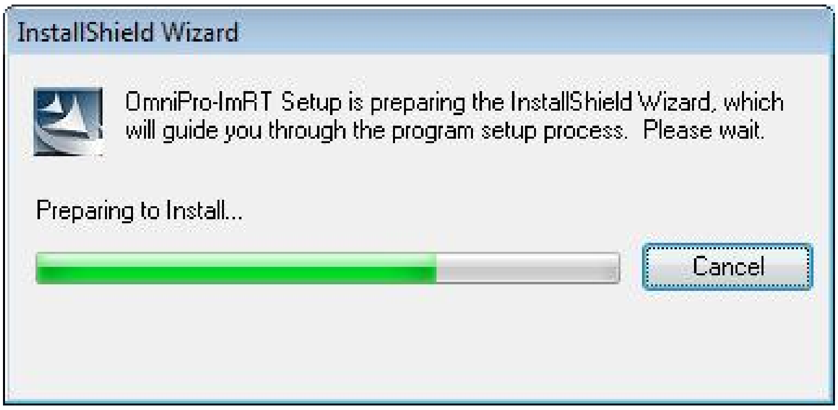

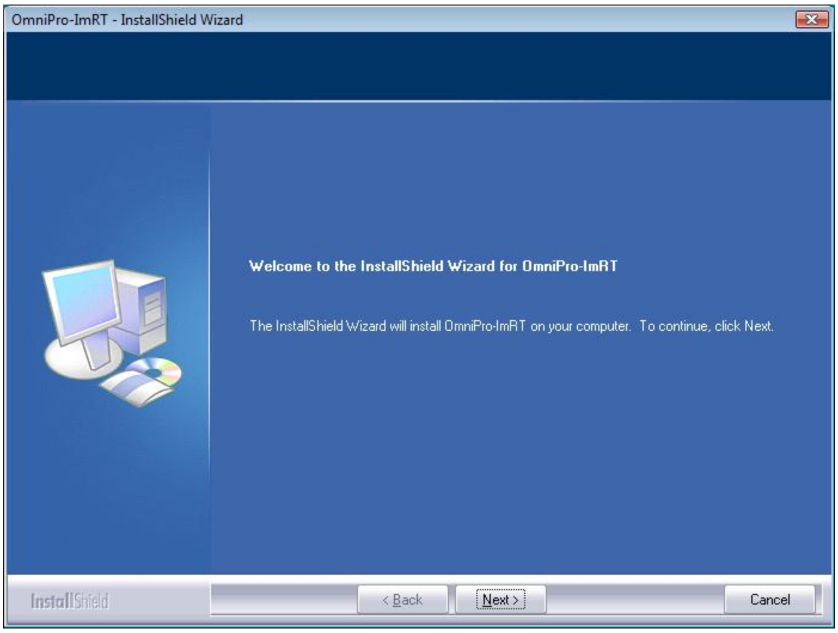

NOTICE

IMPORTANT NOTICE

WINDOWS VISTA

A message "An unidentified program wants access to your computer" might appear. The program is setup.exe. Select **Allow to continue**.

Follow the instructions on the screen:

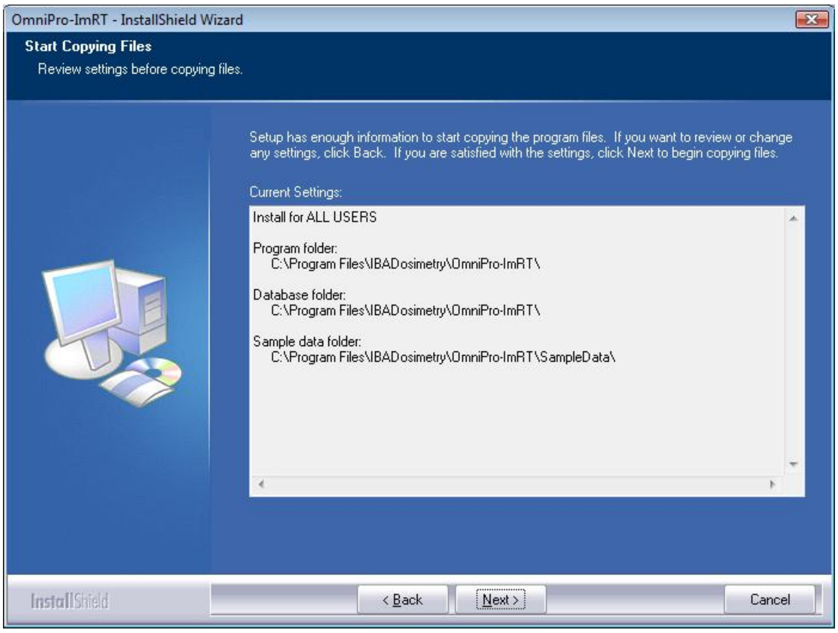

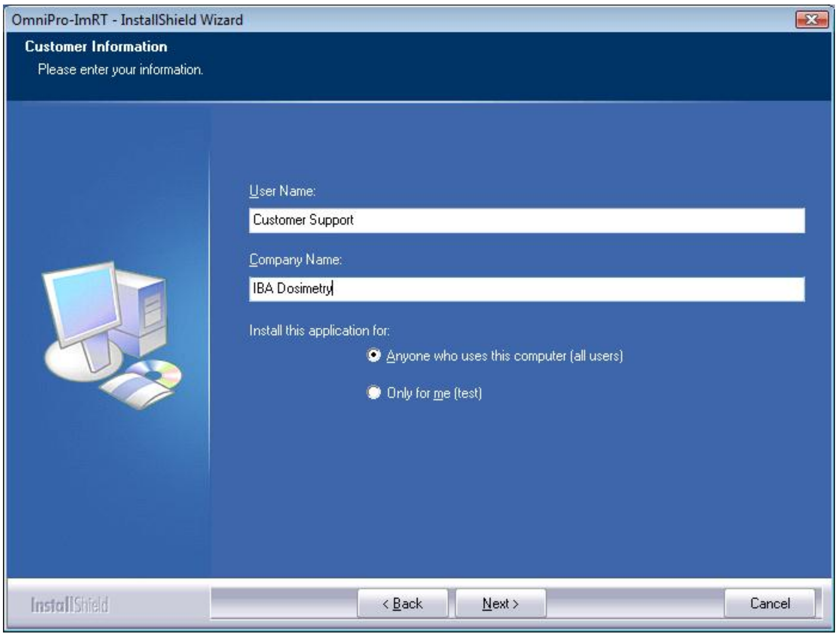

During installation of the software, an empty data base is created in a user definable folder, and a connection to this data source is established.

The database can be installed either locally on a computer, or in a network.

NOTICE

IMPORTANT NOTICE

READ AND WRITE ACCESS

All users must have full read and write access to the OmniPro I'mRT folder where the equipment database is located, to be able to run OmniPro I'mRT.

If OmniPro I'mRT shall be installed on more than one computer, it is recommended to make the database available for all installations:

Choose a directory that is shared via network.

Map this directory on each computer to a drive letter.

NOTICE

IMPORTANT NOTICE

DRIVE LETTER

The drive letter must be the same for all users on an individual computer.

NOTICE

IMPORTANT NOTICE

DESTINATION DIRECTORY

If an installation in a network is planned, the destination directory must be mapped to a driver letter. This driver letter must be the same for all users.

Select the shared drive letter as database folder. Follow the instructions on the screen.

Repeat the installation and select the shared driver letter on all computers.

NOTICE

IMPORTANT NOTICE

SHARED DATABASE LOCATION

When installing OmniPro I'mRT on several computers, select the same shared database location as for the first installation. The installation program will detect that the database already exists, and will connect to it (instead of copying an empty database to the folder).

NOTICE

IMPORTANT NOTICE

NO ACCESS TO THE NETWORK

Sometimes the administrator on the computer does not have access to the network. If that is the case, consult the chapter *Troubleshooting*.

NOTICE

IMPORTANT NOTICE

SOFTWARE VERSION EARLIER THAN 1.4

If an earlier version than version 1.4 is used (e.g. if not all users in a network have yet been upgraded), the equipment database created in version 1.7 will not be accessible.

5.1.3. Test of the Installation

A test is available to ensure that the OmniPro I'mRT software has been installed correctly. Use the example file, delivered with the software, to check the installation:

NOTICE

IMPORTANT NOTICE

WINDOWS VISTA

To be able to run OmniPro I'mRT correctly, and to create the computer ID, the program must be started with **Run as Administrator** the first time, even if you are logged in as administrator.

Right click the OmniPro I'mRT icon, and select **Run as Administrator**.

Select **Accept** when the message "An unidentified program wants access to your computer" appears.

Double-click the OmniPro I'mRT icon on the desktop, or go to the Windows Start menu. Select ***Program-IBA Dosimetry-OmniPro-IMRT***. The program will start.

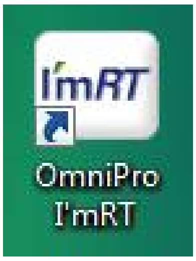

Select the menu command ***File:Open Workspace***. A standard **Open** dialog opens.

Browse for an *.opw file. (It should be stored in the DATA folder). Open the sample file.

The **Workspace** opens. Depending on the type of data loaded, it will be displayed in either **Plan Verification 2D** or **3D** mode.

5.1.4. Registration of the Software

After installation the software works for a limited time (30 days). Thereafter it will run only if it has been registered.

NOTICE

IMPORTANT NOTICE

LOG IN AS ADMINISTRATOR

You must be logged in as Administrator when entering the license number and the license key.

When you start the program after installation, the **About OmniPro I'mRT** dialog will open.

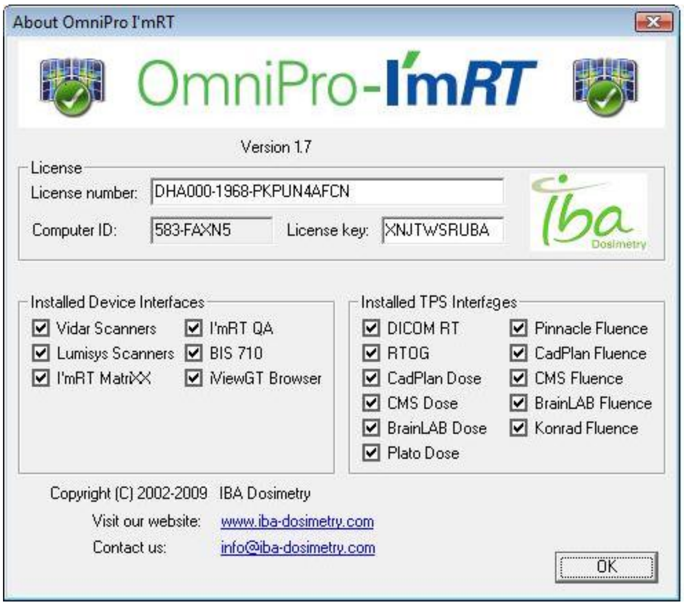

Register the software at IBA Dosimetry in the following way:

8. Note the License Number and the Computer ID for each installation.
9. Contact IBA Dosimetry for registration via the Radiotherapy/Support section of the homepage www.iba-dosimetry.com.

Registration may also be made by e-mail or fax.

By fax, +46 (0)18 12 75 52: fill in and send the included registration form.

By email, register@iba-group.com.

The following information must be included:

- Name and address (do not forget email address).
- Product serial number
- Computer ID

For more information check the Registration Form sent to you together with the installation CD.

After a few days a license key will be sent to you.

Open the **About** box (found under ***Help***), and enter the License Key.

5.1.5. Removing OmniPro I'mRT

NOTICE

IMPORTANT NOTICE

LOG IN AS ADMINISTRATOR

You must be logged in as Administrator to remove the program.

To remove the OmniPro I'mRT software, select ***Settings-Control Panel-Add/Remove Programs*** from the Windows ***Start*** menu.

From the list of installed programs, select OmniPro I'mRT and click **Add/Remove**. The Installation wizard starts. Follow the given instructions.

Alternative procedure:

Insert the OmniPro I'mRT CD-ROM.

Select **Install OmniPro I'mRT**.

The installation program presents three alternatives. Select **Remove**.

All OmniPro I'mRT files, except for the calibration files, setup and equipment information, and measured data, will be removed.

5.1.6. Reinstallation of OmniPro I'mRT

NOTICE

IMPORTANT NOTICE

LOG IN AS ADMINISTRATOR

You must be logged in as Administrator to reinstall the program.

It may be necessary to perform a reinstallation of the software, e.g. when installing a new option.

Insert the OmniPro I'mRT CD-ROM.

Select **Install OmniPro I'mRT**. The installation program will present three alternatives. Select **Modify**. The installation starts.

During the installation, enter the new license number from the License Document.

NOTICE

IMPORTANT NOTICE

DO NOT SELECT REPAIR

Do not select **Repair** to install new options, since OmniPro I'mRT will be reinstalled using the same settings as during the previous setup.

NOTICE

IMPORTANT NOTICE

MOVING THE SOFTWARE TO ANOTHER COMPUTER

If it becomes necessary to move the software to another computer, e.g. after a computer crash, a new registration is necessary. Follow the instructions for a normal registration.

5.2. Computer settings for MatriXX

MatriXX can be connected directly to a PC, or to a local network (LAN), via an Ethernet cable.

Several devices can be connected to a network.

Administrator status is required to perform connections.

NOTICE

IMPORTANT NOTICE

MATRIXX

In the instructions for connection to a PC or a local network (*9.2 Direct Connection to a PC* and *9.3 Connection to a Local Network*) screenshots for I'mRT MatriXX are displayed. The procedure for MatriXX ^{Evolution} is exactly the same as for I'mRT MatriXX.

5.2.1. TCP/IP and IP Addresses

5.2.1.1. TCP

The Transmission Control Protocol is virtual circuit protocol that to create connections between hosts on a network. The protocol guarantees reliable and in-order delivery of data from sender to receiver.

5.2.1.2. IP Address

An IP address (Internet Protocol address) is a unique address that network connected devices use to identify and communicate with each other.

The IP address (IPv4) consists of four numbers (0..255) separated by period, e.g. 192.168.23.34.

5.2.1.3. Subnet Mask

Subnet mask is a mask used to determine what subnet an IP address belongs to. An IP address has two components, the network address and the host address.

Normally the subnet mask is 255.255.0.0 (Class B) or 255.255.255.0 (Class C) for company networks.

As an example, consider the IP address 150.215.017.009:

The subnet mask is 255.255.0.0. The two first numbers in the IP address (150.215), which corresponds to 255.255 in the subnet mask, is the network address. The last two numbers (017.009), which corresponds to 0.0 in the subnet mask, is the host address on the network.

IP Address	150.215.017.009
Subnet mask	255.255.0.0
Network address	150.215.0.0
Host address	0.0.017.009

All the systems on the same subnet must use the same subnet mask, in order to communicate with each other directly. Otherwise a gateway has to be used.

5.2.1.4. Ethernet

Ethernet refers to the family of local-area network (LAN) products covered by the IEEE 802.3 standard that defines what is commonly known as the CSMA/CD protocol. Three data rates are currently defined for operation over optical fiber and twisted-pair cables:

10 Mbps	10Base-T Ethernet
100 Mbps	Fast Ethernet
1000 Mbps	Gigabit Ethernet

5.2.2. Direct Connection to a PC

Follow the instructions of your operative system.

5.2.2.1. Windows Vista

1. Click the Windows **Start** button, open the **Control Panel**, and then double-click **Network and sharing center**.

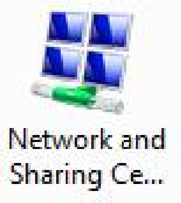

2. Select **Manage Network Connections** in **Network and Sharing Center**.

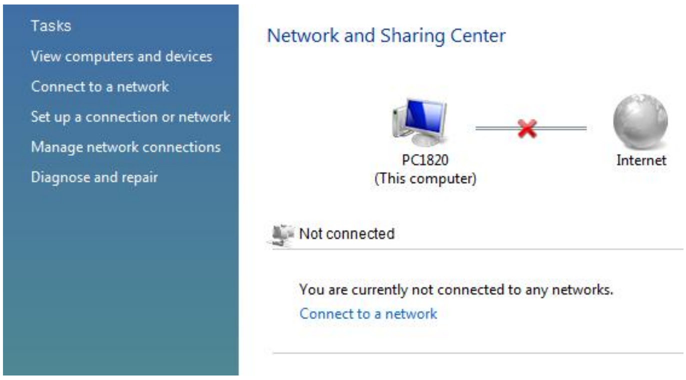

3. Right-click **Local Area Connection**.

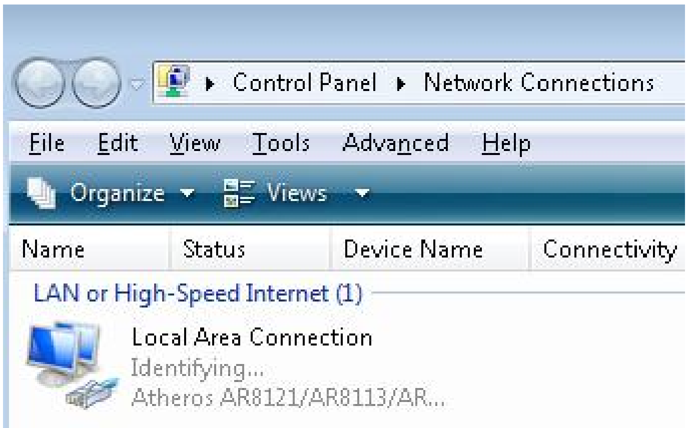

4. Click **Properties** in Local Area Connection to open Local Area Connection Properties.

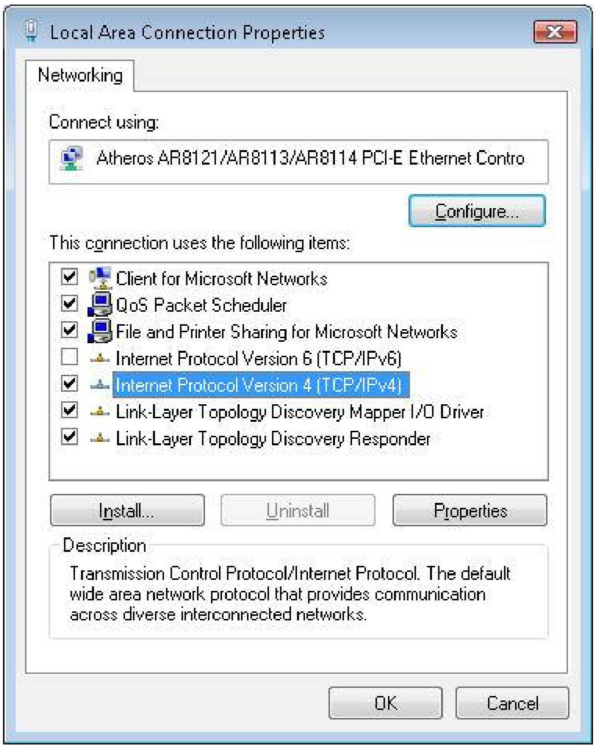

NOTICE

IMPORTANT NOTICE

DEACTIVATE INTERNET PROTOCOL VERSION 6 (TCP/IPv6)

Make sure that the Internet Protocol Version 6 (TCP/IPv6) is deactivated. Otherwise there might be some interferences in proper working of OmniPro I'mRT when this protocol is activated.

5. Click **Continue**, when the Windows permission message appears.
6. Select ***Internet Protocol version 4 (TCP/IPv4)***.
7. Click **Properties** to open **Internet Protocol version 4 (TCP/IPv4) Properties**.

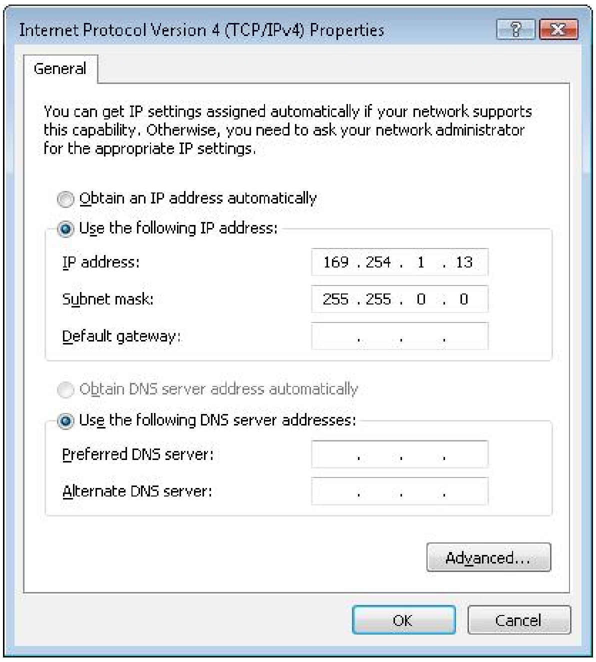

Mark the radio-button **Use the following IP address**, and type the IP address 169.254.1.5 in IP address.

8. Type 255.255.0.0 in **Subnet Mask**. Click **OK**.
9. Click **OK** to close the **Local Area Connection Properties** window.
10. Close the **Network Connections** window.
11. Connect the MatriXX to the PC with an Ethernet cross-over cable.
12. Start OmniPro I'mRT. Select ***MatriXX*** in the ***Equipment*** menu in OmniPro I'mRT, and click **Show online devices** in the **I'mRT MatriXX Setup** dialog. (There is also the possibility to click **New**, and manually define a device.)

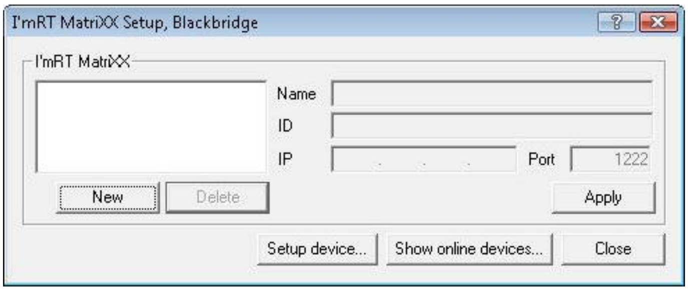

13. Data for the connected MatriXX will be displayed:

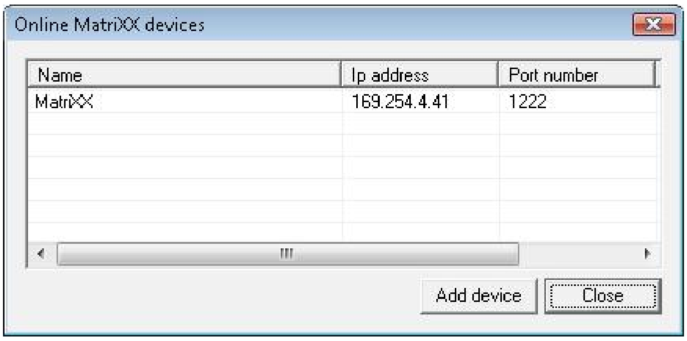

14. Mark the device and click **Add device**.

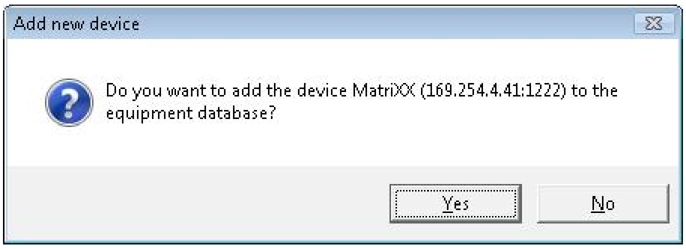

15.Click **Yes** to confirm. The I'mRT MatriXX Setup dialog opens.

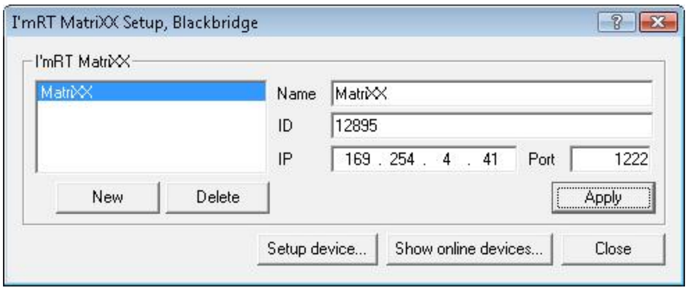

16.The Name and ID can now be changed/entered.

17.Click **Apply**, and then **Close**. The MatriXX is now installed.

5.2.2.2. Windows XP

1. Click **Start**, select **Control Panel**, and then double-click **Network Connections**.
2. Mark the connection that you want to configure, and then, under **Network Tasks**, click **Change settings of this connection**.

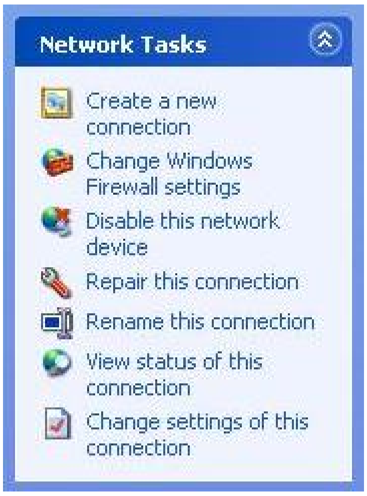

3. In the General tab, under this connection uses the following items, mark Internet Protocol (TCP/IP), and then click **Properties**.

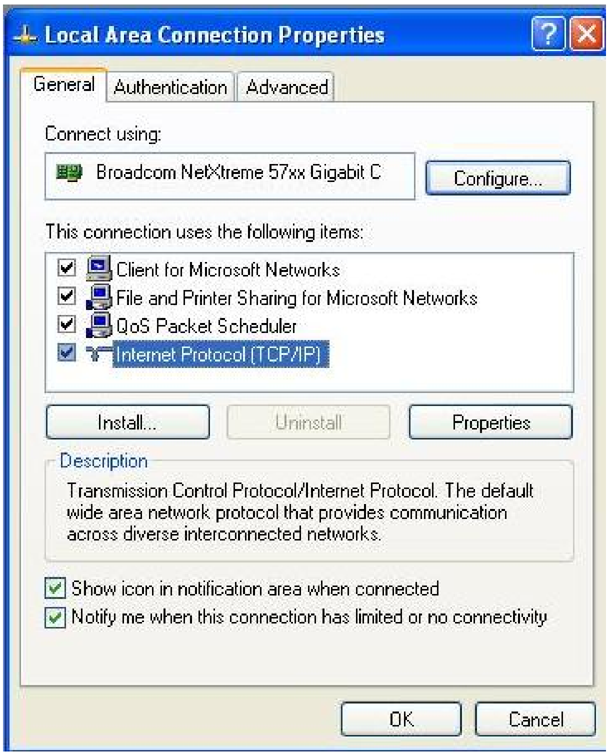

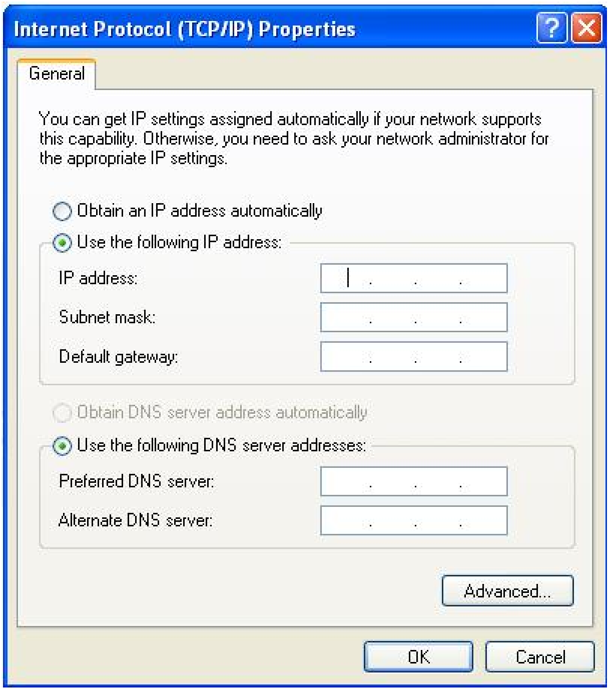

4. Mark **Use the following IP address**.
5. Type the IP address 169.254.1.5 in IP address.
6. Type 255.255.0.0 in **Subnet mask**.

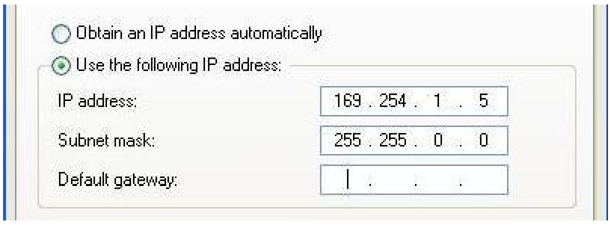

7. Click **OK** to save your new settings and to close the **TCP/IP Properties** window. If prompted, restart your computer.
8. Connect the MatriXX to the PC with the Ethernet cross-over patch cable.
9. Start OmniPro I'mRT. Select ***MatriXX*** in the ***Equipment*** menu in OmniPro I'mRT, and click **Show online devices** in the **I'mRT MatriXX Setup** dialog. (There is also the possibility to click **New**, and manually define a device).

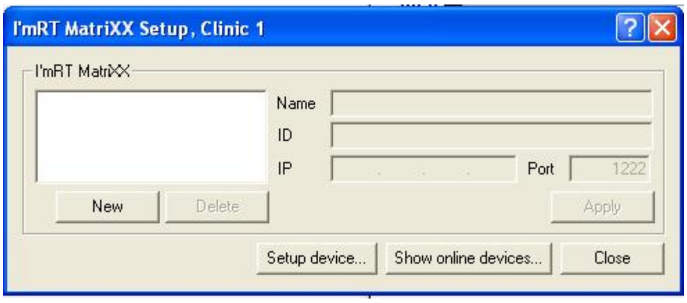

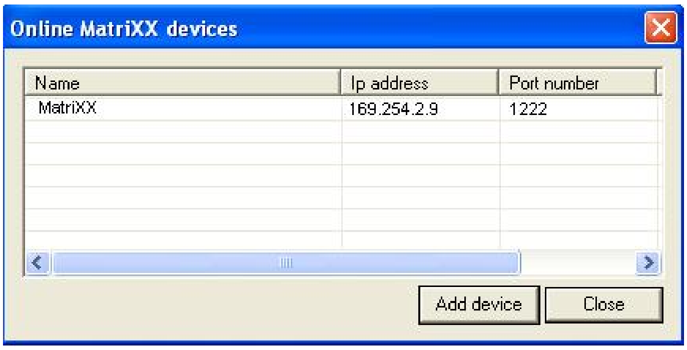

10. Data for the connected MatriXX is displayed.
11. Mark the device and click **Add device**.

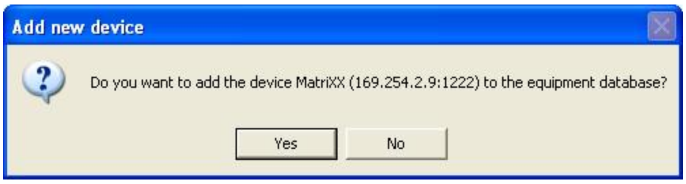

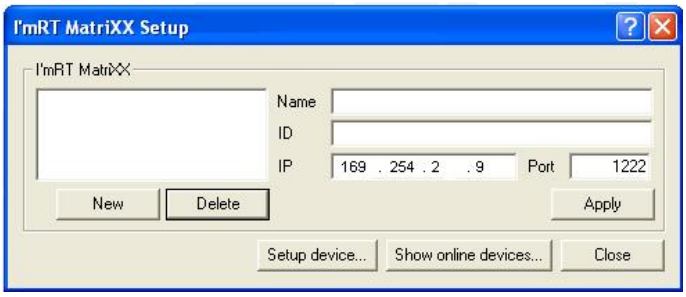

The **Name** and **ID** can now be changed/entered.

12. Click **Apply**, and then **Close**. The MatriXX is now installed.

5.2.2.3. Windows 2000

1. Click **Start**, select **Control Panel**, and then double-click **Network Connections**.
2. Double-click **Local Area Connection**.

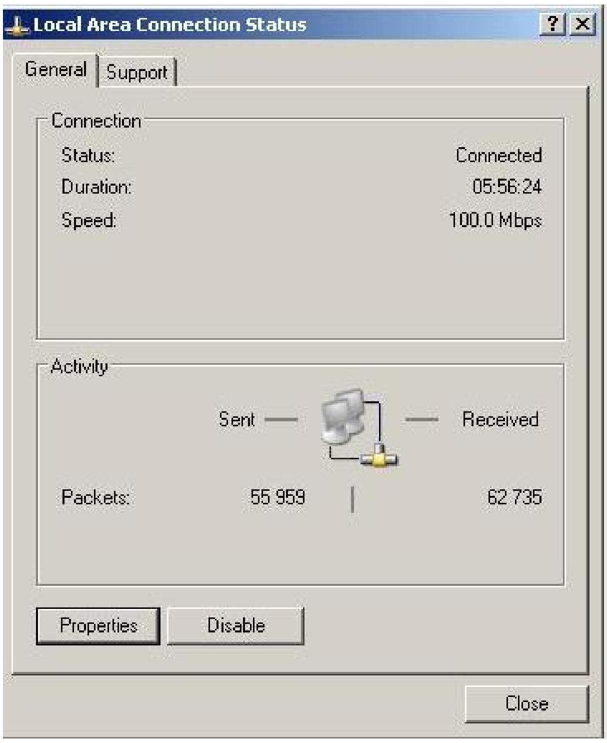

3. Click Properties in the **Local Area Connection Status** page.

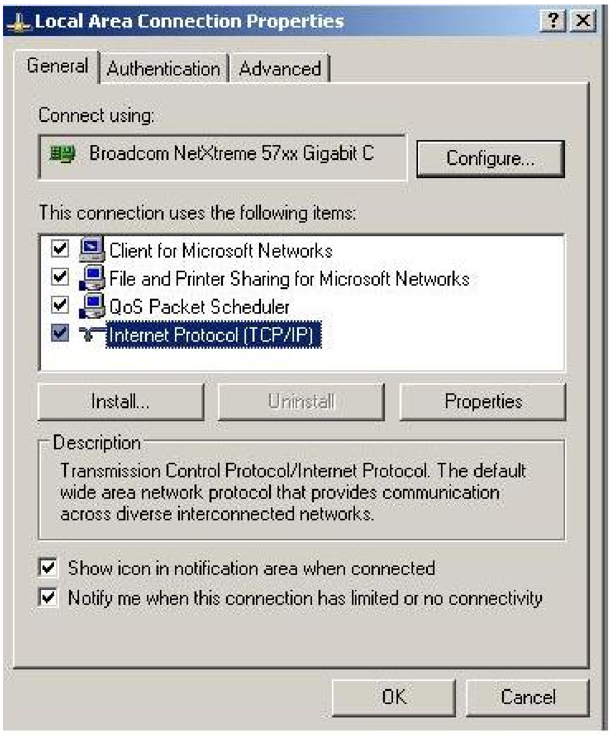

4. Mark **TCP/IP** under **Components checked are used by this connection**. Click Properties.
5. Take notes of the original settings.

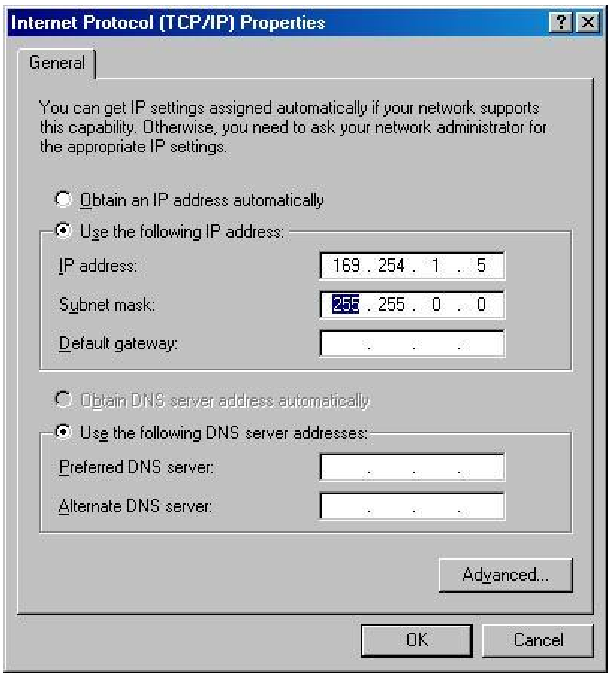

6. Mark **Use the following IP address**, and enter the IP address 169.254.1.5 in **IP address**.
7. Enter 255.255.0.0 in **Subnet mask**.
8. Click **OK** to save your changes and to close the **TCP/IP Properties** window. If prompted, restart your computer.
9. Connect the MatriXX to the PC with the Ethernet cross-over patch cable. Turn on the MatriXX.

10. Start I’mRT. Select ***I’mRT MatriXX*** in the ***Equipment*** menu, and click **Show online devices** in the **I’mRT MatriXX Setup** window. (There is also the possibility to click **New**, and manually define a device).

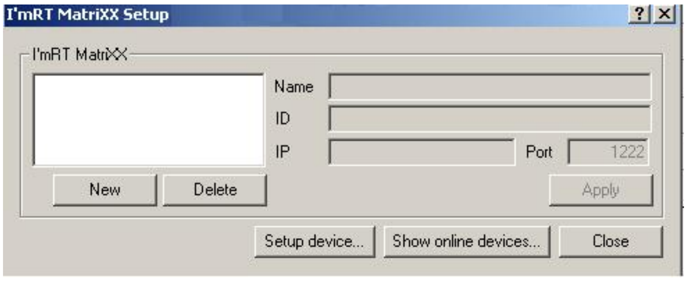

11. Data for the connected MatriXX will be displayed.

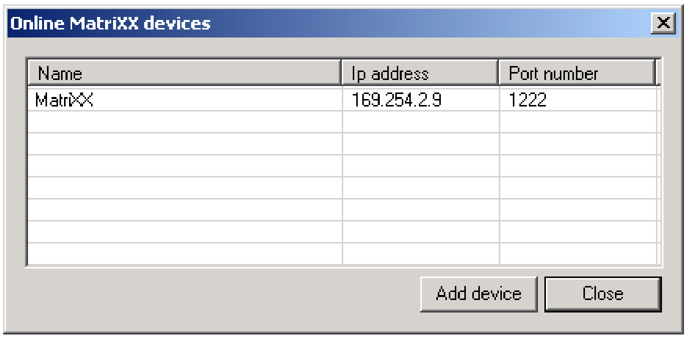

12. Mark the device, and click **Add device**.

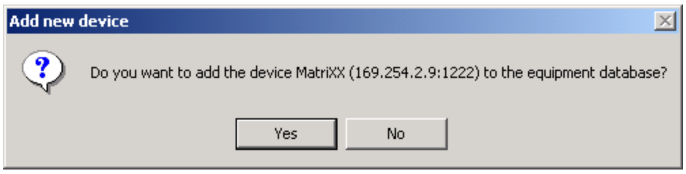

13. Click **Yes** to confirm.

14. The name and ID can now be changed/entered.

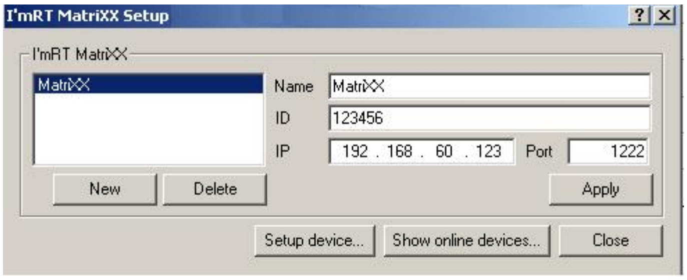

15. Click **Apply**, and then **Close**. The MatriXX is now installed.

5.2.3. Connection to a Local Network

5.2.3.1. Preparations

NOTICE

IMPORTANT NOTICE

NETWORK CARD OR ADAPTER

A network card for Windows XP Service Pack 2 or Windows 2000 Service Pack 4, or a network adapter for Windows Vista is required.

NOTICE

IMPORTANT NOTICE

CONSULT THE LOCAL IT ADMINISTRATOR

It is recommended that the local IT administrator is consulted before connecting the MatriXX to the local network.

NOTICE

IMPORTANT NOTICE

ALLOWED ACCESS TO THE NETWORK

Add the XX Setup tool to the list of applications that are allowed access to the local network.

Before connecting the MatriXX for the first time to the local network, a direct connection to the PC must be established, in order to set or modify the IP address of the device.

(See *5.2.2 Direct connection to a PC*).

5.2.3.2. Connection

1. After establishing the direct connection, start XX Setup.exe from the I'mRT folder (C:\Program Files\ IBA Dosimetry \OmniPro I'mRT).

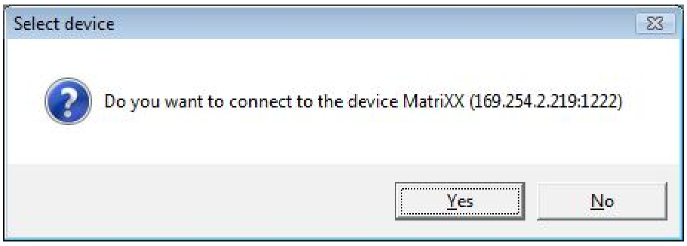

2. Click **Yes** to confirm.

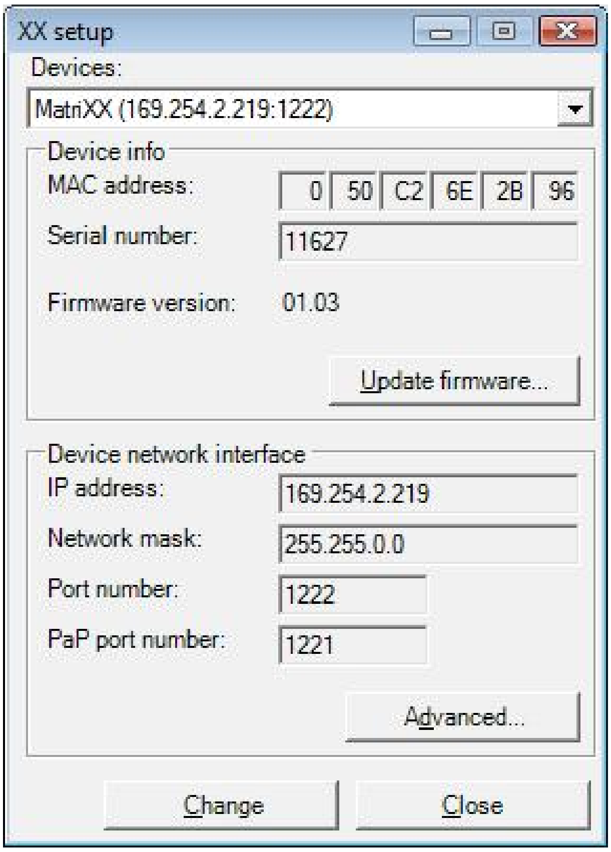

3. Click **Change** to activate the **Device network interface**.
4. Enter the (MatriXX) IP address, provided by your IT administrator, in the **IP address** field.
5. Enter the subnet mask, provided by your IT administrator, in the **Network mask** field (usually 255.255.255.0).
6. If your network uses a gateway to connect to the device, click the **Advanced button**, and enter the gateway information provided by your IT administrator.

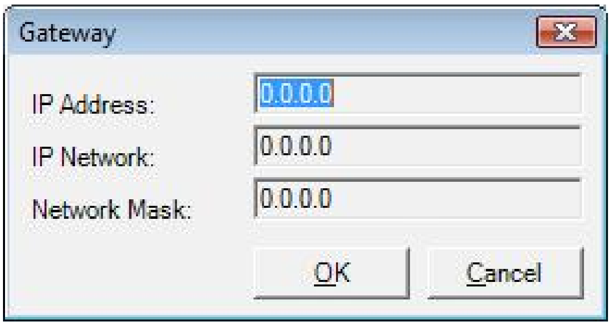

IP Address: Enter the IP address from the MatriXX side.

IP Network: Enter the IP address of the gateway on the far side from the MatriXX, i.e. at the PC side.

Network Mask: Enter 255.255.255.0.

7. Click **OK** to save the settings.
8. Click **Apply changes** in the **XX Setup** dialog.
9. Click **Yes**, and the MatriXX will be restarted automatically.
10. Power off the MatriXX.

11. Remove the direct Ethernet cross-over patch cable between the PC and the MatriXX, and connect both the PC and the MatriXX to the network.
12. Restore the original settings (See *5.2.2 Direct connection to a PC*).
13. Start OmniPro I'mRT. Select ***I'mRT MatriXX*** in the ***Equipment*** menu, and click **Show online devices** in the **I'mRT MatriXX Setup** window. (There is also the possibility to click **New**, and manually define a device.)

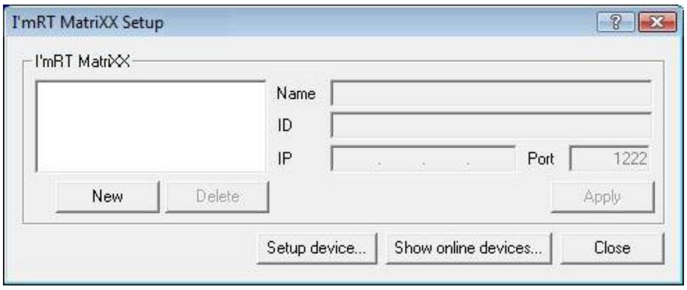

Data for the connected MatriXX will be displayed.

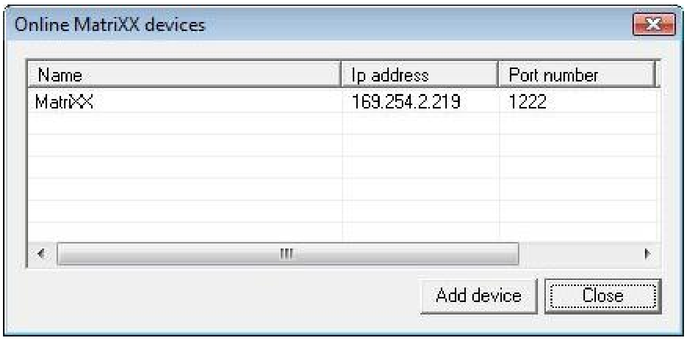

14. Mark the device, and select **Add device**.

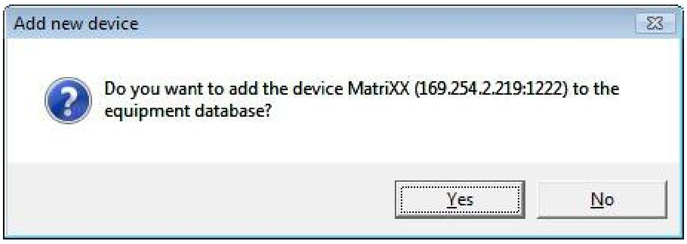

15. Click **Yes** to confirm.

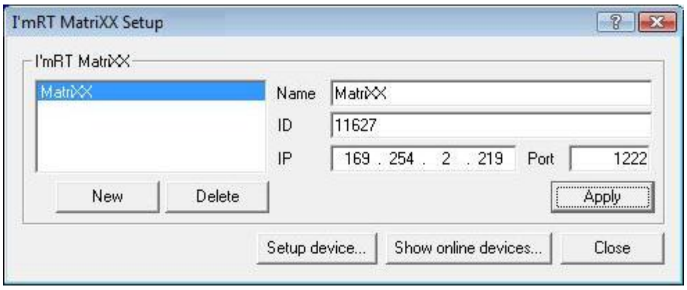

16. Name and ID can now be changed/entered.
17. Click **Apply**, and then **Close**. The MatriXX is now installed.

NOTICE

IMPORTANT NOTICE

RECOMMENDED CABLE LENGTH EXCEEDED

If no connection can be established, the reason may be that the recommended cable length is exceeded. The communication may be amplified by using an Ethernet mini-hub.

If you want to establish a direct connection between the MatriXX and the PC, after it has been connected to the local network, the same steps as described in *5.2.2 Direct connection to a PC* shall be followed. Instead of the default IP address and subnet mask (169.254.1.5 and 255.255.0.0), an IP address and a subnet mask fitting the current settings of the MatriXX shall be used.

If the IP address of a MatriXX device has been lost, it is possible to recover it by pressing the AUX button on the device (see *4.1.3.6 Connectors and LEDs*).

5.2.4. MatriXX Firmware

5.2.4.1. Firmware Update

Firmware updates will be delivered as a binary file. Select ***Equipment/I'mRT MatriXX*** or ***Equipment/MatriXX Evolution*** in OmniPro I'mRT. Open the XX Setup tool to install data.

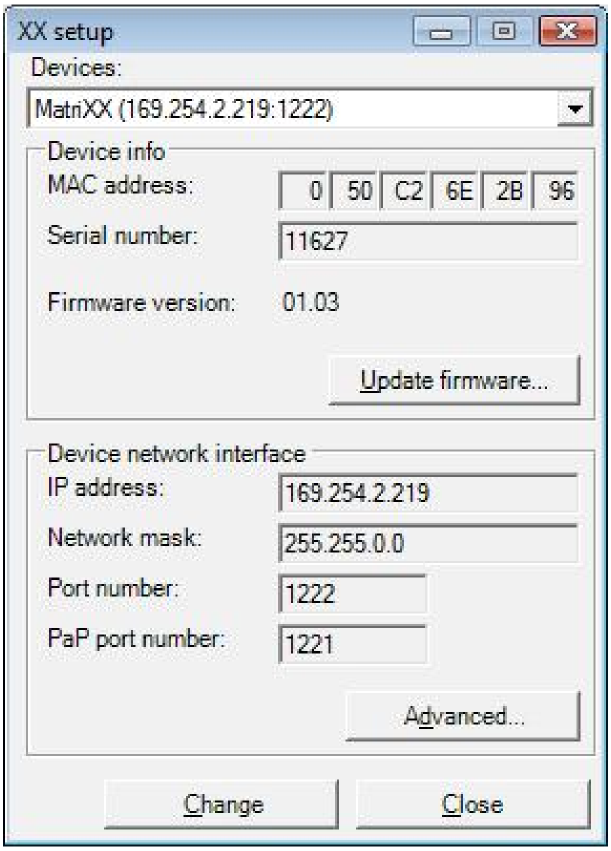

Click **Update firmware**. Browse for, and open the firmware update file.

The new firmware version will be installed.

Select **Yes** when you are prompted to restart the device.

NOTICE

IMPORTANT NOTICE

RESTART

Manual restart of the device is necessary. Turn power off and on to restart the device.

6. Getting started in OmniPro I'mRT software

6.1. Outline of OmniPro I'mRT

6.1.1. Windows Environment

The OmniPro I'mRT software runs under Microsoft® Windows® operating system.

It is assumed that you are familiar with the Microsoft Windows environment. If not, you are strongly recommended to read your Windows documentation first.

6.1.1.1. Printing and Plotting

Printing and plotting can be performed on any installed printer or plotter. To install new printers, choose ***Control Panel*** from the Windows ***Start*** menu and double-click on **Printers**. OmniPro I'mRT will use the default printer unless you select an alternative printer in the **Print Setup** dialog, which is opened by choosing the ***Print Setup*** command from the OmniPro I'mRT ***File*** menu.

6.1.1.2. Change Working Directory

You may want to change your OmniPro I'mRT working directory.

To change directory:

Select ***Programs*** from the Windows ***Start*** menu, right-click the **OmniPro I'mRT** icon and choose the ***Properties*** command from the drop-down menu. The **Properties** dialog opens.

Change working directory and click **OK**.

6.1.2. Screen Structure

6.1.2.1. Panes

The screen is divided into four panes. See *6.1.9 Panes, Single Data Set*, and *6.1.10 Panes During Plan Verification*, for further descriptions.

6.1.3. Main Menu Bar

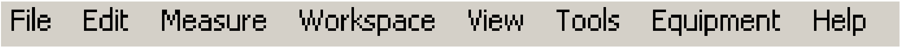

There are detailed menu descriptions in the OmniPro I'mRT on-line help, however, brief descriptions follow below.

6.1.3.1. File Menu

The File menu contains common software commands, e.g. ***Open***, ***Save***, ***Print***, and ***Exit***.

6.1.3.2. Edit Menu

The ***Edit*** menu contains commands to modify data, e.g. smoothing, rescaling, and CAX correction.

6.1.3.3. Measure Menu

From the ***Measure*** menu you can choose which measurement device to use. It also contains commands for editing, copying, loading, and saving measurement parameters.

6.1.3.4. Workspace Menu

In the ***Workspace*** menu you can select which data set to be displayed.

6.1.3.5. View Menu

In the ***View*** menu you can select how the active data set should be displayed. Other view commands, e.g. for zooming and applying cursors, are also found here.

6.1.3.6. Tools Menu

The **Options** dialog, where you choose units and define parameters, is opened from the ***Tools*** menu.

6.1.3.7. Equipment Menu

The ***Equipment*** menu provides commands for the setup of the devices.

6.1.3.8. Help Menu

The ***Help*** menu contains a command for opening of the **About** dialog, where information of the software is displayed, and a command to open the on-line help.

6.1.4. Main Toolbar

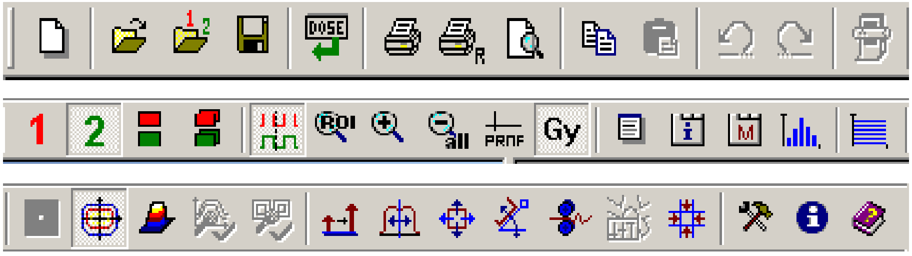

The tool buttons provide shortcuts to common menu commands, such as "selection of the data set to be displayed":

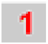 Displays dataset 1 in all panes.

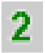 Displays dataset 2 in all panes.

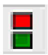 Displays two 2D data sets for comparison.

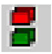 Displays two 3D data sets for comparison.

To get a brief description of the command, move the mouse over the tool button until a tool tip is displayed, or look at the information text in the status bar. Depending on the data displayed, some commands in the toolbar may not be available (dimmed).

To view/hide the toolbar, select ***Workspace:Toolbar***.

Refer to the OmniPro I'mRT on-line help for detailed descriptions of all buttons.

6.1.5. I'mRT QA / BIS Toolbar

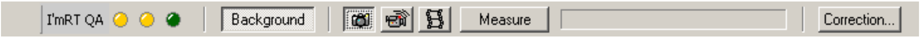

When I'mRT QA/BIS is selected as the measurement device, the OmniPro I'mRT QA/BIS toolbar will be expanded.

The I'mRT QA/BIS toolbar contains status indicators, selection buttons for the measurement mode, start button, and a button for background definition.

6.1.6. I'mRT MatriXX Toolbar

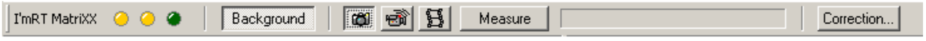

When I'mRT MatriXX is selected as the measurement device, the OmniPro I'mRT MatriXX toolbar will be expanded.

The I'mRT MatriXX toolbar contains status indicators, selection buttons for the measurement mode, start button, and a button for background definition.

6.1.7. Status Bar

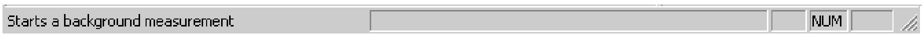

The status bar, at the bottom of the main window, displays information about current actions, and provides brief help texts for menu and toolbar commands. To display or hide the status bar, select ***Workspace:Statusbar***.

6.1.8. Panes

Four different panes are used to display the data. The type of data displayed depends on what kind of data is selected (one data set or two data sets for comparison).

The following general commands are used to select the view:

Toolbar icons

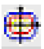 Shows the data as isodose contours.

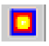 Shows the data as a two-dimensional array.

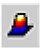 Shows the data as a three-dimensional array.

Pane icons

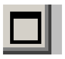 Maximizes a single pane to full window display.

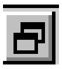 Restores a maximized window to the previous window size.

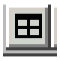 Resets all panes to default sizes.

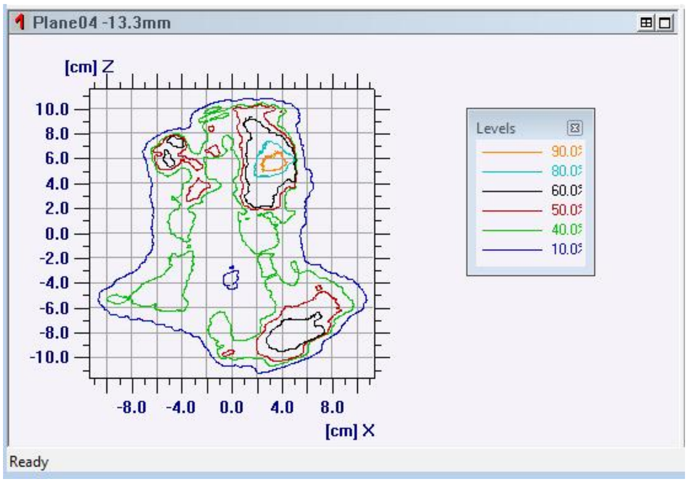

An example of the isodose view. Data is displayed as isodose contours. The isodose levels are user definable.

6.1.9. Panes, Single Data Set

When a single data set is selected (***Workspace:Data set 1*** or ***Data set 2***), all four panes are used to display this data set:

Array Pane: Shows the data as a 2D array.

Isodose or 3D-Pane: Shows the data either as Isodose contours or in a 3D view.

Profile Panes: Shows profiles along the main axes. The profile offset to the origin can be defined when selecting the ***View:Profile Cursor*** inside the **Array Pane**.

See also *9.4 Analyzing one single data set*.

6.1.10. Panes during Plan Verification

When Plan Verification 2D or 3D is selected, two panes are used to display the two single data sets. The other two panes are used for comparison.

Data Set 1 (or 2) Pane: Displays the data set 1 (or 2) as a 2D array, an Isodose contour, or in a 3D View.

Comparison Pane: Displays isodoses or profiles of both data sets in the same window.

Result Pane: Displays the result of a mathematical comparison between the two data sets. Possible operations are, for example, ***Difference***, ***Gamma method***, or ***DTA method***.

In the **Comparison Pane** you can either compare isodoses or profiles:

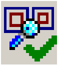

Shows the isodose levels of both data sets in one single pane. Isodose levels can be defined when selecting ***View:Isodose Levels*** inside the data set 1 and 2 panes. For example, click in data set 1 pane, select ***View:Isodose***, and then ***View:Isodose Levels***. (Or right click in the isodose pane and select ***Isodose Levels Set 1*** (or ***2***)).

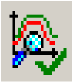

Shows one profile of each data set along one of the main axes. The offset to the origin can be defined when selecting ***View:Profile Cursor*** inside the **Array Pane** of each data set. To switch over to the other axis, right click in the pane and select the other axis.

6.1.11. Data Types

6.1.11.1. Workspace

The workspace files contain data from the entire workspace.

File type	Description
*.opw	OmniPro I'mRT workspace file. The workspace file does not contain any 2D dose distribution data itself, but rather information about where data that belong together are saved. It can contain the link to two data set files (*.opd) and one result file (with the name workspace name_res.opd). Additionally it can contain Absolute Dose verification data and comments.

6.1.11.2. Data Set

Measured data or calculated dose distributions are saved as data sets. Two data sets plus a result file can be linked together as a workspace.

File type	Description
*.opd	OmniPro I'mRT data set file. Contains data from measurements or from calculations (result file or imported calculated dose distribution).
*.opf	OmniPro I'mRT film data.
*.opq	OmniPro I'mRT Generic ASCII data
*.trs	"Torso"-file format. I'mRT QA/BIS measurements performed with the stand alone software, or in OmniPro-Accept, are saved as *.trs files.
*.bri	Data from VidiScan, VidiLum, or VidiBISoftware (e.g. measurements performed with BIS710 software) are saved in the *.bri format.<br><br>OmniPro I'mRT can both read and export data in *.bri format. See *Data Output / Export Data*.
*.arr	WP700 2D Array file (e.g. 2D film data).

OmniPro I'mRT can read *.bri/*.cal, *.arr, and *.trs files.

6.1.11.3. TPS Data

RTPS (Radio-Therapy Planning System) format files from CadPlan, Pinnacle, CMS, and BrainLAB, and DICOM RT and RTOG format files from any RTPS can be imported.

6.1.11.4. Other Data

File type	Description
*.opt	OmniPro I'mRT template file. The file contains a parameter set. The setup in the **Measurement Parameters** dialog can be saved and used for new measurements. Load the parameters from a parameter file, with the menu command ***Measure:Load parameters***.

6.1.12. Open Data

6.1.12.1. Open Workspace

A workspace file contains data from the entire workspace. One workspace file can contain one or two data set files, and a result file.

Select ***File:Open Workspace***. A standard **Open** dialog opens.

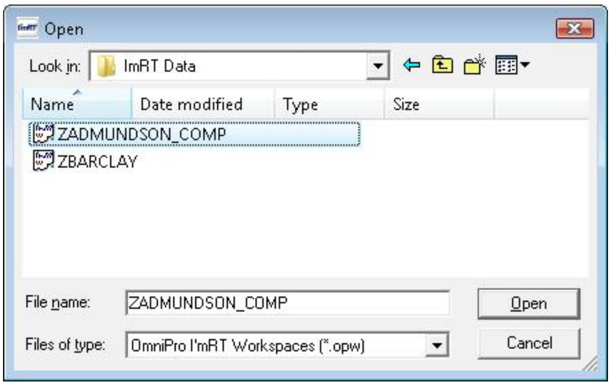

Browse for, and select an *.opw file. Click **Open**.

6.1.12.2. Open Data Set

A data set file contains data from measurements or from calculations (result file).

Select ***File:Open Data Set***. The **Open Data Set** dialog opens.

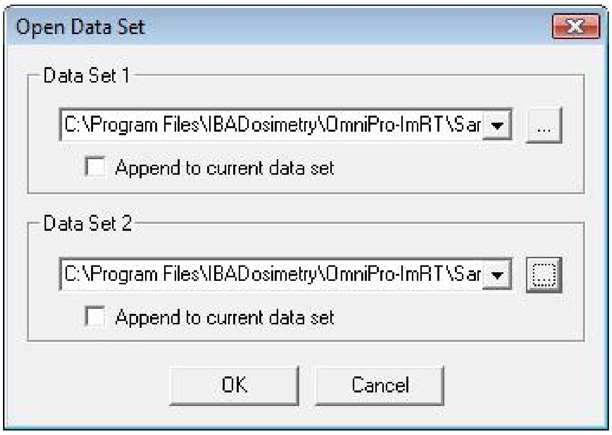

One or two data set files can be opened. Click [...].

A standard **Open** dialog opens.

Browse for an .opd file. Click **Open**. (It is also possible to open *.trs, *.bri and *.arr files.)

If you want to add the data to the open data set, check **Append to current data set**. Use the **Field list** dialog to select data from the data set. To select all data in the **Field list**, press <Ctrl + A>.

6.2. Configuration of OmniPro I'mRT Software

The OmniPro I'mRT system needs to have information about the equipment that is used with the system, such as the clinic, scanners, detectors, film type and the radiation device. Normally this is done directly after you have installed OmniPro I'mRT. Prior to measurements it is then quite easy to select the used equipment from a drop down list. (It is possible to add, modify, or remove equipment at any time.)

Choose the category of equipment that you want to configure from the ***Equipment*** menu:

Clinic: Enter administrative data about your clinic. This information will be stored together with the measurements.

Radiation Devices: Specify the accelerators including energies, wedges, etc.

I'mRT QA/BIS: Define the I'mRT QA/BIS.

I'mRT MatriXX: Define the I'mRT MatriXX.

MatriXX Evolution: Define the MatriXX ^{Evolution}.

Film: Define the type of films you are going to use.

Film Scanner: Define the settings for the film scanner to be used with OmniPro I'mRT.

Body Phantom: Define the body phantom.

Detectors: Not applicable in this SW version.

See the on-line help for detailed descriptions for the configurations.

6.2.1. Specify Units and Parameters

There are a number of items, related to parameter calculation and the user interface, which can be defined according to your preferences. This is done in the **Options** dialog. Open the dialog by selecting the menu command ***Tools:Options***.

6.2.1.1. Environment Tab

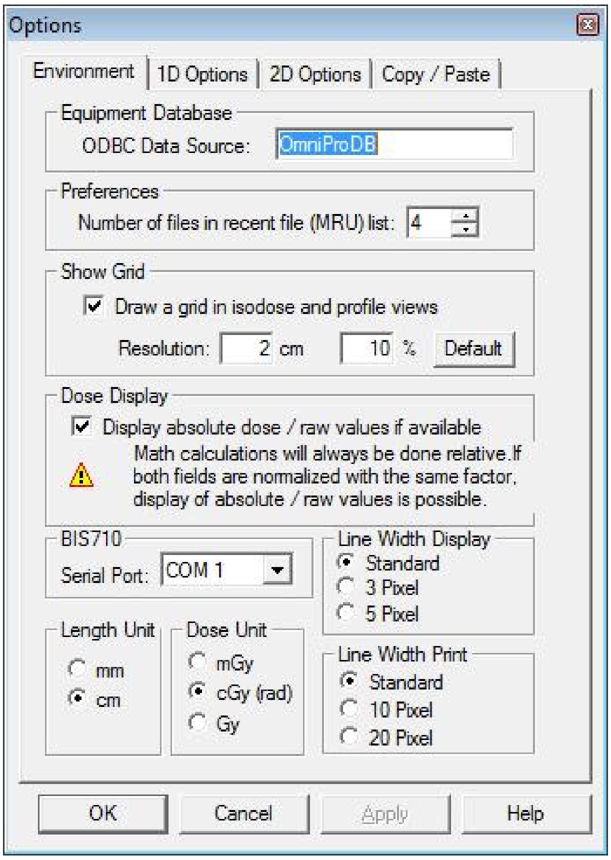

Under this tab you can:

- Specify the ODBC Data source. See *12.1.1 ODBC Data source* for further description.
- Specify if a grid shall be drawn in the views.
- Specify if absolute/raw values shall be displayed.
- Select units for distance and dose.
- Select the serial port to which the BIS 710 controller is connected.
- Define the line width for graphs.

Refer to the OmniPro I'mRT online help for further description.

6.2.1.2. 1D Options Tab

Parameters may be calculated either according to pre-defined protocols, or according to a protocol defined by the user.

6.2.1.2.1. Pre-defined Protocols

A number of pre-defined protocol options are available for electron and photon profile measurements:

- DIN
- IEC
- JARP86
- Elekta
- GE
- Siemens
- Varian

(See *14 Algorithms* and *14.2 References*).

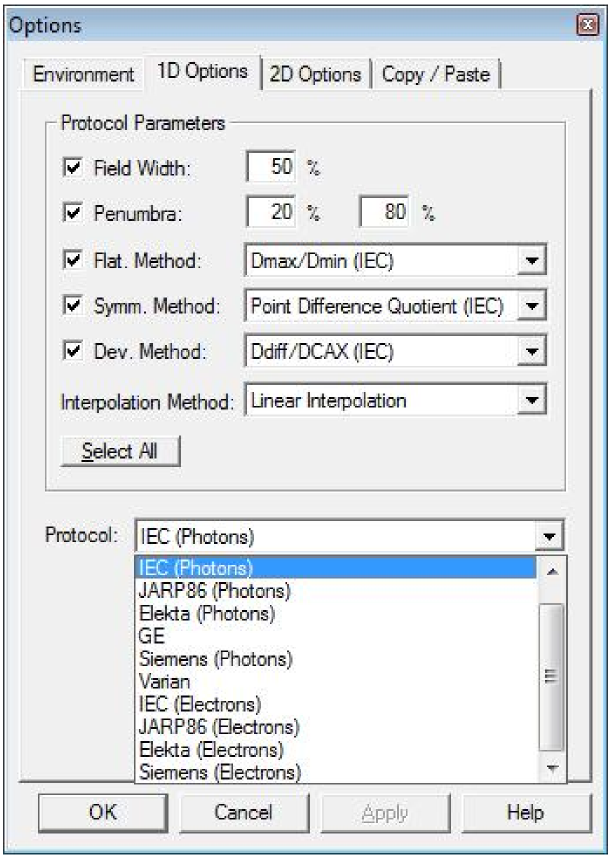

Select the protocol you want to use.

The parameters will be displayed in the profile view:

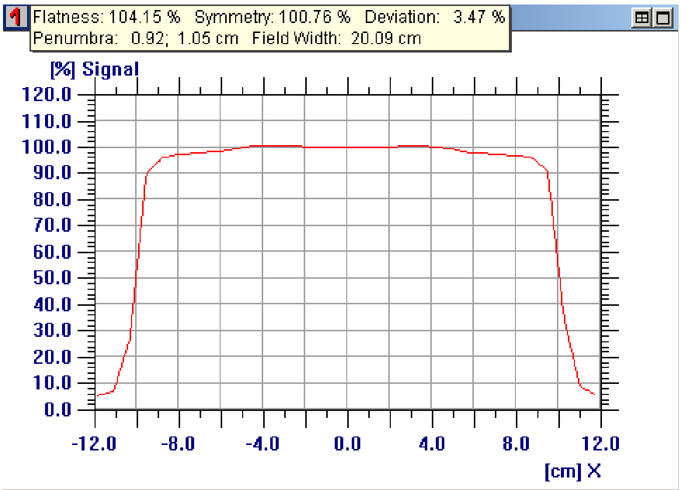

The calculation methods are described in *14 Algorithms*.

6.2.1.2.2. *User-defined Protocols*

To define a customized protocol, options for calculation methods for field width, penumbra, flatness, symmetry, and deviation, are available.

The Fermi-Fit algorithm is an option in Interpolation Method. It improves the determination of the 50% point in penumbrae of open rectangular fields, or MLC delimited segments parallel to the leaf direction.

See also *14.1.1.3 FermiFit Interpolation*.

NOTICE

IMPORTANT NOTICE

INTENSITY MODULATED FIELDS

The FermiFit algorithm is not intended to be used on intensity modulated fields.

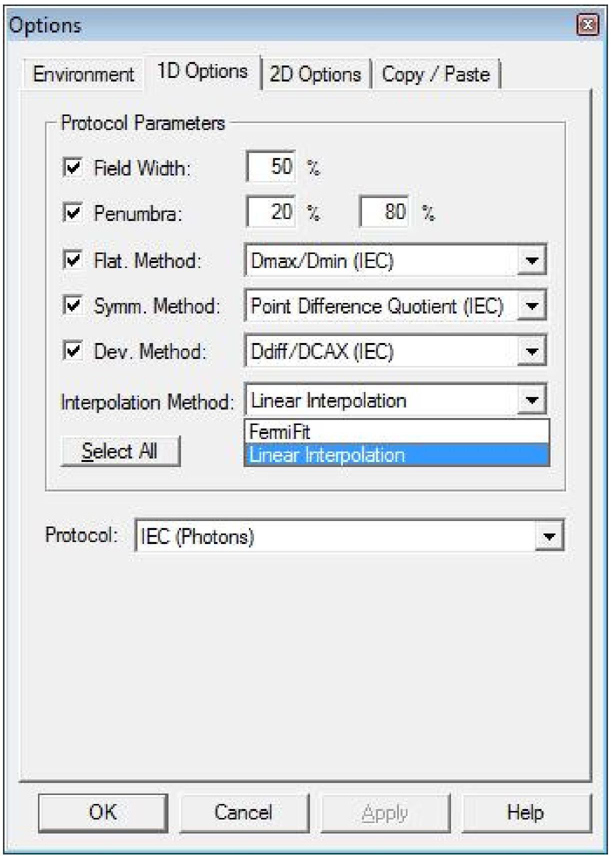

When a parameter in one of the pre-defined protocols is changed, the protocol name will be cleared. Thus the user-defined protocol will not have a name assigned to it.

Calculation methods: See *14.1.1.1 User-defined protocols*.

6.2.1.3. 2D Options Tab

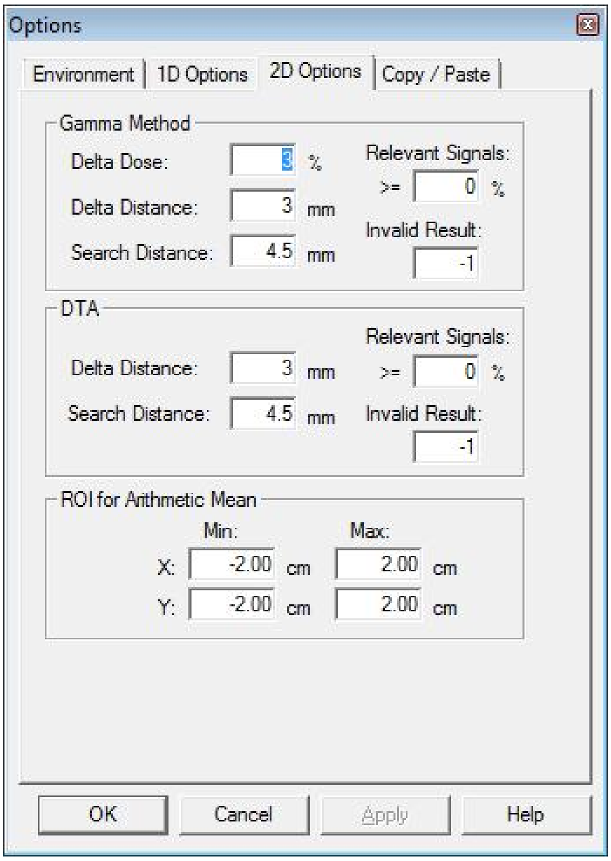

Under this tab you can:

- Specify the parameters for the Gamma method.
- Specify the parameters for the DTA method.
- Define the size and location of the region of interest (ROI).

Search distance: This is the search range for the Gamma algorithm. It should always be larger or equal to the criterion of acceptance. Normally it is sufficient to set the value exactly the same as Delta Distance.

However, if you also like to have values larger than 1, regarding the local aberration with the Gamma method, you must search also beyond this limit. Thus you will see if the gamma value is not correct (Gamma > 1), and the size of deviation as well (e.g. Gamma = 1.5).

Relevant Signal: Defines a threshold value to take underground signals (non irradiated areas) out of statistics.

Invalid Result: Value assigned to all pixels that are below the threshold value. If you assign i.e. -1 for these pixels they will be displayed in the histogram later on but will not be taken into account.

Refer to the OmniPro I'mRT online help for further description.

6.2.1.4. Copy/Paste tab

2D data can be copied and pasted between datasets within the OmniPro I'mRT application, or from OmniPro I'mRT to an external program in ASCII format (e.g. to Excel and Notepad).

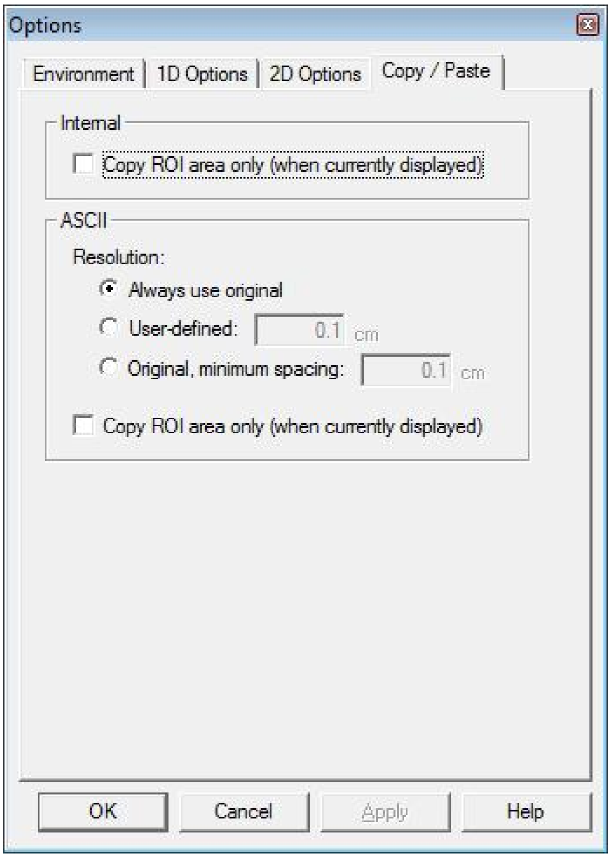

Under this tab you can:

- Specify whether you always want to copy the entire image, or use the region of interest (ROI).
- Define the output resolution for Copy/Paste to an external application.

Refer to the OmniPro I'mRT online help for further description.

7.Setup of MatriXX for measurements

7.1. Setting up I’mRT MatriXX

The diagram below shows the general placement of the MatriXX, the power supply, the PC, and the connections between them.

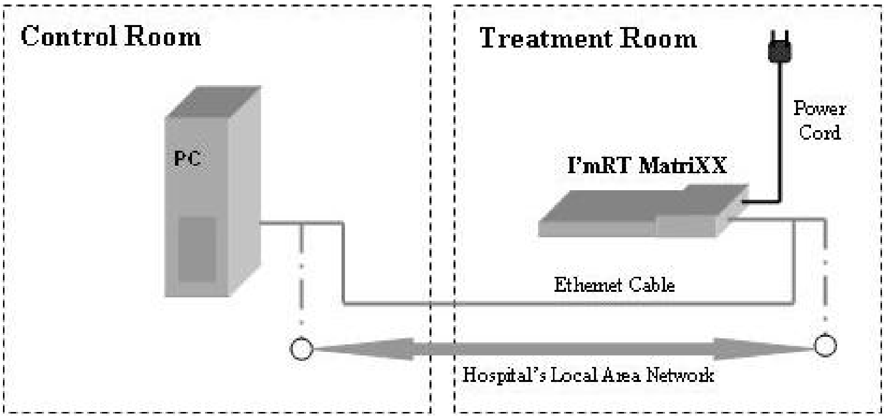

For measurements the MatriXX is placed under the gantry head, either on the treatment couch or on a lifting table, as shown in the figure below, or mounted on the gantry. It is connected to the PC in the control room via an Ethernet cable, or via an existing LAN network.

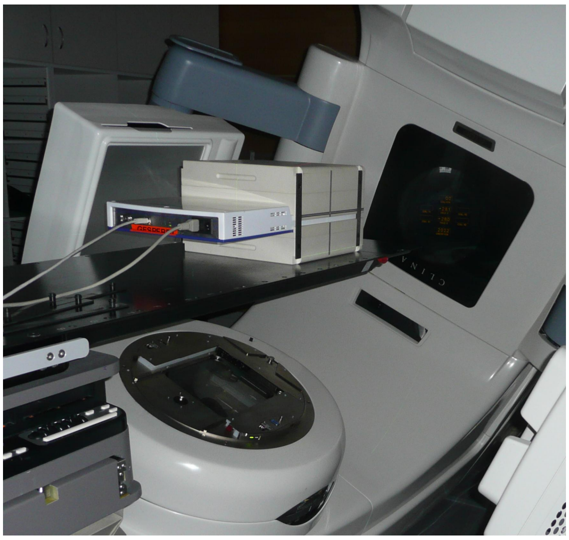

7.1.1. Mounting I'mRT MatriXX

There are two options for the mounting of I'mRT MatriXX for measurement: table mounted or head mounted.

7.1.1.1. Table Mounted

1. Place the I'mRT MatriXX either in the MULTICube or with build-up plates and backscatter plates onto the treatment couch under the treatment head.

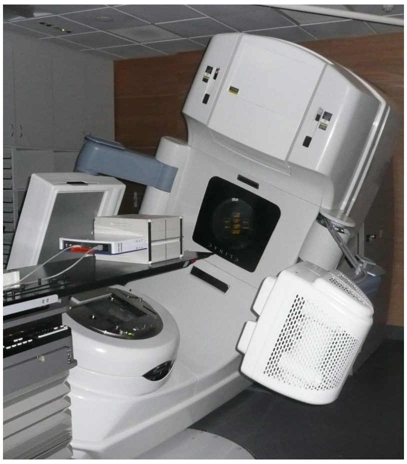

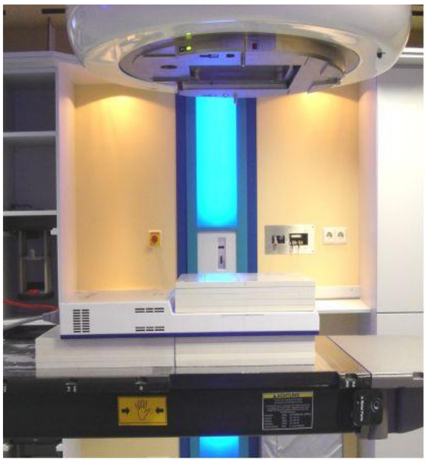

2. The lasers in conjunction with the side cross makers of the MULTICube or on the I'mRT MatriXX can be used to position the device in the center of the beam.
3. Align I'mRT MatriXX, using a spirit level placed on the detector area.
4. At the accelerator, set the field size 20 × 20 cm, and turn on the light field.
5. Use the adjustment capabilities of the treatment couch, or the lifting table, to position I'mRT MatriXX so that the crosshairs at the sensor area and the light field crosshairs are superimposed.

NOTICE

IMPORTANT NOTICE

USE THE SIDE MARKERS FOR POSITIONING

Due to the higher uncertainty when positioning the device in the light field (diffuse penumbras and cross-hair width), for precise positioning it is advisable to refer to the side markers which are engraved in the housing.

6. Ensure that the cables to the PC or the network are properly connected.
7. Plug the power cord into a mains power outlet, and switch on the MatriXX. Verify that only the green LED is illuminated.

Allow an appropriate warm-up time, and pre-irradiate the device during the warm-up (See *7.3.2 Pre-irradiation and Warm-up*).

NOTICE

IMPORTANT NOTICE

SETUP MUST BE EQUAL TO THE CT SCAN SETUP

Make sure that the setup for measurements is equal to the setup while scanning the MatriXX in CT. Please refer to chapter *3.1.1 Scanning of MatriXX in CT scanner.*

7.1.1.2. Head Mounted

For mounting of the MatriXX on the gantry, adapters are available (see *4.2.4 Gantry Adapters*).

1. Mount the MatriXX, the backscatter plates, and the build-up plates (see *7.1.1.3.1 Mounting the Backscatter Holder and Plates*, and *7.1.1.3.3 Build-up Plates*).
2. Mount the gantry adapter (See *7.1.1.3.5 Mounting of Gantry Adapters*).
3. Mount the complete equipment on the gantry.
4. At the accelerator, set the field size 20 × 20 cm, and turn on the light field.
5. Adjust the XY-table (see *7.1.1.3.4 Adjust the XY Table*) so that the crosshairs at the sensor area of the MatriXX and the light field crosshairs are superimposed.

NOTICE

IMPORTANT NOTICE

USE THE SIDE MARKERS FOR POSITIONING

Due to the higher uncertainty when positioning the device in the light field (diffuse penumbras and cross-hair width), for precise positioning it is advisable to refer to the side markers which are engraved in the housing.

6. Ensure that the cables to the PC or the network are properly connected.
7. Plug the power cord into a mains power outlet, and switch on the MatriXX. Verify that only the green LED is illuminated.

Allow an appropriate warm-up time, and pre-irradiate the device during the warm-up (See *7.3.2 Pre-irradiation and 7.3.37.3.2 Warm-up*).

CAUTION

CAUTION

ALL ACCESSORIES MUST BE LOCKED TO THE SUPPORT FRAME

The total weight of the support frame with the MatriXX with 50 mm build up, backscatter holder with 100 mm backscatter plates, and the gantry holder, is 37.9 – 39.6 kg (depending on the type of gantry holder).

Ensure that all knobs and safety clamps are firmly tightened before starting the measurement. Check: Try to pull the MatriXX out of the holder. It must not be possible to move it.

CAUTION

CAUTION

MATRIXX MUST STAY CLEAR OF SURROUNDING EQUIPMENT

Ensure that the mounted MatriXX stays clear of the patient couch or other equipment, when the gantry is rotated.

CAUTION

CAUTION

ENTER GANTRY ADAPTER INFORMATION TO THE LINAC SOFTWARE

Ensure that the gantry adapter is accepted as an accessory by the Linac software; otherwise interlocks may occur.

7.1.1.3. Mounting Equipment into the Support Frame

NOTICE

IMPORTANT NOTICE

START WITH THE BACKSCATTER HOLDER AND PLATES

If backscatter plates are going to be used, start by mounting the backscatter holder and plates.

7.1.1.3.1. Mounting the Backscatter Holder and Plates

Backscatter plates can be mounted from 10 to 100 mm, in steps of 1, 2, 5, and 10 mm.

The delivered backscatter plates are made of water equivalent polystyrene, RW3.

NOTICE

IMPORTANT NOTICE

BACKSCATTER HOLDER

The plates must be positioned and locked in the backscatter holder.

1. Position the plates into the backscatter holder, and lock them with the knobs at the sides of the holder (a). Thin plates should be positioned at the bottom of the stack.

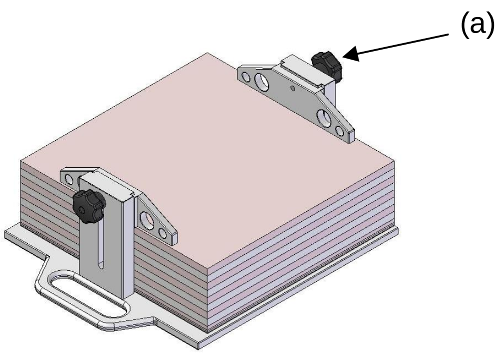

2. Loosen the four knobs at the bottom of the holder (b). Insert the backscatter holder into the frame.

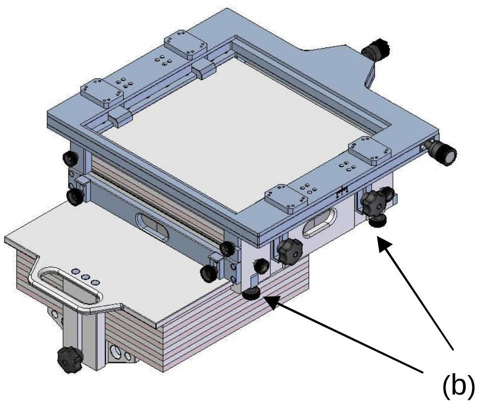

Insert the MatriXX (see *7.1.1.3.2 Mounting the MatriXX in the Support Frame*). Lock the backscatter holder with the four knobs (b). These knobs will also lock the MatriXX to the frame.

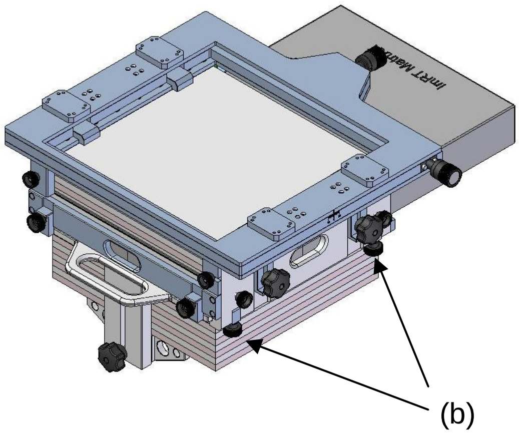

CAUTION

CAUTION

ENSURE THAT ALL KNOBS AND SAFETY CLAMPS ARE PROPERLY TIGHTENED.

The design of the holder is based on friction, therefore proper tightening of the screws is essential.

7.1.1.3.2. Mounting the MatriXX in the Support Frame

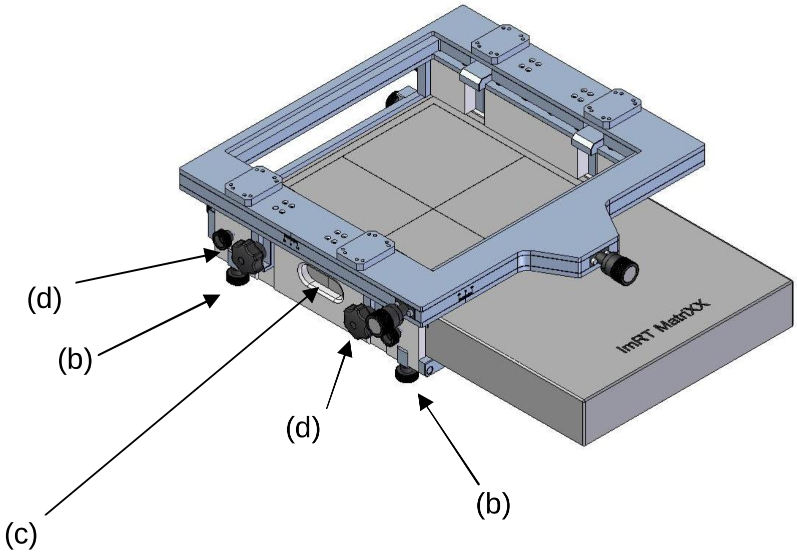

When inserting the MatriXX into the support frame, ensure that it will be positioned tightly to the wall of the frame. The openings in the frame allow the markings for laser adjustment to remain visible (c).

1. Move the MatriXX cautiously to the opposite wall of the frame, by turning the two knobs (d) on the side of the frame. Do not tighten the knobs.

2. Use the four knobs (b) in the bottom of the frame to lift the MatriXX to the upper surface of the frame, so that the distance to the isocenter will be correct (100 or 76.2 cm). These knobs will also lock the MatriXX to the frame.

NOTICE

IMPORTANT NOTICE

SURFACE OF THE DETECTOR AREA

The surface of the detector area is positioned 3 mm below the MatriXX housing surface. The water equivalent thickness is 3.2 mm.

3. Tighten the knobs (b), and then the knobs (d).

CAUTION

CAUTION

LOCK THE MATRIXX TO THE FRAME

Ensure that the MatriXX is locked with the four knobs in the bottom of the frame. The two knobs on the side of the frame will not lock the MatriXX. Check: Try to pull the MatriXX out of the holder. It must not be possible to move it.

7.1.1.3.3. Buildup Plates

Build up can be mounted up to 5 cm, in steps of 1 mm.

The delivered build up plates are made of water equivalent polystyrene, RW3.

The water equivalent depth of the MatriXX housing is 3.2 mm.

1. Remove the safety clamps (a).

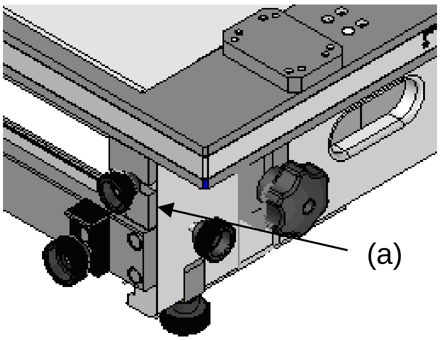

Tip! Turn the clamps 180° and put them on the knob again, to keep them readily at hand.

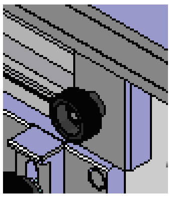

2. Position the plates on the MatriXX surface, in the frame. Lock the plates from above with the four clamps on the side of the frame (b).

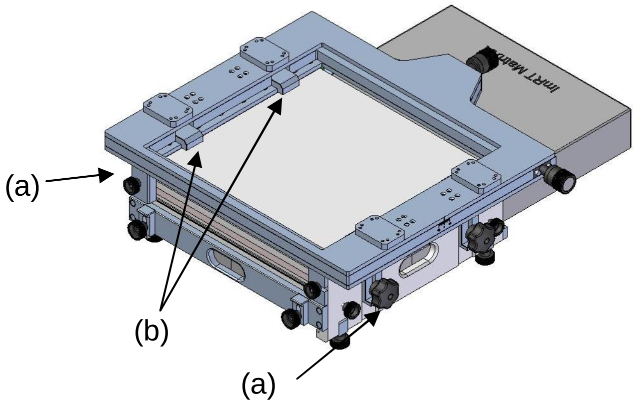

3. Fasten the safety clamps (a) again.

CAUTION

CAUTION

ENSURE THAT ALL CLAMPS (A) AND (B) ARE TIGHTENED

All clamps must be tightened, otherwise the plates may slide out.

7.1.1.3.4. Adjust the XY Table

NOTICE

IMPORTANT NOTICE

BACKSCATTER PLATES AND BUILD-UP PLATES

If backscatter plates and build up plates are going to be used, adjust the XY table after the backscatter holder and the build up plates have been mounted and locked.

NOTICE

IMPORTANT NOTICE

MATRIXX MOUNTED ON THE GANTRY

If the MatriXX is going to be mounted on the gantry, adjust the XY-table after the complete equipment, the gantry adapter included, has been mounted on the gantry (see *7.1.1.3.5 Mounting of the Gantry Adapters*).

XY Table, Standard Version

Loosen the four knobs in the corners of the upper plate of the XY table sufficiently to move the plate. Adjust the plate manually, and fasten the knobs again.

XY Table, Advanced Version

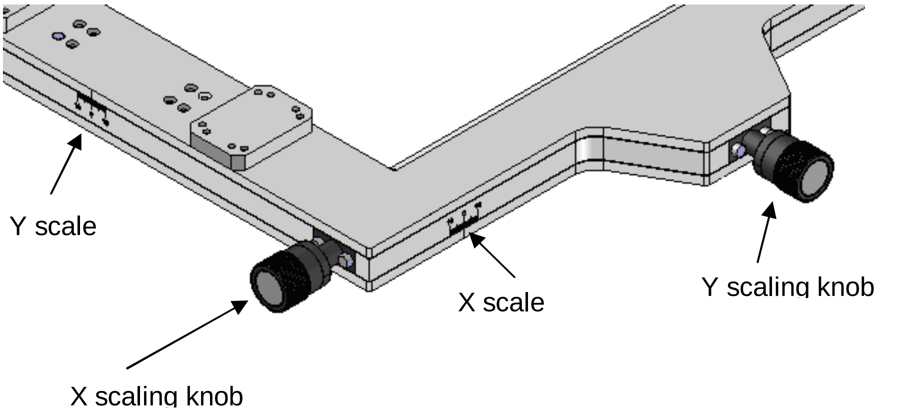

Adjust the upper plate of the XY table by turning the two knobs on the side. The knobs are graded in tenths of a millimeter; one turn is one millimeter. Maximum moving space is ± 10 mm.

There are also two scales, one at each side of the XY table.

7.1.1.3.5. *Mounting of the Gantry Adapters*

Mount the gantry adapter according to the drawings below. Screws, and a 4 mm Allen key are supplied.

Varian Adapter, SDD 100 cm

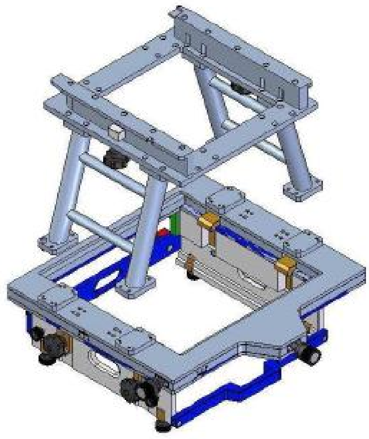	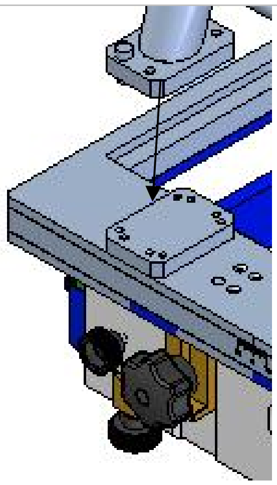
Varian adapter, SDD 100 cm, on the advanced XY-table.	*Detail.*
Fasten the adapter with the supplied screws.	

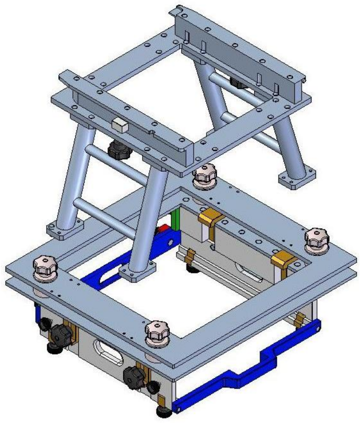	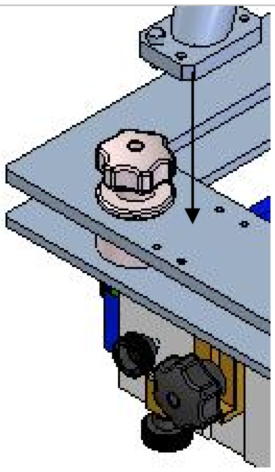
Varian adapter, SDD 100 cm, on the standard XY-table.	*Detail.*
Fasten the adapter with the supplied screws.	

Varian Adapter, SDD 76.2 cm

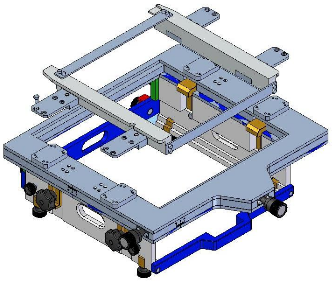	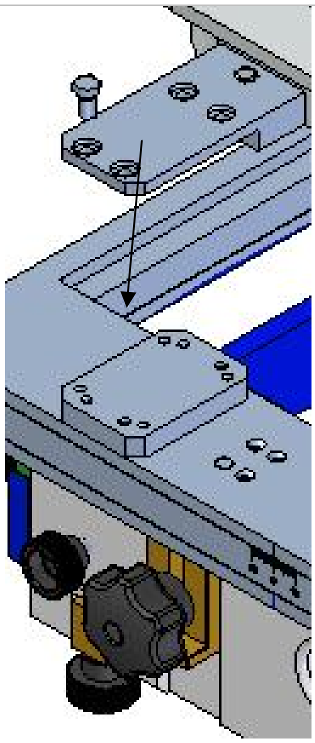
Varian adapter, SDD 76.2 cm, on the advanced XY-table.	*Detail.*
Fasten the adapter with the supplied screws.	

NOTICE

IMPORTANT NOTICE

STANDARD XY TABLE

Varian SDD 76.2 cm adapter can not be mounted on the standard XY-table.

Siemens Adapter, SDD 100 cm

<table>
<tr><td>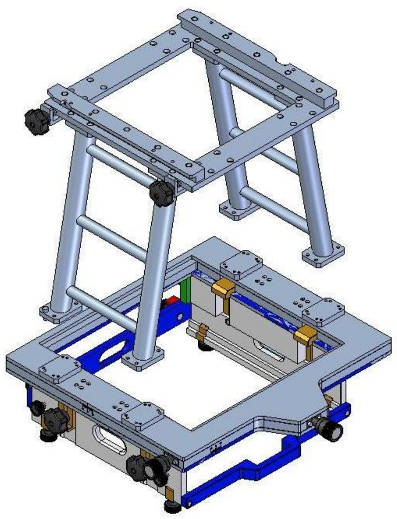</td><td>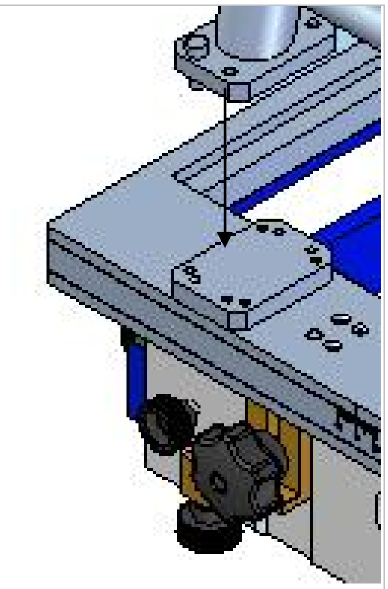</td></tr>
<tr><td>Siemens adapter, SDD 100 cm, on the advanced XY-table.</td><td></td></tr>
<tr><td colspan="2">Fasten the adapter with the supplied screws.</td></tr>
</table>

<table>
<tr><td>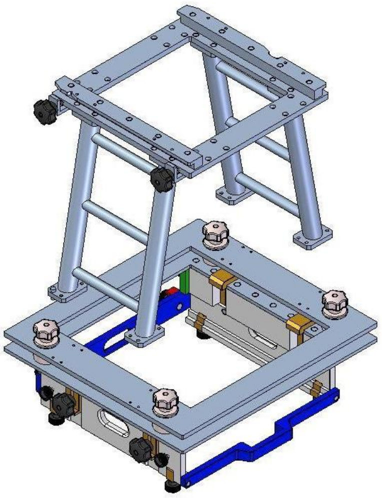</td><td>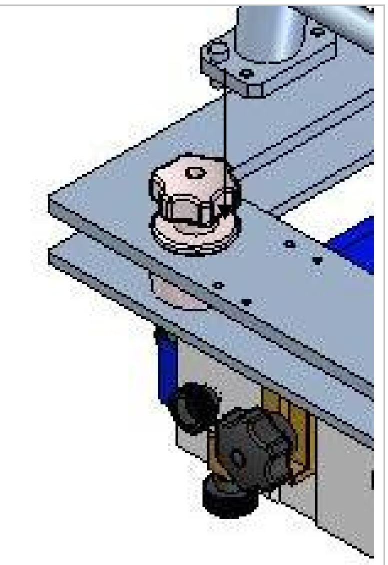</td></tr>
<tr><td>Siemens adapter, SDD 100 cm, on the standard XY-table.</td><td>Detail.</td></tr>
<tr><td colspan="2">Fasten the adapter with the supplied screws.</td></tr>
</table>

Siemens Adapter, SDD 76.2 cm

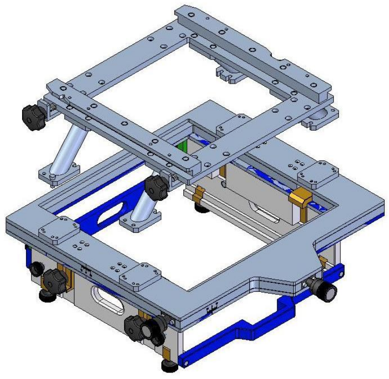	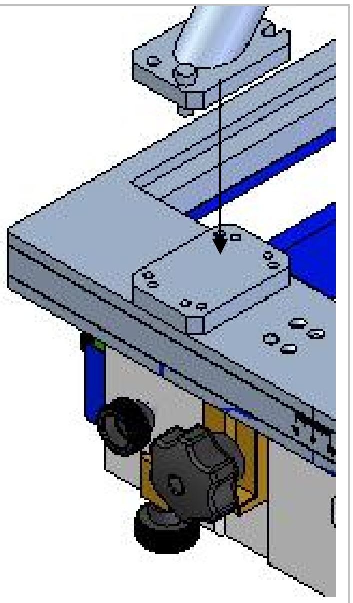
Siemens adapter, SDD 76.2 cm, on the advanced XY-table.	*Detail.*
Fasten the adapter with the supplied screws.	

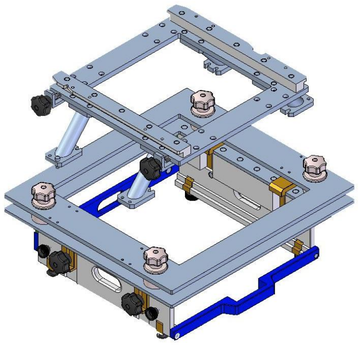	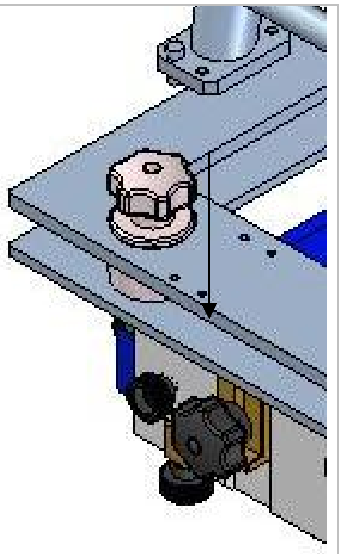
Siemens adapter, SDD 76.2 cm, on the standard XY-table.	*Detail.*
Fasten the adapter with the supplied screws.<br>Start with the inner screws, and insert the screws half-way only (a).<br>Slide the adapter bottom plates against the inner screws (b).<br>Fasten the bottom plates with the outer screws (c).	

Elekta Adapter, SDD 100 cm

<table>
<tr><td>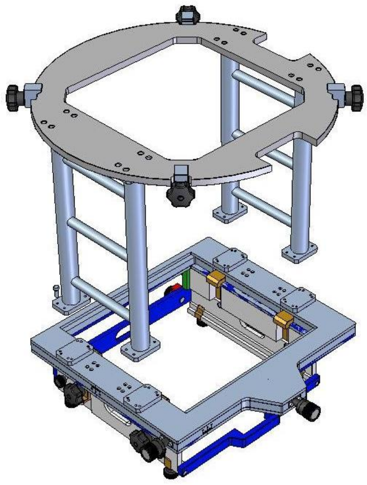</td><td>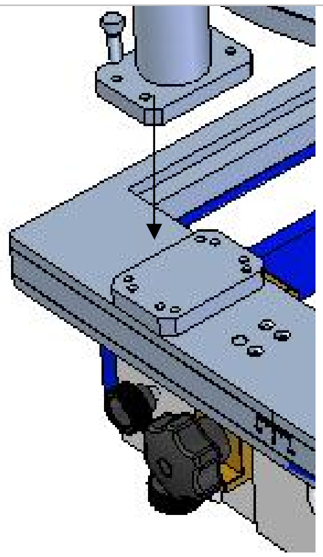</td></tr>
<tr><td>Elekta adapter, SDD 100 cm, on the advanced XY-table.</td><td>Detail.</td></tr>
<tr><td colspan="2">Fasten the adapter with the supplied screws.</td></tr>
</table>

<table>
<tr><td>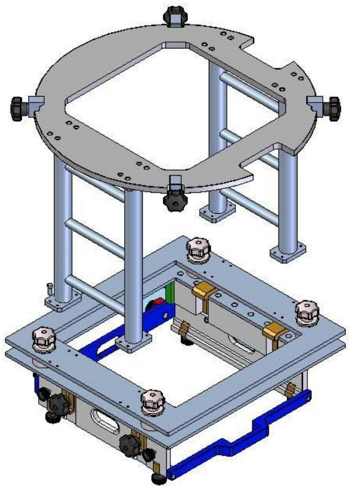</td><td>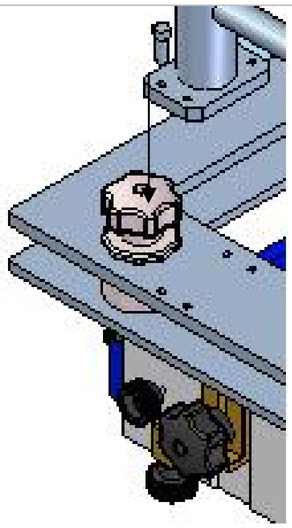</td></tr>
<tr><td>Elekta adapter, SDD 100 cm, on the standard XY-table.</td><td>Detail.</td></tr>
<tr><td colspan="2">Fasten the adapter with the supplied screws.</td></tr>
</table>

Elekta Adapter, SDD 76.2 cm

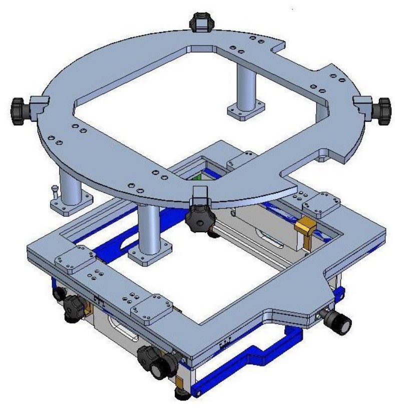	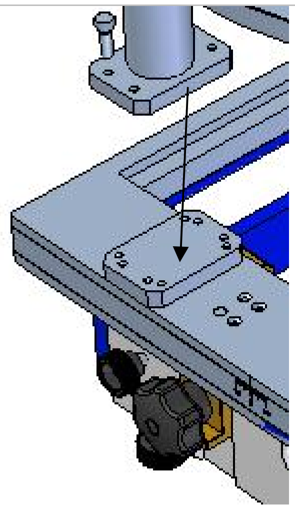
Elekta adapter, SDD 76.2 cm, on the advanced XY-table.	*Detail.*
Fasten the adapter with the supplied screws.	

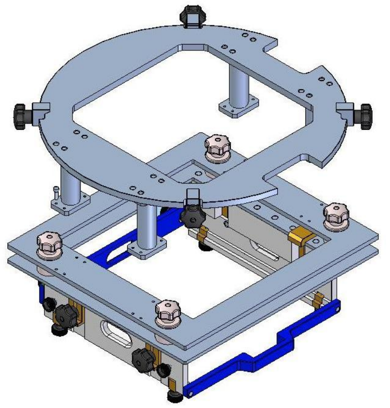	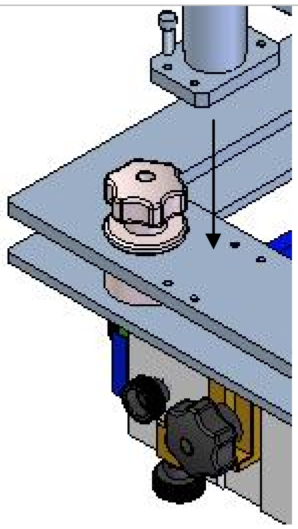
Elekta adapter, SDD 76.2 cm, on the standard XY-table.	*Detail.*
Fasten the adapter with the supplied screws.	

7.2. Setting up MatriXX Evolution

The diagram below shows the general placement of MatriXX Evolution, the power supply, the PC, and the connections between them.

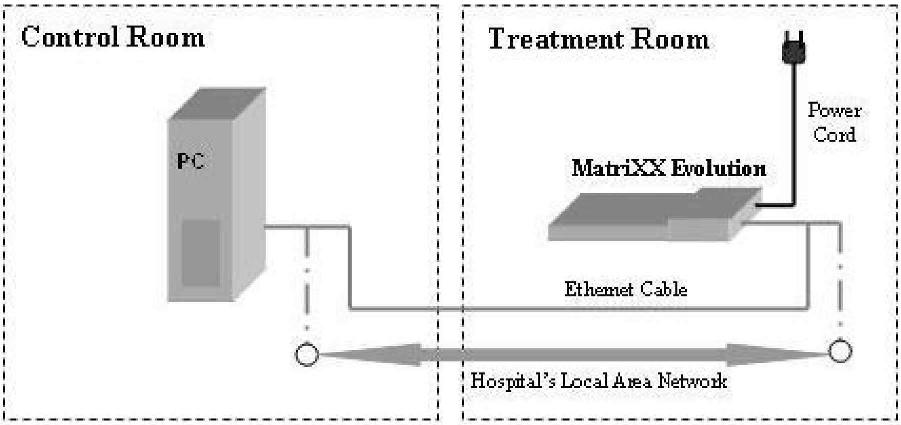

It is connected to the PC in the control room via an Ethernet cable, or via an existing LAN network.

MatriXX Evolution can be positioned on the treatment couch, or mounted in a MULTICube.

7.2.1. Setup with MULTICube

In the MULTICube the device can be positioned in steps of 5 cm vertically, thus raising the measurement plane up to 20 cm above the plane of the couch.

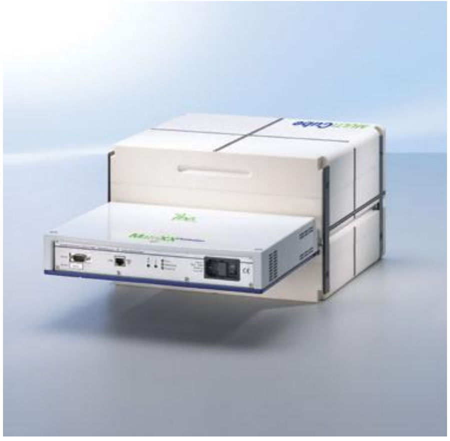

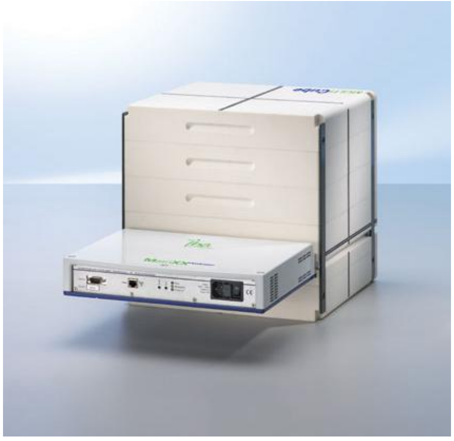

MatriXX Evolution may also be positioned in a vertical position.

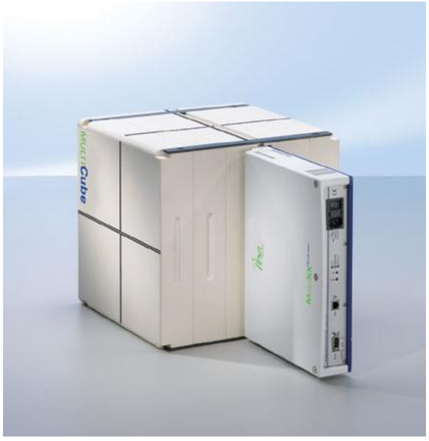

CAUTION

CAUTION

SUPPORT THE ELECTRONICS PART OF THE MATRIXX

When backscatter plates are used, the center of mass is on the electronics side of the detector bed. Support the electronics part of the device to eliminate the risk that the MatriXX will tip over. (Sufficient build-up will offset the weight of the electronics and shielding).

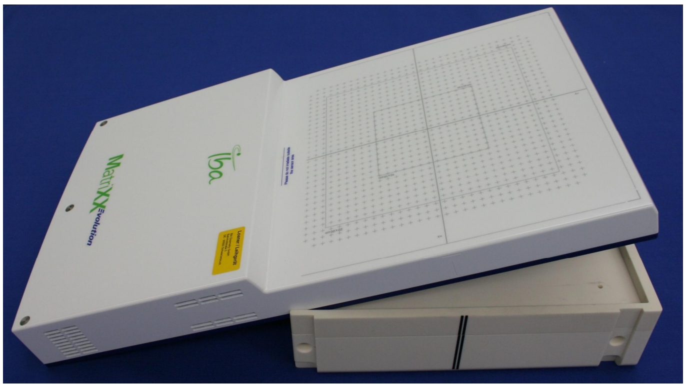

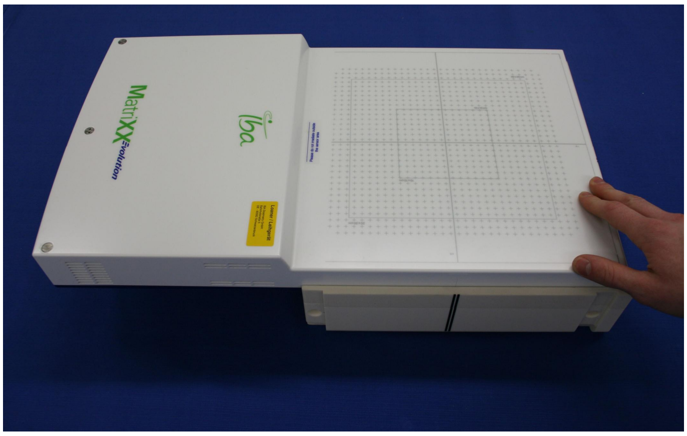

If the MULTICube is not used place at least 5 cm solid water as backscatter and build-up material underneath and on top of the MatriXX.

7.2.2. Setup with Gantry Holder

For details about head mounting and alignment, see 7.1.1.2 *Head Mounted.*

7.3. Preparation for Measurements for I'mRT MatriXX and MatriXX Evolution

7.3.1. Connections

Ensure that the cables to the PC or the network are properly connected.

Plug the power cord into a mains power outlet, and switch on the device. Verify that only the green LED is illuminated.

7.3.2. Pre-irradiation

Like other ion chambers, the MatriXX device needs a certain dose of pre-irradiation before the chamber signal reaches a stable value.

1. Set up the MatriXX under the Linac
2. Start the OmniPro I'mRT software
3. Start the **I'mRT MatriXX** toolbar
4. Select on-line mode
5. Define a sampling time between 500 ms and 1000 ms
6. Deactivate normalization
7. Select maximum field size 27 × 27 cm
8. Turn on the beam (e.g. 6 MV)

The pre-irradiation is completed when the profiles in the profile pane are acceptable, i.e. measured dose values have uniform signal levels, and no deviations are visible.

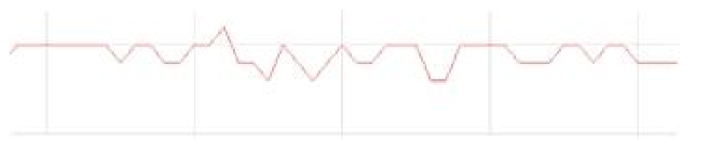	First profile
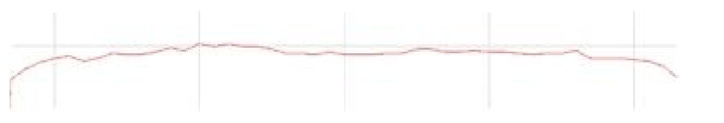	nth profile
	Final profile

Examples of pre-irradiation profiles

Pre-irradiation can be performed during the warm-up phase.

Pre-irradiation has to be repeated every time the device is switched on.

7.3.3. Warm-up Time

For relative dosimetry, the stability of the MatriXX will be sufficient after 15 minutes' warm-up.

For absorbed dose measurements, the stability will be sufficient after 1 hour's warm-up.

7.3.4. Gantry Angle Sensor (available with MatriXX ^{Evolution})

The Gantry Angle Sensor is an additional tool for the MatriXX ^{Evolution}. Its purpose is online detection of the gantry angle while irradiating for treatment verification.

For the Gantry Angle Sensor, the use of MatriXX ^{Evolution} is mandatory, since only the MatriXX ^{Evolution} is intended to be used in rotational applications.

The sensor is equipped with 4 LEDs to facilitate alignment of the sensor in a vertical plane; a precondition for correct measurements (see *7.3.4.2 Mounting and Alignment of the Sensor*).

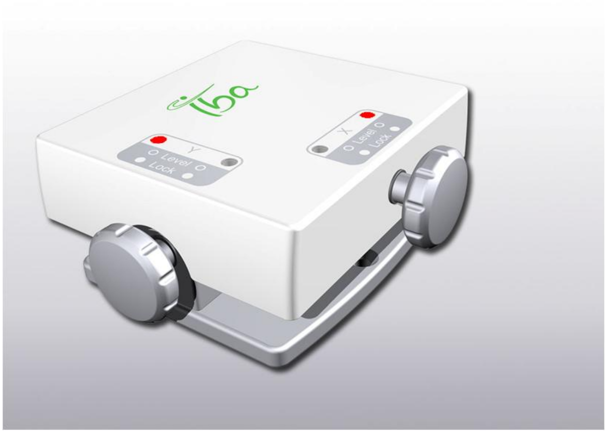

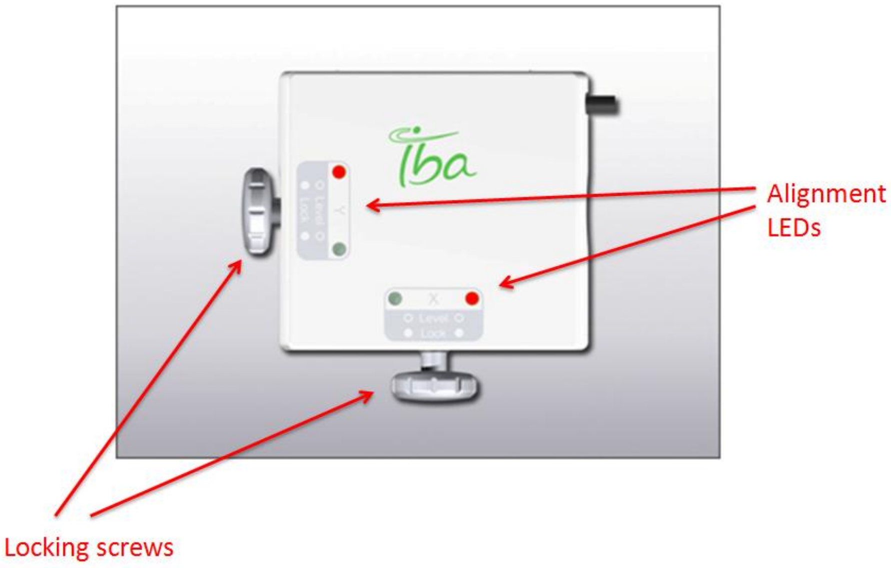

7.3.4.1. Technical Data

Item	Characteristics
Accuracy	± 0.4° during the first year<br>± 0.6° for the remaining lifetime
Temperature range for storage and use	0°C - + 50°C
Cable length	3 m

Connect the sensor to the MatriXX ^{Evolution} Service port.

7.3.4.2. Mounting and Alignment of the Sensor

The sensor shall be fixed on the gantry (e.g. using powerstrips or similar adhesive tape – observe the instructions of use!). In order to provide correct results, the sensor must be aligned in a vertical plane within ± 5°.

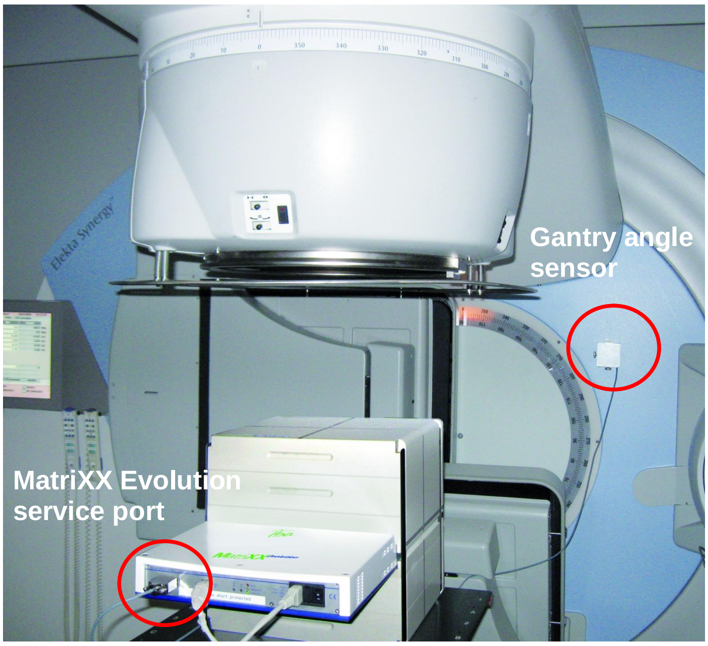

How to align the sensor:

1. Plug in the MatriXX ^{Evolution}.
2. Connect the sensor to the service port of the MatriXX ^{Evolution}.
3. Power on the MatriXX ^{Evolution}.
4. At the sensor, remove the tilt in direction Y.
5. Turn the gantry to 90°.
6. Remove the tilt in direction X.

How to remove the tilt in each direction:

1. Turn the gantry to 0°, loosen the screw for Y direction and align the sensor until no LED for this direction lightens up. Fasten this screw. Repeat this procedure after turning the gantry to 90° for X direction.
2. The LEDs on the sensor will light up if the tilt is not within the permitted range (± 5°) in the respective direction. If no LEDs lighten up anymore, the sensor tilt is within the tolerance for both directions. The sensor can then be calibrated.
3. The correct alignment is checked in the software during calibration of the sensor (see *7.3.4.3 Calibration of the Sensor*).

NOTICE

MPORTANT NOTICE

DISTANCE BETWEEN MATRIXX ^{EVOLUTION} AND GANTRY ANGLE SENSOR

Make sure the distance between MatriXX ^{Evolution} and Gantry Angle Sensor is such that the length of the cable (3 m) is sufficient even when turning the gantry.

NOTICE

IMPORTANT NOTICE

GANTRY ANGLE AND ROTATIONAL DIRECTION

The gantry angle and the rotational direction of the sensor are defined in IEC 61217.

NOTICE

IMPORTANT NOTICE

SENSOR READOUT

The sensor readout can be affected by outside influences (vibration, etc.).

7.3.4.3. Calibration of the Sensor

When the sensor is connected to the MatriXX^{Evolution}, and the OmniPro I'mRT software is started, the software automatically detects the sensor. If a measurement is started without an existing sensor calibration, a warning message appears.

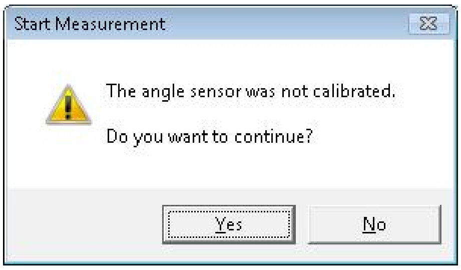

NOTICE

IMPORTANT NOTICE

CALIBRATION OF SENSOR

The sensor calibration must be repeated every time that the MatriXX^{Evolution} is switched on.

1. When the sensor is aligned, go to ***Measure/Calibrate Angle Sensor***. The **Gantry Angle Calibration** window opens.

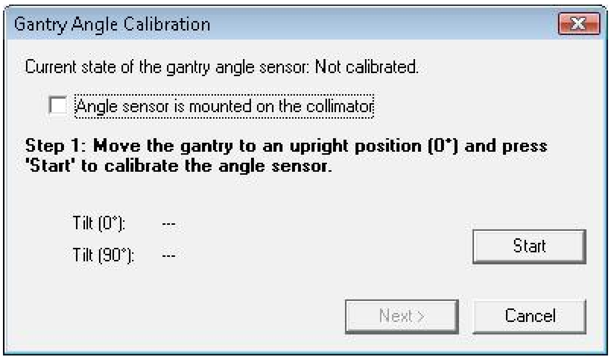

   The checkmark for **Angle sensor is mounted on the collimator** will for now only serve as information for the user. In later versions of the software there will be the possibility to mount the angle sensor at the collimator, and an algorithm will calculate the angle also when the collimator rotates.

   Descriptions of the steps through the calibration process are displayed in the **Gantry Angle Calibration** window.

2. The first step after fixation of the sensor on the gantry is to move the gantry in an upright position (gantry head up) and press the **Start** button. The tilt of the sensor and the gantry angle will be displayed.

   The tolerance to pass the calibration is ± 5°. If the tilt is within this range, a green checkmark is displayed.

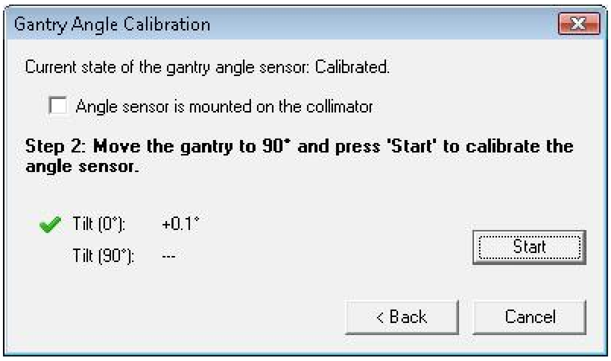

If the tilt is out of range, a red cross is displayed. Re-align the sensor on the gantry, and repeat Step 1.

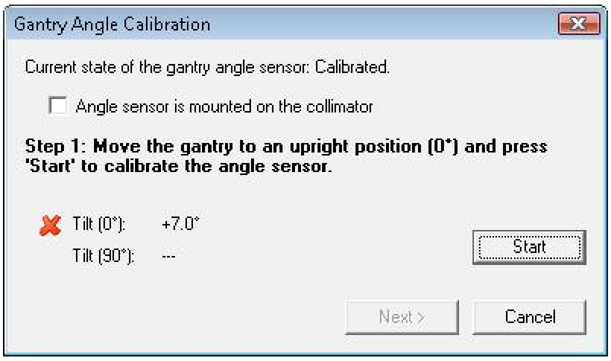

NOTICE

IMPORTANT NOTICE

GANTRY ORIENTATION

Make sure that the gantry is upright in Step 1. Only in step 2 there is plausibility check if the gantry is 90° ± 5° different from Step 1.

3. After passing Step 1, proceed to Step 2. Follow the instruction of Step 2, i.e. rotate the gantry to 90° and press the **Start** button.

   If the gantry was not rotated at all or not to the right position, an error message is popping up requesting to check the reference angle.

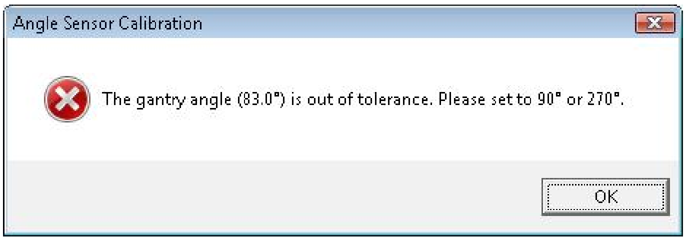

4. The sensor is successfully calibrated when the second step is passed. Close the window.

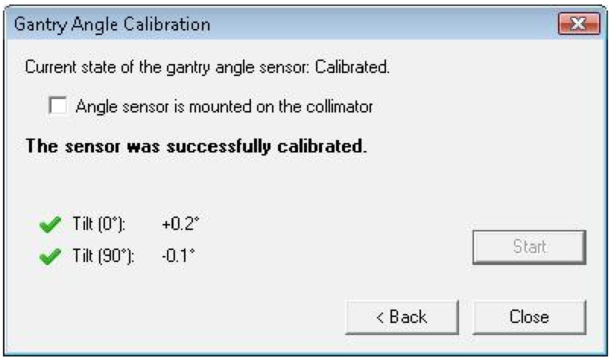

7.3.4.4. Performing Measurements with the Gantry Angle Sensor

Follow the normal procedure for performing measurements as described in chapter *Measurements Using I'mRT MatriXX and MatriXX ^{Evolution}*.

NOTICE

IMPORTANT NOTICE

QA OF ROTATIONAL TREATMENTS

Note that QA of rotational treatments can be performed in **Movie** mode only.

7.3.4.4.1. QA Measurement Procedure for a Rotational Treatment

1. Measure the treatment in **Movie** mode with a sampling time of e.g. 200 ms – 1000 ms (assuming an angular speed of 0.4°/s).
2. Apply the angular correction (see *7.3.4.4.3 Gantry Angle Correction*).
3. Accumulate the corrected images to an integral.
4. Perform the QA in integral of set 4.

After performing a measurement with the gantry angle sensor, the angle will be stored. It is displayed in the dataset pane for each measurement.

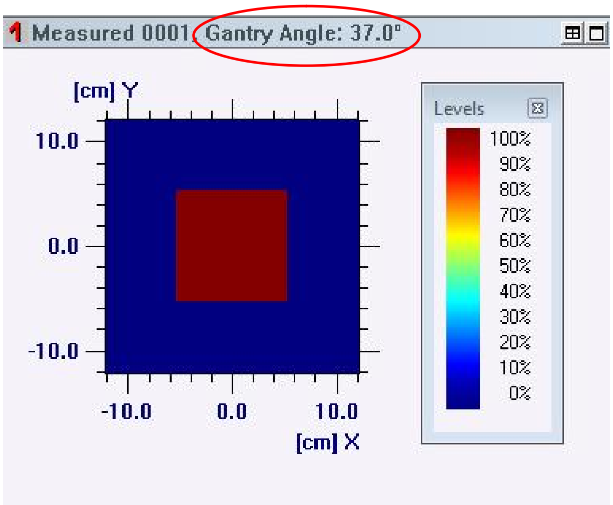

The angle will also be displayed in the field list, for each measurement done with the sensor. For the automatically calculated integral no angle will be displayed.

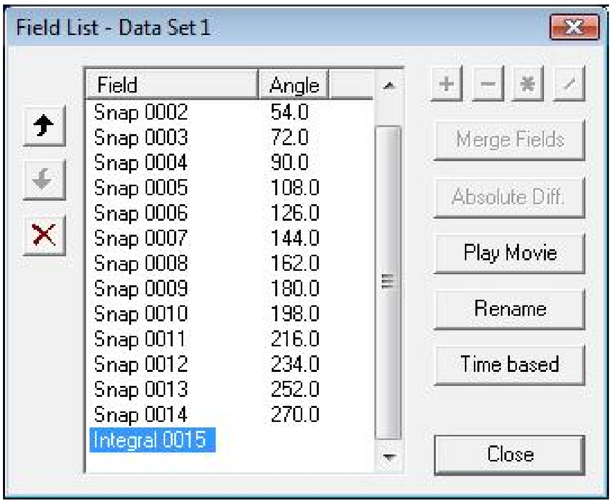

The header information of a measurement done with the gantry angle sensor will contain the gantry angle value.

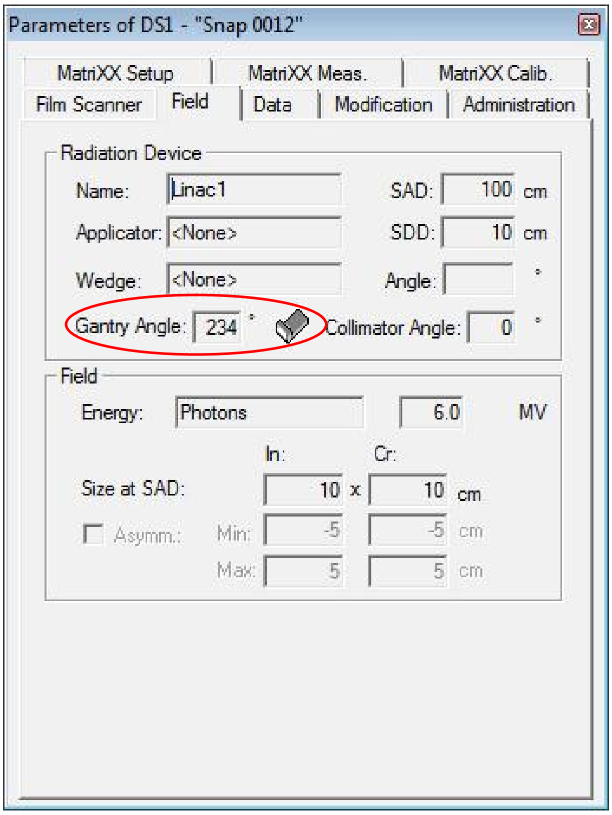

7.3.4.4.2. *Function Check*

To check the function of the sensor, measure two single shots and compare the measured gantry angle with the expected value.

NOTICE

IMPORTANT NOTICE

CHECK FUNCTIONALITY

It is recommended to check the functionality of the sensor once a year.

7.3.4.4.3. *Gantry Angle Correction*

The MatriXX^{Evolution} has a small angular dependence which can be corrected by a calculated matrix. These values are stored in look up tables and can be applied to already measured data dependent from the angle.

NOTICE

IMPORTANT NOTICE

LUT CORRECTION

The values in the look up tables are only valid for the combination of MatriXX^{Evolution} with MULTICube. If the MatriXX^{Evolution} is not used with a MULTICube you can create and use your own angular correction tables (please see below).

NOTICE

IMPORTANT NOTICE

APPLYING ANGULAR CORRECTIONS

Applying angular corrections to measurements is only possible if they were done with the gantry angle sensor.

Settings for Angular Correction

For applying a correction matrix to measurements go to ***Edit/Apply gantry angle correction***.

Linac: In the drop down menu the given correction files are listed. Given by default is a matrix for 6 MV and a beam quality index of 0.666, and a matrix for 18 MV and a beam quality index of 0.783. These are common values for the beam quality index and representative for the 6 MV and 18 MV.

Energy: Select **Set User-Defined** to enable the fields for editing **MV** and **Beam Quality Index** values. Enter energy and a beam quality index.

The **Beam Quality Index** is specified by the tissue phantom ratio TPR20/10. If this value is not available, it can be obtained from the percentage depth dose curve for the given energy:

$$TPR_{20/10} = 1.2661 \cdot PDD_{20/10} - 0.0595$$

where $PDD_{20/10}$ is the ratio of the percent depth dose at 20 cm and 10 cm depths for a field size of 10 cm × 10 cm defined at the phantoms surface with an SDD of 100 cm.

It is possible to select a customized beam quality index by ticking the box **Select Beam Quality Index**. Enter energy and a beam quality index. The software creates via linear interpolation the matrix for the given beam quality index and calculates the angular correction for the measured data. The entered energy is only a header information and does not influence the calculation.

Check the box **Copy the original images** if the original measurements shall be kept. In the field list the originals will be corrected, and a copy of the originals will be created. The integral is still the integration of the originals, even if the originals were replaced by the corrected data.

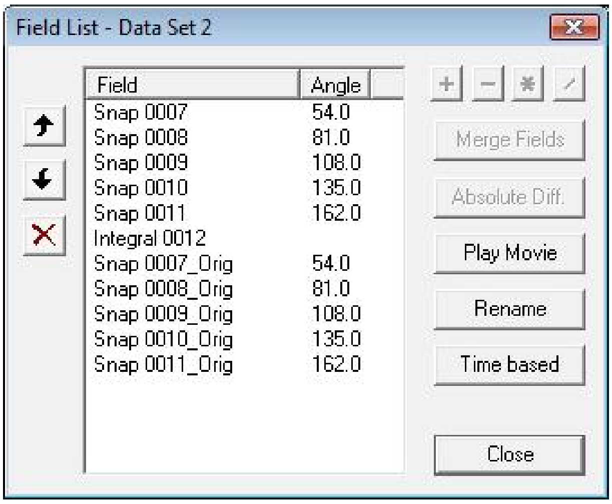

In the field **LUTs** it is possible to delete and export existing look up tables for correction of gantry angles, or to import new tables.

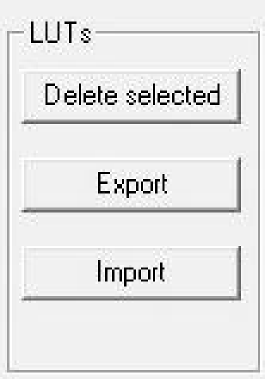

NOTICE

IMPORTANT NOTICE

PATH TO ORIGINAL CSV FILES

If the LUTs were deleted by mistake, the original csv-files are stored under the path: C:\ProgramFiles IBADosimetry OmniPro-ImRT\ SampleData \ AngularCorrection.

To customize existing correction tables, export the LUT by pressing **Export**.

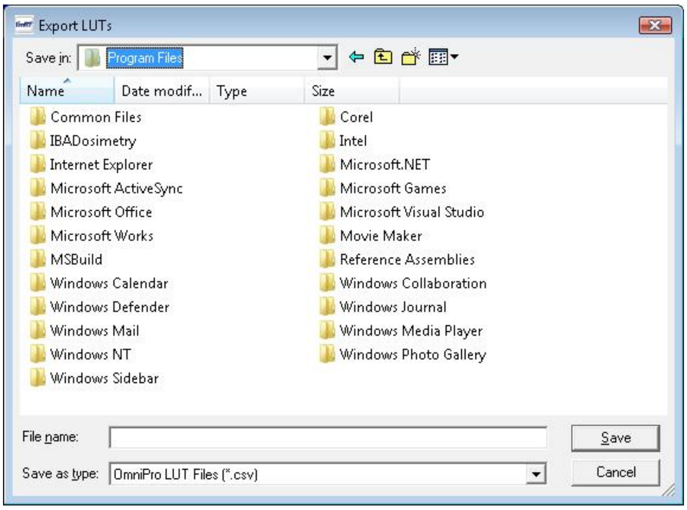

Save the csv-file under an individual name and open it with e.g. Microsoft Office Excel. To do this, in the Windows settings the decimal point must be "." and the list separator must be ",". Only csv-files can be imported.

In a similar way it is possible to customize the values as required (also without the MULTICube).

Create a customized angular correction factor tables

It is possible to create a customized set of correction factor tables from MatriXX measurement and reference dose distributions. By doing so, it is possible to adjust the angular correction of MatriXX measurement to individual reference, e.g. heterogeneous or homogeneous TPS dose distributions or film measurements.

For the measurement of angular correction factors it is recommended to use a set of static fields with incident angles between 0° and 180° with an angular resolution of at least 10° for each photon beam energy (OmniPro ImRT 1.7 assumes symmetry between the angle range [0° - 180°] and [180° - 360°] so for example the correction factor for gantry angle 90° is used for gantry angle 270° as well). Correction factor tables are stored in a simple “comma separated value” (csv) file with following format:

```
LINAC_NAME, NOMINAL_BEAM_ENERGY, TPR20_10, NUM_ROWS, NUM_COLUMNS
GANTRY_ANGLE_1
CF_1, CF_2, ... [NUM_COLUMNS times]
.
. [NUM_ROWS times]
GANTRY_ANGLE_2
CF_1, CF_2, ... [NUM_COLUMNS times]
.
. [NUM_ROWS times]
.
. [for all gantry angles]
[begin next LINAC_NAME entry here]
```

NOTICE

IMPORTANT NOTICE

MULTIPLE CORRECTION TABLES

One csv file can contain multiple correction tables, e.g. correction factor tables for different linacs or different beam energies.

Save the customized correction factor table and import the LUT by pressing **Import** under ***Edit/Apply gantry angle correction***.

Correction of Measurements

1. Copy all data which shall be angular corrected to a field list, or choose the last done measurement.
2. Go to ***Edit/Apply gantry angle correction*** and select one of the listed correction files, and enter/mark the relevant options.
3. Press **OK** to apply the correction matrix to all measurements in the field list.

Example:

Below is an example of a measurement with 10 × 10 cm field size at a gantry angle of 90° and measured with 6 MV photons:

In **Dataset 1** is the original measurement is displayed. **Dataset 2** displays the corrected measurement, for which a beam quality index of 0.67 was used.

When using the plan verification 2D tool and comparing the profiles, the result of the correction is displayed:

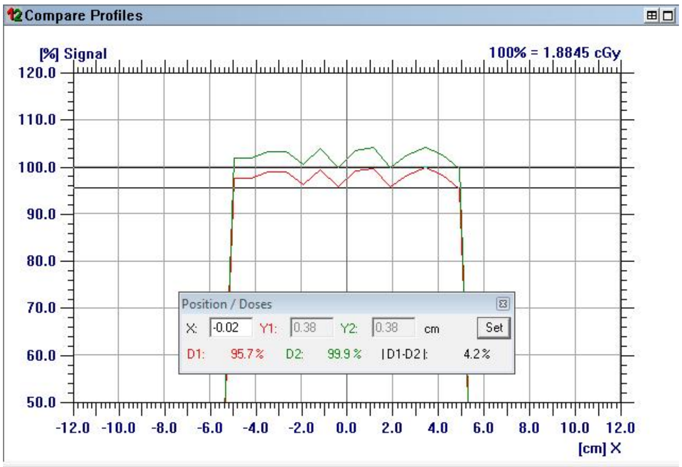

The difference between the original and corrected data is displayed. The absolute measured values for 90° are approx. 4% lower than they shall be (red line). The corrected data (green line) adjust the measured points upwards by 4%.

The angle correction is stored and displayed in the **Parameters** window. Select ***View/Parameters*** and choose the tab **Modifications**. The **Linac** type, and the saved values for the **Energy** and **Beam Quality Index** are displayed.

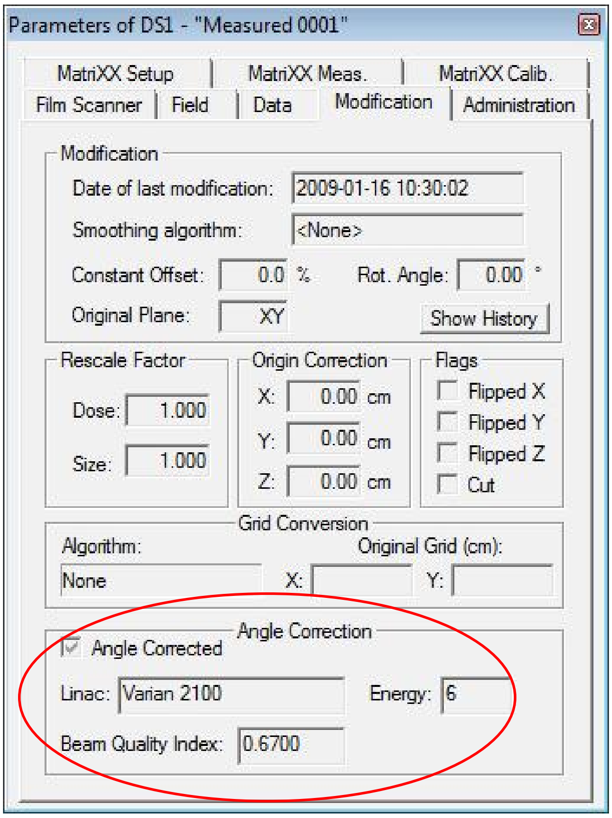

7.4. I'mRT MatriXX and MatriXX ^{Evolution} Measurements

7.4.1. General

With MatriXX, 2D intensity maps can be measured with a time resolution down to 20 ms.

Afterwards each shot, or the integrated signal, can be analyzed.

7.4.2. Calibration

7.4.2.1. Calibration Equation

Calibration is performed according to the equation below:

$D_{ij} = (M-B)_{ij} \cdot N_{DW}(60Co) \cdot K_{uni\ ij} \cdot K_{off\ i,j} \cdot k_{pT} \cdot k_{User}$

where

D_{ij}	is the calibrated dose
M_{ij}	is the matrix of measured values
B_{ij}	is the matrix of background values
$N_{DW}(60Co) \cdot K_{uni\ ij}$	is the Uniformity Correction Matrix, i.e. the uniformity calibration matrix, multiplied with calibration to cobalt in Gy/nC at reference temperature and pressure
k_{User}	is the factor for the detector, obtained by the value of a dose rate or dose measurement in a reference chamber, divided with the average of the values of four middle chambers in the device
$K_{off\ i,j}$	is the additional off axis calibration (user calibration)
k_{pT}	is the correction for pressure (p) and temperature (T): (273.15° K + T) / (273.15° K) · (1013.3 hPa / p)

M_{ij} and **B_{ij}** are obtained from the MatriXX measurement.

N_{DW}(60Co) · $K_{uni\ ij}$ is measured and calculated at the production site.

The **k_{User}** factor can be created by the user (see *Create the k_{User} factor*).

$K_{off\ i,j}$ can be created by the user (see *User-defined off-axis calibration*).

k_{pT} is measured and calculated by the MatriXX system. The pressure factor can be corrected with the offset obtained when comparing the p value as measured by the system and by a reference meter (see 7.4.4.2 *User-defined offset for the k_{pT} factor*).

7.4.3. Uniformity Correction Matrix for MatriXX

NOTICE

IMPORTANT NOTICE

UNIFORMITY CORRECTION MATRIX

The uniformity correction matrix is stored in the device itself, and shall be applied in all measurements.

⚠ WARNING

WARNING

UNIFORMITY CORRECTION MATRIXX FOR MATRIXX

The application of the Uniformity Correction Matrix file is mandatory. If the checkbox **Activate device-internal calibration data** is unmarked, the measurement will be performed without correction.

The uniformity correction matrix is individual for each MatriXX device, and will be used to remove inhomogeneities from the measured image. Data are stored in the device.

1. Select **Correction** in the **I'mRT MatriXX** toolbar.

   The Correction/Calibration dialog opens.

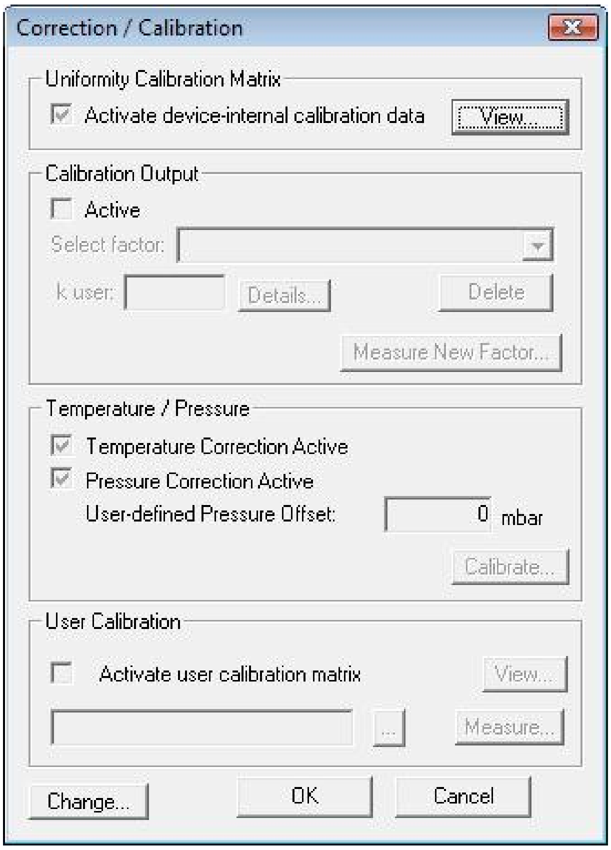

2. Click **Change**. A password dialog opens.

3. Enter the password. The default password is “user”.
4. You have the possibility to change password: Click **Change** in the password dialog. The **New Password** dialog opens.

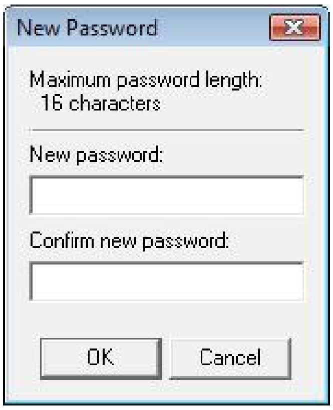

5. Enter and confirm your new password. Click **OK**.

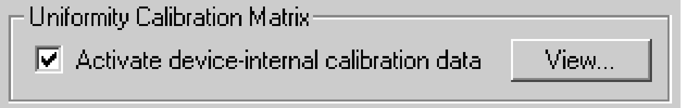

6. Mark the **Activate device-internal data** checkbox. To view data, click the **View** button.

7.4.4. Add Correction Factors

A correction factor for the detector (k_{User}), and of the measured pressure and temperature (k_{pT}), can be added by the user.

The k_{User} factor will be specific for the selected Linac, energy type, and energy value. Several factors, for different Linacs and energies, can be created.

7.4.4.1. Creating a k_{User} Factor for Calibration Output

Create a reference value, by performing a measurement with a reference chamber, e.g. a Farmer chamber. The measurement can be done in dose or dose rate mode.

1. Select ***I'mRT MatriXX*** in ***Equipment:Select Clinic and Device***. Click **Background** in the **MatriXX** toolbar to perform a background measurement.
2. Click **Correction** in the **MatriXX** toolbar.
3. The **Correction/Calibration** dialog opens.
4. Click **Change**, and enter the password. The default password is "user". To change the password, click **Change** in the password dialog, and enter the new password.
5. Click the button **Measure New Factor**.

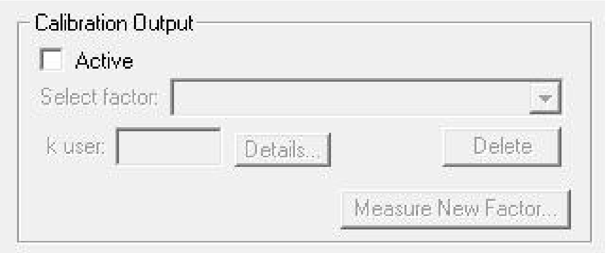

A wizard that guides you through the process will open.

7.4.4.1.1. Dose rate mode

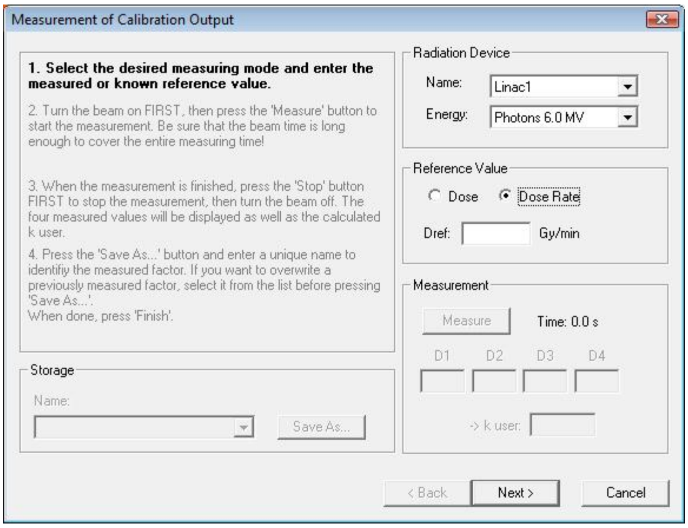

1. Select radiation device, and define the energy.
2. Select **Dose Rate**, and enter the reference value.
3. Click **Next**.

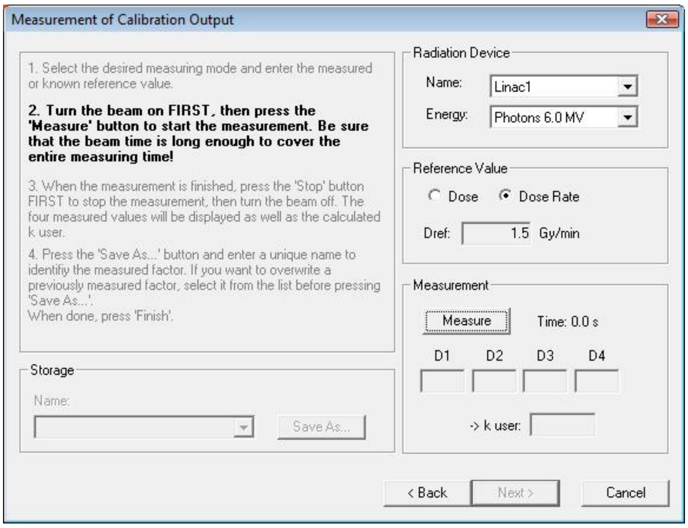

4. Turn on the beam, and click the **Measure** button to start the measurement. Ensure that the beam time is long enough to cover the entire measuring time.

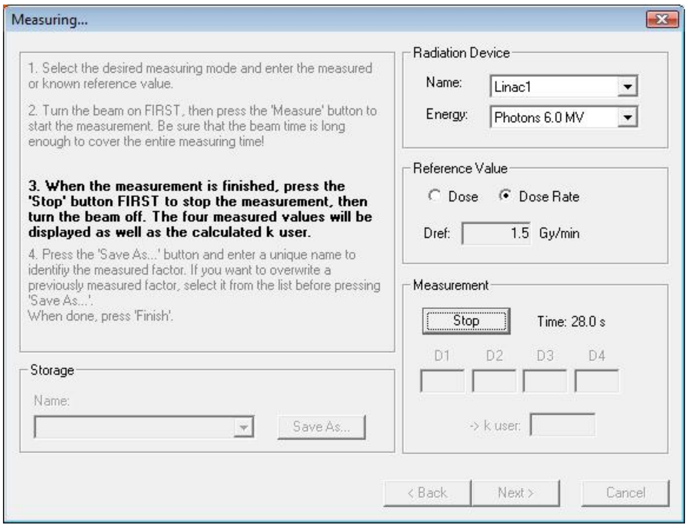

5. Stop the measurement with the **Stop** button, and turn off the beam.

   The system will calculate k_{User}, using the entered reference value and the average of the values of four middle MatriXX chambers. The new factor will be displayed in the k_{User} field.

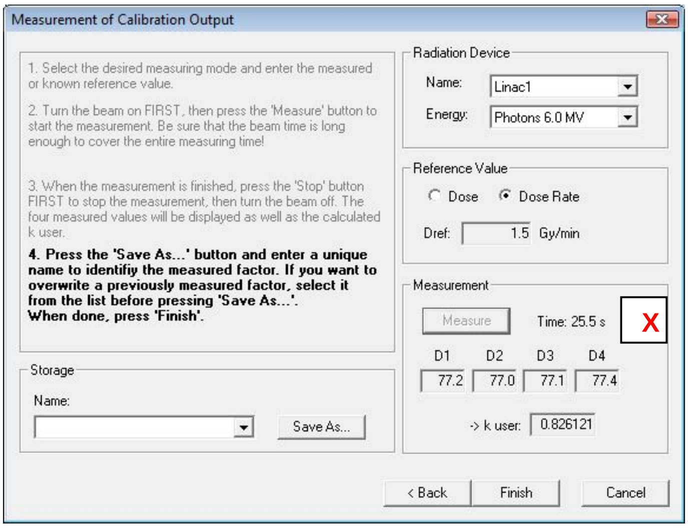

6. Click **Save As** to open the dialog **Save Calibration Factor**.

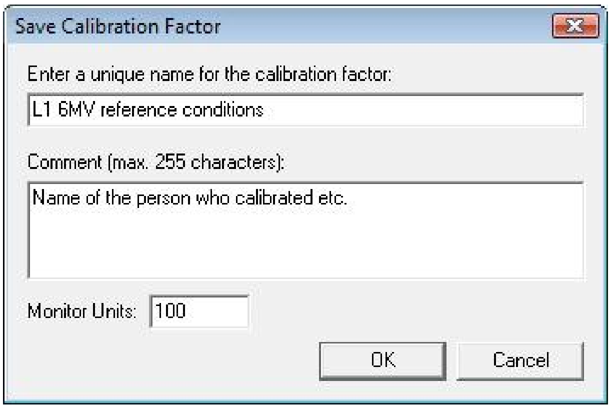

7. Enter a unique name, and (optionally) a comment in the **Comment** field. Click **OK** to save data and close the dialog. To overwrite the value for a previously calculated factor, select the name and click **OK**.

The new factor is persistent between sessions, and will be used until another factor is created.

If the **Active** checkbox in the **Correction/Calibration** dialog is not marked, the factor will be set as 1.

7.4.4.1.2. Dose Mode

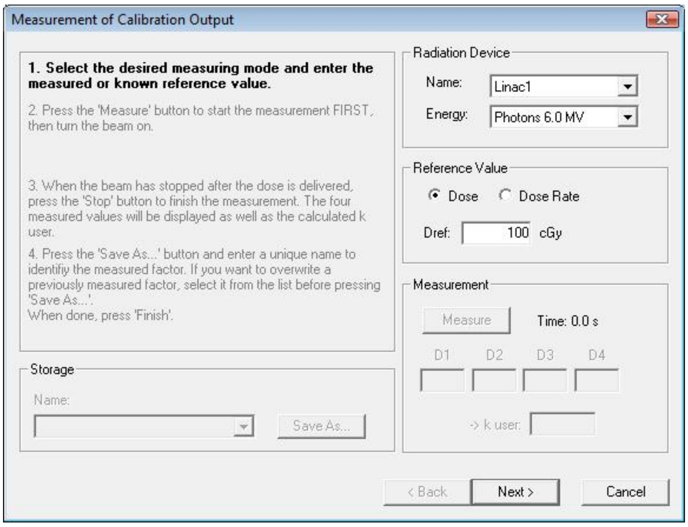

1. Select **Dose** and enter the reference value. The value will be displayed as **mGy**, **cGy**, or **Gy**, depending on the settings in **Options**.
2. Click **Next** to open the next wizard window.

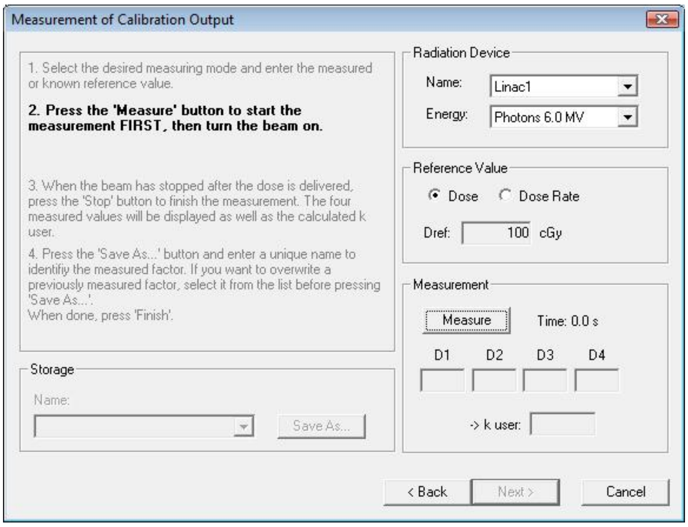

3. Click the **Measure** button to start the measurement. The beam shall be turned on after the measurement has been started. Ensure that the beam time is long enough to cover the entire measuring time.
4. When the defined measurement time has elapsed, turn off the beam, and click the **Stop** button to stop the measurement.

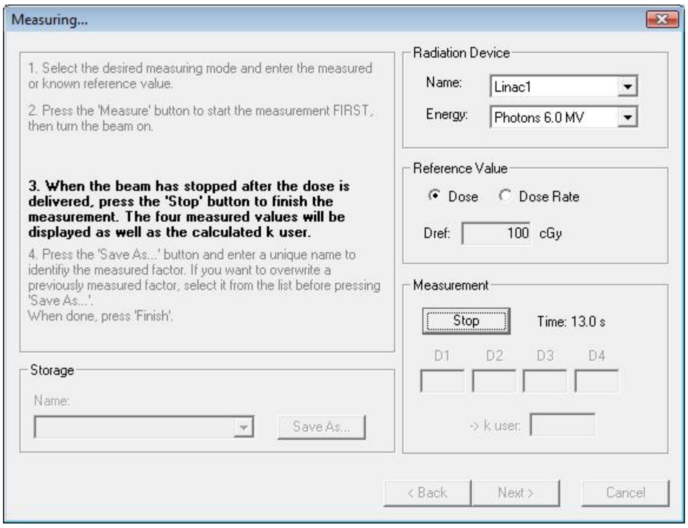

The system will calculate k_{User}, using the entered reference value and the average of the values of four central MatriXX chambers. The new factor will be displayed in the k_{User} field.

5. Click **Save As** to open the dialog **Save Calibration Factor**.

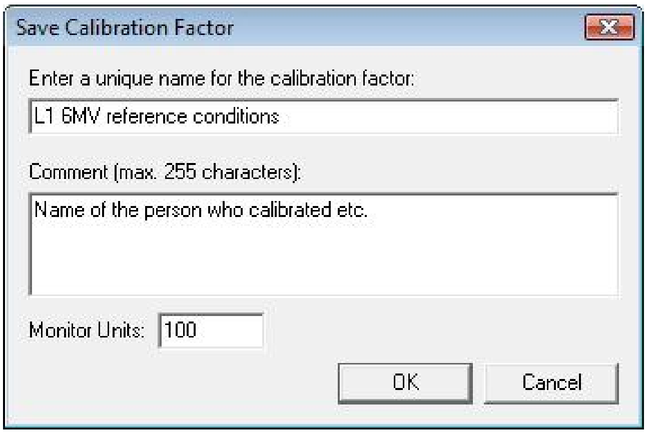

6. Enter a unique name. Optionally a comment in the **Comment** field can be added, and the number of monitor units can be edited. Click **OK** to save data and close the dialog. To overwrite the value for a previously calculated factor, select the name and click **OK**.

The new factor is persistent between sessions, and will be used until another factor is created.

If the **Active** checkbox in the **Correction/Calibration** dialog is not marked, the factor will be set as 1.

7.4.4.1.3. *Selecting the K_{user} Factor for a Measurement*

Open the **Correction/Calibration** dialog from the **MatriXX** toolbar.

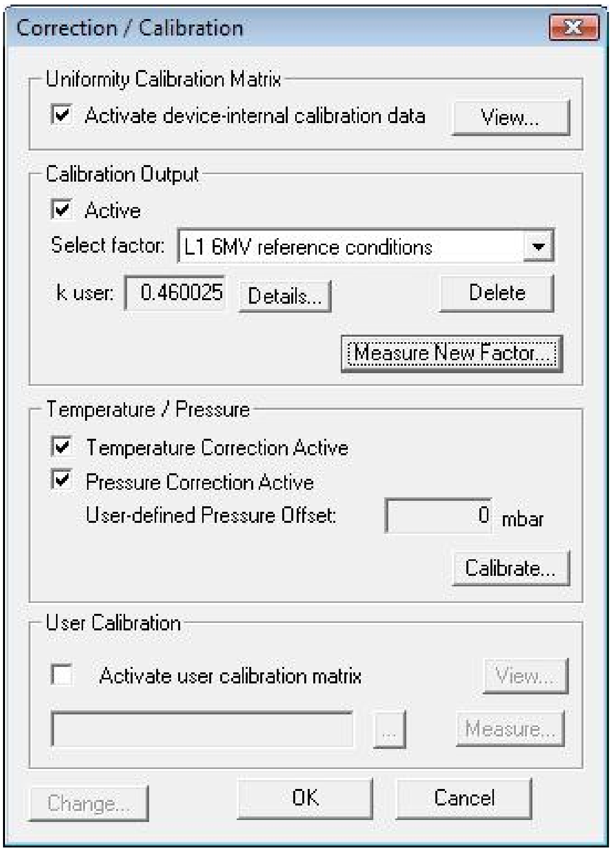

Expand the drop-down list in **Select factor**, and select a factor.

If the selected factor does not fit with the currently selected measurement parameters, a warning will be displayed in the MatriXX toolbar:

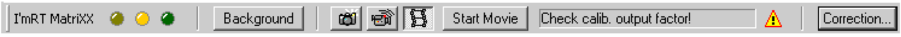

To view data for the selected factor, click **Details**. The following information is displayed:

- Radiation device
- Energy type
- Energy value
- Reference value
- Measured values of the four center diodes
- Reference mode (dose or dose rate)
- Measurement time
- Date and time of the calibration measurement
- Comment entered by the user
- Number of monitor units

The K_{User} factor for stored measurements is displayed in **Parameters**. Open the file with the stored measurement, and select **View/Parameters/MatriXX Calib** tab.

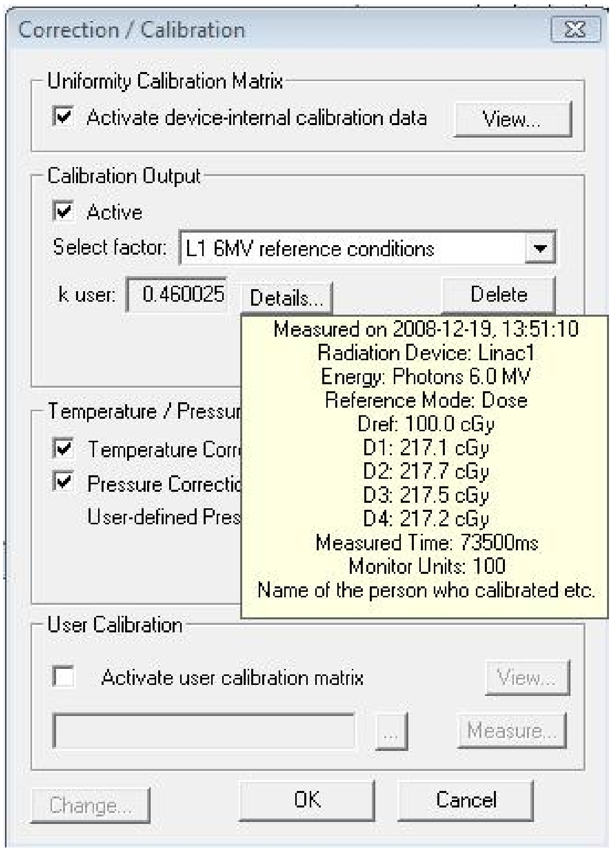

7.4.4.2. User-defined Offset for the kpT Factor

The Temperature and Pressure Correction (the k_{pT} factor) is default based on temperature and pressure as measured by the device. For pressure correction, the offset between measured values and values obtained by a reference meter can be added to the calibration equation.

1. Click **Change** in the **Correction/Calibration** dialog.
2. Enter the password. The default password is "user". To change the password, click **Change...** in the password dialog, and enter the new password.
3. Mark the checkbox **Pressure Correction active**.
4. Click the **Calibrate** button.
5. Measure the pressure with reference meters.
6. Enter the measured value in the **Actual** field.
7. Click the **OK** button.

The difference between the value measured by the device and the entered reference value is displayed in the **User-defined Pressure Offset** field, in the **Correction/Calibration** dialog.

The defined offset is persistent between sessions, and will be used in all measurements until another offset is defined.

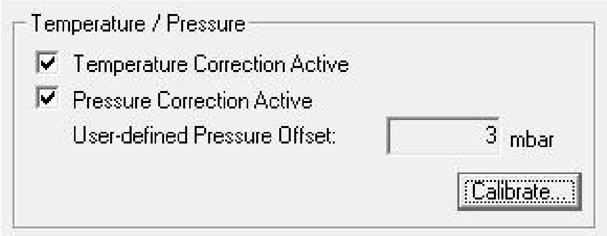

If the **Active** checkbox in the **Correction/Calibration** dialog is not marked, the correction will not be used.

7.4.4.3. User-defined Off-axis Calibration

A user-defined calibration can be applied, to compensate for deviations that occur after the I'mRT MatriXX or MatriXX ^{Evolution} have been in use for some time. The calibration shall be applied as an add-on calibration.

The user calibration makes use of only three full-field measurements in order to calculate user calibration factors for 1020 pixels. Therefore inaccuracies and noise during the user calibration measurement will propagate from one pixel to the other over the full active area of the pixel array, adding a small amount of noise to the calibration data. Therefore it is recommended applying the user calibration only in cases where pixel deviations from uniformity are well above 1%.

The user calibration procedure has been proven to work well for typical cases of miscalibrated pixels, e.g. single or few pixels randomly distributed in the pixel array with arbitrary amount of miscalibration, or Gaussian noise on the entire active area. There might be rare cases however, when the user calibration leads to unsatisfying results. This can happen when miscalibrated pixels are unfavorably distributed in the pixel array, and one miscalibrated pixel is mapped to another one during the user calibration measurement (e.g. many adjacent miscalibrated pixels in one pixel column). In these cases we recommend to recalibrate the device in the factory.

It is recommended to use the gantry holder for the calibration.

7.4.4.3.1. Create the $K_{off\ i,j}$ Factor

1. Click the **Correction** button in the **MatriXX** toolbar. Click **Change**, and enter your password.
2. Unmark **Active** in **Calibration Output**.
3. Mark **Activate user calibration matrix** and click **Measure**.

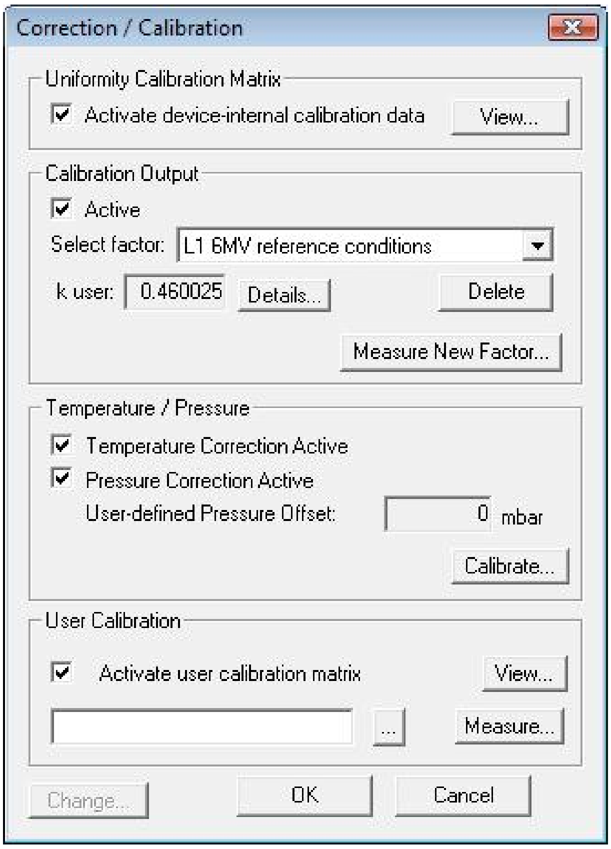

The recommended measurement time for each step in the calibration procedure is 100 MU. The recommended field size is 28 × 28 cm. All channels have to be covered, but the electronics must not be in the beam.

WARNING

WARNING

DEVICE-INTERNAL CALIBRATION DATA MUST BE ACTIVATED

Ensure that the check-box **Activate device-internal calibration data** is marked, before performing the measurements for the $K_{off\ i,j}$ factor.

A series of dialogs will guide you through the procedure.

First measurement

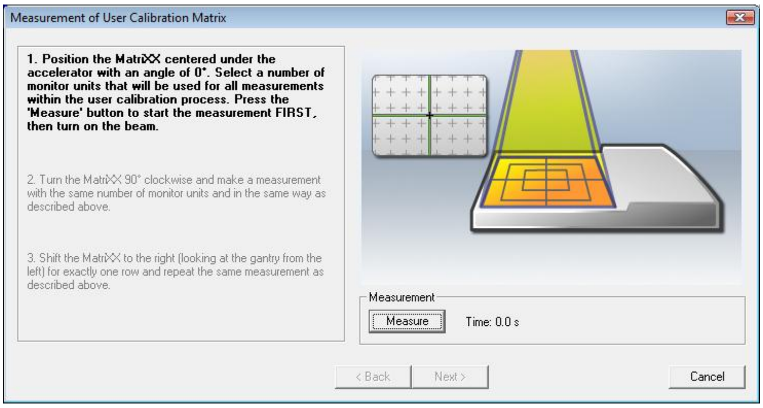

Position the MatriXX centered under the accelerator with an angle of 0 degrees. Select the number of monitor units that will be used for all measurements within the calibration process. Press the **Measurement** button to start the measurement first, and then turn on the beam.

Second measurement

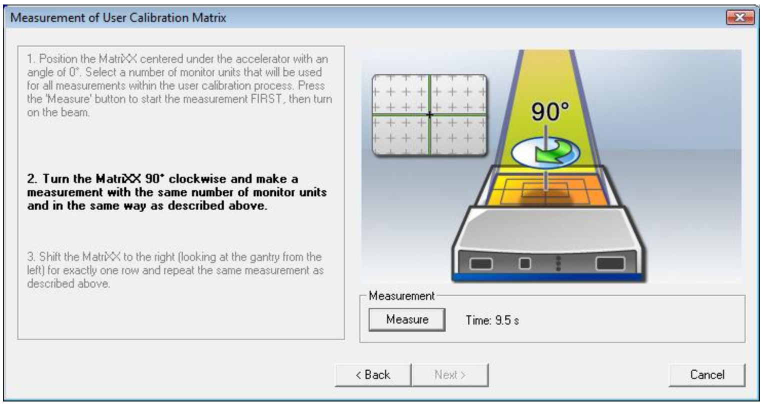

Turn the MatriXX 90 degrees clockwise, and make a measurement with the same number of monitor units, and in the same way as described in *First measurement*.

Third measurement

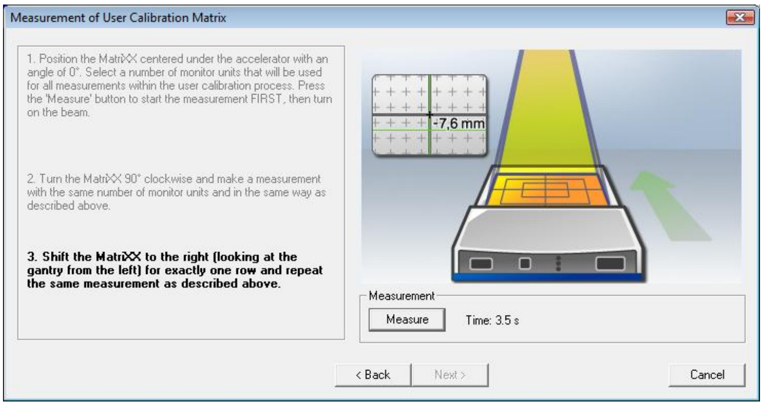

Shift the MatriXX exactly one row of detectors in the right hand direction (looking at the gantry from the front), and repeat the measurement as in *First* and *Second measurement*.

NOTICE

IMPORTANT NOTICE

ADDITIONAL MARKERS

On both sides of the MatriXX there are two smaller additional markers for aligning the MatriXX after shifting one pixel row to the right site.

Click **Finish**, and save the calibration in **Save MatriXX User Calibration**.

Click **View** to open the calibration file. Verify the result.

7.4.4.3.2. Apply the User-defined Calibration

Click the **Correction** button in the toolbar. Mark **Activate device-internal calibration data** and **Activate user calibration matrix**.

⚠ WARNING

WARNING

DEVICE-INTERNAL CALIBRATION DATA MUST BE ACTIVATED

Ensure that the check-box **Activate device-internal calibration data** is marked, when applying the $K_{off\ i,j}$ factor.

Click **OK**.

7.4.5. Measurement Setup

7.4.5.1. Pre-requisites

Before starting measurement with I'mRT MatriXX or MatriXX Evolution, the following is required:

- The Uniformity Correction Matrix is applied. *See 7.4.3 Uniformity Correction Matrix for MatriXX*.
- I'mRT MatriXX or MatriXX Evolution software setup is completed. See *Configuration of OmniPro I'mRT Software.*
- I'mRT MatriXX hardware setup is completed. See the chapters *7 Setup of MatriXX, 7.2 Setting up MatriXX Evolution,* and *7.3.4 Gantry Angle Sensor.*

7.4.5.2. Define the Measurement Parameters

Select ***Equipment:Select Clinic and Device***. Select the appropriate clinic and ***I'mRT MatriXX*** or ***MatriXX Evolution***. The **I'mRT MatriXX** toolbar will be expanded.

Select ***Measure:Edit Parameters***. The **Measurement Parameters** dialog opens.

7.4.5.2.1. MatriXX Setup Tab

Open the **MatriXX Setup** tab.

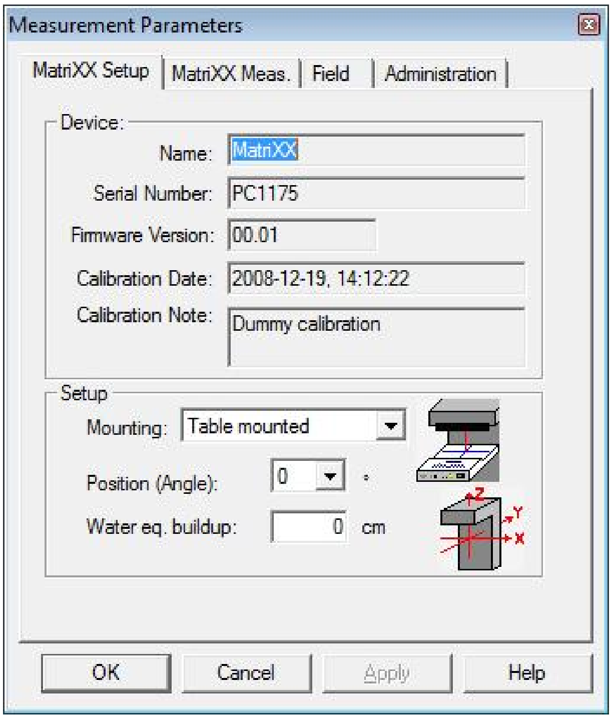

Define how the device is mounted in **Mounting**. The options **Table mounted** and **Head mounted** are available.

Define the position in relation to the accelerator in **Position**.

Device data are found in the **Device** section in the dialog.

7.4.5.2.2. MatriXX Meas. Tab

1. Open the **MatriXX Meas**. tab.

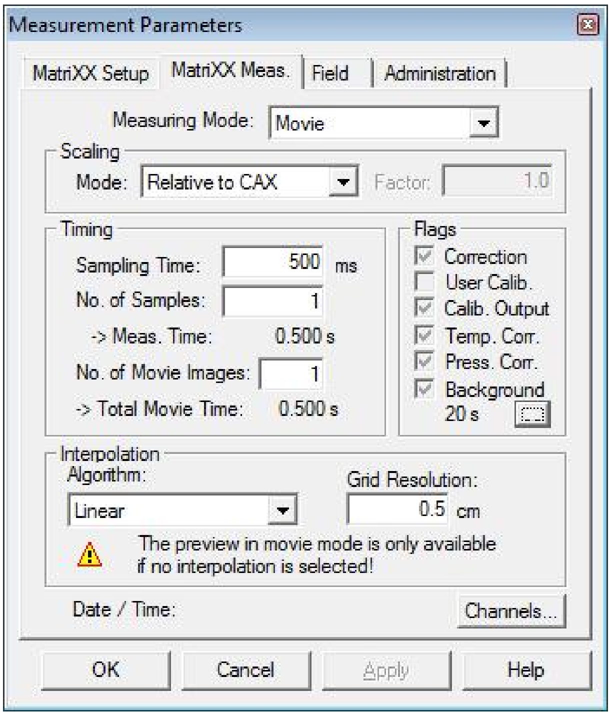

2. Select the type of measurement in **Measurement Mode**.

   Single Shot Mode

   In this mode, one single image is composed of a set of single frames (each one measured with the selected measuring time). The composed image contains the average of the selected number of samples.

   If background compensation is activated, the background is subtracted from each individual frame.

   Movie Mode

   In this mode, a given number of movie pictures (single shots, as described above) are acquired. Each picture, as well as one integrated image, are saved.

NOTICE

IMPORTANT NOTICE

NUMBER OF MOVIE IMAGES

If the No. of Movie Images is set to “0” the movie will not stop until the Stop Movie button is pressed.
Firmware version 1.34 is required to use this option. Therefore please refer to chapter *5.2.4 MatriXX Firmware*

   On-line Mode

   In this mode, single shots, as described above, are measured continuously and displayed on-line. Only the last five images are saved.

   (See also *7.4.6.1 Select measurement mode*).

3. Select scaling mode in the **Mode** drop down list:

   Relative to Maximum: All data is normalized to the maximum value which in turn is set to 100%.

NOTICE

IMPORTANT NOTICE

RELATIVE TO MAXIMUM

It is not recommended to use Relative to Maximum during acquisition, as the original scaling cannot be retrieved later. It also may give the wrong impression that it looks like dose is building up without any beam present since the background will be scaled.

Relative to CAX: All data is normalized to the central axis value (4 central pixels) which set to 100%.

NOTICE

IMPORTANT NOTICE

RELATIVE TO CAX

It is not recommended to use Relative to CAX during acquisition, as the original scaling cannot be retrieved later. It also may give the wrong impression that it looks like dose is building up without any beam present since the background will be scaled.

Fix factor: 1 count on TERA corresponds to 0.1% relative signal, multiplied with the fix factor.

4. In the **Timing** section, enter the **Sampling Time** (i.e. the integration time for one measurement) to be used, and the number of samples and movie images to be used. (See also *7.4.6.1 Select measurement mode*).

NOTICE

IMPORTANT NOTICE

SAMPLING TIME

The sampling time must be entered in multiples of 10.

Single Snap: The measurement time is the product of the sampling time and the number of samples. The relative scaling is given by total integral, divided by the number of samples.

The dose is divided by the number of samples.

NOTICE

IMPORTANT NOTICE

ABSORBED DOSE VALUE

Provided that No. of samples is set to 1, the absorbed dose value is not affected by renormalization, and always represents the total accumulated dose.

NOTICE

IMPORTANT NOTICE

NUMBER OF SAMPLES

It is not recommended to set the no. of samples other than 1.

Movie mode: The measurement time is the product of the sampling time and the number of samples. The relative scaling for single frames is given by total integral, divided by the number of samples.

The total movie time is the product of the measurement time and the number of movie images.

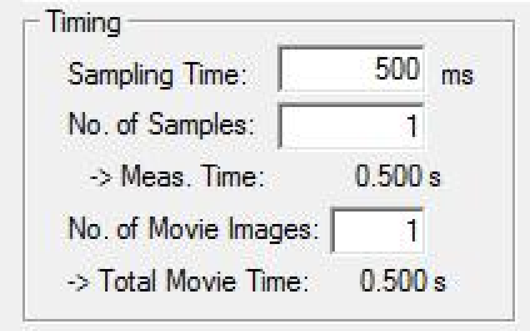

An example of the relation between the measurement time and the total movie time.

NOTICE

IMPORTANT NOTICE

SAMPLING TIME ≤40 MS

If the sampling time is ≤40 ms, the allowed number of images is limited, due to firmware limitations and limitations that might occur in the local network.

The movie acquisition can be stopped manually, without data loss.

At the end of a movie acquisition an integral frame is produced, containing the sum of all required frames. The relative scaling of this integral frame is divided by the number of frames in the movies (regardless if the movie has been aborted manually or completed).

The dose is divided by the number of samples.

NOTICE

IMPORTANT NOTICE

ABSORBED DOSE VALUE

Provided that No. of samples is set to 1, the absorbed dose value is not affected by renormalization, and always represents the total accumulated dose.

NOTICE

IMPORTANT NOTICE

NUMBER OF SAMPLES

It is not recommended to set the no. of samples other than 1.

NOTICE

IMPORTANT NOTICE

TOTAL MOVIE TIME

In Movie Measurement mode (i.e. when checking dynamic or step and shoot fields), the total movie time must be longer than it takes to perform the treatment. Always add some margin for the startup of the accelerator.

5. Select an algorithm (or **None**) in the **Algorithm** drop-down list.

NOTICE

IMPORTANT NOTICE

SAVING THE RAW DATA

It is not recommended to use any interpolation algorithm during acquisition, as the original resolution cannot be retrieved later.

6. Enter a value for **Grid Resolution**. Min value is 0.01 cm.
7. **Channels**: Click to open the **MatriXX Channels** dialog. See *7.4.5.3 Deactivate Channels*.

The checkboxes in the **Flags** section indicate the status of calibrations and background measurement:

Item	Checkbox marked	Checkbox unmarked
Correction	The Uniformity Correction Matrix is applied	The Uniformity Correction Matrix is not applied
User Calibration	The User Calibration is applied	The User Calibration is not applied
Calib. Output	The k_{user} factor is applied	The k_{user} factor is not applied
Temp. Corr.	The k_{pT} factor is applied	The k_{pT} factor is not applied
Press. Corr.	The k_{pT} factor is applied	The k_{pT} factor is not applied
Background	Background is measured for the current setup	Background is not measured for the current setup

See *7.4.3 I'mRT MatriXX Uniformity Correction Matrix* and *7.4.4 Add Correction Factors*.

⚠ WARNING

WARNING

DEVICE-INTERNAL CALIBRATION DATA MUST BE ACTIVATED

Activation of the Uniformity Correction Matrix is mandatory. If the **Activate device-internal calibration data** checkbox in the **Correction/Calibration** dialog is not marked, the measured image will not be corrected.

Option: Change the default background measurement time, by clicking the **Browse** button in the **Flags** section.

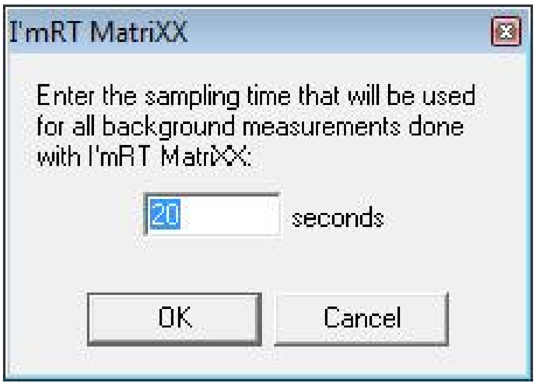

The entered time is persistent between sessions.

7.4.5.3. Deactivate Channels

It is possible to deactivate up to 10 channels in a measurement. In the measured image the deactivated channels will be replaced by the average of the surrounding perpendicular channels.

7.4.5.3.1. Deactivation

1. Click the **Channels…** button in the **MatriXX Meas.** tab in **Measurement Parameters**.

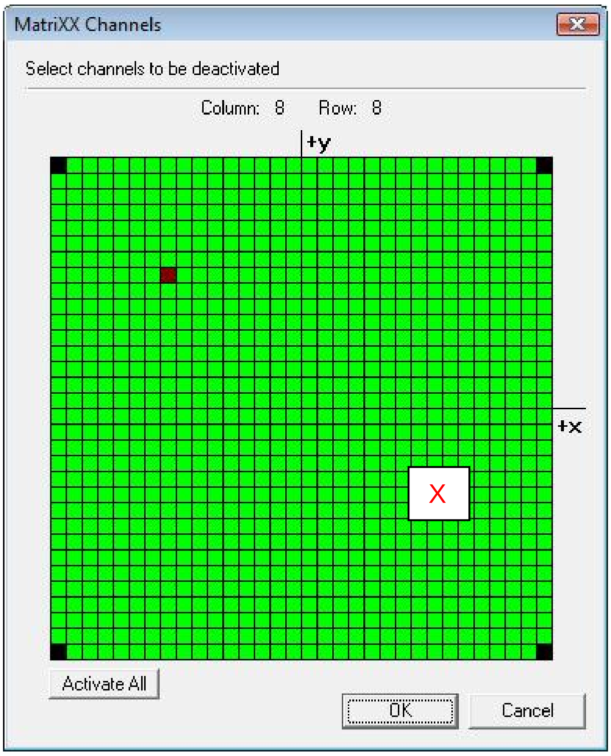

2. Select a channel by moving the cursor and click to deactivate the channel. The deactivated channel is marked with red color. Toggle between deactivated (red) and activated (green) by clicking on the channel.

To remove all deactivations, click **Activate All**.

The four corner pixels are by default deactivated.

NOTICE

IMPORTANT NOTICE

ADJACENT CHANNELS

Two perpendicular adjacent channels must not be selected.

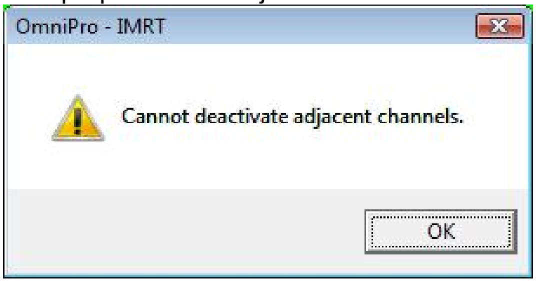

7.4.5.3.2. View Deactivated Channels

Deactivated channels can be viewed in the info parameters for a MatriXX measurement.

Select ***Parameters*** in the ***View*** menu. Select the **MatriXX Meas.** tab.

When moving the mouse cursor to the **Channel** button, a tooltip will display how many channels that are deactivated. Click the button to view the deactivated channels.

If no channels are deactivated, the **Channel** button will be disabled.

7.4.5.4. Sampling Time (Gain Setting)

1. Select the **MatriXX Meas**. tab in **Measurement Parameters**. Select **Not scaled** as the scaling mode.
2. In the **Timing** section, select a start value for the sampling time, e.g. 20 ms.
3. Click **OK** to close the dialog.
4. Measure the background. See *0 Perform Background Compensation*.
5. Select the menu command **Measure:Single Shot**.
6. Start the accelerator.
7. Click the **I'mRT MatriXX** toolbar button **Measure**.

After completed measurement the resulting image is displayed.

8. Stop the accelerator.
9. Check with the cursor, within the ROI (right-click command Zoom ROI), or just by looking at the image, that the maximum dose is around 100%. To view the dose legend, right-click and choose ***Show Legend***.

   If the maximum signal is much higher than 100%, decrease the sampling time.

   If the maximum signal is much less than 100%, increase the sampling time.
10. Repeat the measurement to check if the signal level is now about 100%.

7.4.5.5. Perform Background Compensation

NOTICE

IMPORTANT NOTICE

TURN OFF THE ACCELERATOR

During background definition the accelerator must be turned off.

The background procedure performs a measurement of the signal level on a “dark” image. The defined value will be subtracted from all calculations and presentations.

Click the toolbar button **Background**. The measurement starts.

The button remains dimmed until the measurement is completed.

Background is always measured with one sample, for 20 seconds. To adapt the background level to the level of measured signals, the values are rescaled with the factor sampling time/background sampling time.

NOTICE

IMPORTANT NOTICE

THE BACKGROUND VALUE IS VALID DURING ONE SESSION

The background value can be used for all subsequent measurements during a session. If the device or the software is turned off, a new background value must be defined before the next measurement.

The default background measurement time is 20 seconds. It is possible to change the time period to any longer time. To do this, go to **Measure/Edit Parameters/MatriXX Meas.** Change the background measurement time by clicking the **Browse** button in the **Flags** section.

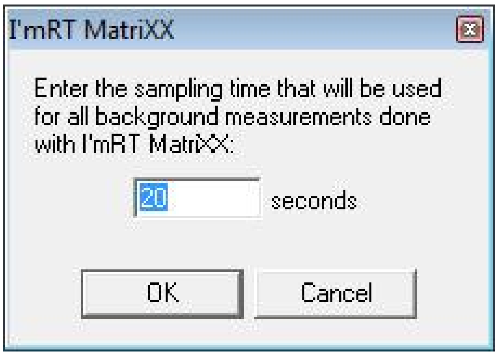

The entered time is persistent between sessions.

To view the background values of all chambers perform a measurement without irradiation and with background compensation switched off. Export this data file i.e. as a .txt file and a table of all 1020 chamber values for background will show up.

7.4.5.6. Field Tab

Select the **Field** tab. The options available in this dialog are dependent upon the specifications setup in the **Radiation Device Setup** dialog.

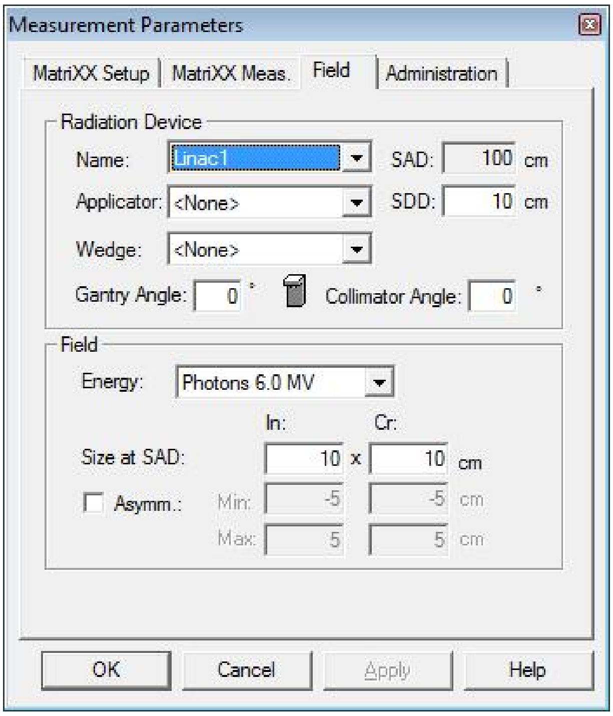

Select the radiation device, the wedge, the applicator, and the energy to be used.

Enter the SDD, and the gantry and collimator angle values.

Specify the field size used as reference field for the selected energy at SAD in inline and crossline.

7.4.5.7. Administration Tab

Select the **Administration** tab.

Since the information on this tab is already defined in the **Clinic setup** dialog, you only have to check if the information is correct.

If the information is correct, click **OK**.

7.4.5.8. Save the Measurement Parameters

It is possible to save the measurement setup.

See *10.4.4 Save measurement parameters*.

7.4.6. Measurement

When I'mRT MatriXX or MatriXX Evolution has been selected as measurement equipment, the corresponding toolbar and menu options will be expanded.

7.4.6.1. Select Measurement Mode

(Please also refer to chapter *7.4.5.2.2 MatriXX Meas. Tab*)

Click the appropriate toolbar button:

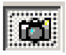 for Single shot mode

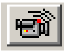 for On-line measurement mode

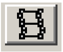 for Movie mode.

Depending on the measurement situation, different measurement modes are available for best results:

Single Shot Mode

In this mode, one single image is composed of a set of single frames (each one measured with the selected measuring time). The composed image contains the average of the selected number of samples.

If background compensation is activated, the background is subtracted from each individual frame.

Movie Mode

In this mode, a given number of movie pictures (single shots, as described above) are acquired. Each picture, as well as one integrated image, are saved.

NOTICE

IMPORTANT NOTICE

NUMBER OF MOVIE IMAGES

If the No. of Movie Images is set to “0” the movie will not stop until the Stop Movie button is pressed.

If background compensation is activated, the background is subtracted from each individual frame.

If no interpolation is selected, the measured data is displayed on-line.

Use this measurement mode e.g. to analyze dynamic wedges, or MLC fields.

NOTICE

IMPORTANT NOTICE

LOW COMPUTER PERFORMANCE

If the computer performance is low, display of movie data may be delayed.

NOTICE

IMPORTANT NOTICE

SHORT SAMPLE TIMES

For low sample time values (<500 ms), an accumulated background rounding error of values may occur.

On-line Mode

In this mode, single shots, as described above, are measured continuously and displayed on-line. Only the last five images are saved.

If background compensation is activated, the background is subtracted from each individual frame.

Use this measurement mode e.g. to trim the field parameters of the accelerator. By looking at the inline and crossline profiles, it is easy to check symmetry and flatness parameters in both orientations simultaneously.

7.4.6.2. Start and Stop the Measurement

Single Shot Mode Measurements

Click the toolbar button **Measure**.

After measurement is completed the resulting image is displayed.

On-line Mode Measurements

Click the toolbar button **Start On-line**.

The images are displayed during the measurement.

When the treatment is completed, click **Stop On-line** to stop the measurement.

Only the last five images are saved. To check these images open the **Field list** dialog by selecting ***View:Field*** list.

Movie Mode Measurements

Click the toolbar button **Start Movie**. Start the treatment as planned.

When the pre-defined time has elapsed, the measurement stops. If you want to stop the measurement before the total time has elapsed, use the **Stop Movie** button. The current image will be finished before the measurement stops.

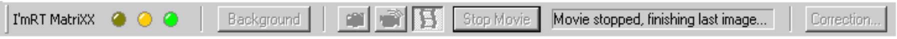

When the measurement is completed the resulting image is displayed. If the **Stop Movie** button was used to stop the measurement, the images that were measured until the stop will be calculated and stored, and an integral image is calculated and stored.

To check single movie pictures, or to run the movie, open the **Field list** dialog by selecting ***View:Field*** list.

Manually Start and Stop Measurements using Movie Mode

If the No. of Movie Images in the Parameters is set to “0” the movie starts by clicking the **Start Movie** button. The software measures as long as the **Stop Movie** button is clicked.

7.5. VMAT/RapidArc – Parameter setup

7.5.1. Hardware Setup

In general the setup for rotational treatments is the same like for I'mRT verification. Therefore please refer to the former chapters for general setup and settings.

Setup the MatriXX Evolution as described in *7.2.1 Setup MatriXX with MULTICube.*

To get the highest level of precise the usage of the MULTICube is highly recommended. The usage of MULTICube assures that the setup in the CT while scanning and the setup under the linac for verification is exactly the same position.

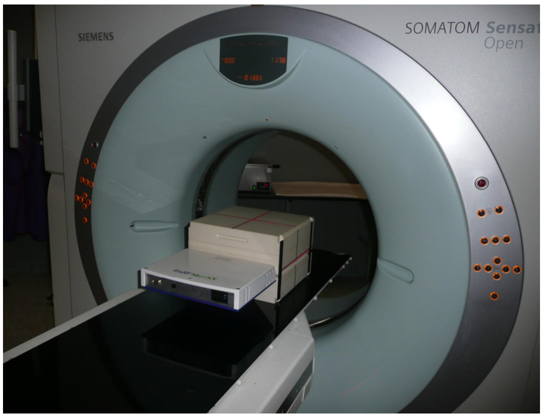

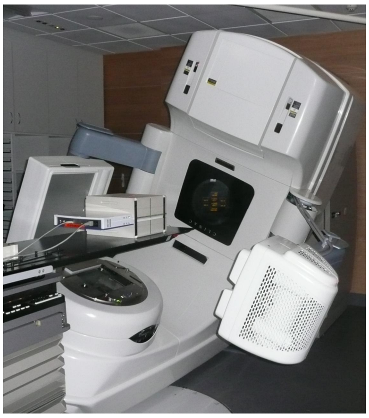

In this constellation the build-up of MatriXX Evolution is 11 cm of Plastic Water®. Please double check the SSD of 89 cm.

MatriXX Evolution is delivered with the Gantry Angle Sensor. For Mounting, Alignment and Calibration the inclinometer please refer to chapter *7.3.4 Gantry Angle Sensor.*

7.5.2. Setup Measurement Parameters

Using the MULTICube please make sure to measure a correction factor for this constellation. Therefore please refer to *7.4.4 Add Correction Factor*. For rotational measurements use the "Dose Mode" for measuring this factor.

For general software settings please refer to the chapter *7.4.5 Measurement Setup*.

NOTICE

IMPORTANT NOTICE

TAKE TREATMENT COUCH IN TPS INTO ACCOUNT

For a precise verification please make sure that the TPS takes the treatment couch into account for calculation the plans.

A recommendation for the measurement parameter setup is given in the following screen shot:

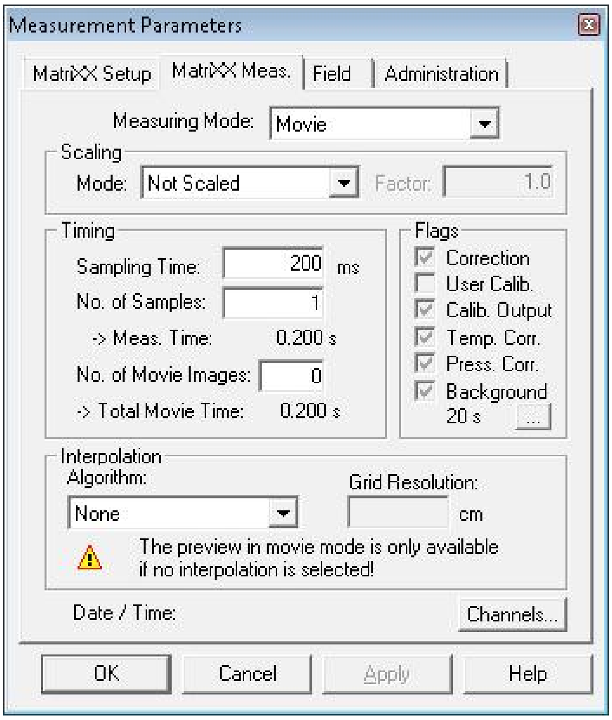

If the **Movie Mode** and a **Sampling Time** of 200 ms is selected the software integrates over 200 ms for each snap. After stopping the measurement an integral over the whole measurement time will be calculated. If the **No. of Movie Images** is set to “0” a manually start and stop mode is active. (Please see *7.4.5 Measurement Setup*.)

NOTICE

IMPORTANT NOTICE

SAMPLING TIME FOR ROTATIONAL TREATMENTS

For movie mode please do not use a sampling time smaller then 100 ms. The amount of measurements data would increase too much.

After the measurement is done the data can be corrected for the angular dependence of the MatriXX. Therefore please refer to chapter *7.3.4.4.3 Gantry Angle Correction.*

7.6. TomoTherapy - Specific Workflow

The MatriXX Evolution is intended for QA of 360° digital IMRT and IMAT fields, and for validation under exact treatment conditions, using TomoTherapy and conventional linac treatment systems.

MatriXX Evolution used together with the Gantry Angle Sensor (*Gantry Angle Sensor*), is required for the measurements.

The analysis can be made in OmniPro I'mRT, after import of DQA files from TomoTherapy (*7.6.2.6 Export of DQA files to OmniPro I'mRT*), or in TomoTherapy, after export of a MatriXX TIFF file from OmniPro I'mRT (*7.6.2.5 Export of TIFF files to TomoTherapy*).

NOTICE

IMPORTANT NOTICE

EXPORT OF MATRIXX TIFF FILES RECOMMENDED

Export of a MatriXX TIFF file, to be analyzed in TomoTherapy, is recommended as a better alternative.

TomoTherapy® is a trademark of TomoTherapy Inc., USA.

7.6.1. Preparation

7.6.1.1. CT Scan of the MatriXX

<u>CT Setup and Configuration</u>

Use the same configuration for the MatriXX as the one that will be used in TomoTherapy.

The physical limitations of the TomoTherapy couch (i.e. it is impossible to raise the couch above a certain point) must be taken into consideration. The plan that will be administered must allow that the plane of the MatriXX will be within the physical limitations of the couch, once the plan is superimposed on the MatriXX.

As a general rule, 5 cm additional backscatter material will be sufficient for most plans, but not for all. If in doubt, choose using more backscatter material rather than less. 10 cm or more can be regarded as sufficient.

Using the MULTICube phantom in its full or "light" configuration will eliminate this problem for virtually all plans.

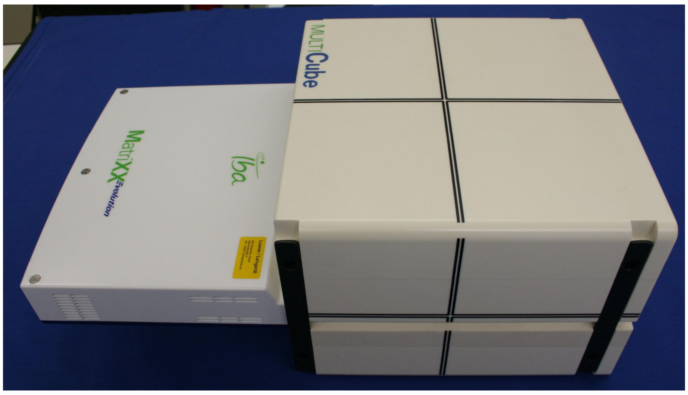

NOTICE

IMPORTANT NOTICE

DIFFERENT CT SCANS FOR DIFFERENT MATRIXX'S

For every MatriXX a CT is necessary. Otherwise deviations in dose calculation and measurements are possible. Ensure that the correct CT scan is used.

Slice Width

The slice width is important, because the exported TomoTherapy DQA (Delivery Quality Assurance) files derive their resolution in the x-direction (parallel to the couch in the DQA) from it.

If the analysis is going to be made in OmniPro I'mRT, use 2 mm slice width, or less. A better resolution improves the landmark selection on the further procedure.

NOTICE

IMPORTANT NOTICE

BANDING IN THE GAMMA ANALYSIS

If 3 mm slice width is used, it will work for analysis in OmniPro I'mRT, but the resolution of the DQA export will be non-isotropic, and "banding" will appear in the gamma analysis:

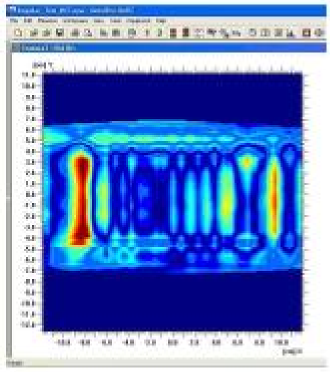

7.6.1.2. Alignment

Align the MatriXX to the laser cross-hairs meticulously, to avoid obtaining skewed slices.

If a MULTICube phantom is not used, it is recommended that a couple of markers are placed right on the cross-hairs on the MatriXX housing. Thus the center of the device will easily be identified on the CT images.

If a MULTICube is used, it is not necessary to use markers, since the engraved crosshairs of the cube will be visible in the CT images.

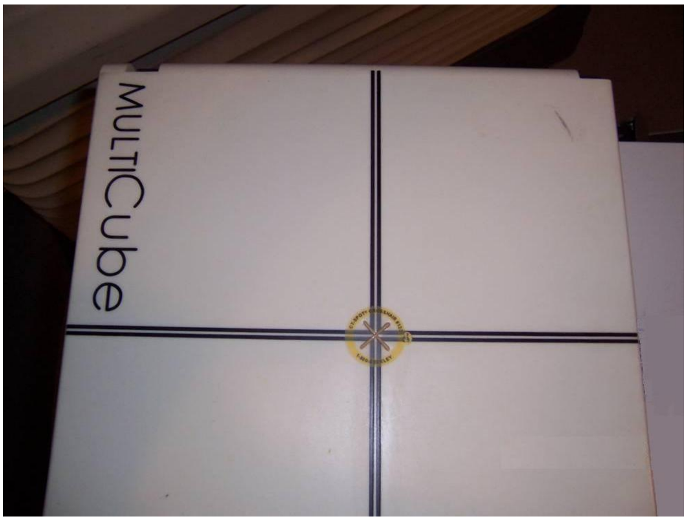

CT DICOM Export Set
TomoTherapy requires phantom CT sets to be named _phantom. In the phantom list in TomoTherapy, the name of the phantom will be the Patient ID that was assigned at the CT, not the patient name. The _phantom will not be displayed in the phantom list.

Thus the Patient ID at the CT should be e.g. MatriXX, and the patient name at the CT must be _phantom.

No other file name will be recognized.

7.6.1.3. Pre-irradiation of the MatriXX

The MatriXX requires pre-irradiation prior to use. The most convenient way to do it is to use a C-Arm Linac, if available (*I'mRT MatriXX – Setting up for Measurements / Pre-irradiation*). Perform the pre-irradiation, un-plug the MatriXX, and move it to the TomoTherapy.

Pre-irradiation can also be performed in the TomoTherapy, either by using a static field, or a "cylindrical" helical plan from TomoTherapy.

Calculation of the Pre-irradiation Parameters
Pre-irradiation dose and couch velocity can be calculated as follows:

D = (D'/L) × (WT), V = L/(60T)

D: pre-irradiation dose (cGy)

D': nominal dose rate of HIART–II (cGy/min)

L: length of topographic irradiation (cm) *)

W: slit width (cm)

T: total irradiation time (min)

60T: total irradiation time (sec)

V: couch velocity (cm/sec)

*) Length of topographic irradiation = field of view

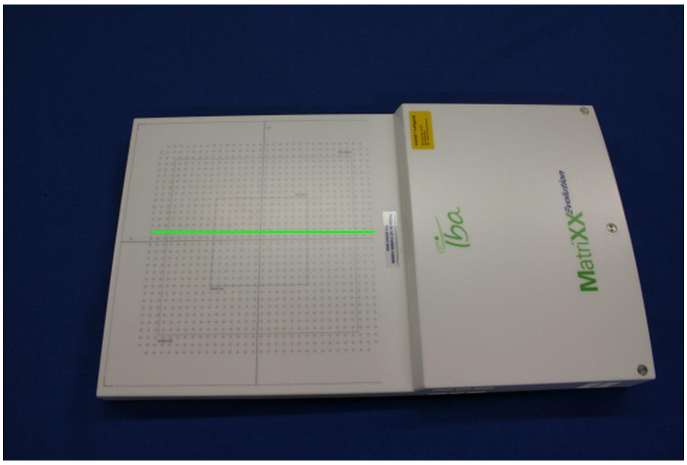

= L (26 cm)

Example:

D’	858 cGy/min
L	26 cm (MatriXX field width)
W	1 cm
T	3 min
60T	180 sec

With these assumed values the pre-irradiation dose (D) will be 99 cGy, and the couch velocity (V) will be 0.144 cm/sec.

Pre-irradiation dose for a typical TomoTherapy dose rate:

In the table below the pre-irradiation dose and couch velocity are calculated for a typical TomoTherapy dose rate and varying T and W values.

D’ = 858 cGy/min

L = 26 cm

T (min)	W (cm)				V (cm/s)
	1.0	1.8	2.5	5.0	
	Dose (cGy)	Dose (cGy)	Dose (cGy)	Dose (cGy)	
1.0	33	59	83	165	0.433
1.5	50	89	124	248	0.289
2.0	66	119	165	330	0.217
2.5	83	149	206	413	0.173
3.0	99	178	248	495	0.144

NOTICE

IMPORTANT NOTICE

THE TOMO THERAPY MVCT CANNOT BE USED FOR PRE-IRRADIATION

The TomoTherapy MVCT cannot be used for pre-irradiation, since it does not deliver the sufficient quantity of MUs. The energy is only 3 MV.

NOTICE

IMPORTANT NOTICE

USE ONLINE MODE IN OMNIPRO I’MRT

For pre-irradiation use the Online Mode in the OmniPro I’mRT software for better observing.

7.6.1.4. Background Subtraction and Energy Calibration

After pre-irradiation, the next step would normally be to do the background subtraction and to obtain the k_{User} factor for energy calibration (see *7.4.4 Add correction factors*).

The need for these procedures depends on which export method that will be used.

7.6.2. TomoTherapy specific setup

7.6.2.1. TomoTherapy Export Methods

DQA Export

No dose is acquired from DQA, only a relative map. Thus the k_{User} factor is irrelevant and does not need to be measured.

TomoTherapy Planar DICOM Export

NOTICE

IMPORTANT NOTICE

TOMO THERAPY DICOM PLANAR EXPORT METHOD TO BE RELEASED

This export method is not yet released at the time of publishing this manual.

The k_{user} factor must be obtained in order to get the same information as with a C-arm Linac. A cylindrical plan to be delivered helically must be created. A known dose will be administered to the four central chambers of the MatriXX.

TIFF Export from OmniPro I'mRT

The k_{user} factor must be obtained in order to get the correct dose information.

7.6.2.2. Creation of the Plan

Export the MatriXX CT images to the TomoTherapy planning station, and then select the plan that will administered to the MatriXX.

When the dose distribution is superimposed on the MatriXX phantom in the TomoTherapy, it is usually considered necessary that the distribution over the phantom is such that it includes the PTV, and other information, e.g. spine. To facilitate this, take into account the fact that the effective point of measurement, P_{eff}, of the MatriXX is at a depth of 3 mm.

The air gaps of the ion chambers will be visible in the CT images rendered by the planning system. Thus it is possible to position the desired planar dose distribution at the MatriXX P_{eff}, which is just slightly beneath the top electrodes of the chambers.

7.6.2.3. MVCT

Align the MatriXX

1. Align the MatriXX with the TomoTherapy lasers. There are two sets of lasers: red and green. The green laser defines the virtual isocenter in TomoTherapy, 70 cm offset from the real isocenter. The red lasers can be viewed and moved manually from the virtual isocenter, to match the patient/phantom fiducially. Usually it is suitable to align the MatriXX to the red patient laser/coordinate system.
2. Select the center axes of the device in order to define the origin of the phantom. This is done by moving the red laser around in the planning station. The BBs or the MULTICube cross-hairs are useful in this procedure.

NOTICE

IMPORTANT NOTICE

ALIGNMENT

Do not align the red laser to the center of the chamber air cavities in the coronal plane. The P_{eff} is on the top electrode.

3. Perform the MVCT.

NOTICE

IMPORTANT NOTICE

MVCT AREA

It is not necessary to do an MVCT of the entire active area of the MatriXX. A few centimeters on either side of the transverse zero slice is sufficient to get the BBs in the MVCT and to make the center of the device visible.

4. Align the CT and MVCT images, using the TomoTherapy checkerboard.

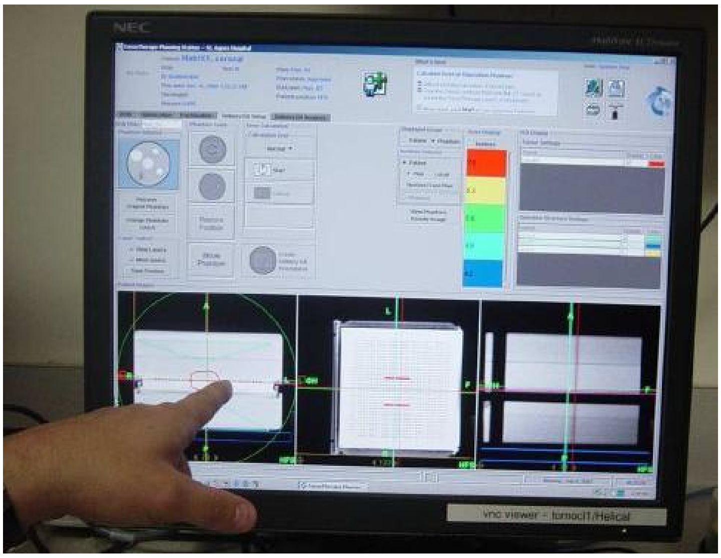

5. Save the phantom position, and create the plan.

7.6.2.4. Treatment

Perform the treatment with the MatriXX in Movie mode (see chapter *7.4.5 Measurement Setup*).

7.6.2.5. Export of TIFF Files to TomoTherapy

1. Select ***File/TomoTherapy/Export Image File*** in OmniPro I'mRT to export the TIFF file.
2. Define the registration points in the TIFF image, by using the "General Coronal" procedure in TomoTherapy.

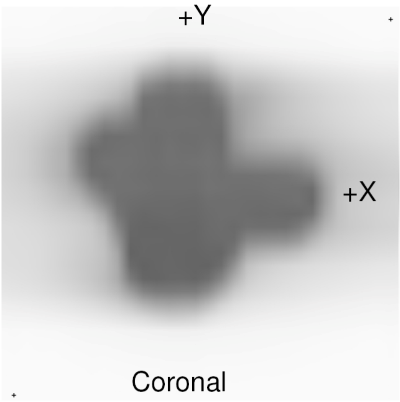

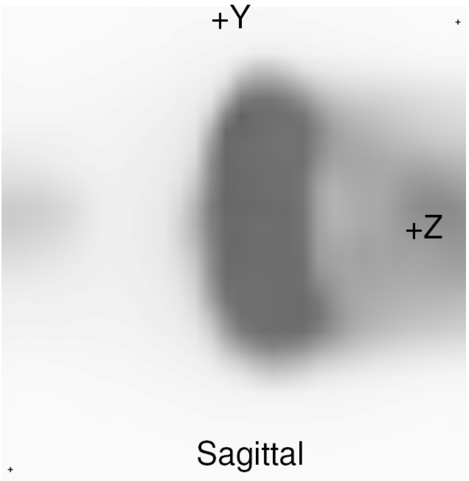

NOTICE

IMPORTANT NOTICE

ONLY TWO REGISTRATION POINTS IN THE TIFF IMAGE

Since there are only two registration points in the TIFF image, the “Coronal plus 4 lasers” procedure cannot be used.

3. Select the plane that shall be exported. A plane that is near the center of the ion chamber air cavities shall be selected.

NOTICE

IMPORTANT NOTICE

AIR CAVITIES MAY NOT BE VISIBLE

If a plane at the top electrode is selected, the air cavities will not be visible.

4. Two registration points will be requested by TomoTherapy. Use the hash marks. Mark the points on the dose plane, corresponding to the hash marks. The hash marks correspond to the chambers on the MatriXX diagonal to the blank spots on the top right corner and the bottom left corner of the device.

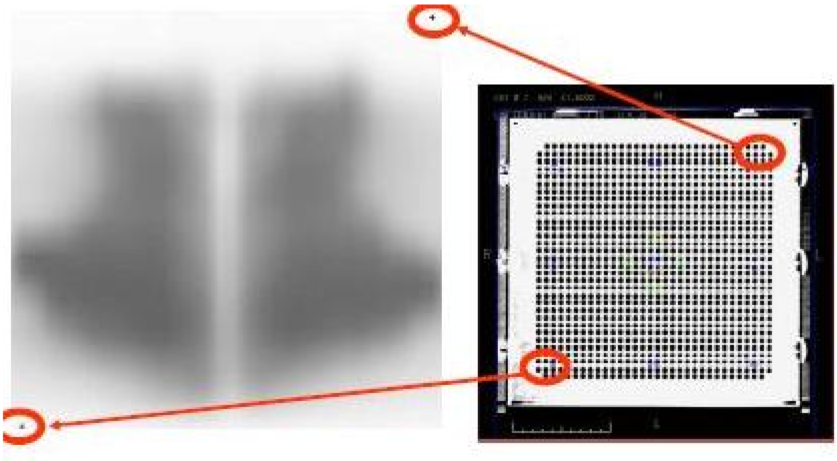

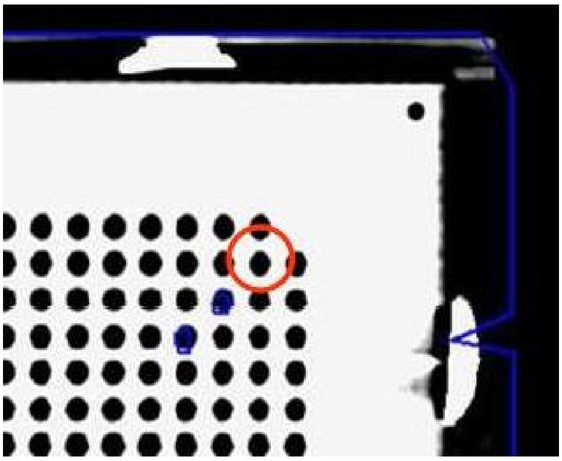

NOTICE

IMPORTANT NOTICE

ENSURE THAT THE CORRECT SPOTS ARE MARKED

The MatriXX may be displayed sideways in the DQA workspace. Ensure that the correct spots are marked.

7.6.2.6. Export of DQA Files to OmniPro I'mRT

<u>Creation of the DQA Export</u>

If the analysis will be made in OmniPro I'mRT, prepare the DQA export from TomoTherapy as follows:

Use the "Text DQA Header and Image Files" for export to OmniPro I'mRT. These files are created by TomoTherapy for export.

NOTICE

IMPORTANT NOTICE

CUBIC DICOM EXPORT

The cubic DICOM export method is not recommended.

Select a random film in the TomoTherapy film panel, and mock register it. The film can be of any size. This action will enable the dose plane export button.

7.6.2.7. Import of DQA Files to OmniPro I'mRT

Select ***File/TomoTherapy/Import ASCII File*** in the OmniPro I'mRT software.

NOTICE

IMPORTANT NOTICE

SAVING DQA FILES

It is not recommended to save the DQA files in sub-folders, since certain sub-folder names or characters will disrupt the function. Copy the files to the desktop, and work from there.

The imported file will look similar to the example below:

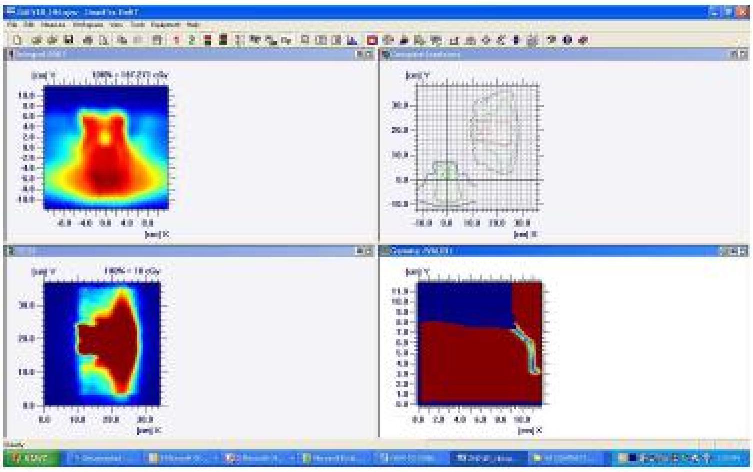

Take notes of the isodose lines and the coordinates corresponding to the import. The position to the import relative to measured data is arbitrary, since there is no coordinate system in the DQA export file. Data must be registered manually, using the ***Turn Array***, ***Flip Horizontal***, and ***Move data*** functions. Turn the array 90degrees clockwise, select ***Flip Horizonta***l, and use the ***Move Data*** function to register the images manually. These functions can be pre-defined in ***Macros***, under the ***Edit*** menu.

The following macro is recommended:

- Turn array 90 degrees in the positive X direction
- Flip horizontal
- Convert grid to 0.762 cm - optional
- Convert grid to 1 mm in order to frequency match the data with the interpolated MatriXX data - optional

NOTICE

IMPORTANT NOTICE

CHANGE THE STEP SIZE

It is recommended to change the step size to 1 cm, instead of 1mm, for the initial alignment.

Use the isodose lines to align data, and then use the profiles for fine-tuning. If appropriate isodose lines are picked, it will be possible to hone in on the ideal registration point without the use of profiles.

7.7. I'mRT Phantom Measurements

7.7.1. Dose Planning

1. Perform a CT-scan of the I'mRT Phantom, and calculate the dose plan you want to verify for the I'mRT Phantom.
2. Use a marker in the I'mRT Phantom origin that can be clearly seen on the CT image, and that can be used for later positioning of the I'mRT Phantom, e.g. a small piece of metal, or a characteristic structure of the phantom.
3. Set the isocenter in such a way that it will be easy to position the I'mRT Phantom under the gantry.
4. Note down the distance from the phantom origin to the isocenter, and the distance from the phantom origin to the origin for the dose calculation (in case the origin for dose calculation is not at isocenter position).

7.7.2. Absolute Dose Measurement in I'mRT Phantom

Absolute Dose Measurements, in single points inside the I'mRT Phantom, can be used to check the absolute dose for a reference point.

7.7.2.1. Measurement Setup

7.7.2.1.1. Prerequisites

Before starting an I'mRT Phantom measurement the following is required:

- The I'mRT Phantom hardware setup is completed. See instructions below.
- The software setup in OmniPro I'mRT is completed. See *7.4.5 Measurement Setup.*

7.7.2.1.2. Hardware Setup

1. Position the I'mRT Phantom onto the treatment couch, and align it according to the treatment plan.
2. Tip! One possibility is to align the I'mRT Phantom in such a way that the I'mRT Phantom is parallel to the couch and the lasers (room coordinate system), and the phantom origin is in isocenter position.
3. Move the treatment couch by the distance between marker and isocenter (as noted when calculating the dose plan).
4. Insert an ionization chamber into the point where you want to verify the absolute dose.
5. Follow the instructions in the hardware manual of the Dose 1 dosimeter.

7.7.2.1.3. Measurement

1. Reset the Dose 1.
2. Start the treatment (start the accelerator).
3. After completed irradiation, note down the measured dose.

7.7.3. Analysis

NOTICE

IMPORTANT NOTICE

DOSE IMPORT

Define the phantom coordinates, orientation, and position correctly during dose import. See *Data Import* / *Dose plan import* for further descriptions.

7.7.3.1. Import the Treatment Plan:

1. Select ***Measure:Absolute Dose Value***. The **Absolute Dose Verification** dialog opens.

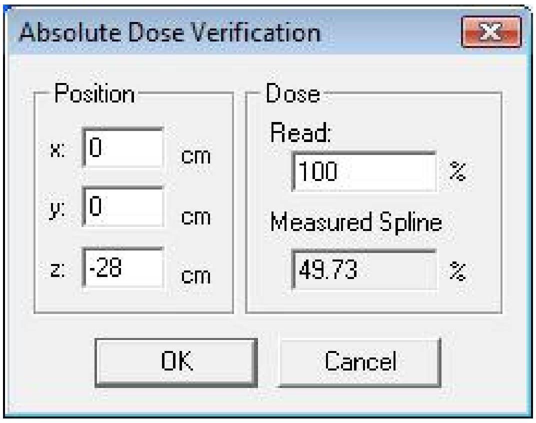

2. Enter the coordinates of the measurement point and the measured dose. The measured dose and the calculated dose are displayed in the dialog.
3. To save the values in the workspace, click **OK**.

7.7.4. Dose Measurements Using Films in the I’mRT Phantom

Film measurements in the I'mRT Phantom can be used to check the relative dose distribution in several planes, with different offset inside a volume.

Radiographical films (in their envelopes) can be used to check the dose distribution in axial planes. To check the dose distribution in other directions, (sagittal, coronal) insert "naked" films inside the cube of the I'mRT Phantom.

The cube inside the I'mRT Phantom can also be used stand alone for verification of head treatments.

NOTICE

IMPORTANT NOTICE

I’MRT PHANTOM REQUIREMENTS

OmniPro I’mRT supports a large variety of body phantoms. Please check that they fulfill the following requirements:

- The film orientation can be sagittal, coronal, or axial.
- Phantom origin must be identifiable on the CT slice, e.g. via a marker or a geometrical outline.
- It must be possible to mark the films. The distance between the film marking and the phantom origin must be known.
- The composition of the material shall be water or tissue equivalent.

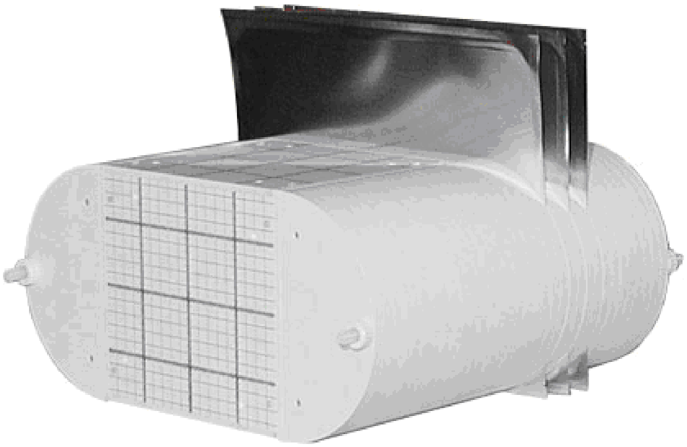

The picture above shows the default orientation of the I’mRT Phantom. If you look in the direction towards the gantry, the origin marker for each film slice is on the right side of the center. If the phantom is rotated, you have to adapt the marker position in the equipment setup.

NOTICE

IMPORTANT NOTICE

HEAD AND NECK TREATMENT

If you want to verify a head and neck treatment, perform a CT-scan and calculate the treatment plan for the cube only.

An example of a dose plan calculated for a patient:

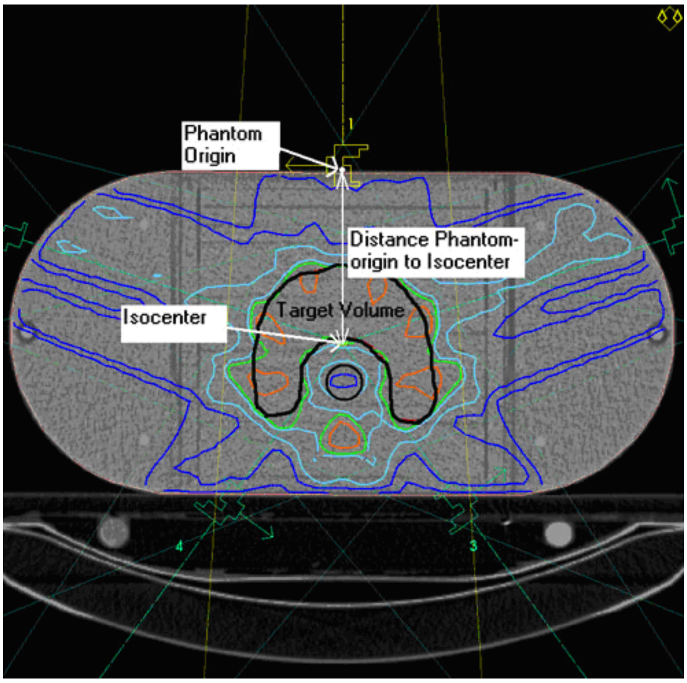

The marker in the origin of the I'mRT Phantom coordinate system is clearly visible. Note that this marker can be in any CT-slice, as long as the distance to the isocenter and to the origin for dose calculation is known.

⚠ WARNING

WARNING

FILM GETS SATURATED

Depending on the choice of film, the saturation dose of the film may be lower than the maximum dose to be verified. Scale down the treatment plan or select an appropriate film.

7.7.4.1. Measurement Setup

7.7.4.1.1. Prerequisites

Before starting the measurement and the film scanning, the following must have been done:

- The SCSI adapter (for LumiScan: ISA board) board is installed. See *4.4.1.1 SCSI*.
- The body phantom hardware setup and the software setup in OmniPro I'mRT are completed. See instructions below, and *6.2 Configuration of OmniPro I'mRT Software*.
- The film scanner setup is completed. See *4.4 Vidar Scanner Installation*, or *4.5 Kodak LS (LumiScan) Scanner Installation*.
- The film and film scanner calibrations are completed. See *7.9.5 Scanner Calibration* and *7.9.6 Film Calibration*.

7.7.4.1.2. I'mRT Phantom Setup

Tip! To avoid mixing up the films, mark each film in a corner far outside the beam.

WARNING

WARNING

MARKERS ON FILM

Markers for identification, orientation, or positioning may overlap or interfere with optical density, due to radiation. Mark your films outside the region where you want to determine the dose.

1. Mount the films into the phantom.
2. Mark the films (for later alignment and positioning).

NOTICE

IMPORTANT NOTICE

ALIGNMENT

The film may be misaligned either in its envelope, or tilted inside the phantom. To correct this misalignment after scanning, use two markers, or a line (e.g. the border of the phantom), to align the film to the body phantom coordinate system. The alignment markers must be parallel to the main axes of the phantom coordinate system.

NOTICE

IMPORTANT NOTICE

ABSOLUTE POSITION

It is important to know the absolute position of the film with respect to the phantom origin. Mark the film, at a precisely defined position in relation to the phantom origin. The absolute position of this marker is entered in the **Body Phantom setup** dialog in **Equipment**.

3. Position the I'mRT Phantom onto the treatment couch, and align it according to the treatment plan.

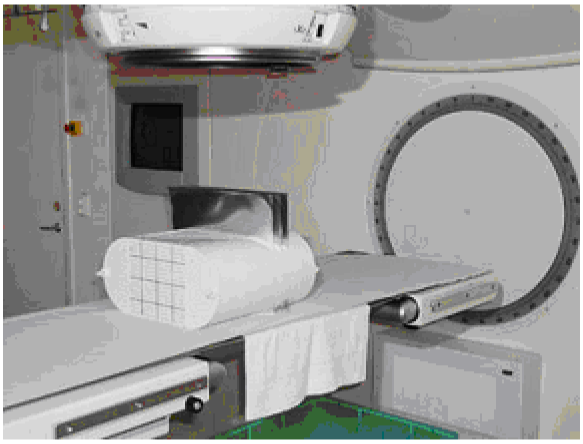

Tip! One possibility is to align the I'mRT Phantom in such a way that the I'mRT Phantom is parallel to the couch and the lasers (room coordinate system), and the phantom origin is in isocenter position. Then move the treatment couch by the distance between marker and isocenter (as noted when calculating the dose plan).

7.7.4.1.3. *Measurement*

Perform the treatment according to the treatment plan.

NOTICE

IMPORTANT NOTICE

VERIFYING ONE PART OF THE TREATMENT ONLY

If you want to verify only one part of the treatment (e.g. one field), insert new films for this field, take them out after irradiation and insert new films for the next field. Remember to mark the films.

7.8. Setup for I'mRT QA/BIS Measurements

7.8.1. General

With the I'mRT QA, 2D intensity maps can be measured with a time resolution down to 120 ms.

With BIS 710 the minimum sampling time is 240 ms.

Afterwards each shot or the integrated signal can be analyzed.

7.8.2. Calibration

7.8.2.1. I'mRT QA/BIS Correction Image

⚠ WARNING

WARNING

I'MRT QA/BIS CORRECTION IMAGE

The specification of the correction image file and the CCD area are mandatory. If you specify an incorrect or non-existing file and/or path, the measured image will not be corrected. If you specify an incorrect CCD area, the measured image will be incorrect. It will be very noisy and shifted, and may not appear fully on the screen. Before performing an I'mRT QA/BIS measurement, the Correction image file, found on the installation disk delivered with the I'mRT QA/BIS, must be copied to your computer.

The Correction image is individual for each I'mRT QA/BIS and will be used to remove inhomogeneities of the scintillator from the measured image. Before performing any analysis tasks the Correction image must be applied to the measured image.

1. Copy the Correction image file from the disk to an appropriate storage location on the computer's hard disk.
2. Start the OmniPro I'mRT software.
3. Select the menu command ***Equipment:I'mRT QA/BIS*** and define your I'mRT QA/BIS.
4. Select the menu command ***Equipment:Select Clinic and Device*** and select your I'mRT QA/BIS.
5. Select the menu command ***Measure:Correction***. The **Correction Image** dialog opens.
6. Click **Change**. A password dialog opens.

7. Enter the password "user". You have the possibility to change this password by selecting Change in the password dialog. The New Password dialog opens.

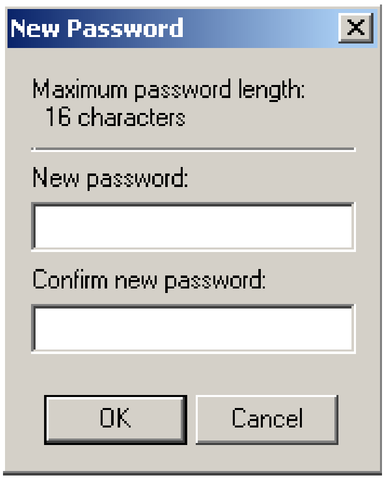

8. Enter and confirm your new password. Click **OK**.

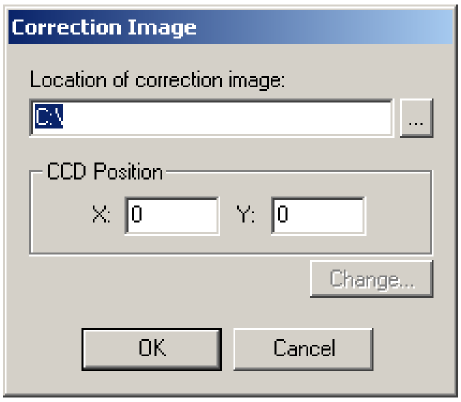

9. Click 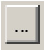. Select the correction image file. (Browse for the correction image file *.cal).
10. Enter the **CCD Position** values from the Factory Correction certificate for I'mRT QA/BIS.
11. Click **OK**.

7.8.3. Measurement Setup

7.8.3.1. Prerequisites

Before starting an I'mRT QA/BIS measurement the following is required:

- Frame grabber board installed. See the I'mRT QA/BIS Manual .
- Correction image applied. See *7.8.2.1 I'mRT QA/BIS Correction Image*.
- I'mRT QA/BIS software setup is completed. See *7.8.3.3 Define the I'mRT QA/BIS Parameters*.
- I'mRT QA/BIS hardware setup is completed. See *7.8.3.2 Hardware Setup*.

7.8.3.2. Hardware Setup

NOTICE

IMPORTANT NOTICE

FRAME GRABBER BOARD

The Frame grabber board must be installed in the computer that will be used for the measurements. Refer to the I'mRT QA/BIS Manual for further instructions.

⚠ WARNING

WARNING

I'MRT QA/BIS CORRECTION IMAGE

The specification of the correction image file and the CCD area are mandatory. If you specify an incorrect or non-existing file and/or path, the measured image will not be corrected. If you specify an incorrect CCD area, the measured image will be shifted and may not appear fully on the screen.

1. Ensure that the correction image is applied correctly. See *7.8.2.1 I'mRT QA/BIS Correction Image*.
2. Place the I'mRT QA/BIS on the treatment couch under the gantry, or mount it on the gantry.
3. Replace the protection plate with the positioning plate. Set the accelerator field to 20 × 20 cm. Level, position, and align the I'mRT QA/BIS using the lasers, hairline cross, and light field ruler (SSD distance).
4. Remove the positioning plate and insert the measurement scintillator. Take care that the position of the image device does not change when inserting the scintillator plate.

NOTICE

IMPORTANT NOTICE

LIGHT FIELD MEASUREMENT

If you want to measure the light field (for comparison of light field with radiation field), insert the light field scintillator instead.

5. Ensure that the scintillator is correctly mounted:

   Measurement scintillator

   The white side shall be located downwards; the black side shall be located towards the radiation source.

   Light field scintillator

   The label I'mRT QA/BIS Light S/N xxx shall be located downwards.

6. Connect the data cable to the frame grabber board in the PC.
7. Plug the power cord of the power supply into a mains power outlet. Verify that the green and yellow LEDs ("Power" and "Image on Demand") are illuminated.
8. Continue by defining the I'mRT QA/BIS parameters.

7.8.3.3. Define the I'mRT QA/BIS Parameters.

Select ***Equipment:Select Clinic and Device***. Select the clinic and ***I'mRT QA*** or ***BIS 710***. The **I'mRT QA/BIS** toolbar will be added.

Select ***Measure:Edit parameters***. The **Measurement Parameters** dialog opens.

The dialog is similar for I'mRT QA and BIS 710, except for the **Meas.** tabs, which differ slightly.

7.8.3.3.1. I'mRT QA/BIS 710 Setup Tab

1. Click the I'mRT QA/BIS 710 Setup tab.

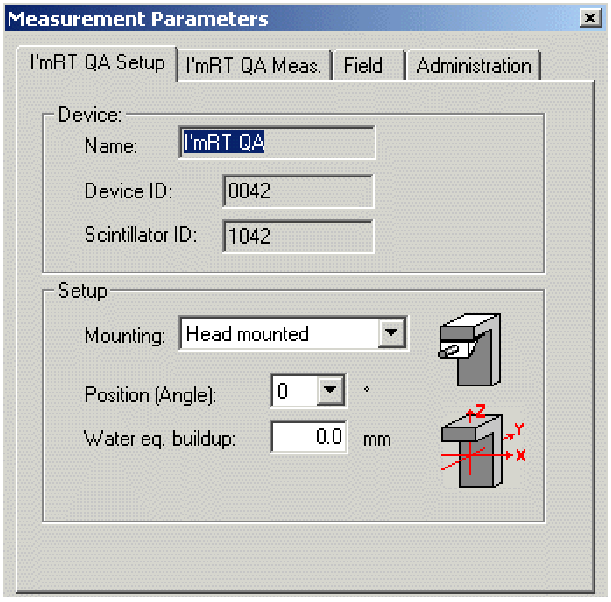

2. Define how the I'mRT QA/BIS is mounted in **Mounting**.
3. Define the image device position in relation to the accelerator, in **Position**.

The icon 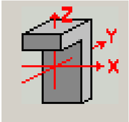 shows the relationship between the IEC and the I'mRT QA coordinate systems.

7.8.3.3.2. I'mRT QA/BIS 710 Meas. Tab

<u>I'mRT QA</u>

1. Click the I'mRT QA Meas. tab.

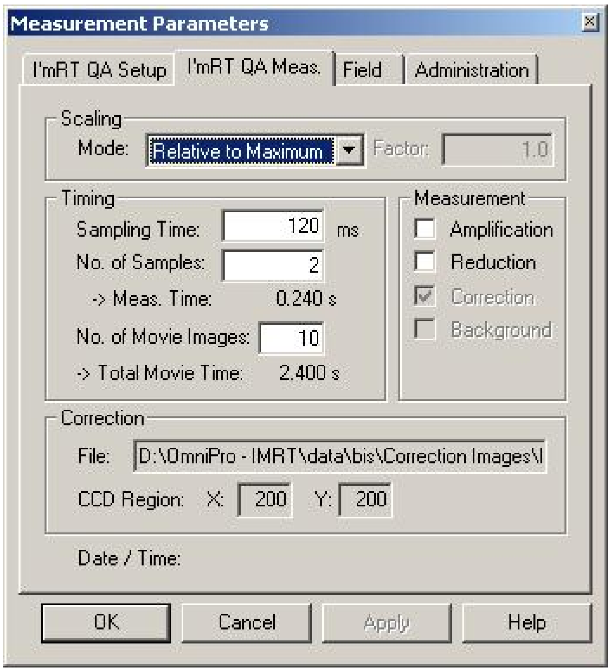

2. Specify information about the scaling mode to be used, in **Mode**.
3. In the **Timing** section, enter the sampling time (i.e. the integration time for one measurement) to be used, and the number of samples and movie images to be used.

NOTICE

IMPORTANT NOTICE

TOTAL MOVIE TIME

In Movie Measurement mode (i.e. when checking dynamic or step and shoot fields), the total movie time must be longer than it takes to perform the treatment. Always add some margin for the startup of the accelerator.

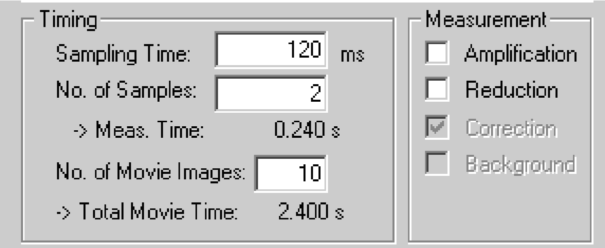

An example of the relation between the measurement time and the total movie time.

The measurement time is the product of sampling time and the number of samples. The total movie time is the product of measurement time and the number of movie images.

Correction: The path for the Correction Image and the CCD Region will be displayed as previously defined.

Date/Time: The date and time for the measurement will be automatically entered.

BIS 710

If BIS 710 is selected, the **Meas**. tab will be displayed as below.

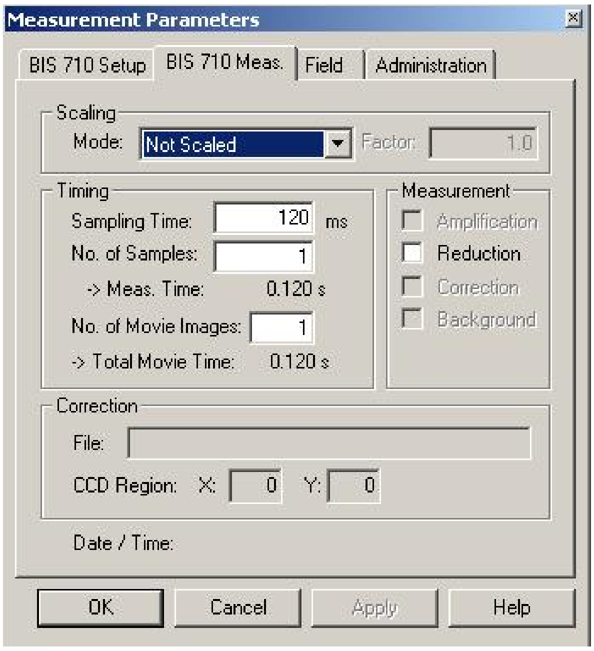

Enter data in **Scaling** and **Timing** as in the I'mRT QA Meas. tab.

NOTICE

IMPORTANT NOTICE

SAMPLING TIME

The minimum sampling time is 240 ms.

NOTICE

IMPORTANT NOTICE

AMPLIFICATION

The **Amplification** option is not available for BIS 710.

7.8.3.3.3. I'mRT QA/BIS 710 Field Tab

1. Click the **Field** tab. The options available in this dialog are dependent upon the specifications setup in the **Radiation Device Setup** dialog.

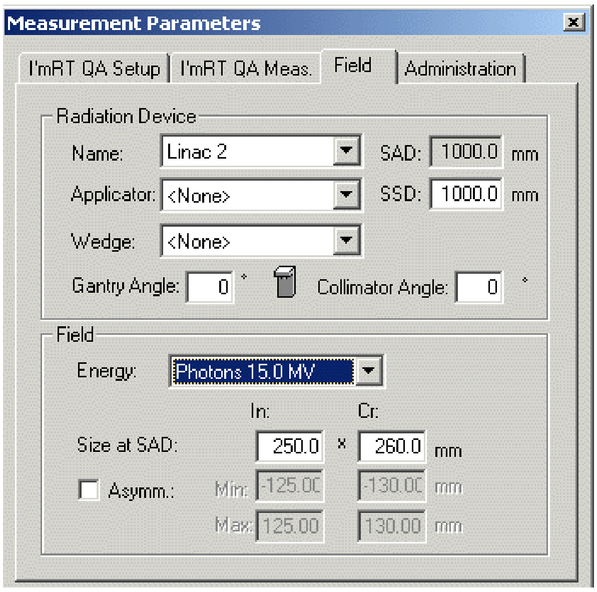

2. Select the radiation device, the wedge, the applicator, and the energy to be used.
3. Enter the SSD and the gantry and the collimator angle values.
4. Specify the field size used as reference field for the selected energy at SAD in inline and crossline.

7.8.3.3.4. I'mRT QA/BIS 710 Administration Tab

Click the **Administration** tab.

Since the information on this tab is already defined in the **Clinic setup** dialog, you only have to check if the information is correct.

If the information is correct, click **OK**.

7.8.3.4. Save the Measurement Parameters

It is possible to save the measurement setup.

See *10.4.4 Save Measurement Parameters.*

7.8.3.5. Sampling Time (Gain Setting)

1. Click the **I'mRT QA/BIS 710 Meas.** tab. Select **Not scaled** as the scaling mode.
2. In the **Timing** section, select a start value for the sampling time, e.g. 200 ms for I'mRT QA, and 240 ms for BIS 710.
3. Click **OK** to close the dialog.
4. Measure the background. See *7.8.3.6 Perform Background Compensation*.
5. Select the menu command ***Measure:Single Shot***.
6. Start the accelerator.
7. Click the I'mRT QA/BIS toolbar button **Start/Stop**.
8. After completed measurement the resulting image is displayed.
9. Stop the accelerator.
10. Check with the cursor, within the ROI (right-click command ***ROI***), or just by looking at the image, that the maximum dose is around 100%. To view the dose legend, right-click and choose ***Show Legend***.
    If the maximum signal is much higher than 100%, decrease the sampling time.
    If the maximum signal is much less than 100%, increase the sampling time.
    Redo the measurement to check if the signal level is now about 100%.

NOTICE

IMPORTANT NOTICE

HIGH MAXIMUM VALUE AT SHORT SAMPLING TIME

In some cases (very high dose rate, short SSD) the maximum value is much higher than 100%, even if the shortest sampling time is selected. In this case select ***Measure:Edit Parameters*** and clear **Amplification** under the **I'mRT QA Meas.** tab (applicable for I'mRT QA only).

7.8.3.6. Perform Background Compensation

NOTICE

IMPORTANT NOTICE

TURN OFF THE ACCELERATOR

During background definition the accelerator must be turned off.

The background procedure performs a measurement of the signal level on a “dark” image. The defined value will be subtracted from all calculations and presentations.

1. Ensure that the measurement scintillator is inserted. No light may enter the image device!
2. Click the toolbar button **Background**. The measurement starts.

The button remains dimmed until the measurement is completed.

NOTICE

IMPORTANT NOTICE

SAMPLING TIME AND NUMBER OF SAMPLES

Background becomes invalid if the sampling time is changed, or if the number of samples used for background compensation is less than the number of samples used for the measurement.

7.8.4. Measurement

When I'mRT QA/BIS has been selected as measurement equipment, the toolbar and menu options will be expanded.

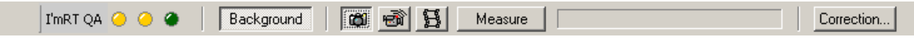

7.8.4.1. Select Measurement Mode

Click the appropriate toolbar button:

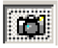 for Single shot mode

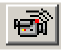 for On-line measurement mode

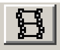 for Movie mode

Depending on the measurement situation, different measurement modes are available for best results:

7.8.4.1.1. Single Shot Mode

In this mode, one single image is composed of a set of single frames (each one measured with the selected measuring time). The composed image contains the average of the selected number of samples.

If background compensation is activated, the background is subtracted from each individual frame.

7.8.4.1.2. Movie Mode

In this mode, a given number of movie pictures (single shots, as described above) are acquired. Each picture, as well as one integrated image, are saved. To ensure acquisition without gaps, the measured data is not displayed on-line.

If background compensation is activated, the background is subtracted from each individual frame.

Use this measurement mode e.g. to analyze dynamic wedges, or MLC fields.

7.8.4.1.3. On-line Mode

In this mode, single shots, as described above, are measured continuously and displayed on-line. Only the last five images are saved.

If background compensation is activated, the background is subtracted from each individual frame.

Use this measurement mode e.g. to trim the field parameters of the accelerator. By looking at the inline and crossline profiles, it is easy to check symmetry and flatness parameters in both orientations simultaneously.

7.8.4.2. Start the Measurement

7.8.4.2.1. Single Shot Mode

Click the toolbar button **Measure**.

After measurement is completed, the resulting image is displayed.

7.8.4.2.2. On-line Mode

Click the toolbar button **Start On-line**.

The images are displayed during the measurement.

When the treatment is finished, press **Stop On-line** to stop the measurement.

The images are not saved.

7.8.4.2.3. Movie Mode

Click the toolbar button **Start Movie**. Start the treatment as planned.

When the pre-defined time has elapsed, the measurement stops. If you want to stop the measurement before the total time has elapsed, use the **Stop Movie** button

After measurement is completed, the resulting image is displayed.

To check single movie pictures, or to run the movie, open the **Field lis**t dialog by selecting ***View:Field list***.

7.9. Setup for Film Control Panel

The handling of films is carried out in the **Film Control Panel**, which contains three tabs: **Scan**, **Calibration**, and **Registration**.

- The **Scan** tab contains the functions for scanning.
- The **Calibration** tab contains functions for calibration of scanners and films, and for saving and loading of calibration data.
- The **Registration** tab provides functions for modification of data.

To open the **Film Control Panel**, a scanner and a film type must first be selected in ***Equipment:Select Clinic and Device***.

7.9.1. Views and Panels

7.9.1.1. Views

The views are designed as a set of panels, where groups of information can be expanded or collapsed. In some cases there may not be room for all information to be displayed in one view. Use the scroll bar to move between the different parts of the panel.

To hide a panel, click the pin button 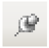 . An icon with the name of the panel will be displayed. To show the panel, move the mouse cursor to the icon. The hidden panel will be temporarily displayed.

Click the pin button 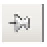 to restore the panel view.

7.9.1.2. Task Panel

Displays commands and data related to the functions available for calibration of scanners and films, scanning, and film display and modification.

7.9.1.3. Info Panel

Contains data and functions related to a scanned film, or to an imported image.

7.9.1.4. Display Panel

Displays the selected image.

7.9.1.5. Film List

When films have been scanned, or imported, thumbnails of the images will be displayed in the **Film List**.

7.9.2. Menus

7.9.2.1. Main Menus

The following main menus are available in the **Film Control Panel**:

Menu	Functions
File	Connect a scanner<br>Open, save, and send an image file<br>Exit the program
Edit	Edit the **Film List**<br>Open the **Options** dialog
View	***Zoom*** functions<br>***Show cursor***<br>***Show out of calibration range values***<br>***Change Palette***
Help	Open the **Help** file<br>Open the **About** box

For details, refer to the **Help** file (also available by pressing <F1>).

7.9.2.1.1. Context Menus

Right-click in the image or the **Display Panel** to display a context menu. The menu contains commands for the zooming function, the cursor function, for indication of values out of the calibration range, and for change of the palette.

7.9.2.1.2. Edit Menu

Right-click in the **Film List** to display ***Edit*** as a context menu. ***Options*** is not included in the context menu. For details, see *7.9.7 Display and Modification of Images*.

7.9.2.1.3. View Menu

Right-click in the **Display Panel** to display ***View*** as a context menu.

7.9.3. Scanning

7.9.3.1. Supported Scanners

The following scanner types are supported:

- Vidar
- LumiScan
- External scanners (not connected to OmniPro I'mRT), e.g TWAIN, are supported via .tiff and .dcm file import. The films are scanned via TWAIN interface and an external scanner software. See *7.9.7.13 Load Image Data*.

7.9.3.2. Select Scanner and Film

1. Open the **Select Clinic and Device** dialog from the ***Measure*** or ***Equipment*** menu.
2. Select **Film scanner** in the **Type** drop-down list box.
3. Select the model name of the device in **Name**. Click **OK**.
4. Select ***Measure:Edit parameters***. The **Measurement Parameters** dialog opens.

NOTICE

IMPORTANT NOTICE

COPY PARAMETERS

You can also copy parameters from the active field, or load them from another measurement. Use ***Measure:Copy Parameters*** or ***Measure:Load Parameters***, respectively.

5. Select the **Film scanner** tab. Select film and the body phantom. Enter the **Film Offset** value.

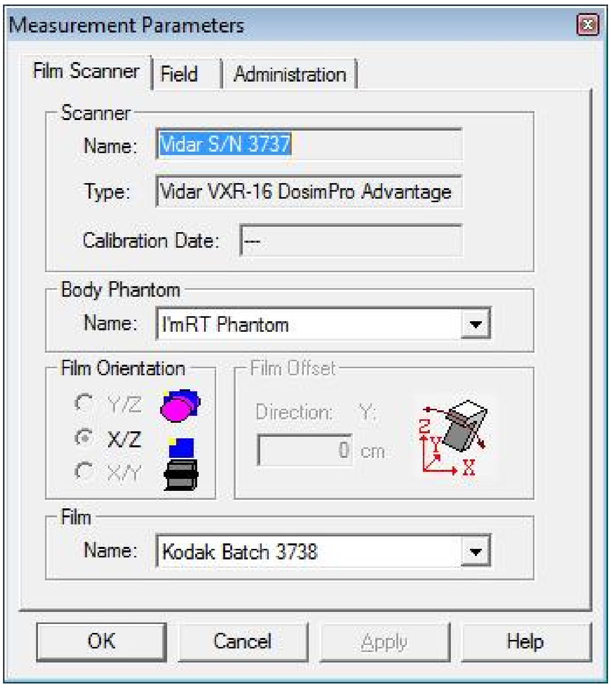

WARNING

WARNING

FILM OFFSET

Film and film-envelope produce an offset. When inserting films in a body phantom, other film planes or slices are shifted, due to the thickness of the films and their envelopes. When defining the offset information in the film setup, take into account the additional offset produced by the other films.

6. Edit the field information under the **Field** tab.
7. Click **OK**.

It is possible to save the measurement setup.

See *10.4.4 Save Measurement Parameters.*

7.9.3.3. Prepare Scanning

1. Open the **Film Control Panel** by selecting ***Measure:Scan Film***, or click the button in the main toolbar. Select the **Scan** tab.
2. Select ***Connect to*** in the ***File*** menu to connect the scanner. The available options are Vidar and LumiScan scanners.

   Data for the selected scanner will be displayed in the **Info Panel**.

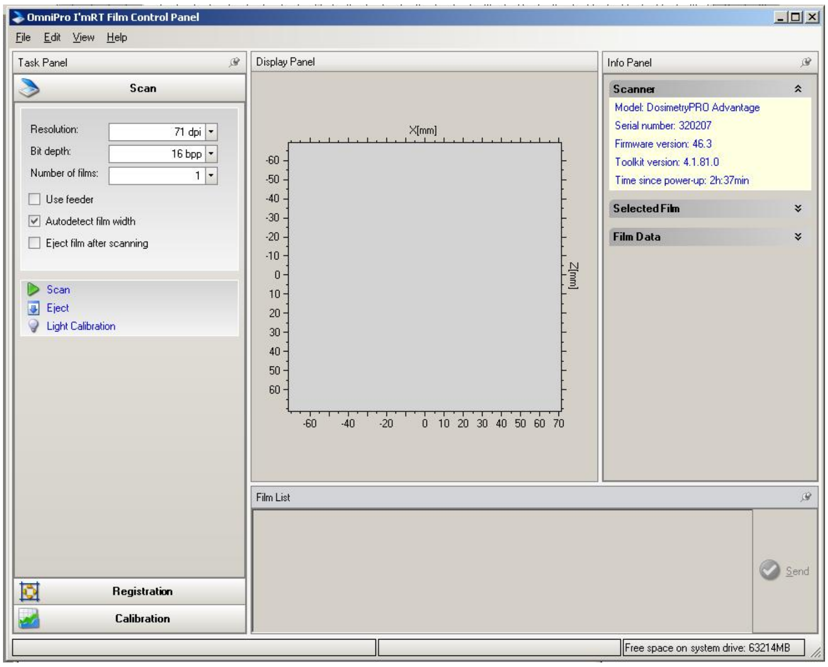

   The resolution and bit depth options available for the connected scanner type are displayed.

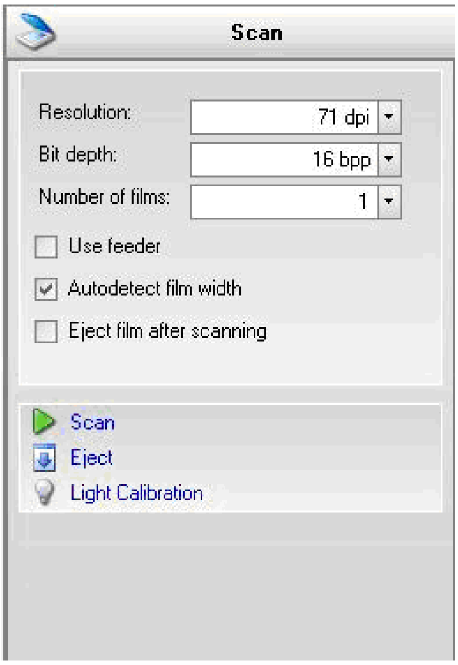

3. Define **Resolution**, **Bit depth**, and **Number of films**.

NOTICE

IMPORTANT NOTICE

RESOLUTION

In most cases the lowest resolution is sufficient, and should be used to minimize the amount of data.

7.9.3.3.1. Options for Vidar Scanners:

Use feeder: Option for Vidar scanners with multi film feeder. Mark the checkbox, and enter the number of films to be scanned, if you want to scan more than one film.

Autodetect film width: Option for all Vidar scanners. If this option is selected, the scanner will detect the width of the inserted film before it starts to scan, and will stop the scanning process when the last line is reached.

NOTICE

IMPORTANT NOTICE

AUTODETECT FILM WIDTH

The displayed progress bar will not be accurate if **Autodetect film width** is selected, since the height of the film cannot be estimated before the start of the scan.

NOTICE

IMPORTANT NOTICE

AUTODETECT FILM WIDTH

Do not select **Autodetect film width** if part of the film is not irradiated.

The selection is persistent between sessions.

If the **Autodetect film width** option is not selected, the entire scan area will be scanned.

Eject film after scanning: If selected, the film will be ejected after the scan. If not selected, the film will be automatically re-positioned for scanning.

Eject: Click to eject the film.

Light calibration: Option for all Vidar scanners. Click this option to perform a light calibration. It takes 1 - 2 minutes to perform the calibration. A progress bar is displayed.

7.9.3.3.2. Option for LumiScan Scanners:

Use film present sensor: Mark the checkbox to activate the function that handles information from the scanner's film detector.

NOTICE

IMPORTANT NOTICE

FILM PRESENCE DETECTOR

If the scanner model used does not have a film presence detector, the I'mRT film present sensor will not receive the information that the film is inserted, and the message **Film Not Inserted** will appear. This applies for a few LumiScan scanner models. If this happens, uncheck the **Use film present sensor** checkbox.

7.9.3.4. Scanning Procedure

Insert a film (or all films to be scanned, if you use a film feeder).

Click **Scan** to start the scan. The option will change to **Cancel**. Click the button to stop before the scanning is finished.

Eject: Click this option to eject the film manually.

The image will be displayed in the **Display Panel**. Thumbnails of all scanned films are displayed in the **Film List**. The label of the film can be changed. See 7.9.7.7 *Options*.

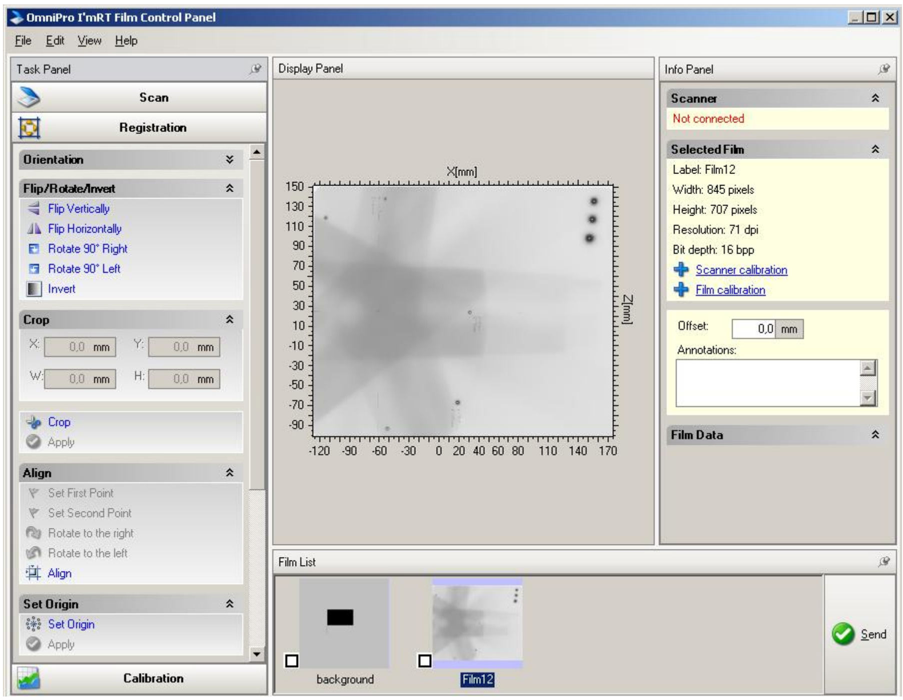

A zoom function and a cursor can be applied on the image, and the palette can be changed. Right-click in the image area and select a menu item in the context menu. See *7.9.7.8 Zoom*.

Annotations: Enter additional information and comments (optional).

Send: Click to send data to the OmniPro I'mRT workspace.

7.9.3.5. Scanning Workflow, Vidar and LumiScan

Below is a summary of the workflow for scanning a film with Vidar or LumiScan scanners. For details, see *7.9.3 Scanning* and 7.9.7 *Display and Modification of Images*.

1. Define film and body phantom in the **Edit Parameter** dialog.
2. Select ***Scan film*** in the ***Measurement*** menu to open the **Film Control Panel**. Select the **Scan** tab.
3. The film orientation is transferred to the **Film Control Panel**, and displayed in the **Task Panel**. The data will be applied to the scanned image.
4. Define the options for the scanner.
5. Insert the film and click the **Scan** button. The image will be displayed in the **Display Panel**.
6. Select the **Registration** tab. Rotate, align and crop the image, if necessary.
7. Set origin.
8. Option: Save the data in .TIF format (***File:Save***). Calibration data and origin definition data are saved with the image.
9. Select the images to be sent. Click the **Send** button in the **Film List**.

7.9.3.6. Scanning Workflow, External Scanners

Below is a summary of the workflow for scanning a film imported from an external scanner. For details, please refer to *7.9.3 Scanning* and 7.9.7 *Display and Modification of Images*.

1. Scan the films in the external scanner and save it as .TIF file
2. Define film and body phantom in the **Edit Parameter** dialog.
3. Select ***Scan film*** in the ***Measurement*** menu to open the **Film Control Panel**. Select the **Scan** tab.
4. The film orientation is transferred to the **Film Control Panel**, and displayed in the **Task Panel**. The data will be applied to the scanned image.
5. Open the scanned film file. The image will be displayed in the **Display Panel**.
6. Select the **Registration** tab. Rotate, align and crop the image, if necessary.
7. Set origin.
8. In the **Task Panel**, check that the proper scanner and film calibrations are loaded. Apply the current calibration to the image.
9. Option: Save the data in .TIF format (**File:Save**) Calibration data and origin definition data are saved with the image.
10. Select the images to be sent. Click the **Send** button in the **Film List**.

7.9.4. Scanner Artifacts Correction

This method has been developed to correct scanned films from possible scanner artefacts created by CCD scanners (using a laser light source) to digitize light-scattering exposed films (Gafchromic). These artefacts are caused by light scattering from the film during the scanning. The correction is done in the following steps:

1. Select the **Scan** tab.
2. Take a film form the batch where the corrected films will be from.
3. Scan this un-irradiated film in the same orientation as the films that shall be corrected.
4. Rename the image as “background”.
5. Expand **Scanner artifacts** in the **Task Panel**.

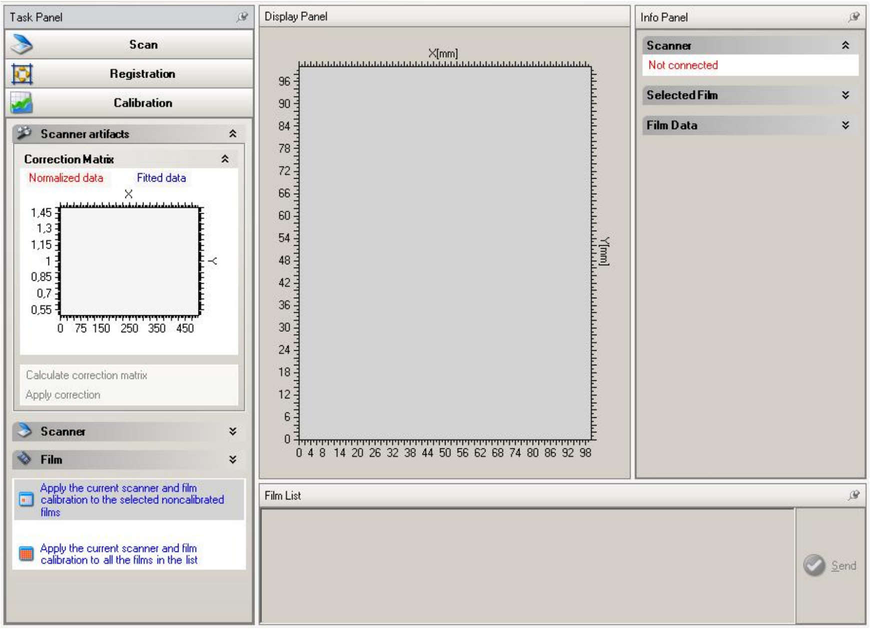

6. Click **Calculate correction matrix**. Data from the “background” image is displayed.

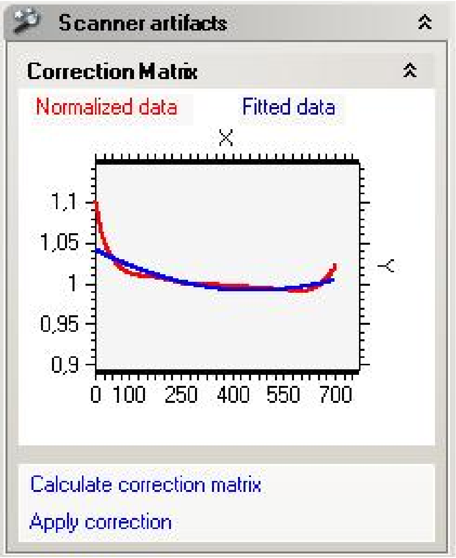

7. Apply the scanner artifacts correction to that film using for the scanner and film calibration.

NOTICE

IMPORTANT NOTICE

Apply the calculated correction matrix always before performing the scanner/film calibration.

8. Perform the calibration as described in *7.9.5 Scanner Calibration* and *7.9.6 Film Calibration.*

NOTICE

IMPORTANT NOTICE

ALWAYS USE THE CALCULATED CORRECTION FOR THE CALIBRATION FILM

Apply the calculated correction matrix always to the films used for the calibration procedure. The steps should be the follows:

- Scan film.
- Correct the film with the correction matrix.
- Calibrate the film.

9. For applying the correction matrix open an image (or scan a film), and select it in the **Film List** by checkmarking.

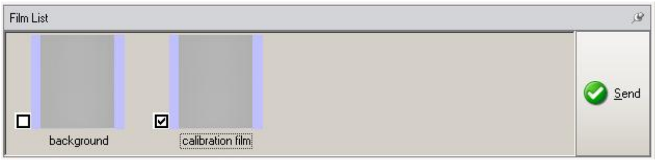

10. Click **Apply correction**. The background data will be subtracted from the image. The name of the image will appear in green in the **Film List**.

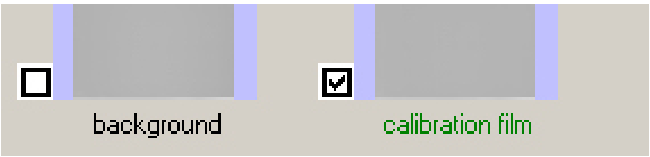

11. Save the corrected image.

CAUTION

CAUTION

DO NOT APPLY CORRECTION TO A SAVED FILE

The information that the image is corrected is not saved. To prevent that an image will be corrected more than once, do not apply this function to a saved file.

7.9.5. Scanner Calibration

When measuring with films, you are, as with relative dosimetry in water or air, interested in relative dose, not in relative signal or relative optical density. Without calibration, however, you would only get relative signal. It is therefore necessary to calibrate the film scanner and the film.

Select the **Calibration** tab.

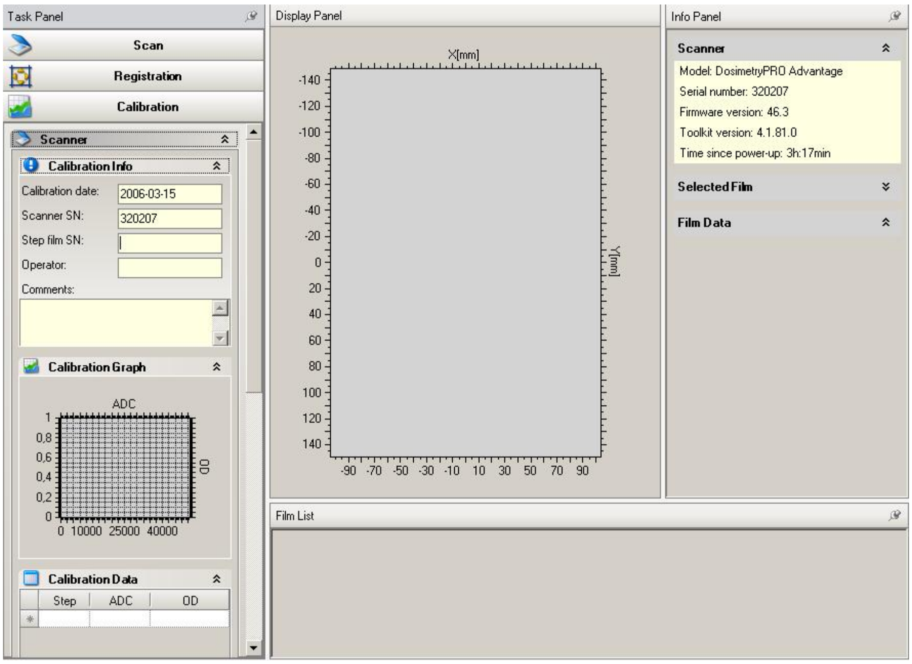

7.9.5.1. Signal to Optical Density

The Optical Density (OD) scanner calibration defines the relationship between the scanner signal and Optical Density. The step film delivered with the scanner can be used for calibration.

7.9.5.2. Prepare Calibration

7.9.5.2.1. Vidar and LumiScan Scanners

1. Switch on the film scanner to warm-up for at least 15 minutes before the calibration starts. (See the hardware manual of the scanner for correct warm-up time.)
2. Insert the calibration film in the film magazine of the scanner. Orient the film so that the step with the lowest density is scanned first.
3. Select the **Scan** tab, and prepare the scan in the **Task Panel** (see *7.9.3.3 Prepare Scanning*).
4. Define **Resolution** (the lowest resolution is sufficient for calibration) and **Bit depth**. The default value for **Number of films** is 1.
5. Select scanner options. Click **Scan** to perform the scanning.

NOTICE

IMPORTANT NOTICE

OLDER TYPES OF VIDAR SCANNERS

For older types of Vidar scanners it is important that the film guides are closed as much as possible in order to avoid ambient light disturbing the calibration process. If the step film is narrower than the film guide opening, it needs to be attached to a larger sheet of film, or similar, which is not transparent to the light from the scanner.

7.9.5.2.2. External Scanners

Scan the calibration film according to the instructions for the scanner. Save the image in .TIF format. Open the file in the **Film Control Panel**.

7.9.5.3. Calibration Procedure

The scanned (Vidar and LumiScan scanners) or imported (external scanner) image will be displayed in the **Display Panel**.

NOTICE

IMPORTANT NOTICE

SCANNER ARTIFACTS CORRECTION

If a correction for scanner artifacts will be applied – this needs to be done before the calibration. Therefore please refer to chapter *7.9.4 Scanner Artifacts Correction*.

1. Select **New calibration** in the **Task Panel**:

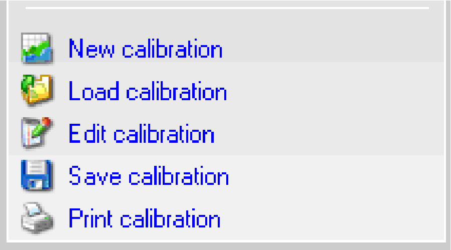

   Current date is automatically displayed in **Calibration Info**.

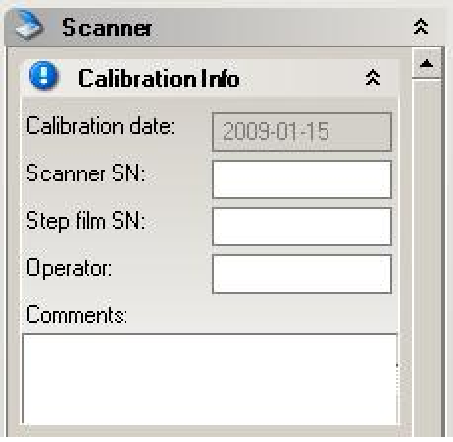

2. Enter the serial number of the step film, and the name of the operator.

   Option: Enter additional information in the **Comments** field.

3. Define the step film properties in **Options**:

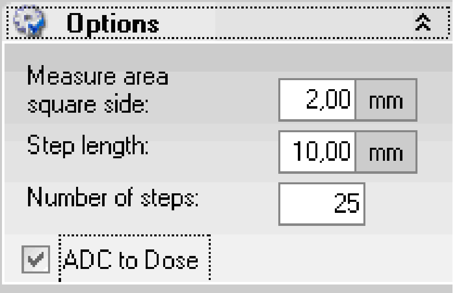

4. Enter the side length of the square to be used for ADC mean value calculation, in **Measure area square side**.

5. Enter the Number of steps.
6. Enter the **Step length**.

   ADC to Dose: See *7.9.5.4 ADC to Dose*.
7. Right-click in the image, and select ***Show cursor*** in the context menu.
8. Position the cursor in the area with the lowest OD value in the step film image. Ensure that the cursor is positioned at half the step length from the border to next step (i.e. if the step length is 10 mm, position the cursor 5 mm below the border).

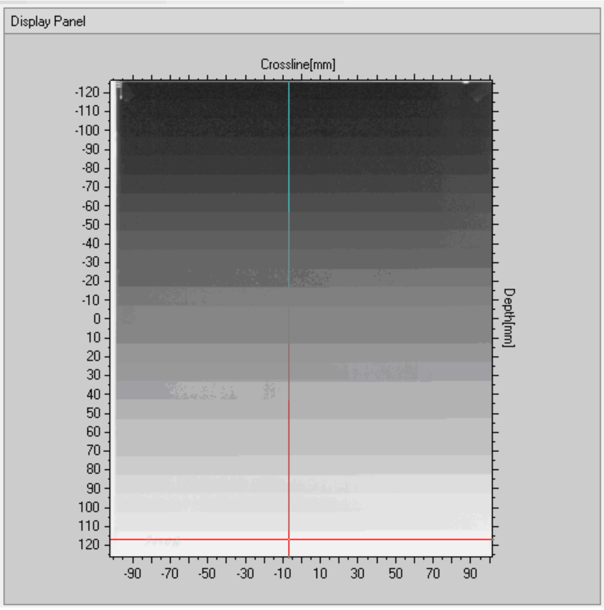

9. Select **Load steps**. All steps are automatically entered in the **ADC** column in the **Calibration Data** table.

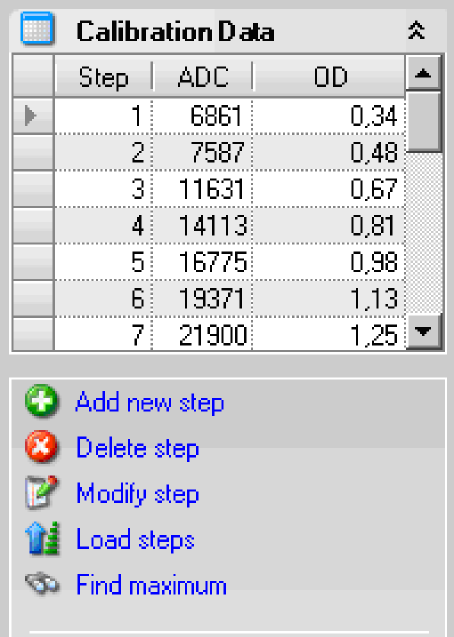

Step	ADC	OD
1	6861	0,34
2	7587	0,48
3	11631	0,67
4	14113	0,81
5	16775	0,98
6	19371	1,13
7	21900	1,25

If the step length in the step film varies, enter one step at a time. Position the cursor in a field, and select **Add new step**. Repeat for each step. Hold the <Shift> key to display the measuring area.

10. Enter the density value (from the density table, delivered with the step film) for each field in the **OD** column. Data will also be graphically displayed in **Calibration Graph**. Double-click in the graph to enlarge the view. Double-click again to restore the original scale.

7.9.5.3.1. Find Maximum

The function Find Maximum is useful to get rid of the "out of calibration values". Click the button to find the maximum ADC value. The cursor will move to the pixel with highest ADC value.

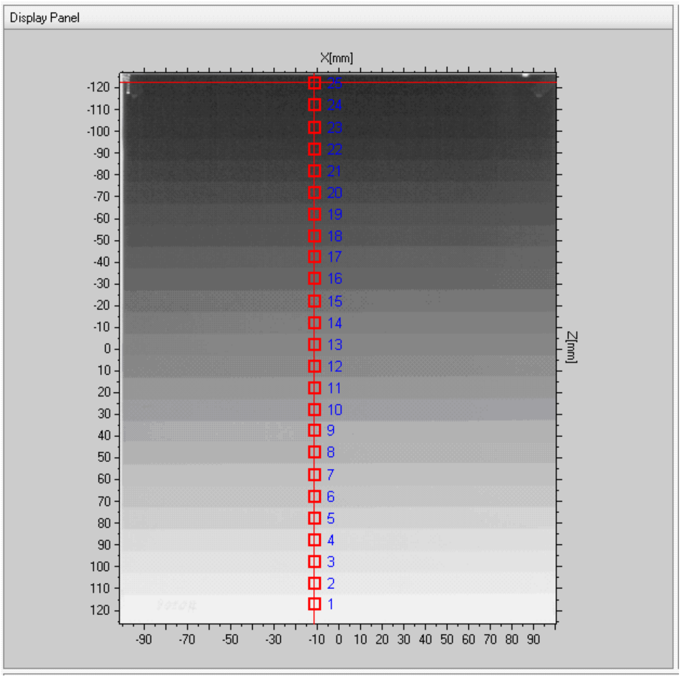

1. Click **Add step** to add the value to the calibration table, and enter an appropriate density value.
2. Calibration points can be deleted or modified. Select **Delete step** or **Modify step**.
3. Select **Save calibration** to save calibration data in the **Film Control Panel**. Data is saved as a .cal file. To save data in the OmniPro I'mRT database, see *7.9.5.5 Save Calibration Data*.
4. Select **Print calibration** to print the calibration table and graph.

NOTICE

IMPORTANT NOTICE

PREVIOUSLY SAVED FILMS

Re-calibration will not change the measurement values on previously scanned and saved films.

7.9.5.4. ADC to Dose

It is possible to perform a direct signal to dose calibration, when performing a scanner calibration. The calibration is only valid with this specific scanner.

⚠ WARNING

WARNING

SAVE ADC TO DOSE IN THE FILM CONTROL PANEL

ADC to dose calibration must be saved in the **Film Control Panel** (**Save calibration)**, and loaded (**Load calibration**) before use.

It is strongly recommended to calibrate both the scanner and the film.

7.9.5.5. Save Calibration Data

Data is automatically saved in the OmniPro I'mRT main application when the dialog is closed. It is also possible to save data to the main application manually, by clicking the **Send** button in the **Film List**.

Option: Save calibration data in the **Film Control Panel** by clicking **Save calibration**.

NOTICE

IMPORTANT NOTICE

SAVING DATA IN THE FILM CONTROL PANEL

Saving in the **Film Control Panel** must be carried out before data is sent to the main application.

Data saved in the **Film Control Panel** is saved as a .cal file, in xml format. Calibration data, including date, scanner and step film serial numbers, operator, and comments are saved.

Calibration data is automatically saved in the OmniPro I'mRT database when the **Film Control Panel** is closed. Data can also be saved by clicking the **Send** button in the **Film List**. Select **Overwrite** in the dialog to save the calibration for the current film.

7.9.5.6. Load Calibration Data

Click **Load Calibration** to import calibration files.

7.9.5.7. Scanner Calibration Templates

A scanner calibration can be saved as a template. The positions of the calibration points and the entered OD values are saved in the template, and will be used when the template is loaded for a new calibration.

7.9.5.7.1. Save Template

1. Define the calibration point, and enter the corresponding OD values. For details about calibration, see *7.9.5 Scanner Calibration* and *7.9.6 Film Calibration*.
2. Click **Save template** to save the calibration template as a .tem file.

7.9.5.7.2. Load Template

The Load template function is similar to the Load steps function.

NOTICE

IMPORTANT NOTICE

APPLY SIMILAR CONDITIONS

The calibration films, on which the template is applied, must be scanned under approximately the same conditions (orientation and size), otherwise the values will be read from the wrong positions.

1. Open (or scan) the calibration film. Adjust it in the same way as the film in the calibration template that is going to be used.
2. Click **Load Template**, and select a template file (.tem).

   When the template file is loaded, the position and OD value for each point is loaded, and the ADC value is read from the image. A new calibration table is created.
3. Save the calibration.

7.9.5.8. Scanner Calibration Workflow, Vidar and LumiScan Scanners

Below is a summary of the workflow for Vidar or LumiScan scanner calibration. For details, please refer to *7.9.5.2 Prepare Calibration* and *7.9.5.3 Calibration Procedure*.

1. Switch on the scanner to warm up for at least 15 minutes before the calibration.
2. Select the **Scan** tab in the **Film Control Panel**. Define the options for the scanner (*7.9.3.3.1 Options for Vidar Scanners* or *7.9.3.3.2 Option for LumiScan Scanners*).
3. Define **Resolution** (the lowest resolution is sufficient for calibration) and **Bit depth**. Set the value for **Number of films** to 1.
4. Vidar scanners only: Click the **Light Calibration** button (*7.9.3.3.1 Options for Vidar Scanners*).
5. Insert the calibration step film. Orient the film so that the step with the lowest density is scanned first.
6. Click the **Scan** button to perform the scanning. The scanned image will be displayed in the **Display Panel**.
7. Select the **Calibration** tab, and expand **Scanner** in the **Task Panel**.
8. Select **New calibration**.
9. The current date is automatically displayed in **Calibration Info**. Enter the serial number of the scanner and the step film, and the name of the operator. Option: Enter additional comments in the **Comments** field.
10. Option: If a calibration template is already saved (*7.9.5.7 Scanner Calibration Templates),* load the template by clicking **Load template** in the **Task Panel**. The position and OD values for each point are loaded, and the ADC values are read from the image. If this option is used, continue by saving calibration data.
11. Define the step film properties in **Options** in the **Task Panel** (7.9.5.3 Calibration Procedure).
12. Right-click in the image, and select ***Show cursor*** in the context menu.
13. Position the cursor in the area with the lowest OD value in the step film image. The cursor must be positioned at half the step length form the border to the next step.
14. Click **Load steps**. All steps are automatically entered in the **Calibration Data** table. If the step length in the images varies, use **Add new step** to enter one step at a time (*7.9.5.3 Calibration Procedure*).
15. Enter the density values, delivered with the step film, in the OD column in **Calibration data**.
16. Option: Click **Find maximum** to find the point with the highest ADC value. Enter the value with **Add new step** and enter an appropriate OD value.
17. Option: Save the calibration template in the **Film Control Panel** by clicking **Save template**.
18. Option: Save calibration data in the **Film Control Panel** by clicking **Save calibration** *(7.9.5.5 Save Calibration Data*).
19. Calibration data is automatically saved in the OmniPro I'mRT database when the **Film Control Panel** is closed. Data can also be saved by clicking the **Send** button in the **Film List**.

7.9.5.9. Scanner Calibration Workflow, External Scanners

Below is a summary of the workflow for external scanner calibration. For details, please refer to *7.9.5.2 Prepare Calibration* and *7.9.5.3 Calibration Procedure*.

1. Scan the calibration film according to the instructions for the external scanner
2. Save the image in .tif format.
3. Open the file in the **Film Control Panel**. The scanned image will be displayed in the **Display Panel**.
4. Select **New calibration**.
5. The current date is automatically displayed in **Calibration Info**. Enter the serial number of the scanner and the step film, and the name of the operator. Option: Enter additional comments in the **Comments** field.
6. Option: If a calibration template is already saved (*7.9.5.7 Calibration Templates)*, load the template by clicking **Load template** in the **Task Panel**. The position and OD values for each point are loaded, and the ADC values are read from the image. If this option is used, continue by saving the calibration data.
7. Define the step film properties in **Options** in the **Task Panel** (*7.9.5.3 Calibration Procedure*).
8. Right-click in the image, and select ***Show cursor*** in the context menu.
9. Position the cursor in the area with the lowest OD value in the step film image. The cursor must be positioned at half the step length form the border to the next step.
10. Click **Load steps**. All steps are automatically entered in the **Calibration Data** table. If the step length in the images varies, use **Add new step** to enter one step at a time (*7.9.5.3 Calibration Procedure*).
11. Enter the density values, delivered with the step film, in the **OD** column in **Calibration data**.
12. Option: Click **Find maximum** to find the point with the highest ADC value. Enter the value with **Add new step**.
13. Option: Save the calibration template in the **Film Control Panel** by clicking **Save template**.
14. Option: Save calibration data in the **Film Control Panel** by clicking **Save calibration** (*7.9.5.5 Save Calibration data*).
15. Calibration data is automatically saved in the OmniPro I'mRT database when the **Film Control Panel** is closed. Data can also be saved by clicking the **Send** button in the **Film List**.

7.9.6. Film Calibration

Film calibration defines the conversion factor between the Optical Density (OD) and absorbed dose. The calibration is dependent upon the type of film in use and how it was developed. The calibration is stored with the selected film type in the measurement setup. It is required that each film type has an individual calibration. For greater accuracy every film batch should be calibrated individually.

⚠ WARNING

WARNING

SENSITIVITY MAY VARY FROM FILM TO FILM

The sensitivity of radiographic films is strongly dependent on parameters like type, age, batch, or storage conditions. Perform a film calibration when using films where one or more of the above parameters differ from the film used for the current film calibration.

To perform the calibration, you can choose to use multiple films exposed to various dose levels or a single film exposed to multiple various dose levels, covering the range of interest (e.g. 0.0 to 3 Gy).

7.9.6.1. Fog Determination

For fog determination, you can choose to scan an unexposed film, or use an unexposed part of a film with multiple exposed dose levels. Enter the dose value 0 to the corresponding optical density in the calibration table.

7.9.6.2. Optical Density to Dose Determination

⚠ WARNING

WARNING

OD VALUES OUTSIDE FILM SCANNER CALIBRATION RANGE

If a film is calibrated or measured outside the film scanner calibration range, the measurement values can be incorrect. Never calibrate or measure a film outside the OD range for which the film scanner has been calibrated.

⚠ WARNING

WARNING

OD VALUES OUTSIDE FILM SCANNER CALIBRATION RANGE

If a film is measured outside the film calibration range, the measurement values can be incorrect. Never measure a film outside the dose range for which the film has been calibrated.

Mark the points on the scanned film, where you know the dose. The points will be added in the film calibration table with the optical density value.

7.9.6.3. Prepare Calibration

1. Place a single or multiple exposed developed film into the film magazine of the scanner.
2. Open the **Scan** tab in the **Film Control Panel**.
3. Define **Resolution** (the lowest resolution is sufficient), **Bit depth**, and **Number of films**, and select scanner options (see *7.9.3.3 Prepare Scanning*).
4. Click **Scan** to perform the scanning.

7.9.6.4. Calibration Procedure

NOTICE

IMPORTANT NOTICE

SCANNER ARTIFACTS CORRECTION

If a correction for scanner artifacts will be applied – this needs to be done before the calibration. Therefore please refer to chapter *7.9.4 Scanner Artifacts Correction*.

1. Open the **Calibration** tab. The scanned image will be displayed in the **Display Panel**.
2. Select **New calibration** in the **Task Panel**:

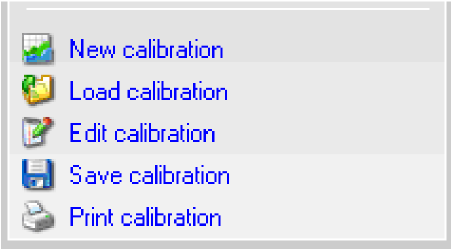

3. Select **Film** in the **Task Panel**.

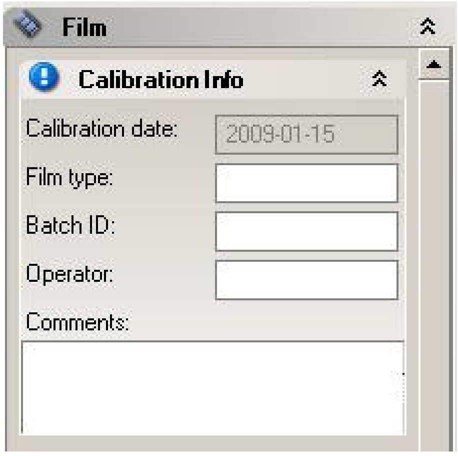

The current date will be displayed automatically.

4. Enter the film type, the batch ID, and the name of the operator.
5. Option: Enter additional information in the **Comments** field.
6. Right-click in the image, and select ***Show cursor*** in the context menu.
7. Position the cursor at the point where you want to define the dose. Hold the <Shift key> to display the measuring area.

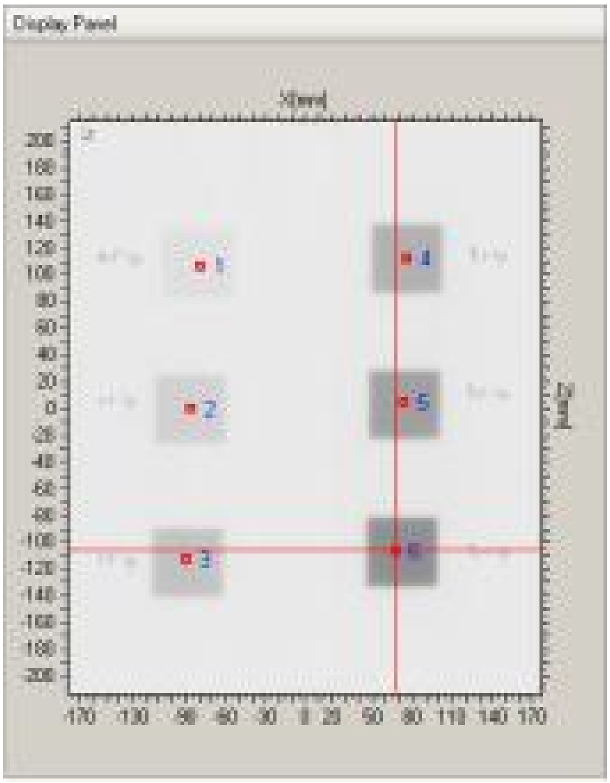

8. Select **Add new step**. The OD value is displayed in the **OD** column in the **Calibration Data** table.
9. Repeat for each point.
10. Enter the dose value for each field in the **Dose** column.

Calibration Data

Step	OD	Dose [Gy]
1	1,51	0
2	16,07	0,5
3	23,64	1
4	31,60	1,5
5	40,95	2
6	50,44	2,5
7	59,92	3

The points can be deleted or modified. Select **Delete step** or **Modify step**.

11. Data will also be graphically displayed in **Calibration Graph**. Double-click in the graph to enlarge the view. Double-click again to restore the original scale.
12. Option: Save calibration data in the **Film Control Panel** by clicking **Save calibration**. See *7.9.6.8 Save Film Calibration Data*.
13. Calibration data is automatically saved in the OmniPro I'mRT database when the **Film Control Panel** is closed. Data can also be saved by clicking the **Send** button in the **Film List**. Select **Overwrite** in the dialog to save the calibration for the current film.
14. Select **Print calibration** to print the calibration table and graph.

NOTICE

IMPORTANT NOTICE

MEASUREMENT POINTS

For a valid calibration you need at least three measurement points.

NOTICE

IMPORTANT NOTICE

RECALIBRATION

Recalibration on a regular basis is recommended, due to variation in film quality, and especially due to variation when developing the film.

NOTICE

IMPORTANT NOTICE

PREVIOUSLY SAVED FILMS

Recalibration will not change the measurement values on previously scanned and saved films.

7.9.6.5. Film Calibration Templates

A film calibration can be saved as a template. The positions of the calibration points and the entered OD values are saved in the template, and will be used when the template is loaded for a new calibration.

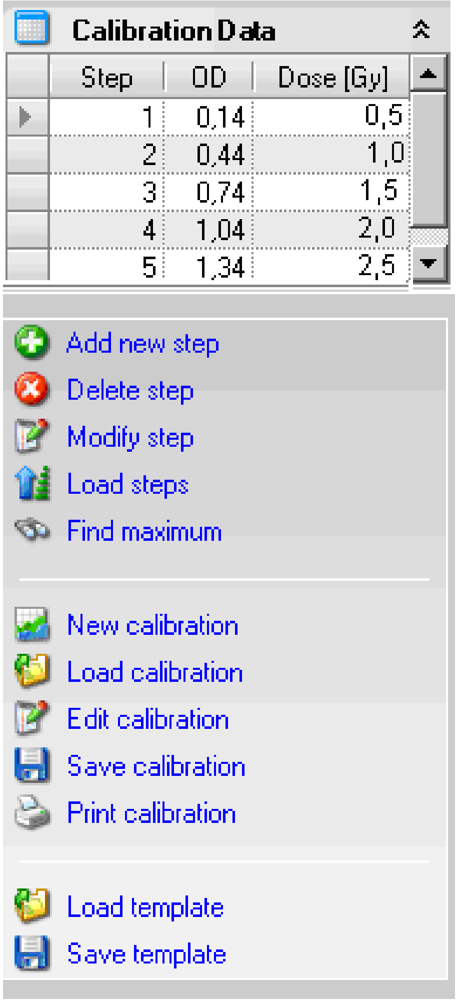

7.9.6.5.1. Save Template

Define the calibration points, and enter the corresponding OD values. Click **Save template** to save the calibration template as a .tem file.

7.9.6.5.2. Load Template

The Load template function is similar to the Load steps function.

NOTICE

IMPORTANT NOTICE

APPLY SIMILAR CONDITIONS

The calibration films, on which the template is applied, must be scanned under approximately the same conditions (orientation and size), otherwise the values will be read from the wrong positions.

Open (or scan) the calibration film. Adjust it in the same way as the film in the calibration template that is going to be used.

Click **Load Template**, and select a template file (.tem).

When the template file is loaded, the position and dose value for each point is loaded, and the OD value is read from the image. A new calibration table is created.

Save the calibration.

7.9.6.6. Film Calibration Workflow, Vidar and LumiScan Scanners

Below is summary of the workflow for film calibration, with Vidar or LumiScan scanners.

1. Open the **Film Control Panel**. Select the **Scan** tab.
2. Define the options for the scanner (*7.9.3.3.1 Options for Vidar Scanners* and *7.9.3.3.2 Options for Lumisys Scanners*).
3. Define **Resolution** (the lowest resolution is sufficient for calibration) and **Bit depth**. Set the value for **Number of films** to 1.
4. Vidar scanners only: Click the **Light Calibration** button (*7.9.3.3.1 Options for Vidar Scanners*).
5. Insert an unexposed film into the scanner, to make the fog determination (*7.9.6.1 Fog Determination*).
6. Select the **Scan** tab, and click the **Scan** button to perform the scanning.
7. Insert a single or multiple exposed developed film into the scanner (*7.9.6.4 Calibration Procedure* and *7.9.6.2 Optical Density to Dose Determination*).
8. Click the **Scan** button to perform the scanning. The scanned images will be displayed in the **Display Panel** and in the **Film List**.
9. Select the **Calibration** tab, and expand **Film** in the **Task Panel**.
10. Select **New calibration**. For recalibration select **Edit calibration**.
11. The current date is automatically displayed in **Calibration Info**. Enter the serial number of the step film, and the name of the operator. Option: Enter additional comments in the **Comments** field.
12. Select the image of the unexposed film. Right-click in the image, and select ***Show cursor*** in the context menu.
13. Position the cursor at the point where you want to define the fog value (no dose).

14. Option: If a calibration template will be used, click **Load template**, and continue by saving the calibration data.
15. If no calibration template is used: Select the image of the exposed film.
16. Position the cursor at the point where you want to define the dose, and click **Add new step**.
17. Repeat for each point.
18. Enter the dose value for each field in the **Dose** column in **Calibration data**. The dose value for the unexposed film shall be set to 0.
19. Option: Save the calibration template in the **Film Control Panel** by clicking **Save template**.
20. Option: Save calibration data in the **Film Control Panel** by clicking **Save calibration** (*7.9.6.8 Save Film Calibration Data*).
21. Calibration data is automatically saved in the OmniPro I'mRT database when the **Film Control Panel** is closed. Data can also be saved by clicking the **Send** button in the **Film List**.
22. Select **Overwrite** in the dialog to save the calibration for the current film.

7.9.6.7. Film Calibration Workflow, External Scanners

Below is a summary of the workflow for film calibration, with an external scanner.

1. Scan an unexposed developed film into the scanner, to make the fog determination (*7.9.6.1 Fog Determination*).
2. Save the image as a .tif file.
3. Scan a single or multiple exposed developed film into the scanner (*7.9.6.4 Calibration Procedure* and *7.9.6.2 Optical Density to Dose Determination*). Save the images as .tif files
4. Open the scanned images in the **Film Control Panel**.
5. Load the proper scanner calibration
6. Apply the scanner calibration to all the dose calibration images
7. Select the **Calibration** tab, and expand **Film** in the **Task Panel**.
8. Select **New calibration**. For recalibration select **Edit calibration**
9. The current date is automatically displayed in **Calibration Info**. Enter the serial number of the step film, and the name of the operator. Option: Enter additional comments in the **Comments** field.
10. Select the image of the unexposed film. Right-click in the image, and select ***Show cursor*** in the context menu.
11. Position the cursor at the point where you want to define the dose.
12. Option: If a calibration template (*7.9.6.5 Film Calibration Templates*) will be used, click **Load template**, and continue by saving the calibration data.
13. Select the image of the exposed film.
14. Position the cursor at the point where you want to define the dose, and click **Add new step**.
15. Repeat for each point.

16. Enter the dose value for each field in the **Dose** column in **Calibration data**. The dose value for the unexposed film shall be set to 0.
17. Option: Save calibration template in the **Film Control Panel** by clicking **Save template**.
18. Option: Save calibration data in the **Film Control Panel** by clicking **Save calibration** (*7.9.6.8 Save Film Calibration Data*).
19. Calibration data is automatically saved in the OmniPro I'mRT database when the **Film Control Panel** is closed. Data can also be saved by clicking the **Send** button in the **Film List** (*7.9.6.8 Save Film Calibration Data*).
20. Select **Overwrite** in the dialog to save the calibration is the current film.

7.9.6.8. Save Film Calibration Data

7.9.6.8.1. Save Calibration Data in the Film Control Panel

Click **Save calibration** to save data in the **Film Control Panel**. The data will not be saved in the OmniPro I'mRT database. Data is saved as a .cal file.

7.9.6.8.2. Save calibration data in the OmniPro I'mRT database

Data is automatically saved in the database of the main application when the **Film Control Panel** is closed.

⚠ WARNING

WARNING

SAVE ADC TO DOSE IN THE FILM CONTROL PANEL

ADC to dose calibration must be saved in the **Film Control Panel** (**Save calibration)**, and loaded (**Load calibration**) before use.

It is also possible to save data by sending it to the I'mRT Workspace:

1. Select the calibration image and click **Send** in the **Film List**. The **Importing data…** dialog opens.

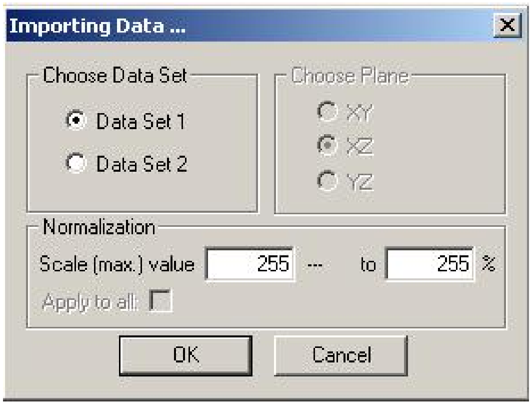

2. Select data set, and click **OK**. The **Calibration Data** dialog opens.

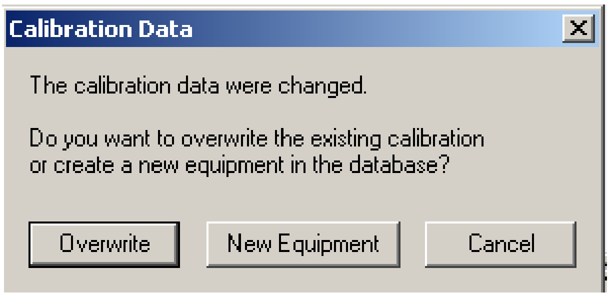

3. Select **Overwrite**.

7.9.6.8.3. Load Calibration Data

Click **Load calibration data**, and select the calibration to be used.

7.9.6.9. Print Calibration Data

Click **Print calibration** to print the currently loaded calibration. A **Print Preview** dialog opens, which displays the print-out. Click the **Print** button to print.

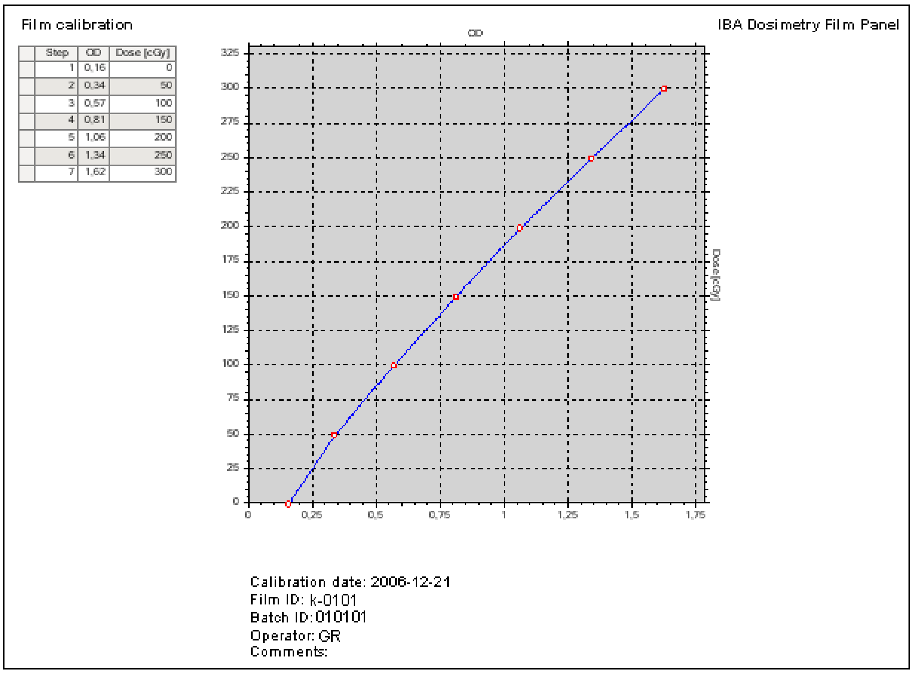

Step	OD	Dose [cGy]
1	0,16	0
2	0,34	50
3	0,57	100
4	0,81	150
5	1,06	200
6	1,34	250
7	1,62	300

7.9.6.10. Apply Current Calibration

7.9.6.10.1. Apply Current Calibration to a Non-calibrated Film

The currently loaded calibration can be applied to un-calibrated film. Select the **Calibration** tab. Select film(s).

Click the button **Apply the current scanner and film calibration…** in the **Task Panel**.

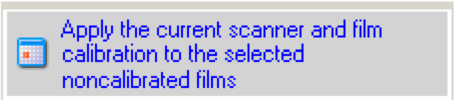

The current calibration data will be applied, and displayed in the **Info Panel**.

7.9.6.10.2. Apply Current Calibration to All Films in the Film List

The current calibration can be applied simultaneously to all films, calibrated as well as un-calibrated, that are displayed in the **Film List**.

NOTICE

IMPORTANT NOTICE

CHECK LOADED CALIBRATION

When the **Film Control Panel** is opened, the calibrations for the selected scanner and film will be displayed in the **Task Panel**. Ensure that the correct calibration is loaded before applying it to the film.

1. Load the calibration that shall be used.
2. Click the button **Apply the current scanner and film calibration to all films** in the list in the **Task Panel**:

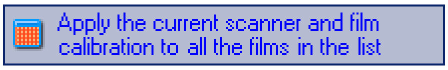

The current calibration data will be applied, and displayed in the **Info Panel**.

⚠ WARNING

WARNING

RISK OF UNPREDICTABLE RESULTS

Applying a different calibration table on an already calibrated film can produce unpredicted results.

7.9.6.11. Replace Calibration Data in a Calibrated Film

The calibration data embedded in a film can be replaced with the current calibration. Select the **Calibration** tab.

Select the film to which the current calibration data shall be applied, by marking the checkbox in the **Film List**. Click the **Scanner** or/and the **Film Calibration** button in the **Info Panel**. The embedded calibration data is displayed. Click the button **Apply selected global calibration**.

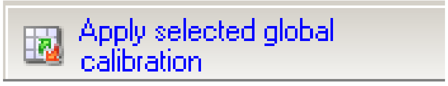

The current calibration data will be applied, and displayed in the **Info Panel**.

⚠ WARNING

WARNING

RISK OF UNPREDICTABLE RESULTS

Applying a different calibration table on an already calibrated film can produce unpredicted results.

7.9.7. Display and Modification of Images

To modify an image, and to change the display, select the **Registration** tab.

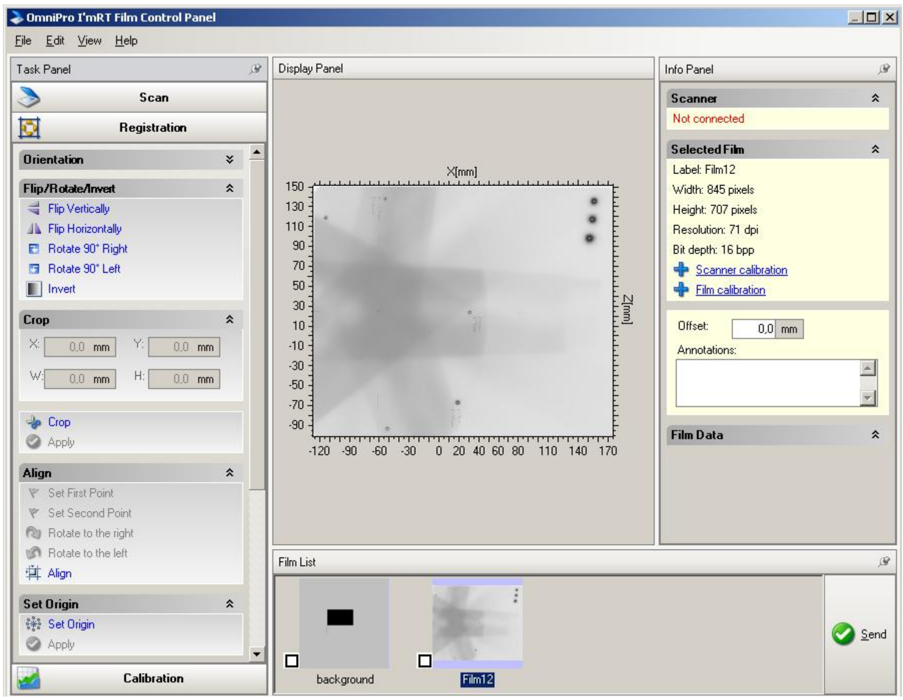

7.9.7.1. Phantom Orientation

Expand **Orientation** in the **Task Panel**.

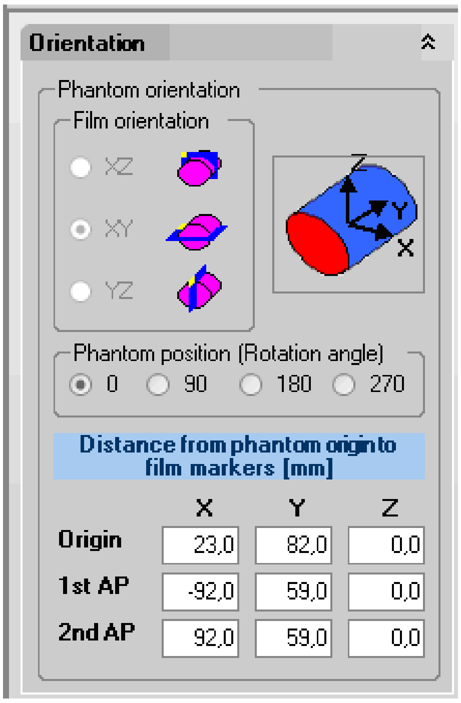

Film orientation: The orientation is displayed relative to the phantom, as defined in **Equipment:Body Phantom Setup**.

Phantom position (Rotation angle): Select the rotation angle. The IEC coordinate system is used.

CAUTION

CAUTION

CHANGE THE POSITION OF THE MARKER IF THE PHANTOM IS ROTATED

The default orientation of the body phantom is as shown in the screen-shot above. If the phantom is rotated, the position of the marker should also be changed.

Distance from phantom origin to film markers: Define the offsets for origin and alignment points (the distance from the phantom origin to these markers). If the offsets are defined in ***Equipment:Body Phantom Setup***, they will be displayed as read-only.

7.9.7.2. Flip/Rotate/Invert

Flip Horizontally flips the image along the vertical axis.

Flip Vertically flips the image along the horizontal axis.

Rotate 90° Right/Left turns the image 90° to the right or left.

Invert inverts the ADC values by subtracting the current values from the maximum ADC value, for the current bit depth (255, 4095, or 65535).

The modification can be applied simultaneously on all films in the film list. Mark all checkboxes, and apply the modification. The **Restore**/**Undo**/**Redo** commands will be applied on the currently displayed film.

7.9.7.3. Crop

Select **Crop**. A square appears in the image. Define the cropping area by dragging the corners of the square. Select **Apply**.

To return to a previously cropped area, select **Undo Crop**.

To return to the original image, select **Restore Original Film**.

The modification can be applied simultaneously on all films in the film list. Mark all checkboxes, and apply the modification. The **Restore/Undo/Redo** commands will be applied on the currently displayed film.

7.9.7.4. Set origin

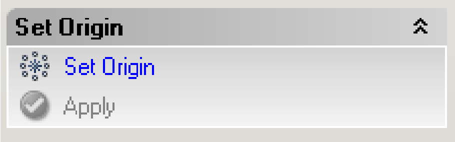

Select **Set Origin**.

Shortcut: <Ctrl-O>

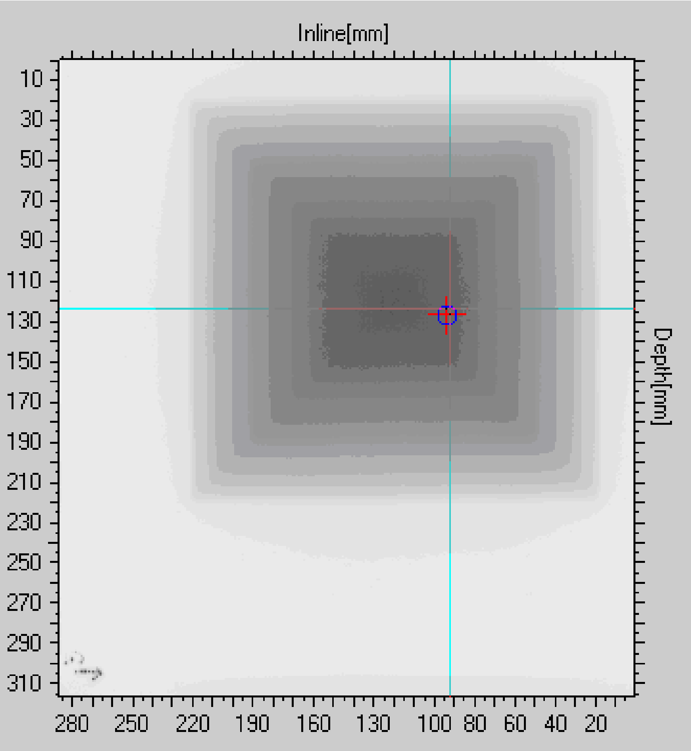

Define the origin by clicking on the point.

Click **Apply**.

Shortcut: <Ctrl-Shift-O>

7.9.7.5. Align

Select **Align**.

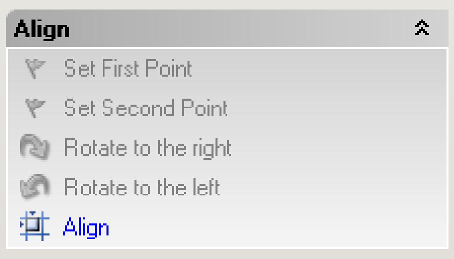

7.9.7.5.1. Manual Setting of Alignment Points

Set First Point: Select the first alignment point by clicking on the point. Use the **Zoom** function to improve precision.

Set **Second Point**: Select the second alignment point in the same way.

7.9.7.5.2. Automatic Setting of Alignment Points

Alignment points can also be set automatically.

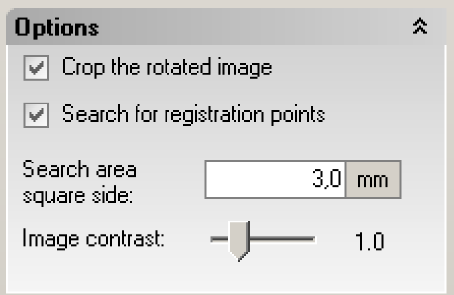

Mark the check-box **Search for registration points**, in **Options** in the **Task Panel**. Define the search area in **Search area square side**.

If the check-box **Crop the rotated image** is marked, the aligned film will be cropped to original size.

Search for the alignment points by clicking in the area surrounding them. The points with the lowest ADC values will be detected.

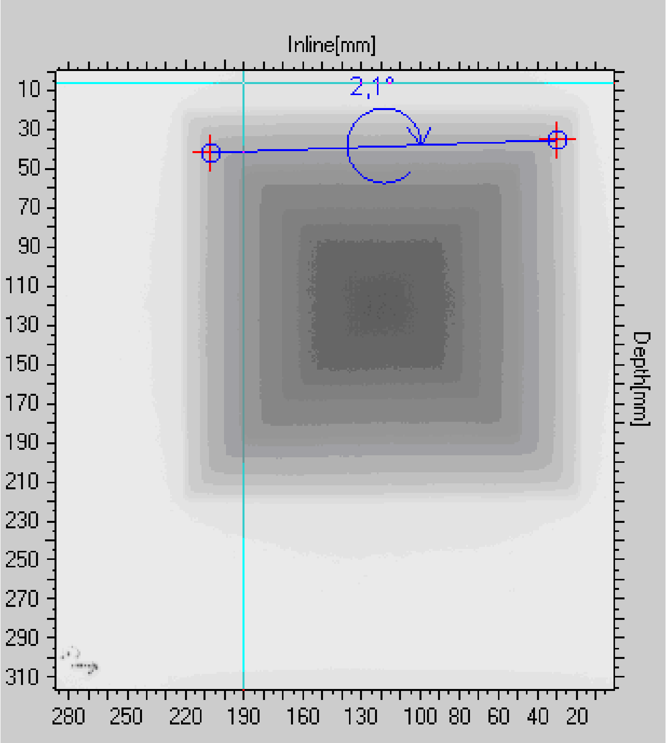

Rotate to the right/left in the **Align** box: Select the rotation direction.

7.9.7.6. Undo/Redo

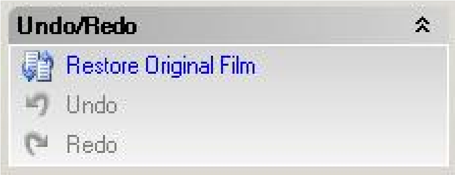

Restore Original Film will remove all modifications made.

Undo will remove the last modification made.

Redo will restore the modification that was removed with **Undo**.

7.9.7.7. Options

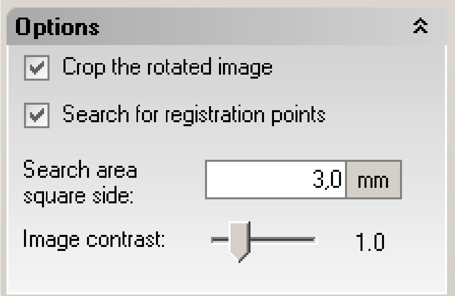

Crop the rotated image: See *7.9.7.5 Align*.

Search for registration points: See *7.9.7.5 Align*.

Search area square side: See *7.9.7.5 Align*.

Image contrast: Change the contrast of the image with the slider. The value 1.0 is default, and means that no contrast modification is applied. Changing the contrast does not change data, only the display of the image.

7.9.7.8. Zoom

Right-click in the image, and select ***Enable Zoom*** in the context menu.

A cursor appears. Define the zooming area with the cursor.

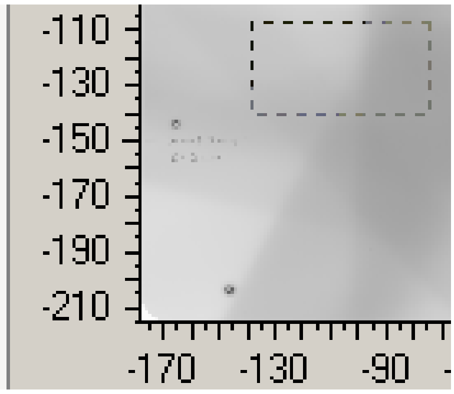

It is possible to define a new zooming area inside the current one. New zooming areas can be defined down to the minimum size 3 × 3 pixels.

To return to the previous zooming area, select ***Unzoom*** in the context menu.

To return to the original scaling, select ***Unzoom All*** in the context menu.

The zoom function is available in all tabs in the **Film Control Panel**.

7.9.7.9. Show Cursor

Right-click in the image and select ***Show cursor*** in the context menu. Move the cursor with the left mouse button held down.

Hold down the <Shift> button to display the Average value area.

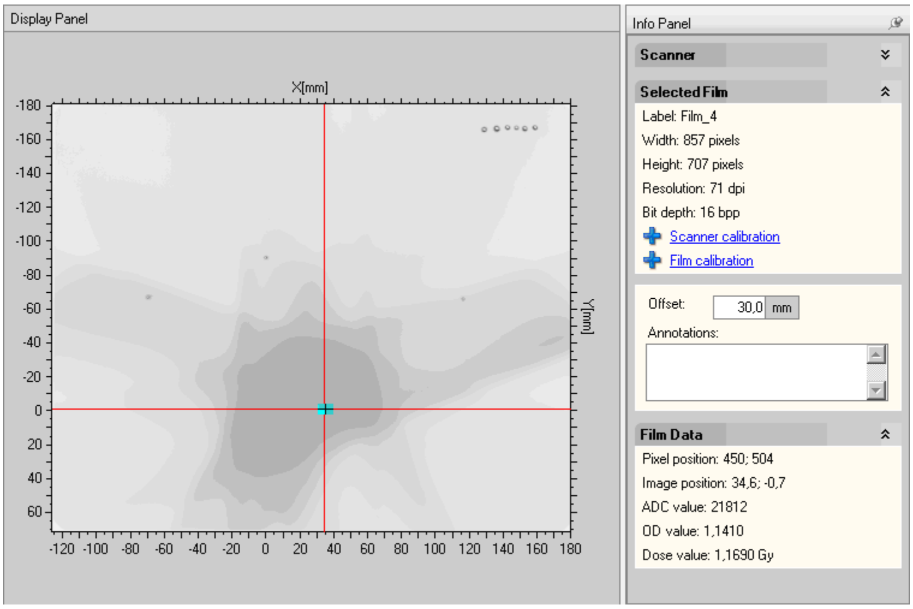

The current pixel position, image position, ADC, OD, and Dose values are displayed in **Film Data** in the **Info Panel**.

The show cursor function is available in all tabs in the **Film Control Panel**.

7.9.7.10. Change Palette

Right click in the image and select ***Change Palette*** in the context menu. The options ***Grayscale***, Grayscale Inverse, and **Rainbow** are available.

The change palette function is available in all tabs in the **Film Control Panel**.

7.9.7.11. Show Out of Calibration Range Values

Right click in the image and select ***Show out of calibration range values*** in the context menu.

Areas with values outside the calibration range will be marked with red color.

7.9.7.12. Save Image Data

Select ***Save*** in the ***File*** menu to save the current image. Images are saved in .tiff format. Calibration information and origin definition information is preserved.

Saving as an un-calibrated image is not possible, if the scanner and film are calibrated.

7.9.7.13. Load Image Data

Select ***Open..*** in the ***File*** menu to open image files.

The following formats are supported:

- .tiff format
  8, 16, 24, and 48 bit images (including GAFCHROMIC films) are recognized. For 24 and 48 bit RGB images individual channels can be extracted. The channel is selected in Edit:Options, and the selection is persistent between sessions.
- .opf format
  .opf is an internal OmniPro I'mRT format
- .flf format
  .flf is the internal OmniPro-Accept film format.
- Option:
  .dcm format
  .dcm is the DICOM CR film format.

7.9.7.14. Edit Menu, Options

Select ***Options*** in the ***Edit*** menu to define the following options:

- Check for out of range values
- Default origin position
- Dose unit
- Extract channel
- Length unit
- Measure area square side

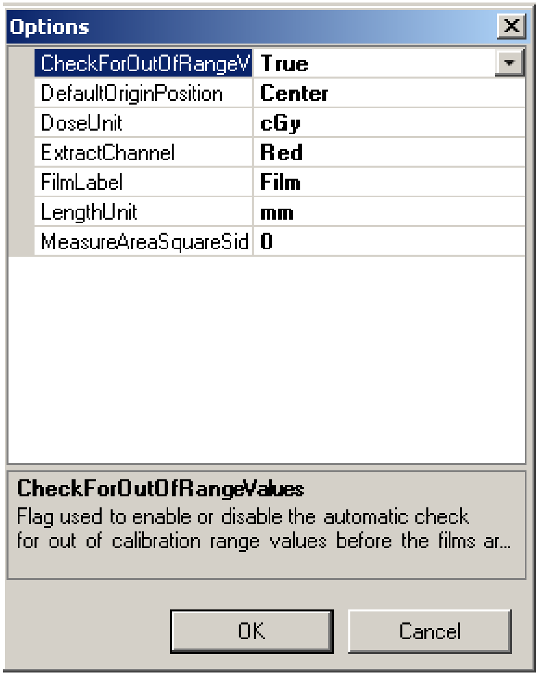

Check For Out Of Range Values: Select **True** or **False** to turn the function on/off.

Default Origin Position: Define the default origin position. The default origin is applied only if the image has no origin defined.

Dose Unit: The selected unit will be applied in all views and files.

Extract Channel: Select channel for import of RGB files.

Film Label: Define a name that will be used as film label in the **Film List**. The default label is **Film**.

Length Unit: The selected unit will be applied in all views and files.

Measure Area Square Side: Define the area used for ADC mean value calculation (average value area).

NOTICE

IMPORTANT NOTICE

MEASURE AREA IN CALIBRATIONS

The measure area used in calibrations is defined in the **Options** box in the **Task Panel** in the **Calibration** tab:

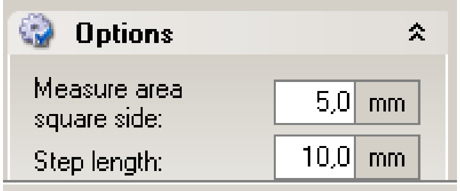

8. Data Import

NOTICE

IMPORTANT NOTICE

CORRUPT FILES

There are file types that are not secured via a check sum. If such a file has been corrupt by editing and manipulating the file, it will in most cases not be possible to import or open the file. However, in a few cases it may be possible to import or open a file that contains incorrect information. Never manipulate or edit files in a way not intended by the manufacturer! Always check the consistency of data!

NOTICE

IMPORTANT NOTICE

LARGE FILES

Depending of the RAM capacity of the computer used, it may be impossible, or take very long time, to import very large files.

NOTICE

IMPORTANT NOTICE

DICOM CONFORMANCE

OmniPro I'mRT is a common platform for digital, film, and EPID IMRT verification. To support these functions some of the radiotherapy objects (RT Plan, RT Image, and RT Dose), and the CR Image modality defined by the DICOM standard are supported for import. Please refer to the DICOM Conformance Statement [2] for details. See *References*. The document is available on the OmniPro I'mRT installation disk.

NOTICE

IMPORTANT NOTICE

IMPORT OF DATA IN GRAPHIC FORMAT

Import of graphic files is restricted to 16 bit grey scale images, and any attempt to open an image with another bit depth will result in an error.

Since the files do not contain any calibration information, the current scanner and film calibration will be applied.

NOTICE

IMPORTANT NOTICE

ORIENTATION OF ISOCENTER

The transformation of the coordinate system where the dose is stored to the gantry coordinate system is not possible from the dose information. Therefore a movement of the orientation might be done manually.

8.1. Import of Dose Planes and Dose Cubes

Select the menu command ***File:Import Data*** to import dose planes or dose cubes in the following formats:

- DICOM RDOSE
- RTOG,
- CadPlan
- CMS
- Plato

Dose Planes

Dose plane is calculated 2D data. Select the menu command ***File:Import Data:TPS Dose Planes***, or click the button 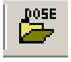 in the toolbar.

Dose cubes

Dose cube is calculated 3D data. Select the menu command ***File:Import Data:TPS Dose Cube***.

A window for TPS data import will open. In the left panel patient and data information, embedded in the imported image, will be displayed.

The panel to the right contains three tabs, **Imported Data**, **Data Transformation**, and **Options**, containing the functions and information needed for import and data transformation.

8.1.1. DICOM Import

Select the DICOM RTDOSE tab. The Import dose distributions in DICOM RTDOSE format dialog opens.

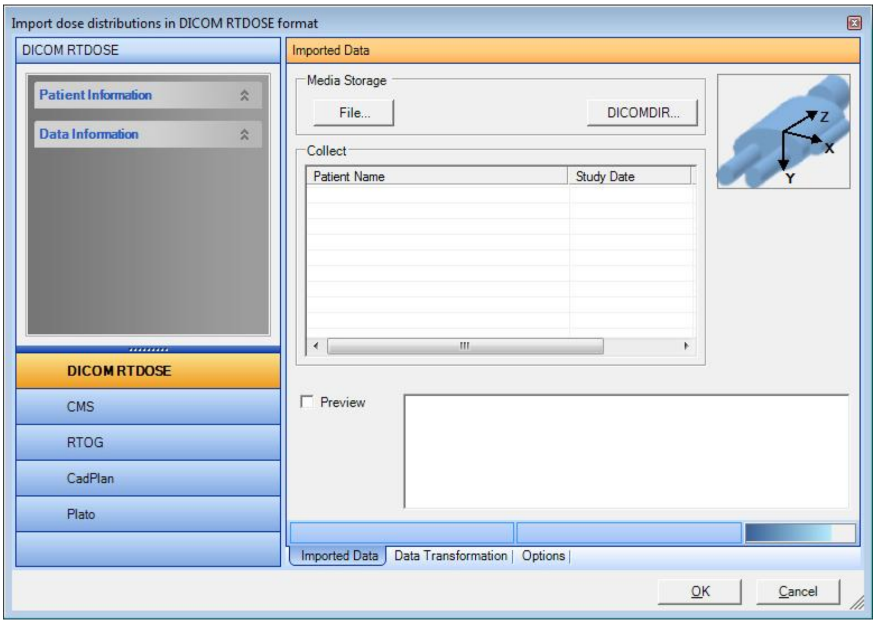

The orientation of the imported slice will be visualized in the coordinate system in the upper right corner of the dialog. The orientation of the coordinate system will be changed corresponding to the attributes from the imported DICOM data. It is not possible to import dose planes with different orientation at the same time.

The images will be displayed in the **Image Preview** box, with the original TPS orientation.

The images can be imported from file, via DICOMDIR, or via TCP/IP (StorageSCP Service).

Select the **Options** tab and define the TCP parameters, and the logging directory (optional):

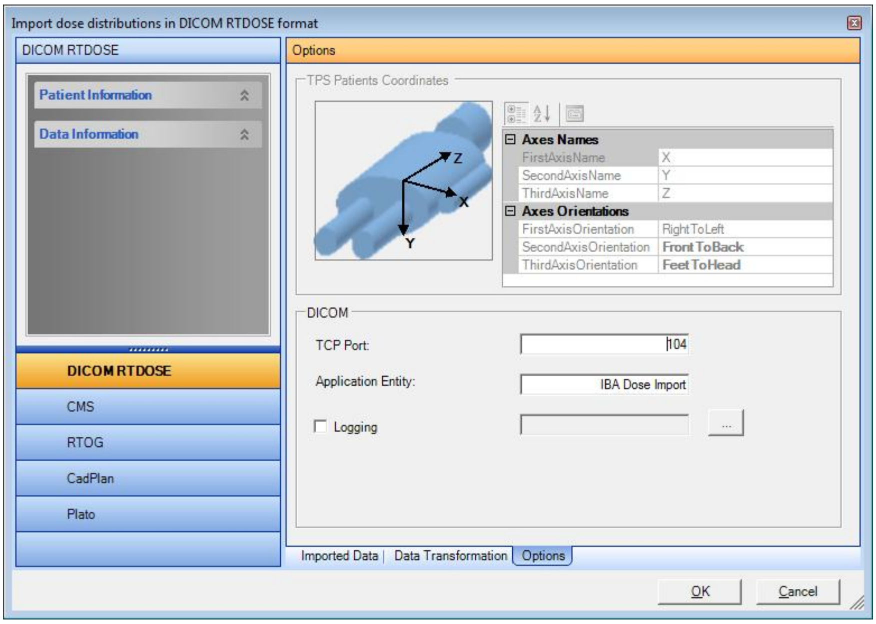

Port number: 0 - 65535.

Application Entity: Application entity title.

NOTICE

IMPORTANT NOTICE

SPACES IN TITLE

Do not use spaces in the application entity title.

8.1.1.1. Import from a Local Data Source

Select the **Imported Data** tab.

Click **File** to import files from your local data source.

A standard **Open** dialog opens. Browse for and select the required file. Click **Open**.

The file will be imported and displayed.

To continue, see *8.1.1.4 Change coordinates*.

8.1.1.2. Import via DICOMDIR

DICOMDIR is a file which contains information about other DICOM files. These files shall be in the same folder or in a folder below the DICOMDIR file. Filenames must be 8 characters without extension.

Click the **DICOMDIR…** button.

Select the folder where the DICOMDIR file is. A list of data is displayed. Only RT Dose data is displayed.

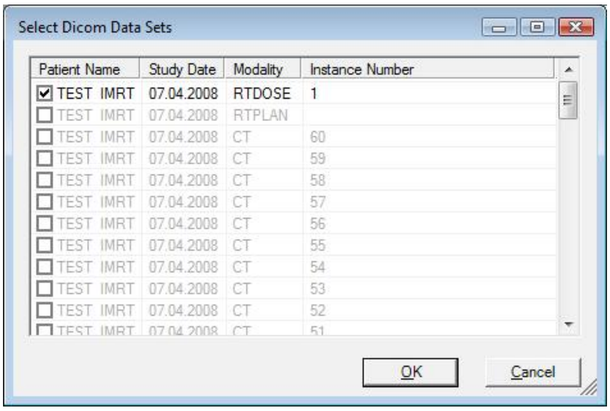

Select which data to import and click **OK**. The images are displayed in the image list.

To continue, see *8.1.1.4 Change coordinates*.

8.1.1.3. TCP/IP (StorageSCP Service) Import

The StorageSCPService is a Windows service, which collects DICOM data via TCP/IP and saves it on the disk. The service is started at system start, so files can be collected even if the OmniPro I'mRT software is not running.

The TCP settings are changed in the **Options** dialog.

The collected data is displayed in the **Collect** list. Only RT Dose data is displayed.

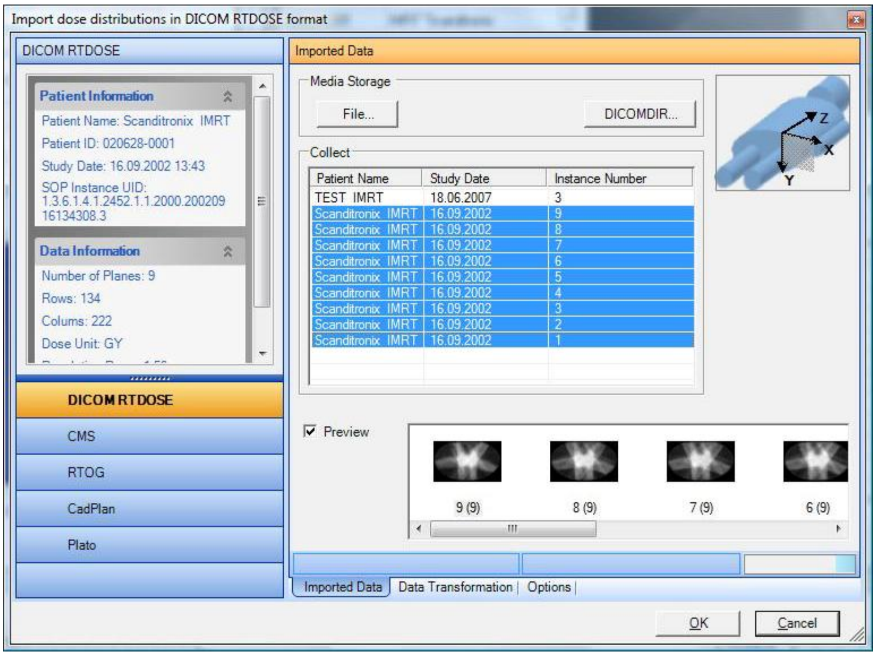

Select the data to import. If **Preview** is selected, the data is displayed in the image list.

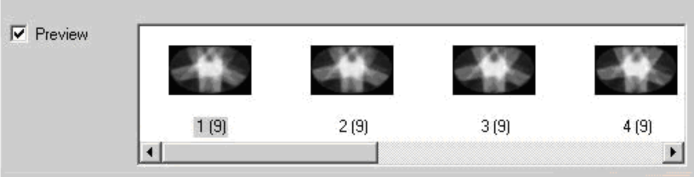

The imported data is automatically removed from the list.

To continue, see *8.1.1.4 Change coordinates*.

8.1.1.4. Change Coordinates

Select the **Data Transformation** tab. Click **Change** and enter the distance from TPS origin to phantom origin. The changed data is reflected in **Extensions**.

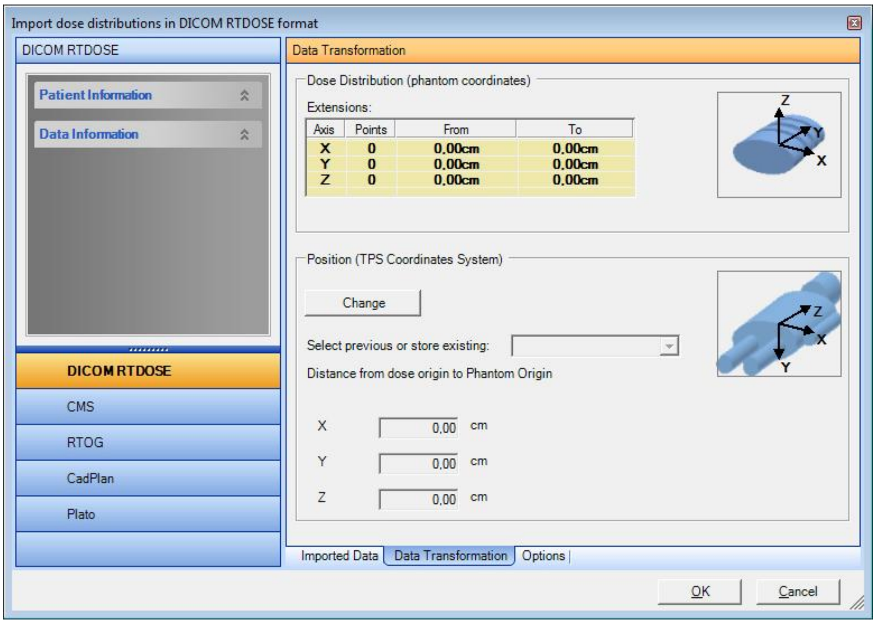

Different offsets can be named and selected in **Select previous or store existing** offset. The selection of offsets will be persistent.

Add a new offset by typing a new name, and then change the numerical values. An existing, named offset can be changed by changing the numerical values.

To continue, see *8.1.6 Send to I'mRT Workspace*.

8.1.2. CMS Import

1. Select the CMS tab to open Import Dose Distributions in CMS Format.
2. Select the Imported Data tab.

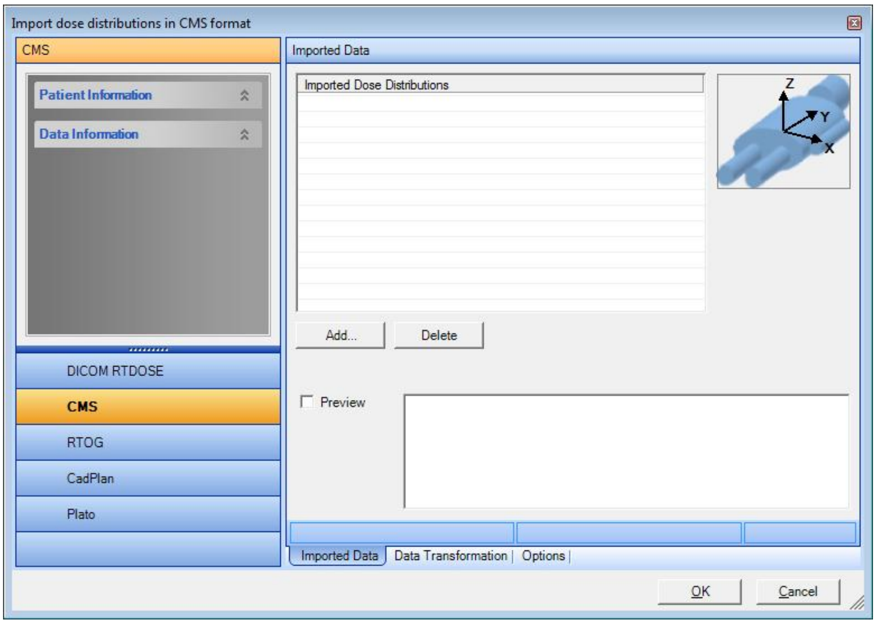

3. Click **Add**, and select the requested CMS files.

The images will be displayed in the Image Preview box, with the original TPS orientation.

The orientation of the imported slice will be visualized in the coordinate system in the upper right corner of the dialog.

4. Select the **Data Transformation** tab. Click **Change** and enter the distance from TPS origin to phantom origin. The changed data is reflected in **Extensions**.

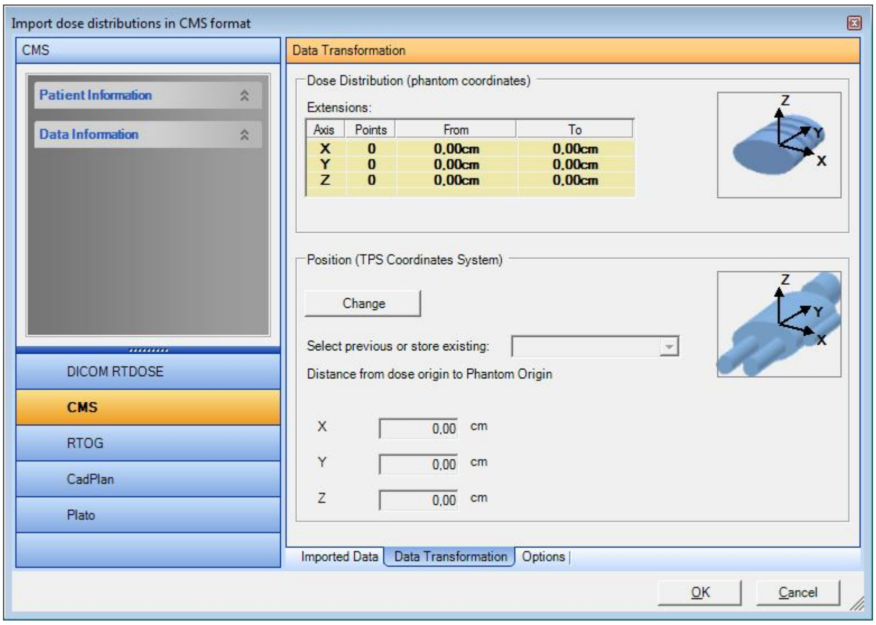

Different offsets can be named and selected in **Select previous or store existing** offset. The selection of offsets will be persistent.

Add a new offset by typing a new name, and then change the numerical values. An existing, named offset can be changed by changing the numerical values.

The **Options** tab displays the TPS coordinate system.

5. To continue, see *8.1.6 Send to I'mRT Workspace*.

8.1.3. RTOG Import

Binary and ASCII RTOG files can be imported.

NOTICE

IMPORTANT NOTICE

FILE NAMING

The directory file must end with zeros. The number of zeros determine the format of image files reference by their image number in the reference file. E.g. directory file name: aapm0000. If an image number in this is 12, the corresponding image file name has to be 'aapm0012'.

Click **RTOG**. The **Import dose distributions in DICOM RTDOSE format** dialog opens.

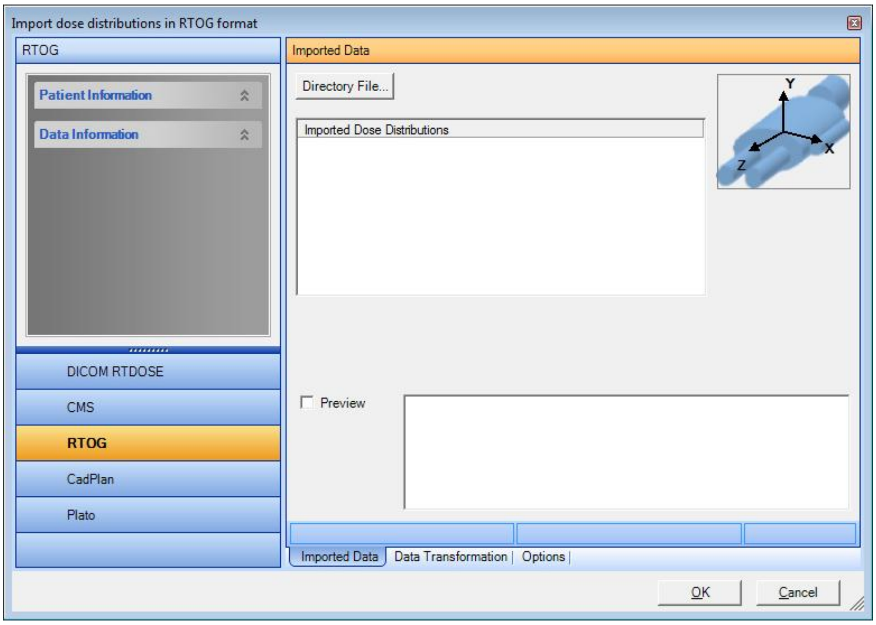

Select the **Imported Data** tab.

Click **Directory File**. A standard **Open** dialog opens.

The orientation of the imported slice will be visualized in the coordinate system in the upper right corner of the dialog.

Browse for, and select the directory file. All dose distributions in the file are displayed. Select a dose distribution in the list view.

The images will be displayed in the **Image Preview** box, with the original TPS orientation.

Select the **Data Transformation** tab. Click **Change** and enter the distance from TPS origin to phantom origin. The changed data is reflected in **Extensions**.

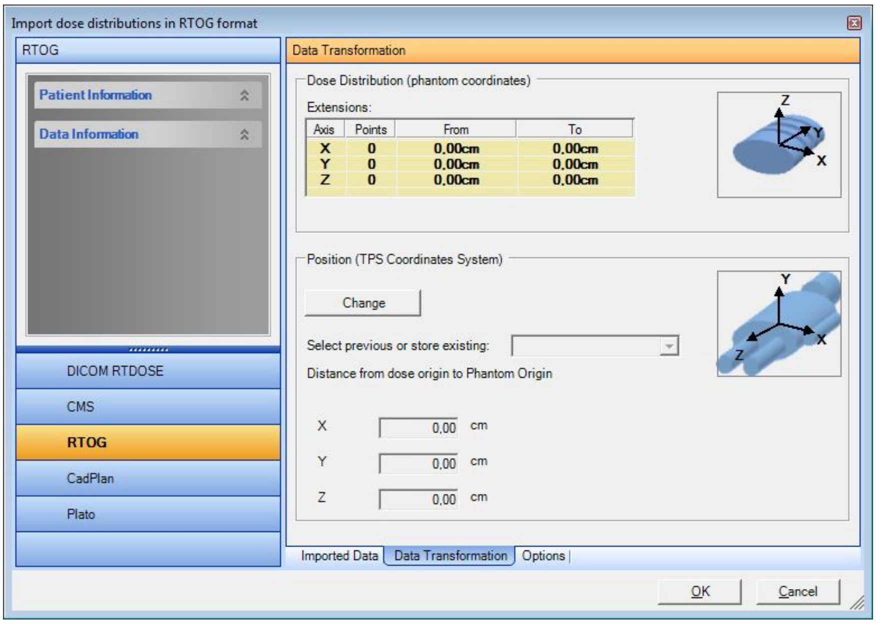

Different offsets can be named and selected in **Select previous or store existing** offset. The selection of offsets will be persistent.

Add a new offset by typing a new name, and then change the numerical values. An existing, named offset can be changed by changing the numerical values.

The **Options** tab displays the TPS coordinate system.

To continue, see *8.1.6 Send to I'mRT Workspace*.

8.1.4. CadPlan Import

Select the **CadPlan** tab. **Import dose distribution in CadPlan format** opens.

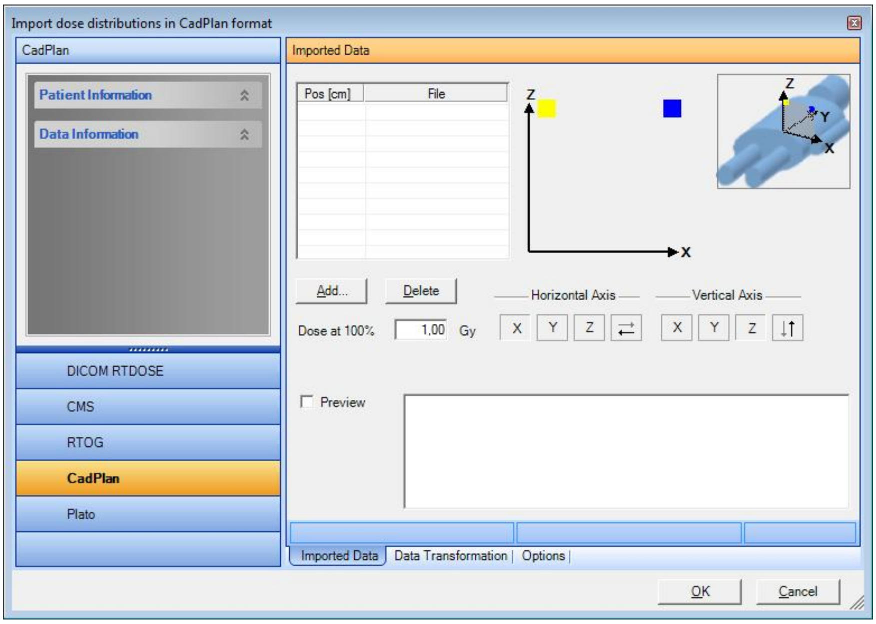

Select the **Imported Data** tab.

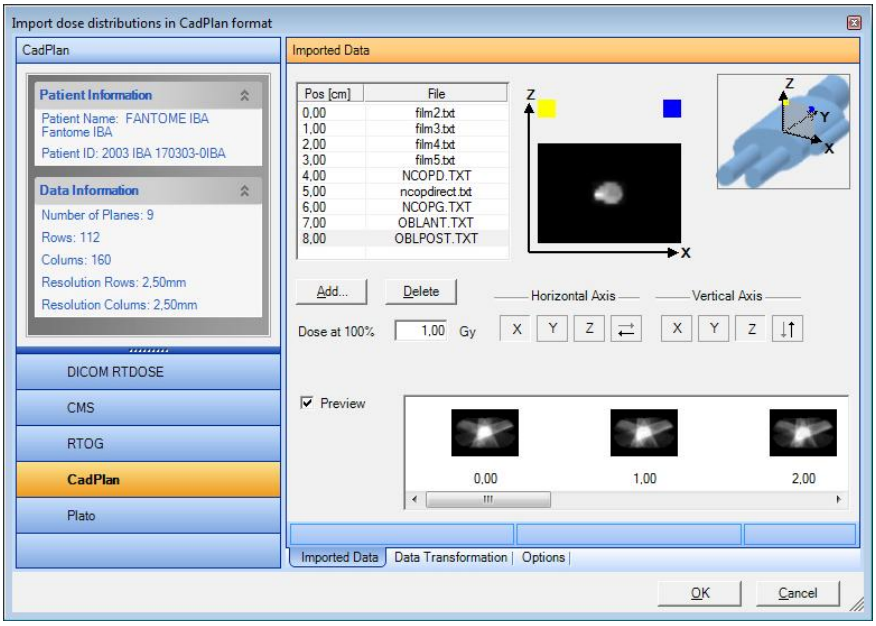

Click **Add**, and select the requested CadPlan files. Click **Open**.

Select the **Options** tab. Define a coordinate system for the TPS, and (optional) name the labels of the axes.

Select the **Data Transformation** tab.

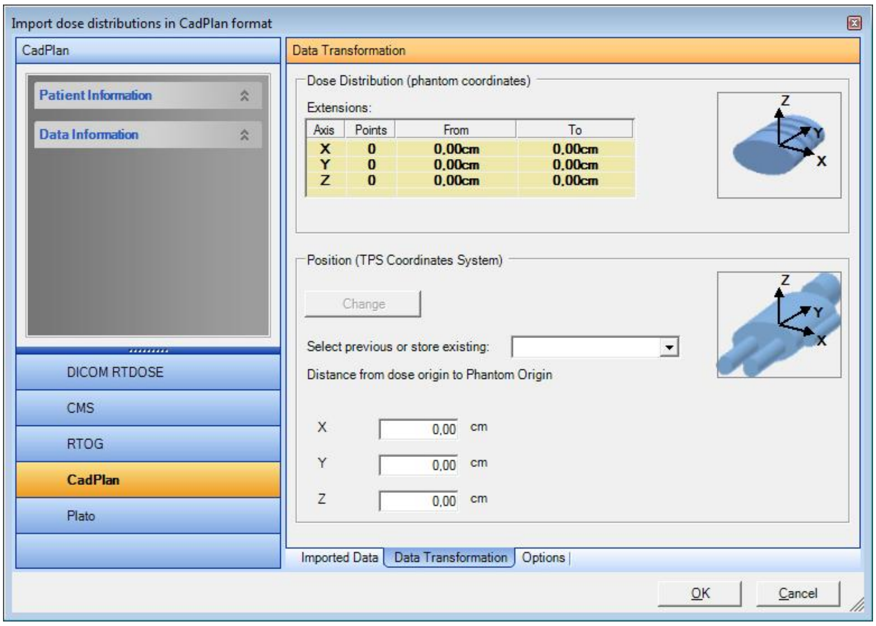

Click **Change** and enter the distance from TPS origin to phantom origin. The changed data is reflected in **Extensions**.

Different offsets can be named and selected in **Select previous or store existing** offset. The selection of offsets will be persistent.

Add a new offset by typing a new name, and then change the numerical values. An existing, named offset can be changed by changing the numerical values.

Select the **Imported Data** tab. Click the **X**, **Y**, **Z**, and arrow buttons to define the plane orientation.

Enter the plane positions in the **Pos (cm)** fields. The default distance value is 0.1 cm.

Enter the dose at 100%.

To continue, see *8.1.6 Send to I'mRT Workspace*.

8.1.5. Plato Import

Select the **Plato** tab. **Import dose distribution in Plato format** opens.

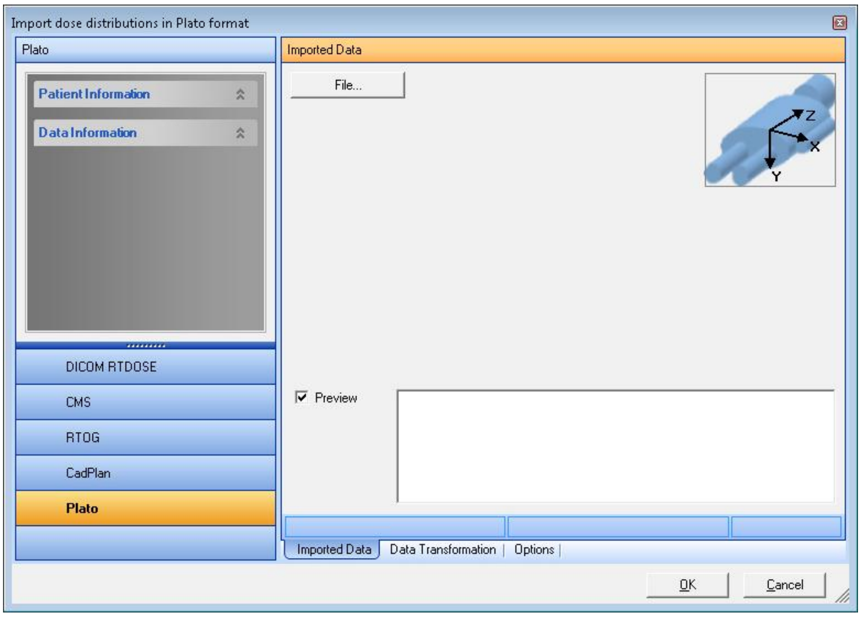

Select the **Imported Data** tab.

The icon in the upper right corner displays the TPS coordinate system.

Click **File**, and select the requested files. The images will be displayed in the **Image Preview** box, with the original TPS orientation.

Select the **Data Transformation** tab. Click **Change** and enter the distance from TPS origin to phantom origin.

The changed data is reflected in **Extensions**.

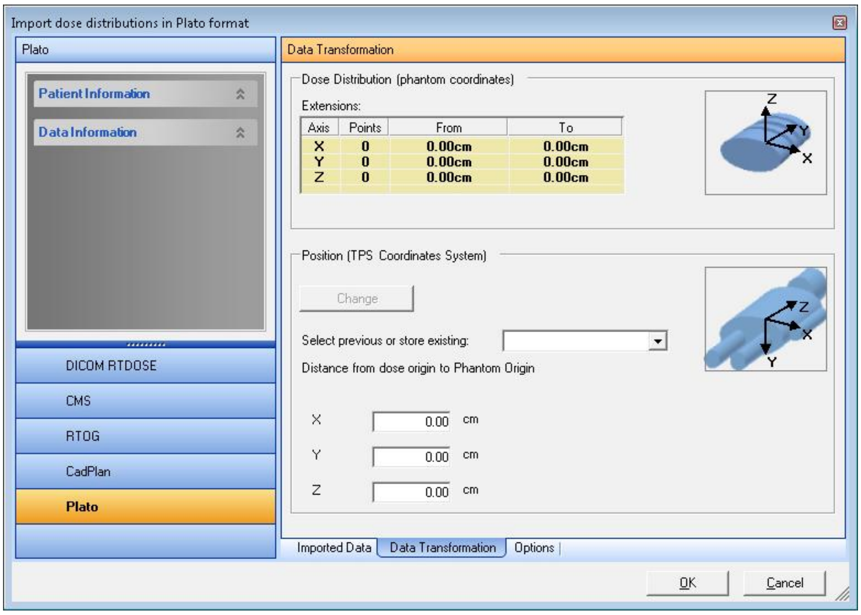

Different offsets can be named and selected in **Select previous or store existing** offset. The selection of offsets will be persistent.

Add a new offset by typing a new name, and then change the numerical values. An existing, named offset can be changed by changing the numerical values.

The **Options** tab displays the TPS coordinate system.

To continue, see *8.1.6 Send to I'mRT Workspace*.

8.1.6. Send to I'mRT Workspace

Click **OK** in the **Imported Data** tab. The **Importing Data** dialog opens.

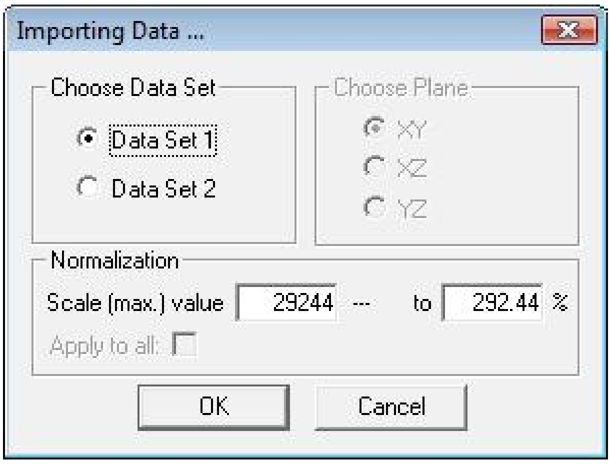

Choose if data shall be saved as **Data set 1** or **Data set 2**.

Define the normalization value.

Tip! By default the **Normalization** is selected in such a way that 1 Gy becomes 100% in the OmniPro I'mRT main program. This means that the values displayed in OmniPro I'mRT can be interpreted as cGy (or rad). Film data may also be imported with a rescaling factor of 1 Gy = 100%. By this, the planned data and measured data can be compared without further rescaling.

Click **OK**. The imported data is displayed in the OmniPro I'mRT Workspace.

8.2. Fluence Import

Fluence files can be imported from the following treatment planning systems:

- CadPlan,
- Pinnacle,
- RTPlan Compensator,
- CMS,
- KonRad.

Select the menu command ***File:Import Data:TPS Fluence Map***.

A window for TPS fluence map import will open. In the left panel patient and data information, embedded in the imported image, will be displayed.

The panel to the right contains three tabs, **Imported Data**, **Fluence Planes**, and **Options**, containing the functions and information needed for import and data transformation.

8.2.1. CadPlan, Pinnacle, and CMS

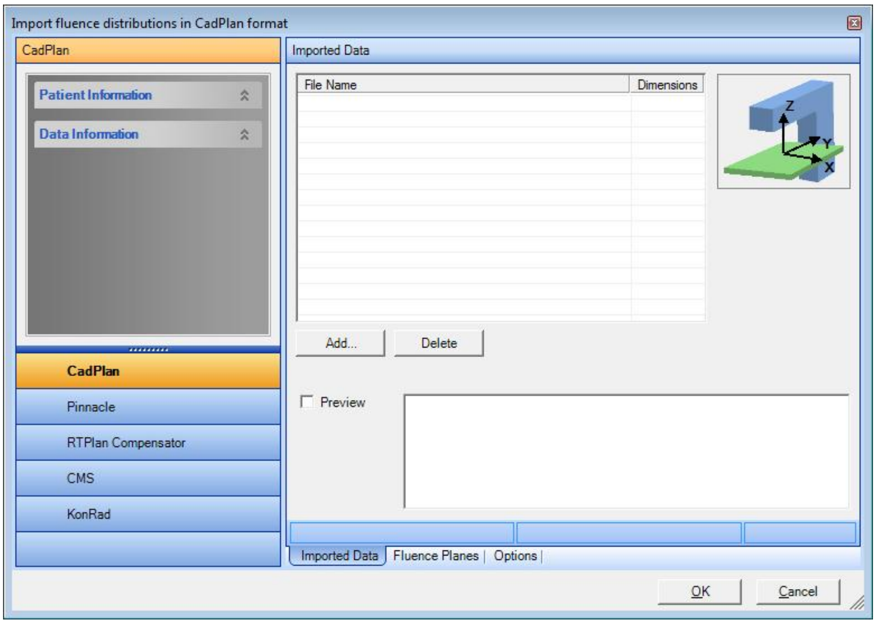

Select **CadPlan**, **Pinnacle**, or **CMS**.

Select the Imported data tab. Click **Add**. A standard **Open** dialog opens. Select the required file and click **Open**. The imported fluence map data is displayed.

Select the **Fluence Planes** tab to edit **SSD** (Source to Surface Distance) and **Depth** (build-up).

Distance, i.e. the distance from the source to the fluence plane, will be calculated automatically.

If required, enter the value for the collimator **Rotation** and the **Gantry** angle.

NOTICE

IMPORTANT NOTICE

IMAGE ROTATION

The imported fluence will be rotated in the same direction as the collimator is rotated (according to the definition in IEC 61217). The image will therefore appear to be rotated in the direction opposite to the specified angle. The definition of the direction of angles in I'mRT is in the other direction. Therefore the angle displayed in the **Field** tab and **Modification** tabs of the Parameter window will have the opposite sign.

The **Options** tab displays the X-Ray Receptor coordinate system.

NOTICE

IMPORTANT NOTICE

PINNACLE 6.2 AND LATER

Pinnacle v.6.2 and later: A flipping error in the x-axis has been corrected in OmniPro I'mRT, from version 1.4.

To continue, see *8.1.6 Send to I'mRT Workspace*.

8.2.2. DICOM Compensator Fluence

Fluence maps can be imported from the compensator transmission data of the RTPlan IOD. Select **RTPlan Compensator**.

Select the **Imported Data** tab.

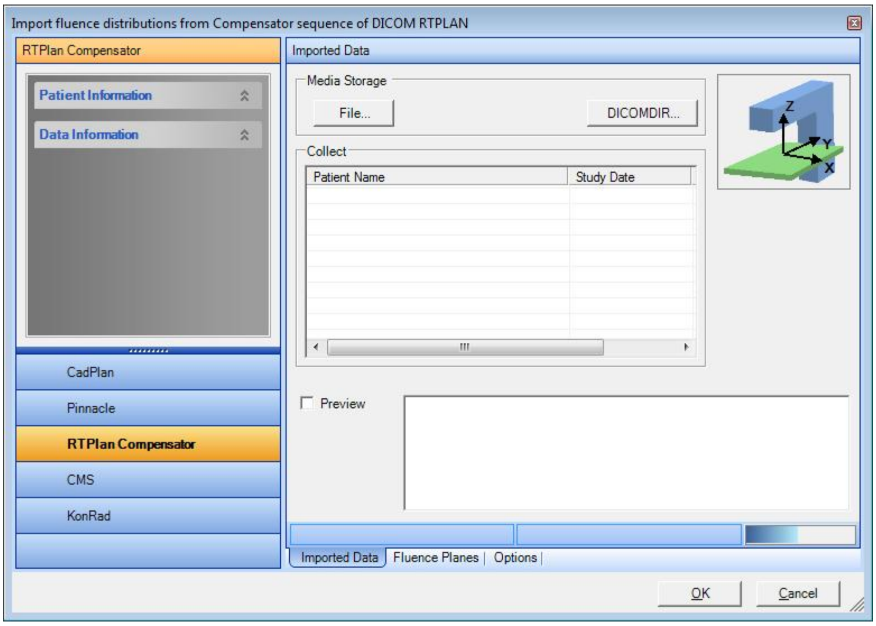

The beams can be imported from file, via DICOMDIR or via TCP/IP import (StorageSCP Service)

Two compensator types are available: **OPTIMAL** and **TOTAL ACTUAL**. Select the **Options** tab.

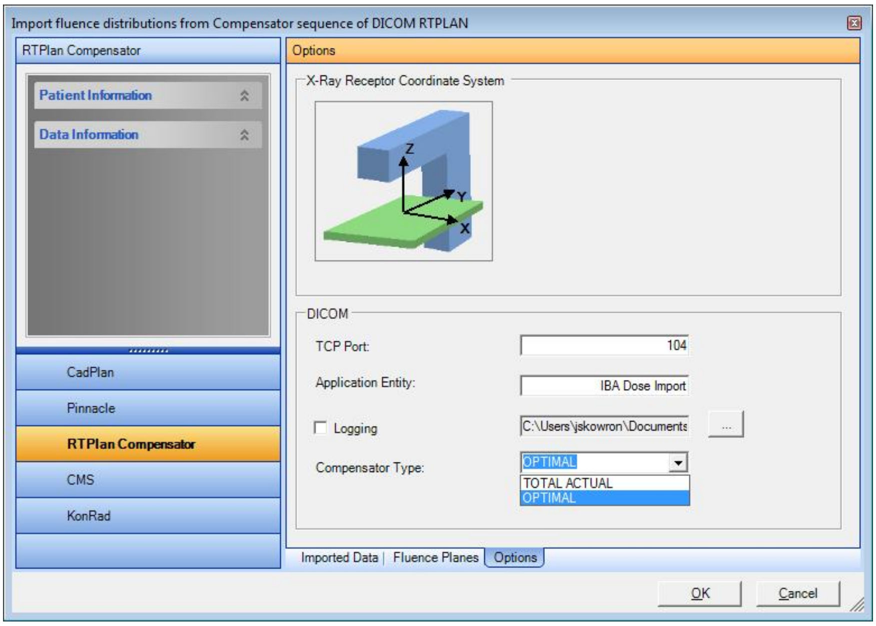

Select the compensator type. The selection is persistent between sessions.

Define the TCP port, and the logging directory (optional):

Port number: 0 - 65535.

Application Entity: Application entity title.

NOTICE

IMPORTANT NOTICE

SPACE IN TITLE

Do not use space in the application entity title.

8.2.2.1. Import from a Local Data Source

Click **File** in the **Imported data** tab to import files from your local data source.

A standard **Open** dialog opens. Browse for and select the required file. Click **Open**.

Enter location.

8.2.2.2. Import via DICOMDIR

DICOMDIR is a file which contains information about other DICOM files. These files shall be in the same folder or in a folder below the DICOMDIR file. Filenames must be 8 characters without extension.

Click the **DICOMDIR…** button.

Select the folder where the DICOMDIR file is. A list of data is displayed. Only RTPlan data is displayed.

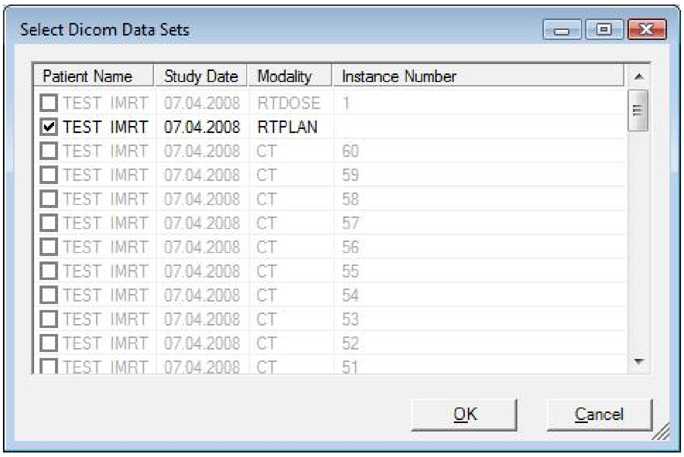

Select the data to import and click **OK**. The images are displayed in the image list.

To continue, see *8.2.5 Enter location*.

8.2.2.3. TCP/IP Import (StorageSCP Service)

The StorageSCPService is a Windows service, which collects DICOM data via TCP/IP and saves it on the disk. The service is started at system start, so files can be collected even if the OmniPro I'mRT software is not running.

The TCP settings are changed in the **Options** tab.

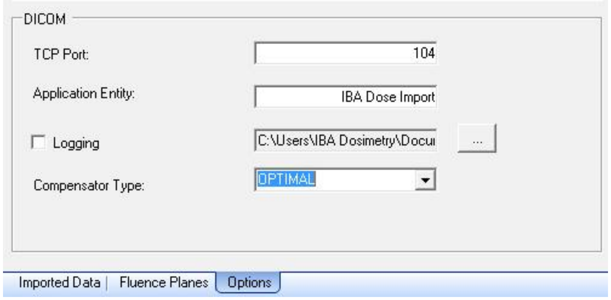

The collected data is displayed in the **Collect** list in the **Imported data** tab. Only RT Plan with Compensator data is displayed.

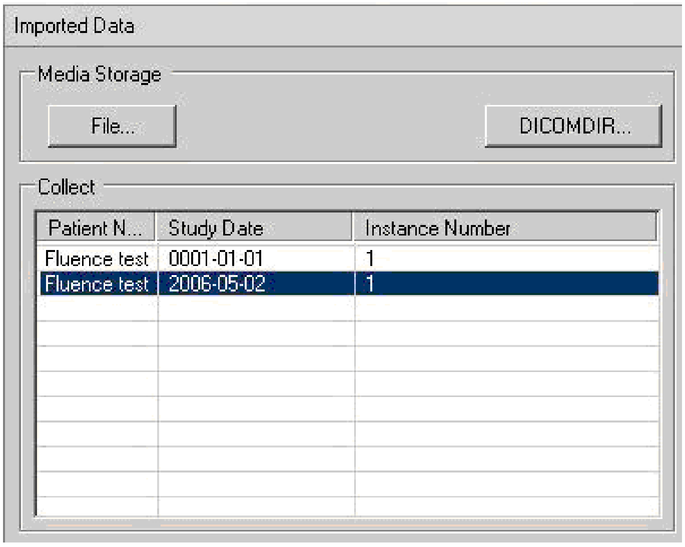

Select the data to import. If **Preview** is selected the data is displayed in the image list.

The imported data is automatically removed from the list .

To continue, see *8.2.5 Enter location*.

8.2.3. KonRad Fluence Import

Select the **KonRad** button. The **Import fluence distributions in KonRad format** dialog opens.

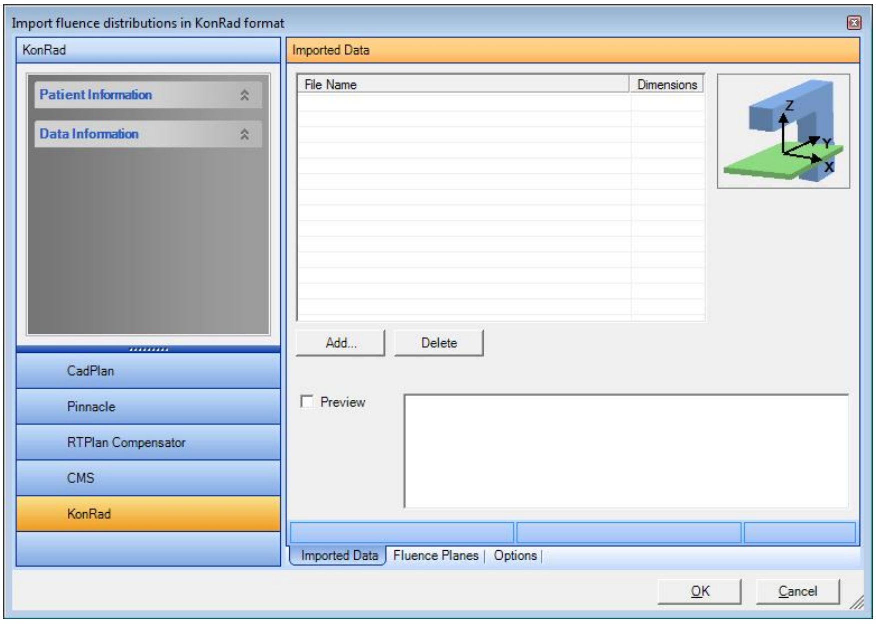

Click **Add** and select the requested files. The imported fluence map data is displayed.

Select the **Fluence Planes** tab to edit **SSD** (Source to Surface Distance) and **Depth** (build-up).

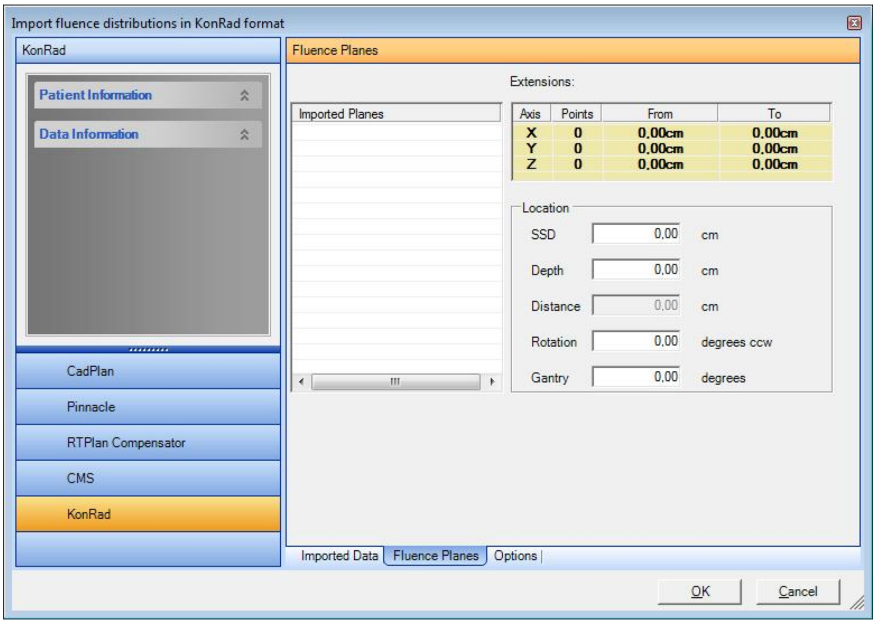

Distance, i.e. the distance from the source to the fluence plane, will be calculated automatically.

If required, enter the value for the collimator rotation and collimator angle.

NOTICE

IMPORTANT NOTICE

IMPORTED FLUENCE ROTATION

The imported fluence will be rotated in the same direction as the collimator (according to the definition in IEC 61217). The image will therefore appear to be rotated in the direction opposite to the specified angle. The definition of the direction of angles in I'mRT is in the other direction. Therefore the angle displayed in the **Field** tab and the **Modification** tab of the **Parameter** window will have the opposite sign.

The **Options** tab displays the X-ray receptor coordinate system.

Click **OK** in the **Fluence** tab. The **Importing data** dialog opens.

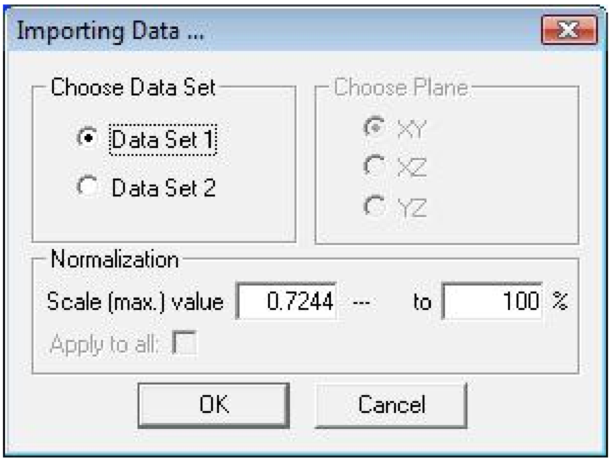

Select the data set and, if necessary, edit the scale values.

Click **OK**. Data will be sent to the I'mRT Workspace. The data imported are internal fluence values. To convert fluence to monitor units (MU), the following conversion formula has to be applied:

MU = 100 · fluence / number of fractions.

Open the **Rescaling** dialog, and select **With fix factor**.

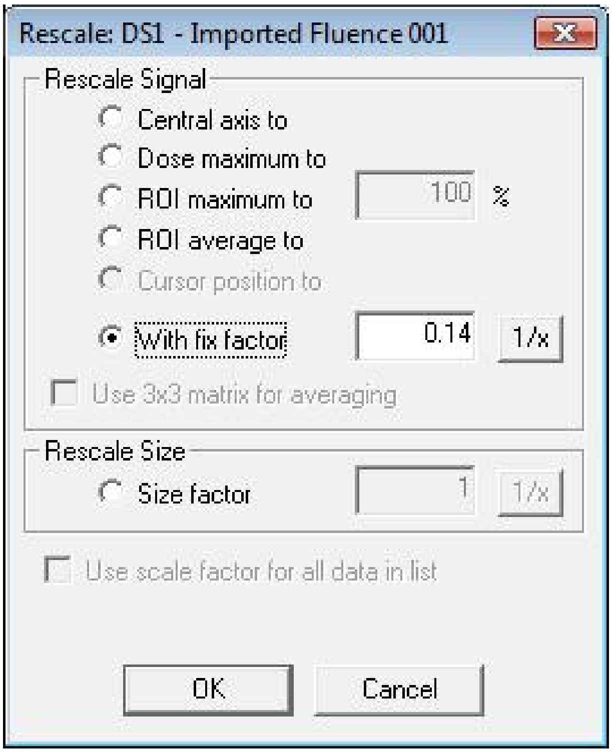

Enter the number of fractions, divided by 100.

Click the **1/x** button.

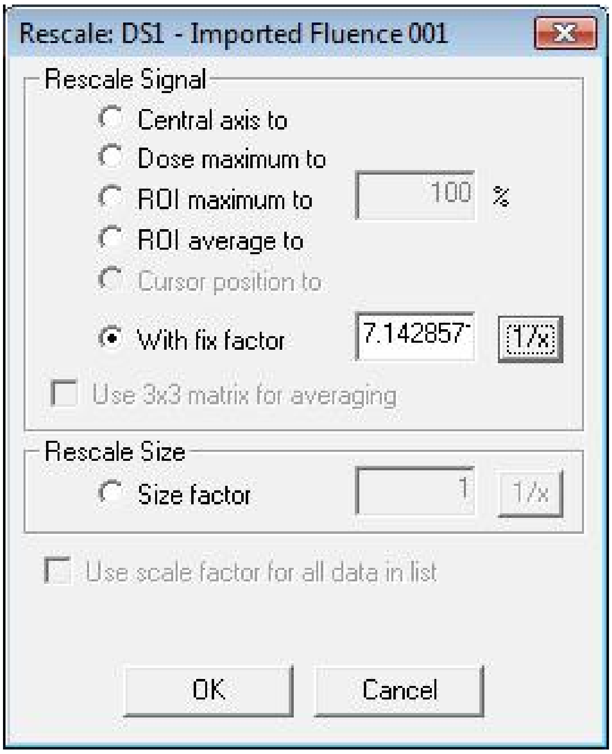

Click **OK**. The conversion formula will be applied.

8.2.4. DICOM RT Image Import

Select ***File:Import Data:DICOM RT Image***. The **Import Dicom RTImage** window will open. In the left panel, patient and data information, embedded in the imported image, will be displayed.

The panel to the right contains three tabs, **Imported Data**, **Image Planes**, and **Options**, containing the functions and information needed for import and data transformation.

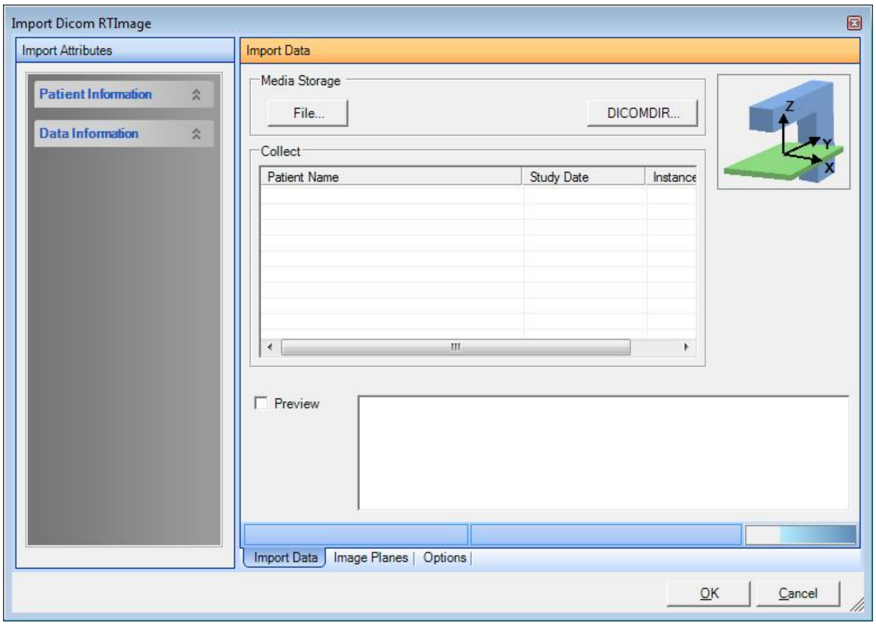

The images can be imported from file, via DICOMDIR or via TCP/IP (StorageSCP Service).

Select the **Options** tab, and define the TCP port, and the logging directory (optional):

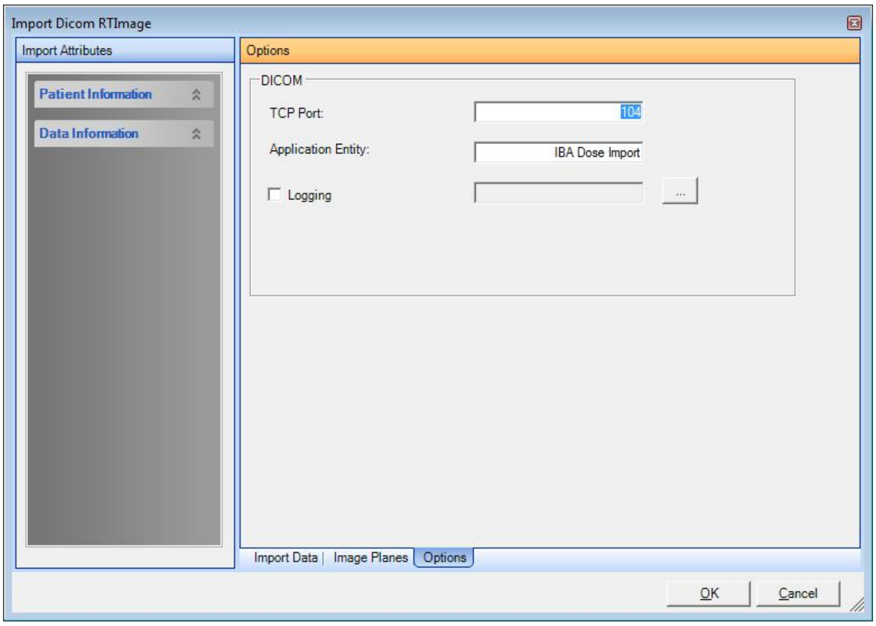

Port number: 0 - 65535.

Application Entity: Application entity title.

NOTICE

IMPORTANT NOTICE

SPACE IN TITLE

Do not use space in the application entity title.

8.2.4.1. Import from a Local Data Source

Click **File** to import files from your local data source.

A standard **Open** dialog opens. Browse for and select the required file. Click **Open**.

The selected file is imported and displayed.

To continue, see *8.2.5 Enter location*.

8.2.4.2. Import via DICOMDIR

DICOMDIR is a file which contains information about other DICOM files. These files shall be in the same folder or in a folder below the DICOMDIR file. Filenames must be 8 characters without extension.

Press the **DICOMDIR…** button.

Select the folder where the DICOMDIR file is. A list of data is displayed. Only RTImage data is displayed.

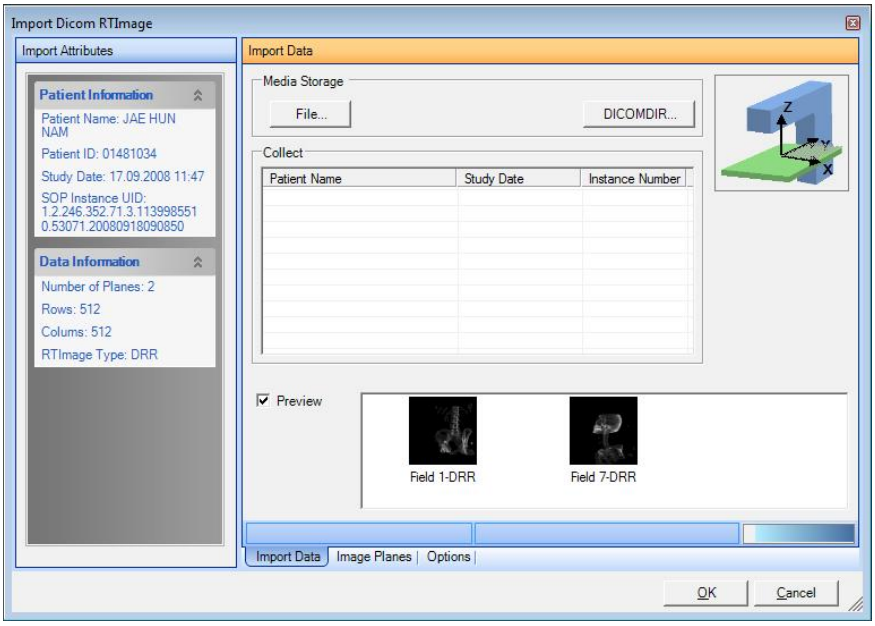

Select the data to import and press **OK**. The images are displayed in the image list.

To continue, see *8.2.5 Enter location*.

8.2.4.3. TCP/IP Import (StorageSCP Service)

The StorageSCPService is a Windows service, which collects DICOM data via TCP/IP and saves it on the disk. The service is started at system start, so files can be collected even if the OmniPro I'mRT software is not running.

The TCP settings are changed in **Options** tab.

The collected data is displayed in the **Collect** list in the **Import Data** tab. Only RT Image data is displayed.

Select the data to import. If **Preview** is selected, the data is displayed in the image list.

The imported data is automatically removed from the list.

To continue, see *8.2.5 Enter location*.

8.2.5. Enter Location

Select the **Image Planes** tab to enter **SSD** and **Depth** (build-up).

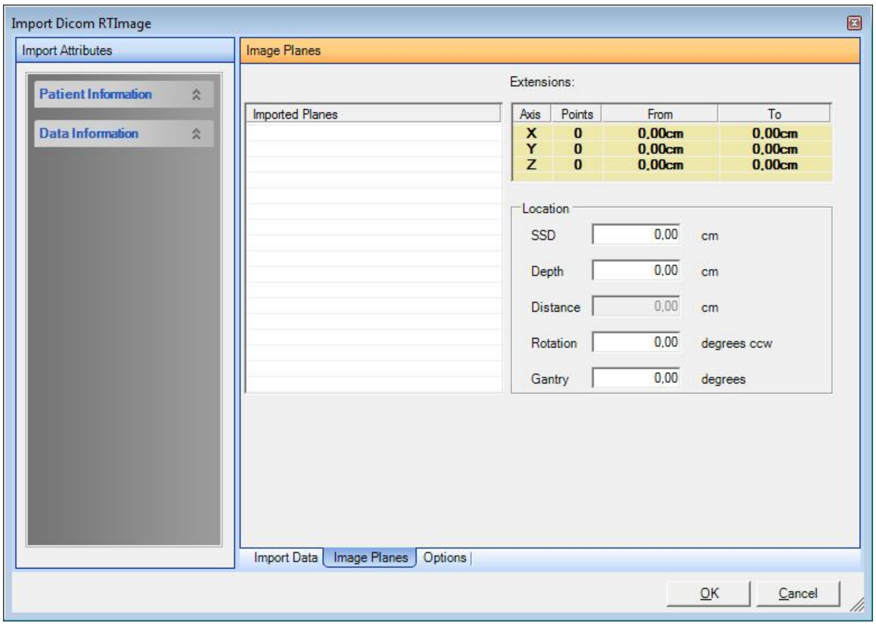

Distance, i.e. the distance from the source to the fluence plane, will be calculated automatically.

If required, enter the value for the collimator **Rotation** and the **Gantry** angle.

NOTICE

IMPORTANT NOTICE

IMAGE ROTATION

The imported fluence will be rotated in the same direction as the collimator is rotated (according to the definition in IEC 61217). The image will therefore appear to be rotated in the direction opposite to the specified angle. The definition of the direction of angles in I'mRT is in the other direction. Therefore the angle displayed in the **Field** tab and **Modification** tabs of the **Parameter** window will have the opposite sign.

To continue, see *8.1.6 Send to I'mRT Workspace*.

8.3. BrainLAB import

Select ***File:Import Data:Import BrainLAB*** to import BrainLAB dose and fluence files.

8.3.1. Patient Coordinate System

The BrainLAB coordinate system must be transformed to the OmniPro I'mRT (IEC) coordinate system:

BrainLab:

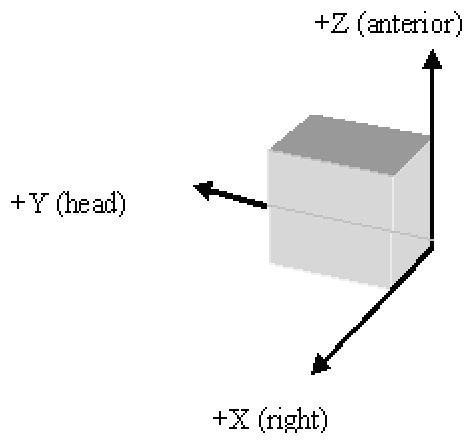

OmniPro I'mRT (IEC-61217):

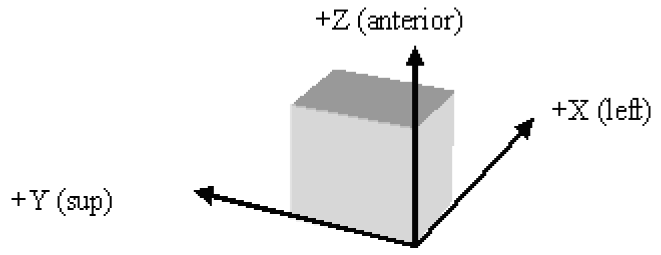

Standard planes and volumes

Standard planes and volumes use a patient-related coordinate system.

$X_{BrainLAB}$ becomes –XIEC

$Y_{BrainLAB}$ becomes YIEC

$Z_{BrainLAB}$ becomes ZIEC

8.3.2. Individual Beams (“Fluence Maps”):

The coordinate system used is fixed to the collimator, with the x-direction in leaf movement direction (a row in the file is parallel to the x-axis), and the y-direction perpendicular to the leafs (a column in the file is parallel to the y-axis). The first row gives the x-coordinate of each column running from –x to +x. The first column gives the y-coordinate of each row running from –y to +y. The isocenter is at position (x = 0, y = 0).

$X_{Collimator}$ (leaf movement direction) and $Y_{Collimator}$ (perpendicular to the leaves) are mapped one-to-one to X_{IEC} and Y_{IEC}.

NOTICE

IMPORTANT NOTICE

COORDINATES

If the file is viewed without using the coordinates given in the first row and column, you have to keep in mind the following fact: the y-coordinates are increasing with increasing row number, with the consequence that the rows have to be mirrored to get the correct coordinate system layout (x pointing to the right, y pointing upward).

The orientation of the exported 2D dose map in relation to the gantry and table depends on the LINAC convention. The table below shows the collimator angle used to get the leaves moving crossline in a way that the resulting dose map on a film will have the same orientation as in the file. This means that the leaves are moving from left to right in a standard beam’s eye view (gantry in upright position, table straight, observer standing on the table facing the gantry and looking downwards in beam direction):

MLC type	LINAC convention	Mounting option	Collimator angle
All MLCs	DIN<br>IEC<br>IEC1217		0°
VARIAN MLCs	VARIAN		180°
BrainLAB m3	DIN<br>IEC	right mounted	0°
BrainLAB m3	DIN<br>IEC	left mounted	180°
BrainLAB m3	VARIAN	left mounted	0°
BrainLAB m3	VARIAN	right mounted	180°
BrainLAB m3	DIN<br>IEC<br>VARIAN	front mounted	270°

8.3.3. File Format

For a description of the BrainLAB file format, see "BrainSCAN Dose Export Interface – Rev 2". 'Profiles' (chapter 5.1) and 'Misc. Planes' (5.3) are not supported.

8.3.4. User Interface

For a description of the user interface, see *8.4 Generic ASCII Import*.

8.3.5. Error Messages

Error messages are displayed as follows:

1st row: Name and path of the erroneous file.

2nd row: The number of the line where the error occurred, and the error number.

3rd row and forward: Descriptive text.

The following error messages may occur during the import of BrainLAB files:

Error #	Text	Description / Possible cause
1	Unexpected symbol: "symbol 1"<br><br>Expected: "symbol 2"	The format of the file is not correct as symbol 1 was found, whereas symbol 2 was expected in the given line.
5	"Token" expected	The format of the file is not correct as the Token was expected but not found in the given line.
7	Error when reading Object	A general error occurred when reading the named Object. Either the error cannot be specified in more detail, or the error is displayed in addition to a previous error.
8	"Type" not supported	The file has a valid format, but contains objects that are not supported by the import component.

NOTICE

IMPORTANT NOTICE

FLUENCE MAP IS SET TO ABSOLUTE DOSE

According to the BrainLAB descriptions, fluence files contain real dose values. Thus, an imported fluence map from BrainLAB is set to absolute dose.

8.4. Generic ASCII Import

8.4.1. Coordinate System

The generic ASCII import uses an IEC-61217 patient-related coordinate system. As this is the same as used in OmniPro I'mRT, the coordinate axes are assigned one-to-one from the import file to the main program.

8.4.2. File Format

8.4.2.1. Structure of the file

Item	Description
<opimrtascii>	File marker that identifies the file as an OmniPro I'mRT ASCII File.
Header	File contains one header that is valid for all subsequent bodies.
Body 1<br>Body 2<br>….etc.	File can contain various bodies.
</opimrtascii>	File marker that defines the end of the file.

8.4.2.2. Header

Identifier	Purpose / Remarks	Type / Possible values
<asciiheader>	Marks the beginning of the header	Mandatory
File version	Identifies the version of the file	Number → Set to 2
Separator	Defines the character(s) used to separate values in a matrix one from each other	Optional<br>Default: [Tab]<br>Any character or string (must be in double quotes) plus some special definitions:<br>[CR] ('\r') Carriage Return<br>[LF] Line Feed ('\n')<br>[CR][LF] CR/LF Pair ('\r\n')<br>[TAB] Tabulator ('\t')
Workspace name	Name of the original workspace when exported from OmniPro I'mRT	Optional<br>String<br>valid part → can be empty for import

Identifier	Purpose / Remarks	Type / Possible values
File name	Name of the original dataset file when exported from OmniPro I'mRT	Optional<br><br>String<br>valid part → can be empty for import
Image name	Name of the image inside a dataset when exported from OmniPro I'mRT	Optional<br><br>String<br>valid part → can be empty for import
Radiation type	Defines the radiation type<br><br>If none or any other radiation type than mentioned here is entered, it will be set to 'Unknown' in the resulting data.	Optional<br><br>String<br>X-Ray<br>XRay<br>Roentgen<br>Electrons<br>Photons<br>Neutrons<br>Protons<br>Co<br>Cobalt<br>Ir<br>Iridium<br>Cs<br>Caesium<br>Cesium
Energy	Energy value and unit<br><br>The unit is informational only. In the imported data the unit will be chosen according to the energy type:<br><br>kV for X-Ray/Roentgen<br><br>MV for Photons<br><br>MeV else	Optional<br><br>Number + string
SSD	Source Surface Distance	Optional<br><br>Number + string (mm or cm) *)
SID	Source Isocenter Distance	Optional<br><br>Number + string (mm or cm) *)
Field Size Cr	Field size in crossplane direction	Optional<br><br>Number + string (mm or cm) *)
Field size In	Field size in inplane direction	Optional<br><br>Number + string (mm or cm) *)

*) The length units for the four values above are informational only. The values are asserted to have the length unit as declared in the header.

Identifier	Purpose / Remarks	Type / Possible values
Data type	Type of the data.<br>If 'Absolute' or 'Abs. Dose' the Data Unit field of this header will be considered, otherwise the units are assumed to be 1/10%.	Optional. If not set, data will be considered as relative values.<br>String<br>Absolute<br>Abs. Dose<br>Relative<br>Rel. Dose<br>Fluence
Data factor	A factor that is multiplied with each data value when the file is imported. This is usually only meaningful in the export, when data is normalized, but can of course also be used for import data.	Optional.<br>Default: 1.0<br>Number
Data unit	Data unit (only useful for absolute dose values. Relative values are assumed to be 1/10%)	Optional. If not set, values are considered to be Gy for absolute dose, and 1/10% for relative dose.<br>String<br>mGy<br>cGy<br>rad<br>Gy<br>1/10% (only informational as this is the default for relative values)
Length unit	Length unit for some parameters in the header (see above) and the positions in the bodies.	Optional. If not set, length unit is considered to be mm.<br>Default: mm<br>String<br>mm<br>cm
Plane	Plane type according to IEC-61217 coordinate system.<br>Applies to all planes in the file.	Optional.<br>Default: XY<br>XY<br>XZ<br>YZ
No. of columns<br>or<br>Columns	Number of columns (horizontal) that applies to all subsequent bodies	Number

Identifier	Purpose / Remarks	Type / Possible values
No. of rows<br>or<br>Rows	Number of rows (vertical) that applies to all subsequent bodies.	Number
No. of bodies	Number of bodies (planes) in the file.	Optional<br>Default: 1<br>Number
Operators note	A comment, which is stored in the imported data up to 80 characters.<br>It must not contain line breaks. If the comment of an exported dataset contains line breaks they are substituted by a '||' each.	Optional<br>String
</asciiheader>	Marks the end of the header	Mandatory

8.4.2.3. User Defined Blocks

Identifier	Purpose / Remarks	Type / Possible values
<tagname>	Marks the beginning of a user-defined block.	tagname can contain any characters, > and new line excluded
…	An arbitrary number of lines with any content.<br>OmniPro I'mRT will skip these lines until the end tag is reached.	
</tagname>	Marks the end of a user-defined block.	

8.4.2.4. Body Header

Each body can be preceded by an arbitrary number of user-defined tagged blocks in the following format (ignored by OmniPro I'mRT):

Identifier	Purpose / Remarks	Type / Possible values
<tagname>	Marks the beginning of a user-defined block.	tagname can contain any characters, > and new line excluded
…	An arbitrary number of lines with any content.<br>OmniPro I'mRT will skip these lines until the end tag is reached.	
</tagname>	Marks the end of a user-defined block.	

8.4.2.5. Body

Identifier	Purpose / Remarks	Type / Possible values
<asciibody>	Marks the beginning of a body	Mandatory
Plane position	The position of the following plane in the 3rd direction.<br>The length unit is informational only. The values are asserted to have the length unit as declared in this header.	Optional.<br>Default: 0.0<br>Number + string (mm or cm)
Direction [length unit]	Direction and length unit of horizontal values inside the plane. The direction must be the first direction of the plane type defined in the header.<br>The length unit must equal the one defined in the header.	Optional. If set, the next line below must also be entered.<br>string<br>X [mm]<br>Y [mm]<br>Z [mm]<br>X [cm]<br>Y [cm]<br>Z [cm]
Positions in the first direction	All positions in the first direction in the defined length unit.	Optional. If not set, the "Spacing" value from the header is used, and the data is centered horizontally.<br>n: Numbers separated by the Separator defined in the header, where n must be the number of columns as defined above.

Identifier	Purpose / Remarks	Type / Possible values
Direction [length unit]	Direction and length unit of vertical values inside the plane. The direction must be the second direction of the plane type defined in the header.<br><br>The length unit must equal the one defined in the header.	Optional. If set, the first value of each following line must be a position.<br><br><u>string</u><br><br>X [mm]<br><br>Y [mm]<br><br>Z [mm]<br><br>X [cm]<br><br>Y [cm]<br><br>Z [cm]
Positions in the second direction + data values	The first value in each row is the position in the second direction in the defined length unit.<br><br>The subsequent numbers are the data values in the unit defined in the header.	1 Number (optional), followed by n numbers, separated by the Separator defined in the header each.<br><br>n must be the number of columns as defined above.<br><br>*)

*) The line above is repeated x times, where x must be the number of rows as defined in the header.

Identifier	Purpose / Remarks	Type / Possible values
</asciibody>	Marks the end of a body.	Mandatory

8.4.3. Example Files

8.4.3.1. Exported from OmniPro I'mRT

```
<opimrtascii>

<asciiheader>
File Version:        3
Separator:           ","
Workspace Name:      Test Workspace
File Name:           D:\OmniPro - IMRT\data\test.opd
Image Name:          Test Image
Radiation Type:      Electrons
Energy:              12.0 MeV
SSD:                 1000.0 mm
SID:                 1000.0 mm
Field Size Cr:       100.0 mm
Field Size In:       100.0 mm
Data Type:           Abs. Dose
Data Factor:         1
Data Unit:           mGy
Length Unit:         mm
Plane:               XY
No. of Columns:      10
No. of Rows:         13
Number of Bodies:    2
Operators Note:      This is a test file for importing Ascii data to OmniPro-ImRT
</asciiheader>

<asciibody>
Plane Position:     0.0 mm

X[mm]       -46.0 , -36.0 , -26.0 , -16.0 ,  -6.0 ,   4.0 ,  14.0 ,  24.0 ,  34.0 ,  44.0 ,
Y[mm]
  -62.5   ,     0 ,     0 ,     0 ,     0 ,     0 ,     0 ,     0 ,     0 ,     0 ,     0 ,
  -52.5   ,     0 ,     0 ,     0 ,     0 ,     0 ,     0 ,     0 ,   200 ,     0 ,   140 ,
  -42.5   ,     0 ,     0 ,     0 ,     0 ,     0 ,    60 ,   320 ,   660 ,   600 ,   320 ,
  -32.5   ,     0 ,     0 ,     0 ,     0 ,    60 ,   460 ,   400 ,   860 ,   660 ,   140 ,
  -22.5   ,     0 ,     0 ,   940 ,  1000 ,   140 ,     0 ,    60 ,     0 ,   800 ,   260 ,
  -12.5   ,     0 ,   200 ,   200 ,   320 ,     0 ,     0 ,     0 ,   320 ,   660 ,   260 ,
   -2.5   ,     0 ,     0 ,   600 ,   260 ,   460 ,   400 ,   200 ,   320 ,   260 ,   260 ,
    7.5   ,     0 ,     0 ,    60 ,   660 ,    60 ,   460 ,    60 ,   860 ,     0 ,   540 ,
   17.5   ,     0 ,   200 ,   320 ,   660 ,   460 ,     0 ,     0 ,   140 ,  1000 ,   140 ,
   27.5   ,     0 ,   400 ,   260 ,   940 ,   400 ,    60 ,     0 ,     0 ,   400 ,   260 ,
   37.5   ,     0 ,     0 ,   720 ,   540 ,     0 ,   720 ,     0 ,   320 ,   400 ,     0 ,
   47.5   ,     0 ,     0 ,   320 ,     0 ,     0 ,   400 ,  1000 ,   940 ,   320 ,     0 ,
   57.5   ,     0 ,     0 ,     0 ,     0 ,     0 ,    60 ,   400 ,   320 ,   140 ,     0 ,
</asciibody>


<MyFileHeader>

Operator:    A. Dent
My 1st value: 42
…
</MyFileHeader>
```

→ **Optional, user-defined block that will be ignored by OmniPro I'mRT**

```
<MyBodyHeader>                    → Optional, user-defined block that will be ignored by OmniPro I'mRT
...
Information on the following body
...
</MyBodyHeader>


<asciibody>
Plane Position:   0.0 mm


X[mm]       -46.0 , -36.0 , -26.0 , -16.0 ,  -6.0 ,   4.0 ,  14.0 ,  24.0 ,  34.0 ,  44.0 ,
Y[mm]
  -62.5   ,     0 ,     0 ,     0 ,     0 ,     0 ,     0 ,     0 ,     0 ,     0 ,     0 ,
  -52.5   ,     0 ,     0 ,     0 ,     0 ,     0 ,     0 ,     0 ,   200 ,     0 ,   112 ,
  -42.5   ,     0 ,     0 ,     0 ,     0 ,     0 ,    60 ,   320 ,   660 ,   600 ,   256 ,
  -32.5   ,     0 ,     0 ,     0 ,     0 ,    60 ,   460 ,   400 ,   860 ,   660 ,   112 ,
  -22.5   ,     0 ,     0 ,   940 ,  1000 ,   140 ,     0 ,    60 ,     0 ,   800 ,   208 ,
  -12.5   ,     0 ,   200 ,   200 ,   320 ,     0 ,     0 ,     0 ,   320 ,   660 ,   208 ,
   -2.5   ,     0 ,     0 ,   600 ,   260 ,   460 ,   400 ,   200 ,   320 ,   260 ,   208 ,
    7.5   ,     0 ,     0 ,    60 ,   660 ,    60 ,   460 ,    60 ,   860 ,     0 ,   432 ,
   17.5   ,     0 ,   200 ,   320 ,   660 ,   460 ,     0 ,     0 ,   140 ,  1000 ,   112 ,
   27.5   ,     0 ,   400 ,   260 ,   940 ,   400 ,    60 ,     0 ,     0 ,   400 ,   208 ,
   37.5   ,     0 ,     0 ,   720 ,   540 ,     0 ,   720 ,     0 ,   320 ,   400 ,     0 ,
   47.5   ,     0 ,     0 ,   320 ,     0 ,     0 ,   400 ,  1000 ,   940 ,   320 ,     0 ,
   57.5   ,     0 ,     0 ,     0 ,     0 ,     0 ,    60 ,   400 ,   320 ,   140 ,     0 ,
</asciibody>

<MyBodyHeader>         → Optional, user-defined block that will be ignored by OmniPro I'mRT
…
Information on the following body
…
</MyBodyHeader>



<asciibody>
Plane Position:     20.0 mm

X[mm]       -46.0 , -36.0 , -26.0 , -16.0 ,  -6.0 ,   4.0 ,  14.0 ,  24.0 ,  34.0 ,  44.0 ,
Y[mm]
  -62.5   ,     0 ,     0 ,     0 ,     0 ,     0 ,     0 ,     0 ,     0 ,     0 ,     0 ,
  -52.5   ,     0 ,     0 ,     0 ,     0 ,     0 ,     0 ,     0 ,   160 ,     0 ,   112 ,
  -42.5   ,     0 ,     0 ,     0 ,     0 ,     0 ,    48 ,   256 ,   528 ,   480 ,   256 ,
  -32.5   ,     0 ,     0 ,     0 ,     0 ,    48 ,   368 ,   320 ,   688 ,   528 ,   112 ,
  -22.5   ,     0 ,     0 ,   752 ,   800 ,   112 ,     0 ,    48 ,     0 ,   640 ,   208 ,
  -12.5   ,     0 ,   160 ,   160 ,   256 ,     0 ,     0 ,     0 ,   256 ,   528 ,   208 ,
   -2.5   ,     0 ,     0 ,   480 ,   208 ,   368 ,   320 ,   160 ,   256 ,   208 ,   208 ,
    7.5   ,     0 ,     0 ,    48 ,   528 ,    48 ,   368 ,    48 ,   688 ,     0 ,   432 ,
   17.5   ,     0 ,   160 ,   256 ,   528 ,   368 ,     0 ,     0 ,   112 ,   800 ,   112 ,
   27.5   ,     0 ,   320 ,   208 ,   752 ,   320 ,    48 ,     0 ,     0 ,   320 ,   208 ,
   37.5   ,     0 ,     0 ,   576 ,   432 ,     0 ,   576 ,     0 ,   256 ,   320 ,     0 ,
   47.5   ,     0 ,     0 ,   256 ,     0 ,     0 ,   320 ,   800 ,   752 ,   256 ,     0 ,
   57.5   ,     0 ,     0 ,     0 ,     0 ,     0 ,    48 ,   320 ,   256 ,   112 ,     0 ,
</asciibody>

</opimrtascii>
```

8.4.4. File Fulfilling the Minimum Requirements

<opimrtascii>

<asciiheader>
File Version: 3 → **Not mandatory, but should be present to avoid problems with future versions.**
No. of Columns: 10
No. of Rows: 10
Spacing: 7.619 → **Not mandatory. If not set, 1 mm as default will be used.**
</asciiheader>

<asciibody>

341	823	847	811	823	823	823	811	870	800
376	894	929	835	823	811	823	835	917	752
364	894	917	823	776	741	800	823	917	670
364	882	894	788	741	729	729	788	905	858
694	870	811	741	705	705	717	776	858	905
882	870	764	682	647	658	682	764	858	964
929	835	694	588	564	576	623	741	882	1000
717	647	482	447	423	435	494	623	752	917
658	611	470	364	294	317	411	517	670	811
717	670	576	505	423	364	423	529	658	788

</asciibody)

</opimrtascii>

8.4.5. User Interface for ASCII and BrainLAB Import

The user interfaces of the ASCII and the BrainLAB Import look exactly the same. They only differ in the header information that is displayed.

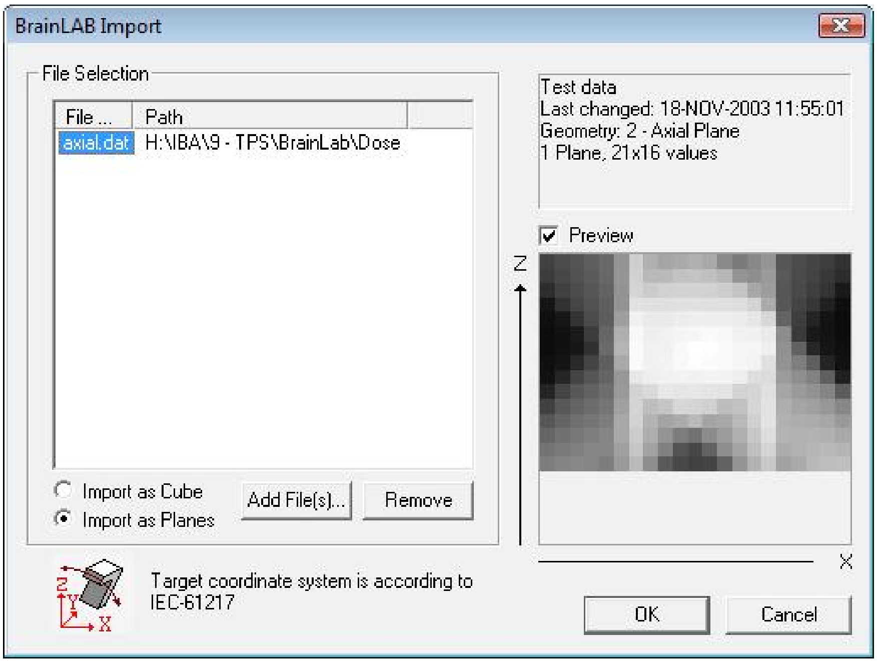

File Name and **Path** display the file name and folder location of the files selected for import.

In the pane to the right of the **File Selection** pane, header information on the selected file is displayed. If more than one file is selected, the pane displays the text **Multiple Selection**. If a file that has not the appropriate format is selected, the text **File has an invalid format** is displayed.

The preview of the selected file can be switched on or off, by marking or unmarking the **Preview** checkbox. The loading time of large files can be reduced by switching the preview off.

The preview pane displays a preview image of the currently selected file and the coordinate axes. If the **Preview** checkbox is unchecked, or more than one file or a file containing more than one plane is selected, the preview pane is empty.

Select import as a 3d cube or as single planes with the radio-buttons **Import as Cube** and **Import as Planes**.

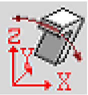
This image shows the target coordinate system in OmniPro I'mRT, which is always according to IEC-61217.

The **Add File(s)** button opens a standard **Open** dialog.

The **Remove** button removes the selected files from the list.

If **OK** is clicked, all files in the list that have the correct format are being imported. For each file the dialog below is opened. Select the target dataset, and the scaling of the image.

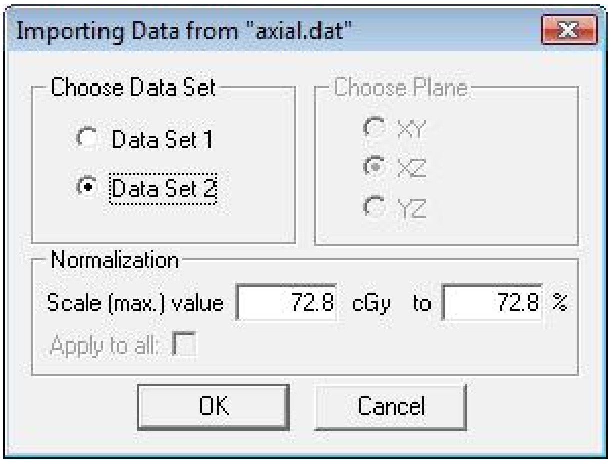

The name of the current dataset is displayed in the caption. In the edit box the user can enter the percentage value that represents the maximum value of the imported image, as shown in the left (read only) edit box.

8.4.6. Header Information for OmniPro I'mRT ASCII Files

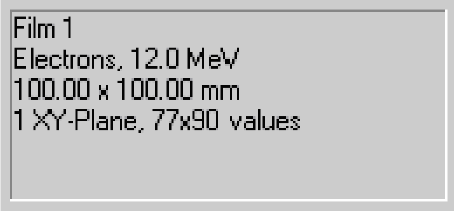

Line #	According to field(s) in ASCII file
1	Image Name
2	Radiation type / Energy
3	Field Size In / Field Size Cr
4	Number of Bodies / Plane / No. of Columns / No. of Rows

8.4.7. Header Information for BrainLAB Files

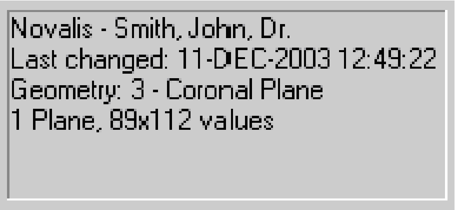

Line #	According to field(s) in BrainLAB file
1	Patient
2	Last changed
3	Geometry
4	Number of Planes / Number of Rows / Number of Columns

8.4.8. Error Messages

Error messages are displayed as follows:

1st row: Name and path of the erroneous file.

2nd row: The number of the line where the error occurred, and the error number.

3rd row and forward: Descriptive test.

The following error messages may occur during the import of generic ASCII files:

Error #	Text	Description / Possible cause
1	Unexpected symbol: "symbol 1"<br>Expected: "symbol 2"	The format of the file is not correct, as symbol 1 was found whereas symbol 2 was expected in the given line.
5	"Token" expected	The format of the file is not correct as the Token was expected but not found in the given line.
7	Error when reading Object	A general error occurred when reading the named Object. Either the error cannot be specified in more detail, or the error is displayed in addition to a previous error.

Error #	Text	Description / Possible cause
8	"Type" not supported	The file has a valid format, but contains objects that are not supported by the import component.
1000	Wrong number of planes<br>m declared, n defined	The number of planes, as declared in the "Number of Bodies" key in the header, is different from the actual number of defined planes.
1001	Direction in body (dir) is not corresponding to the plane defined in the header (plane)	A direction in the matrix (e.g. X[mm]) is not corresponding to the plane that is defined in the "Plane:" key in the header. Either columns or rows may have been swapped, or the direction is not even part of the plane.
1002	Length unit in body (lu1) is not corresponding to the one defined in the header (lu2)	The length unit, used to describe a direction in the matrix (e.g. X[mm]), is different from the one that is defined in the "Length Unit:" key in the header.

8.5. TomoTherapy ASCII import

Select ***File:Tomo Therapy:Import ASCII File..*** to open the **TOMO Ascii Import** dialog.

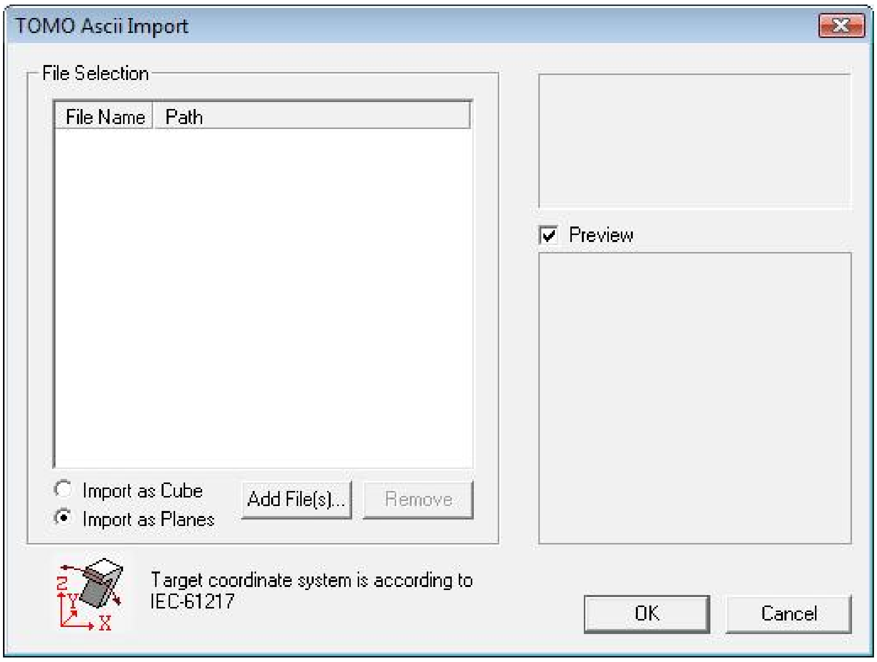

Click **Add File(s)..** Select **Import as Cube** or **Import as Planes**, and select one or more *.header files.

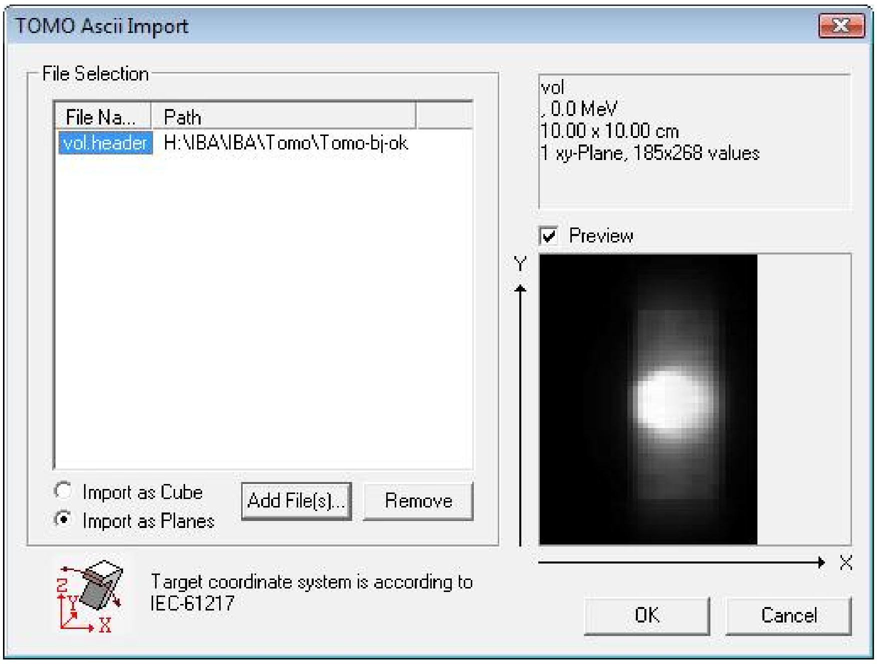

Click **OK**. Select data set and, if necessary edit the scaling value in the **Importing Data** dialog.

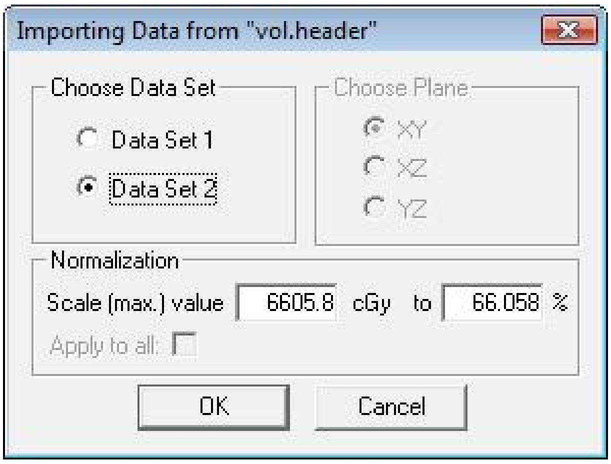

Click **OK**. The **Number of Fractions** dialog opens.

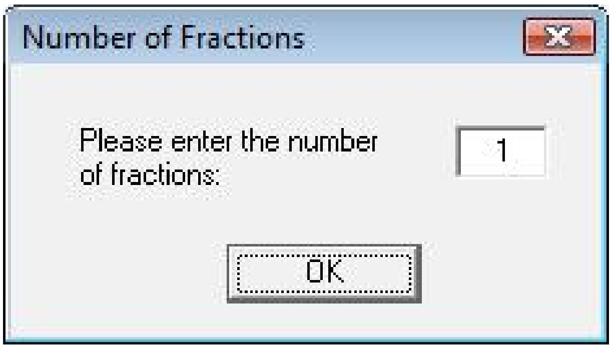

Total absolute dose values are imported, and therefore the user must set the number of fractions in this dialog. The dose values will be divided by the entered number.

Click **OK** to finalize the import to the I’mRT Workspace.

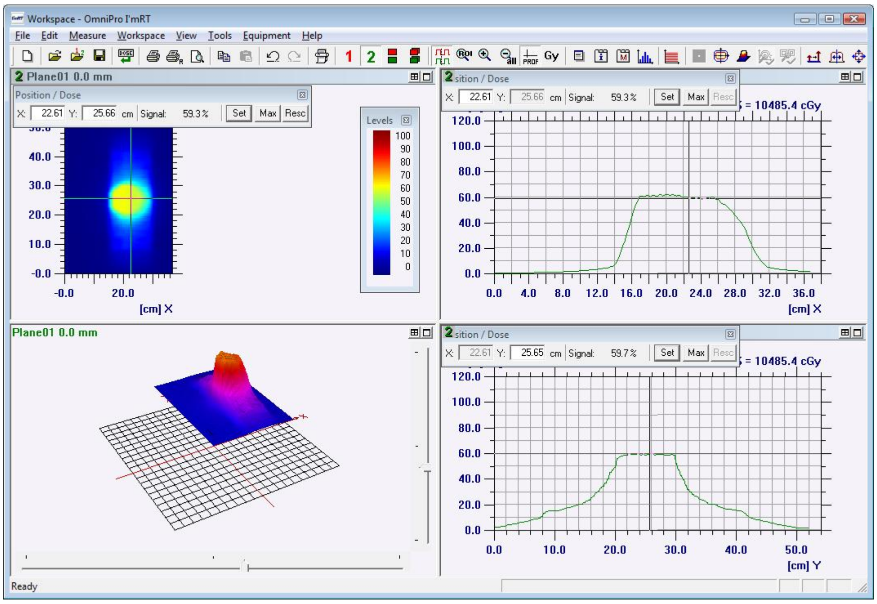

9. Data Analysis

9.1. Convert Grid

OmniPro I'mRT is designed for operation with various data sources (MatriXX, I'mRT QA, radiographic and radiochromic film, EPID, TPS…), which have inherently very different pixel size and numbers. It is often desirable to make comparisons between different data sets using the same grids, which might be accomplished by integration of a fine grid to a more coarse one, or by linear interpolation of a coarse grid down to a finer one.

In lower resolution grids, it is not sufficient to specify only the width of the grid, but also the position of the center of the grid. OmniPro I'mRT allows the specification of two modes:

- One center line: The center of the pixel is in the center of the array
- Two center lines: The touching corners of four pixels are in the center of the array

For the reconstruction of finer grids, a two-dimensional linear interpolation is used between the points of the coarser grid.

The I'mRT MatriXX measures with cylindrical detectors in a 7.62x7.62 mm grid. As an approximation, the dose measured in one pixel can be considered as the integral over a square with the side length 7.62 mm.

For comparison with the treatment planning data, TPS dose data have to be evaluated with the same grid.

Select ***Convert Grid*** in the ***Edit*** menu. Select the appropriate interpolation method (either **Linear** or **Cubic spline)**.

NOTICE

IMPORTANT NOTICE

CUBIC SPLINE

It is not recommended to use the setting "cubic spline" for interpolation.

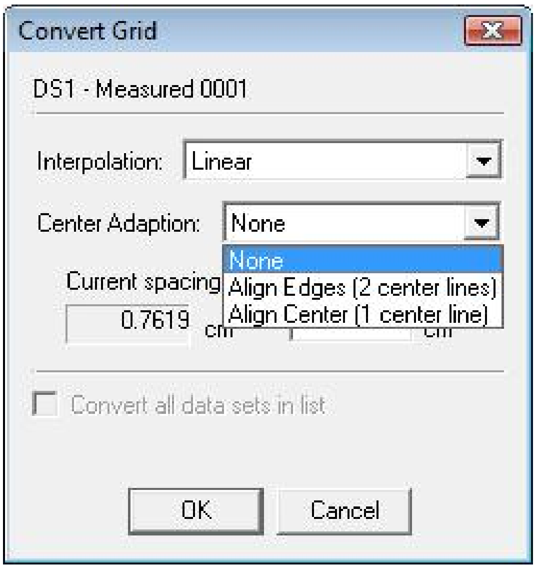

More details regarding the interpolation algorithms are given in the chapter *Algorithms*.

Select the appropriate center adaption. The following center alignments are possible:

1. **Align center (1 center line)**: The coordinate system will be shifted (if necessary) such that there is one pixel line in each direction directly along the zero lines.

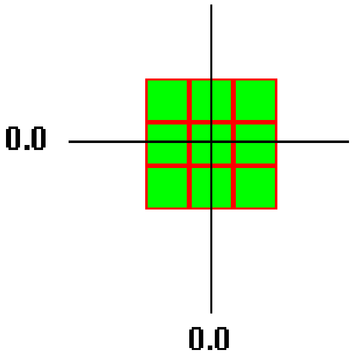

Center Line

2. **Align edges (2 center lines)**: The coordinate system will be shifted (if necessary) such that there are two pixel lines in each direction that are adjacent directly along the zero lines.

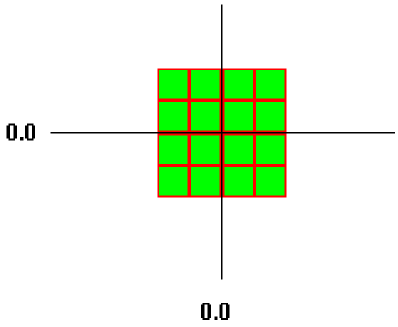

Center Lines

3. **None**: The most convenient method is used. This option should only be used when the alignment is not important (e.g. when having a very high resolution).

4. Enter the desired grid resolution in **New spacing**. Minimum value allowed is 0.01 cm.

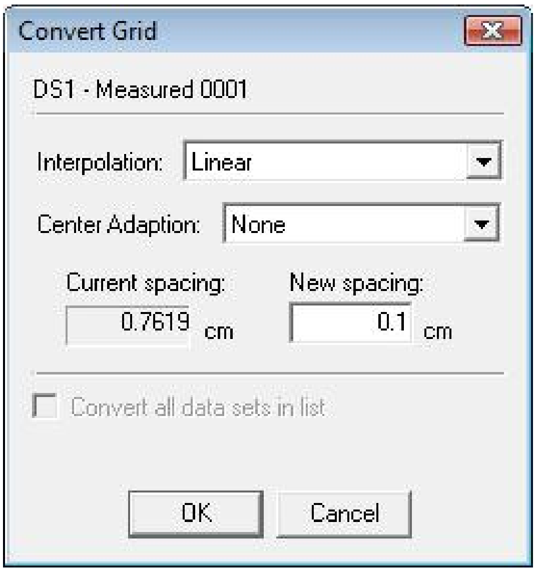

5. Mark **Convert all data sets in list** if all data sets in the **Field List** shall be converted. Leave this unchecked if only the chosen one shall be converted.

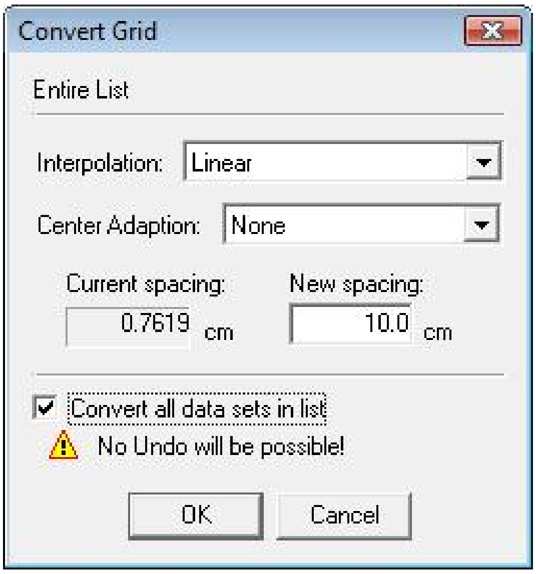

NOTICE

IMPORTANT NOTICE

UNDO NOT POSSIBLE

It is not possible do undo this selection once you have clicked **OK**.

9.2. Modifying Data

9.2.1. Add Constant Value

Use this tool to add a constant value to an array. Select ***Add constant value*** in the ***Edit*** menu.

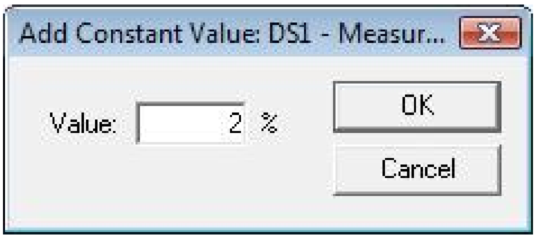

To remove it, open the dialog and subtract the corresponding value.

9.2.2. Rescale

To rescale (renormalize) the dose values, select the menu command ***Edit:Rescale***, or use the right-click command ***Rescale***, or click the **Rescale** tool bar button. The **Rescale** dialog opens.

This command can be used when you want to rescale two dose distributions with different normalization to the same relative signal level.

1. Click **Synchronize Views**.
2. Display the region of interest (ROI). Change it in position and size in such a way that the dose distribution inside the ROI is quite homogeneous.
3. Rescale the ROI average of both dose distributions to a common value.

NOTICE

IMPORTANT NOTICE

DATA RESCALED

The **Rescale** command affects the data of the active pane only, even if **Workspace:Synchronize Views** is selected.

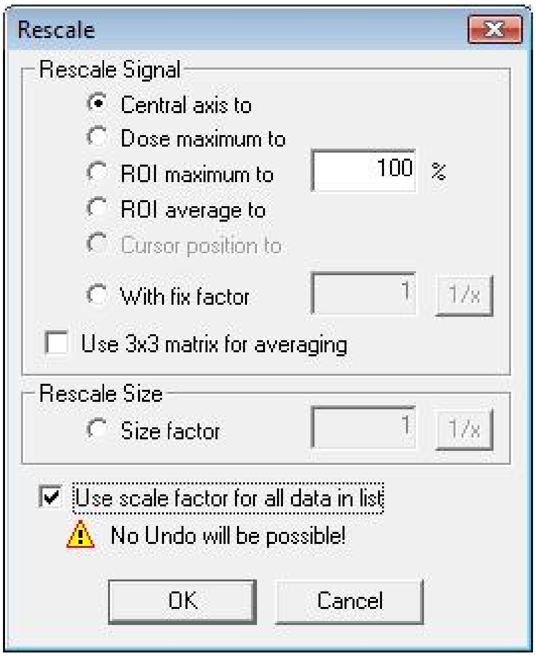

Rescale Signal section:

Select **Rescale Signal** method, and enter a value.

Central axis to: Rescales the data so that the dose at coordinate origin (for intensity map measurements the central axis (CAX)) becomes equal to the dose value defined by the user.

Dose maximum to: Rescales the data in such a way that the dose at dose maximum becomes equal to the dose value defined by the user.

ROI maximum to: Rescales the maximum value in the user selected ROI to the entered dose value.

ROI average to: Rescales the medium dose value in the user selected ROI to the entered dose value.

Cursor position to: Rescales the dose in the cursor position to the entered dose value. Activate the profile cursor to enable the **Cursor position** option.

With fix factor: Rescales the data with a fixed factor.

Use 3x3 matrix for averaging: (enabled only if **Central axis to** or **Cursor position to** is selected): Instead of taking only one pixel, the average value of a 3x3pixel matrix is used for rescaling.

Rescale Size section:

Size Factor: Rescales the geometric scale with the entered factor.

Use scale factor for all data in list: If this option is selected, all data in the field list will be rescaled with the same factor. If it is not selected, only the active field will be rescaled.

NOTICE

IMPORTANT NOTICE

UNDO NOT POSSIBLE

Once **OK** has been clicked, it is not possible to undo the selection **Use scale factor for all data in list**.

9.2.3. CAX Correction

If you have square fields or rectangular fields (measurements normal to the beam axis, e.g. fluence measurements), you can check how much the geometric center of the field is off the origin in this plane. To check (or adjust) the central axis (CAX) deviation, select the menu command ***Edit:Correct Origin***, or use the right-click command ***Correct Origin***, or click the **Correct Origin** tool bar button. The **Correct Origin** dialog opens.

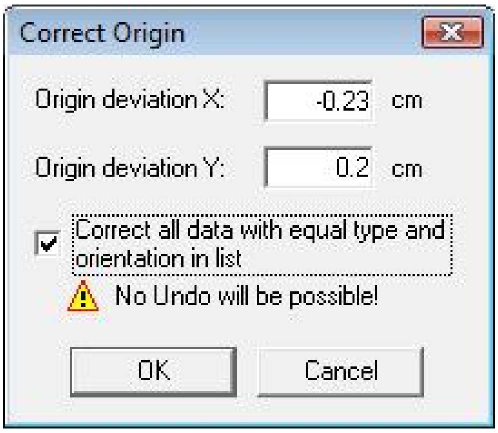

NOTICE

IMPORTANT NOTICE

DATA ADJUSTED

The **Correct Origin** command affects the data of the active pane only, even if **Workspace:Synchronize Views** is selected.

The values displayed are the actual off-centre displacements in inline or crossline directions. These values were calculated using the 50% CAX values on the main axis.

To automatically move the image so that the CAX deviation is 0, click **OK**. The specified image will be moved by the CAX deviation multiplied with -1. If opening this dialog again, the CAX deviation will show 0.

NOTICE

IMPORTANT NOTICE

IRREGULAR FIELD SHAPE

If the field has an irregular shape, it might be necessary to perform this operation several times before the data is centered.

To move data manually, enter the desired distance and multiply it with -1. Click **OK**.

Correct all data with equal type and orientation in list: If this option is checked, all fields that are in the list of the current dataset and have the same orientation (x/y, x/z or y/z) as the active image, will be corrected.

NOTICE

IMPORTANT NOTICE

UNDO NOT POSSIBLE

Once **OK** has been clicked, it is not possible to undo the selection **Correct all data with equal type and orientation in list**.

9.2.4. Move

This function is used to register two datasets manually. One dataset is moved relative to the other dataset, until the dose distributions overlay in the correct way.

Select the dataset to be moved.

Select ***Edit:Move Data*** to open the **Move Data** dialog.

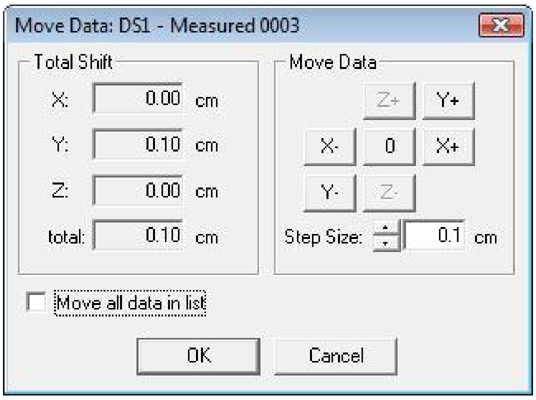

Select the step size.

Press one of the move buttons: The selected data will be moved into the selected direction with the selected step size.

Tip! You get a clear visual control if the **Compare Isodoses** view is shown in the **Compare** pane.

Total shift: Displays the shift due to the **Move data** operation in all three dimensions and in total.

Move all data in list: If selected, all data in the field list will be moved.

NOTICE

IMPORTANT NOTICE

UNDO NOT POSSIBLE

Once **OK** has been clicked, it is not possible to undo the selection **Move all data in list**.

9.2.5. Turn Array

To turn the 2D dose distributions (2D arrays), use the **Edit:Turn Array** command. Use this function e.g. in a case where the film was misaligned in the body phantom, and had not been aligned after scanning.

Example:

A film was tilted in a body phantom, and had not been aligned after scanning. Two markers, or the phantom border parallel to the phantoms x, y or z axis, are clearly seen on the image.

Right-click in the **Array** pane and select ***Turn Array***. The **Turn Array** dialog opens. You can also use the ***Edit:Turn Array*** command, or click the **Turn Array** toolbar button 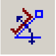.

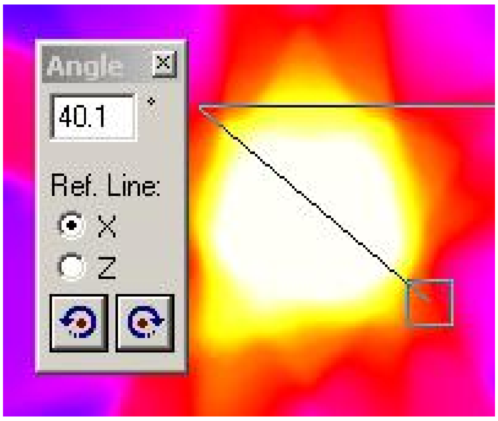

Select the orientation of the reference line with the **Ref. Line** radio-buttons.

Move the intersection of the two legs to one of the markers (or onto the border).

Move the square onto the other marker (alternatively so that the movable leg is parallel to the border).

Select left or right hand turn with the buttons 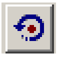 and 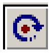.

9.2.6. Smooth

To choose algorithm for smoothing the selected measurement data, select the menu command ***Edit:Smooth***, or use the right-click command ***Smooth***, or click the **Smooth** tool bar button. The **Smooth** dialog opens.

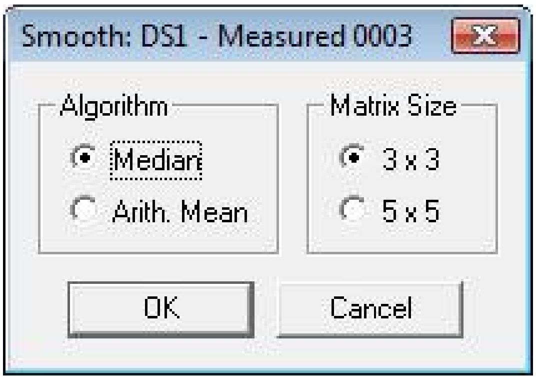

NOTICE

IMPORTANT NOTICE

SMOOTHED DATA

The **Smooth** command affects the data of the active pane only, even if **Workspace:Synchronize Views** is selected.

NOTICE

IMPORTANT NOTICE

SMOOTHED DATA

Smoothing is performed only on the field displayed on the screen, not on all fields in the file.

Select optional algorithm and matrix size and click **OK**.

Algorithm section:

Median: Retains the slope of a scan but not its maximum. Median is a good method when trying to eliminate measuring faults in single pixels, like noise values due to scattered irradiation hitting the CCD camera.

Arith. mean: This algorithm cannot conserve the maximum and the slope of a scan.

Matrix Size section:

Select the size of the matrix that will be used for mean value calculation.

9.2.7. Flip Image

The orientation of the image can be changed.

9.2.7.1. Flip Horizontal

Select ***Edit:Flip Horizontal*** to flip the image 180° horizontally.

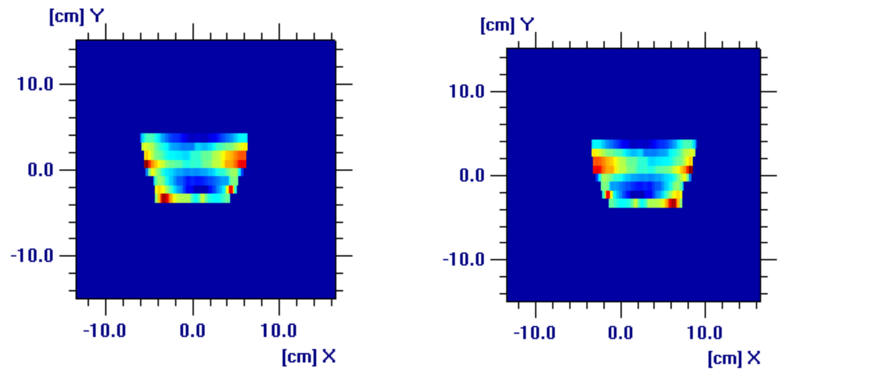

Original image *Flip horizontal*

9.2.7.2. Flip Vertical

Select ***Edit:Flip Vertical*** to turn the image 180° vertically.

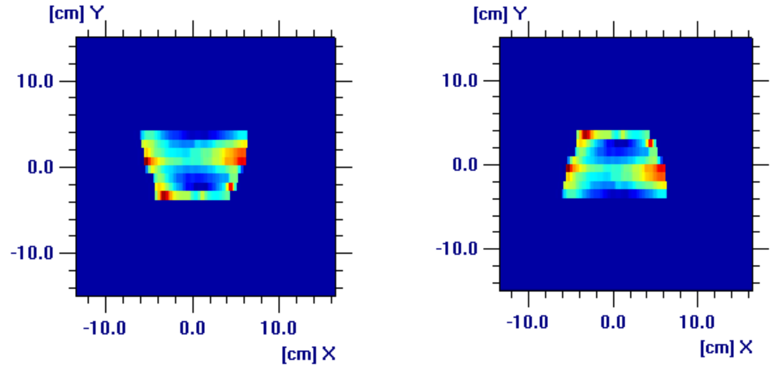

Original image *Flip vertical*

9.2.8. View ROI

Click the button 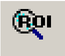 in the main toolbar, or select ***View:Zoom ROI***, to view the region of interest (ROI).

To return to the original view, click the button 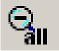, or select ***View:Unzoom***.

9.2.9. Cut ROI

Select ***Edit:Cut ROI*** to remove all data not belonging to the region of interest (ROI) from the workspace. Data can be saved in this view.

To return to the original view, select ***Edit:Undo Cut ROI***.

9.2.10. Change Plane

Select ***Edit:Change Plane*** to change the orientation of the image.

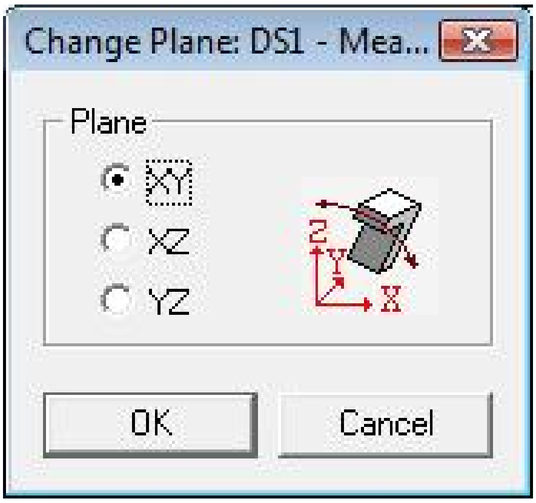

9.2.11. Filter

A filter matrix may be applied to the image.

Select ***Filter*** in the ***Edit*** menu to open the **Filter Matrix** dialog.

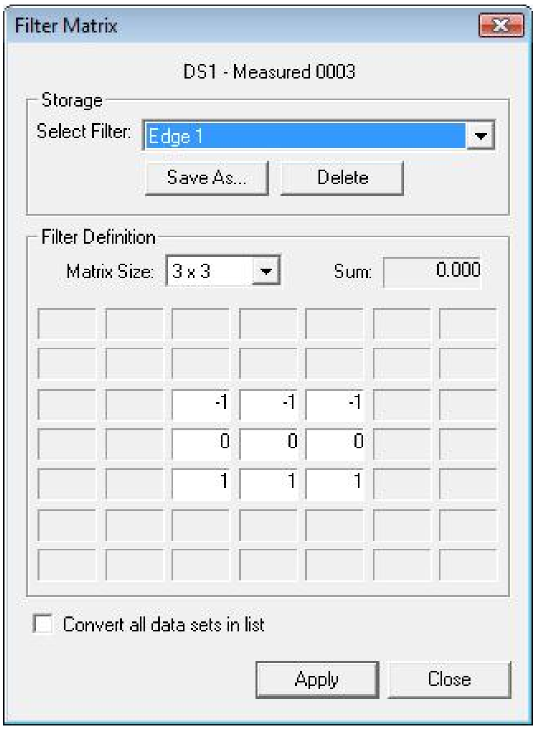

Select a previously defined filter, or define a new filter.

Example:

An edge filter, defined as below, with the matrix size 3 × 3, is applied on an area with the following pixels:

...	...	...	...	...
...	121	123	123	...
...	130	135	140	...
...	137	144	149	...
...	...	...	...	...

Edge filter:

-1	-1	-1
0	0	0
1	1	1

The sum of the matrix values is 0. In this special case the sum of the calculated values will be divided by 1. The center pixel will be recalculated as follows:

$(-1 \cdot 121 + -1 \cdot 123 + -1 \cdot 123 + 0 \cdot 130 + 0 \cdot 135 + 0 \cdot 140 + 1 \cdot 137 + 1 \cdot 144 + 1 \cdot 149) / 1 = \mathbf{63}$

The selected filter can be applied simultaneously to all data sets in the field list. Mark the checkbox **Convert all data sets in list**, and click **Apply**.

NOTICE

IMPORTANT NOTICE

UNDO NOT POSSIBLE

Once **Apply** has been clicked, it is not possible to undo the selection **Covert all data sets in list**.

9.3. Macros

Several operations for data modification can be combined in a macro. The macro may be applied to one field or to a whole field list.

The parameters displayed below can be combined in a macro.

Function	Parameters	Value	Example
ADD	<VALUE>	Percentage	ADD, 5%
RESCALE	<METHOD><br><VALUE>	DMAX, CAX, ROIMAX, ROIAVG, FACTOR, VALUE	RESCALE, DMAX, 100%
CORR_ORI	<X>, <Y>	X, Y coordinates<br>or<br>Default values from CAX correction	CORR_ORI, 10, 12
MOVE	<X>, <Y>, <Z>	X, Y, Z coordinates	MOVE, 0, 0, 5
TURN	<ANGLE>	Angle	TURN, 2
SMOOTH	<ALGORITHM><br><SIZE>	Median, ArithMean<br>3x3, 5x5	SMOOTH, Median, 3x3
FLIP	<DIRECTION>	H, V	FLIP, H
CUT_ROI	<X>, <Y>	X, Y coordinates	CUT_ROI, 100,100
CHANGE_PLANE	<PLANE>	XY, XZ, YZ	CHANGE_PLANE, XZ
CONVERT_GRID	<INTERPOL><br><CENTERADAPTION><br><GRID>	Linear, Spline, None<br>Edges, Center<br>Grid size	CONVERT_GRID, Linear, None, 1
FILTER	<FILTER_NAME>	Name of saved filter	FILTER, “Gaussian Blur”
ECHO	<STATUSBAR>,<br><STRING>	Info text to be displayed in status bar	ECHO, STATUSBAR, “Hello ImRT World”
MSGBOX	<STRING>	Text to be displayed as a MsgBox	MSGBOX, “Hello ImRT World”
BEEP		Computer will beep	BEEP

9.3.1. Define a Macro

1. Select a dataset.
2. Select ***Macro:Edit Macros*** in the ***Edit*** menu.
3. Click **New** in the **Macros** dialog. The **New Macro** dialog opens.

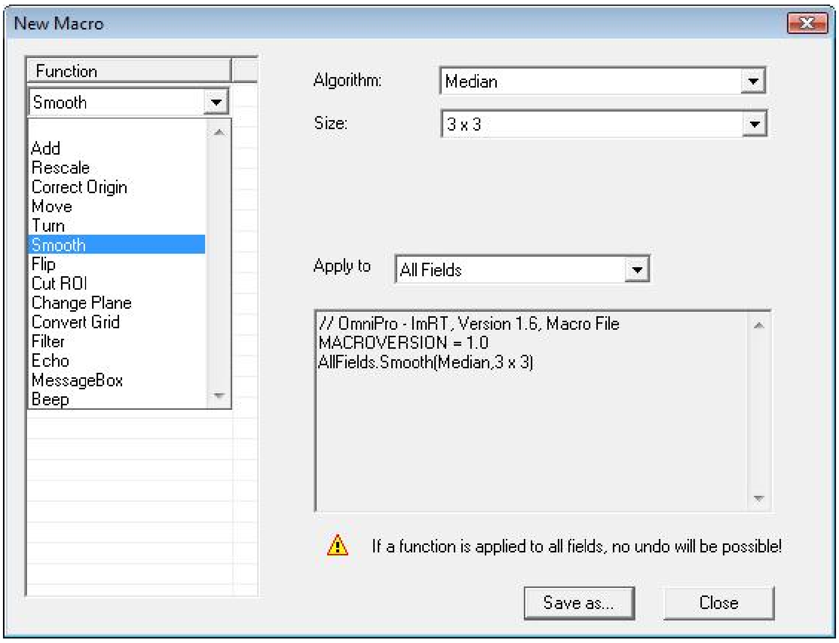

4. Select a function.

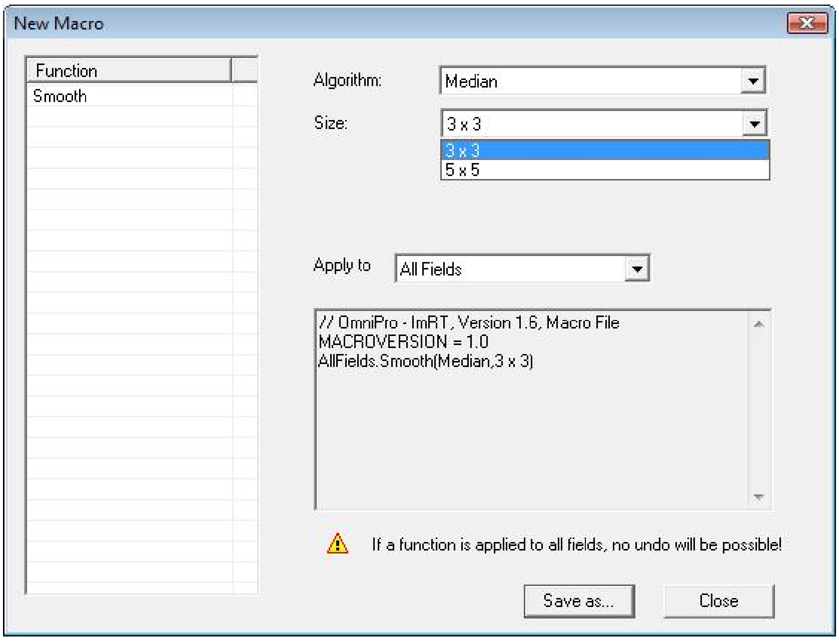

5. Define the parameters for the function. Define whether the function shall be applied to the active field, or to all fields in the **Field List**.

NOTICE

IMPORTANT NOTICE

UNDO NOT POSSIBLE

Once **Save As** has been clicked, it is not possible to undo the selection **Apply to All Fields**.

6. Select and define the other functions that you want to include in the macro, and click **Save as**.

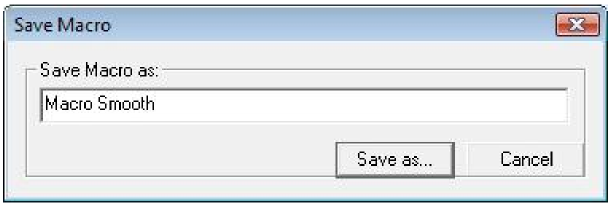

7. Define a name for the macro, and click **Save as**.
8. Click **Close** in the **New Macro** dialog. The **Macros** dialog opens.

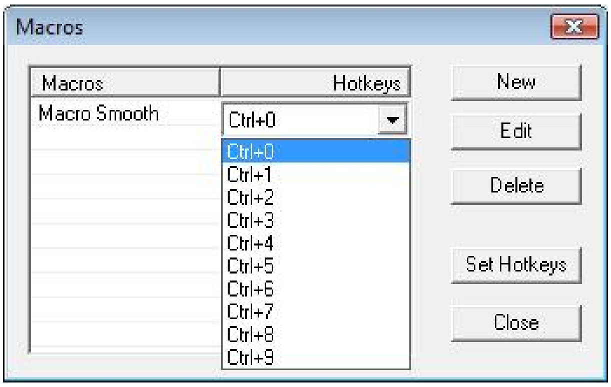

9. Select a hotkey and click **Set Hotkeys**.
10. Click **Close** to leave the dialog.

9.3.2. Apply a Macro

1. Select a field, or the complete field list, and select ***Macro*** in the ***Edit*** menu.
2. Select the macro you want to apply. A macro can also be applied by using the corresponding hotkey.

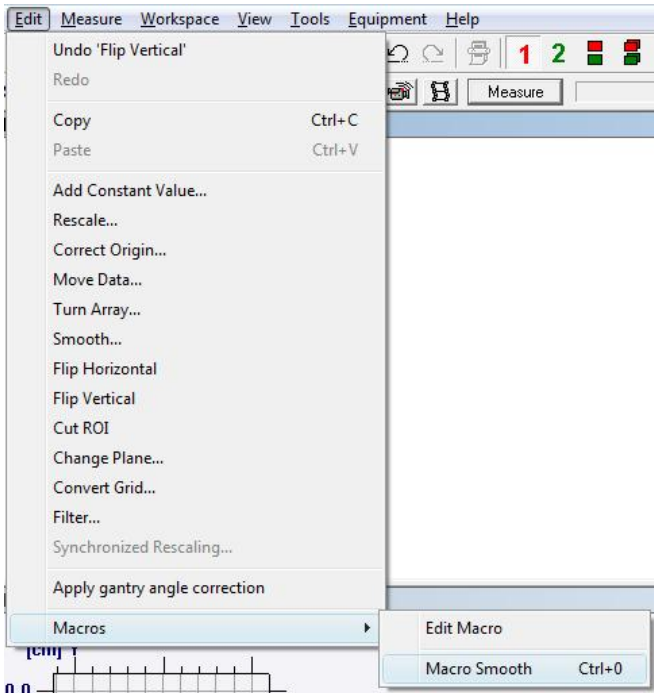

The selected macro will be applied on all selected fields. The operation can be aborted by pressing <ESC>.

9.4. Analyzing One Single Data Set

You can analyze a single data set in the following ways:

- Evaluate isodose curves (e.g. distances between different isodoses).
- Evaluate symmetry and flatness along the main axes.

To select the data set to analyze, select 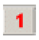 or 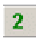 for data set 1 or 2 respectively. The opened file is displayed in a 2D array view in the upper left pane, and in an isodoses or a 3D view in the lower left pane. The profiles along the x- and y-axis are displayed in the **Profile** panes.

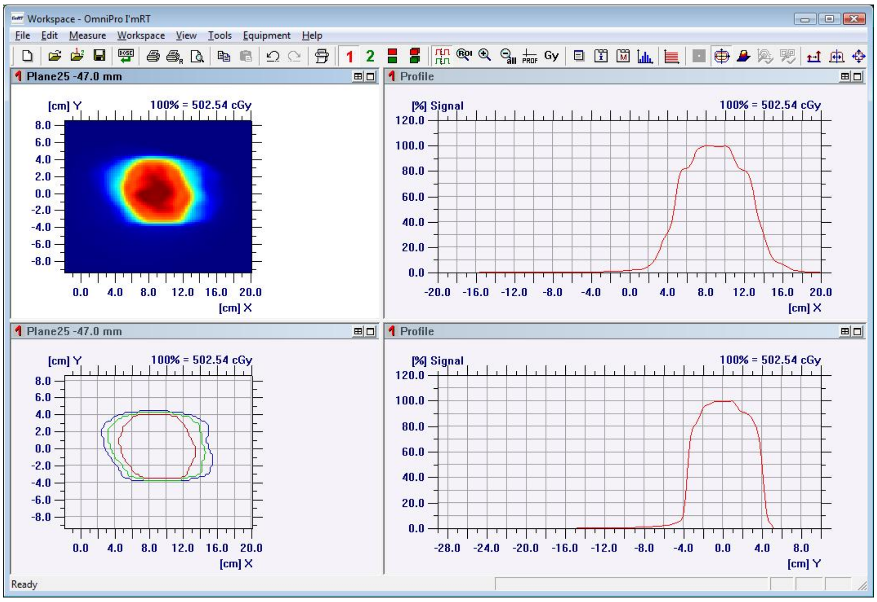

9.4.1. Isodoses

1. To analyze isodoses, follow the instructions below.
2. Click 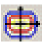 to display the isodose view.
3. Click 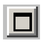 to maximize the pane size.
4. Select the right-click command ***Isodose Levels***. The **Isodose Values** dialog opens.

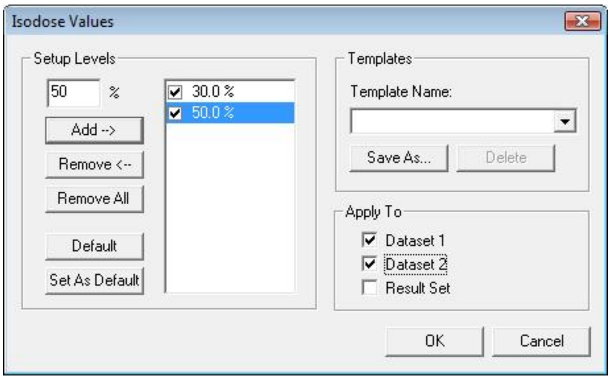

5. Create a list with the desired isodose levels by entering the values in the text box and clicking **Add**. Click **OK** to finish.

   Check/uncheck the checkboxes in the **Apply to pane**.

8. If the entered data shall be used as default values, click the **Set As Default** button.
9. Data can also be saved as a template. Click the **Save As** button and enter a name in the **Save Template** dialog.

Option: To view a legend of the isodose levels, select the right-click command ***Show Legend***.

Option: Zoom the area of interest. Move the mouse to the selected pane. A cross will appear. Drag the cross, without pressing a mouse button, to a corner of the area you want to zoom. Press the left mouse button and drag the rectangle until it covers the desired area. Release the mouse button.

9.4.1.1. Distance Between Isodose Curves

To check the distances between isodose curves, select the right-click command ***Positions/Distance***. The **Positions/Distance** dialog opens. When the dialog opens, the cursor changes into a hand symbol. Two endpoints, with a line connecting them, are displayed.

- To move one end point: Click near one of the two endpoints, move this point and release the mouse button at the desired end position.
- To move the line, connecting the two endpoints, parallel: Click near the line and move it to the desired end position, then release the mouse button.

The dialog window simultaneously displays the position of the end points, and the inline, crossline, and absolute distance between the endpoints.

An alternative way to set the positions is to enter the desired positions for both end points in the text boxes and click **Set**.

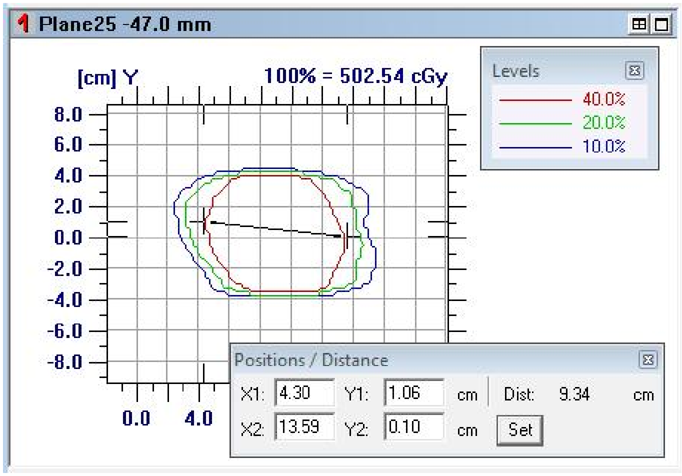

NOTICE

IMPORTANT NOTICE

FLUENCE MAPS

Use this function e.g. for fluence maps. Set the end point at the nominal field size border by entering the exact position. Position the other endpoint manually to a special dose level (e.g. 50%).

NOTICE

IMPORTANT NOTICE

ARRAY VIEW

It is also possible to check the distance between two points in the array view.

9.4.2. Profile

To analyze the profiles, follow the instructions below.

1. Click the upper left pane, the array view.
2. Option: Zoom the area of interest. See *9.4.1 Isodoses*.
3. Select the right-click command ***Profile Cursor***. The **Position/Dose** dialog opens.

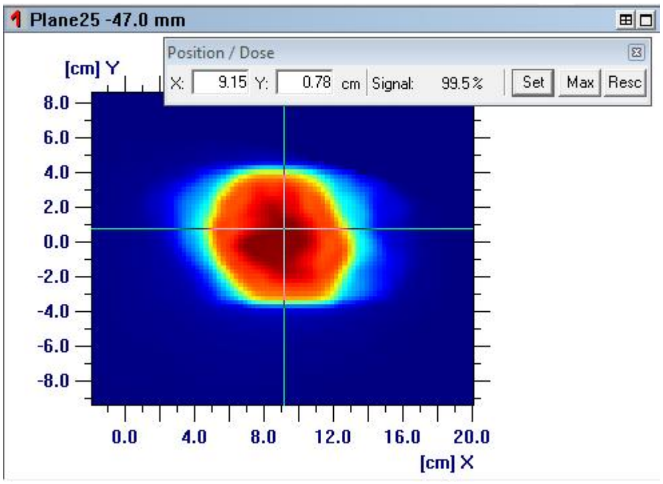

When the dialog opens, the profile cursor, in a form of a cross, is displayed. Click on the centre of the cross and move the mouse. When moving the cursor the profiles in the graph panes change according to the actual cursor position. The profiles displayed in the profile panes are those along the cross of the profile cursor. When the cursor is at the desired position, release the mouse button.

An alternative way to select profiles: Enter the desired coordinates in the text boxes and click **Set**.

4. To set the dose range for a profile, select the right-click command **Dose Range**. The **Dose Range** dialog opens.

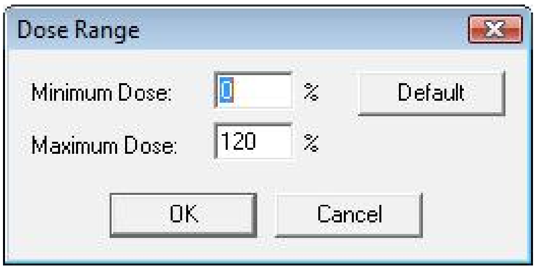

5. Enter desired values in the text boxes and click **OK**.
6. Zoom in if necessary. See *9.4.1 Isodoses.*

9.4.2.1. Flatness and Symmetry

To set the flatness criteria for the profile, select the right-click command ***Setup Markers***. The **Setup Flatness Marker** dialog opens.

Enter the dose values for the markers in the text boxes. Decide if the markers shall be displayed at absolute dose levels or relative to CAX dose. Click **OK**.

To display the flatness marker, select the right-click command ***Show markers***. Two horizontal lines are displayed at the chosen dose levels.

To display symmetry and flatness values, select the right-click command ***Profile analysis***.

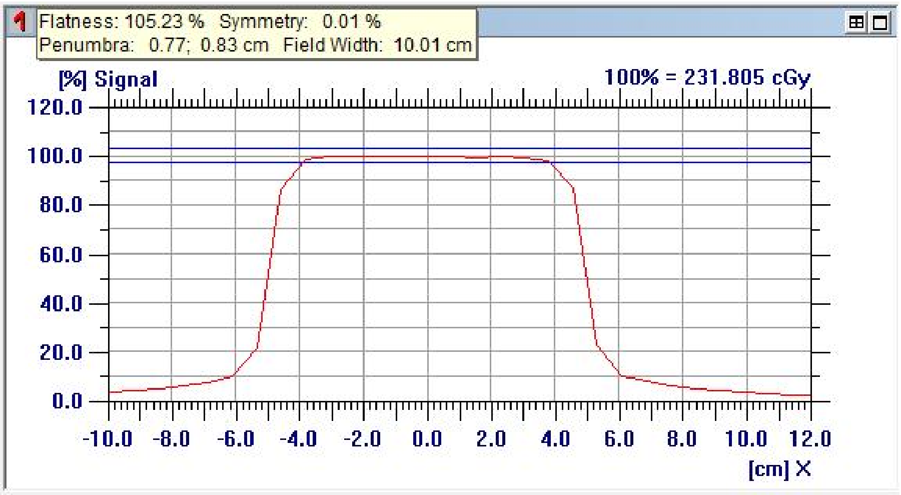

⚠ WARNING

WARNING

PARAMETERS MEASURED UNDER NON-REFERENCE CONDITIONS

Dosimetry protocols define the reference conditions for the determination of symmetry or flatness. Definition of water as medium, a certain depth, and a certain field is required, and also the type of detector to be used may be specified. If measuring under different conditions (e.g. when using I'mRT QA/BIS in air), these parameters may differ from those measured under reference conditions.

To edit or change the protocol for penumbra and field width parameterization, select ***Tools:Options*** and choose the **1D-Options** tab in the dialog.

To edit or change the protocol for flatness or symmetry parameterization, select the ***Tools:Options*** command and choose the **1D-Options** tab in the dialog.

9.4.3. Build a 3D Cube

Film data is 2D data. If this 2D data shall be compared with calculated 3D Dose distributions the measured 2D data must first be converted into 3D data.

To create a 3D cube of measured or imported data you need several images of the same measuring type with different offset values. These images then are “piled on each other” to build the 3D cube.

NOTICE

IMPORTANT NOTICE

SINGLE 2D SET

It is also possible to convert just one single 2D set (e.g. one film) into 3D dose format.

1. Select ***Workspace:Data Set 1*** or ***Data set 2***.
2. Select ***Tools:Build 3D Cube***. The **Film list** dialog opens, showing all data in the data set.
3. 2D plan verification: Select the first 2D data set and check if the offset value, i.e. the distance to the plane through the origin, is correct. If not enter the correct value and click **Set**.
4. Repeat until the offsets of all 2D data set are correct.
5. Click **Build 3D**. The result is named 3dcube and displayed.
6. Select ***Workspace:Plan Verification 3D*** to view the data in 3D View.

NOTICE

IMPORTANT NOTICE

USE 2D DATA FROM THE SAME DATA SET

You must use 2D data from the same data set to form a 3D cube. To add data to a current data set open one data set (***File:Open Data set***), select the file to be added to the current data set and select **Append to current data set**.

9.4.4. Play a Movie

After a measurement in **Movie** mode a list of the images is available in the **Field list** dialog. To create and play a movie:

1. Select ***View:Field List***. The **Field list** dialog opens.
2. Click **Play movie** to play all images after each other. The button text will change to **Stop movie**. Click this button to stop the movie. See *Field list dialog* in the on-line help for detailed instructions.

NOTICE

IMPORTANT NOTICE

MOVIE NOT PLAYED IN REAL TIME

A movie is not played in real time. The number of frames per second depends on the speed of the PC.

9.4.5. Multiple Row Analysis

For each row a 1D scan is internally extracted from the field array.

Data is normalized to 100% at the central axis, and a numerical analysis is performed. The protocol currently selected in **Options, 1D Options** is used.

Select a field.

Select ***Multiple Row Analysis*** in the ***Tools*** menu, or click the button 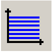 in the toolbar.

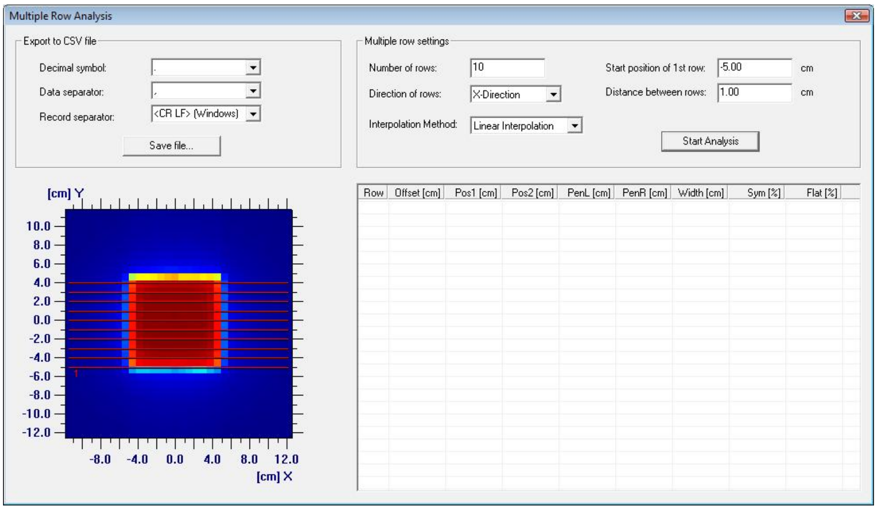

9.4.5.1. Field Display

The currently selected field is displayed in the lower left area of the dialog.

The rows are displayed as colored lines that reflect the orientation and positions.

The row number is displayed close to the line. If there is not enough space to display all row numbers, only the first row is marked.

9.4.5.2. Multiple Row Settings

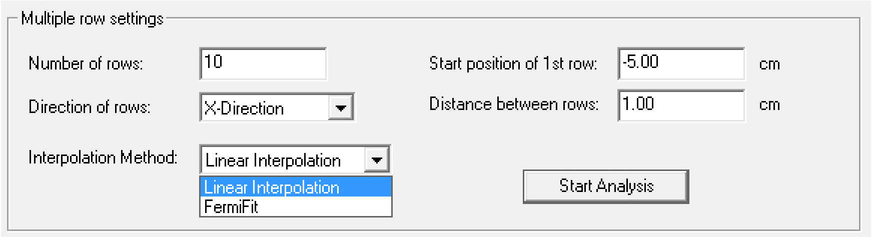

Define the settings.

NOTICE

IMPORTANT NOTICE

Z DIRECTION

It is not possible to define rows in Z direction. Z is normally depth, and is not relevant for this analysis.

The Fermi-Fit algorithm is an option in **Interpolation Method**. It is used for determination of field width (e.g. 50%) and penumbra width (e.g. 20% - 80%). It improves the determination of the 50% point in the penumbra in open rectangular fields, or MLC delimited segments parallel to the leaf direction. See also *14.1.1.3 FermiFit Interpolation*.

NOTICE

IMPORTANT NOTICE

INTENSITY MODULATED FIELDS

The FermiFit algorithm is not intended to be used on intensity modulated fields.

9.4.5.3. Result Fields

Each row is displayed with a caption (row number) as the first record.

The following parameters will be displayed:

- Offset
- Pos 1
- Pos 2
- Pen L (left penumbra)
- Pen R (right penumbra)
- Symmetry
- Flatness

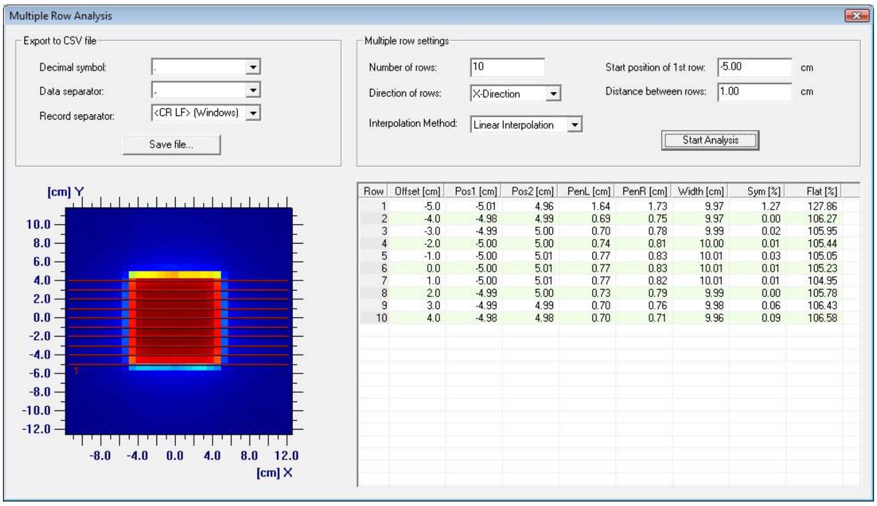

Multiple row analysis, linear interpolation

9.4.5.4. Export to CSV File

Select separator for decimal, data, and record.

Click **Save File**, and select the name and location of the file. The default path is the workspace directory of the currently displayed field.

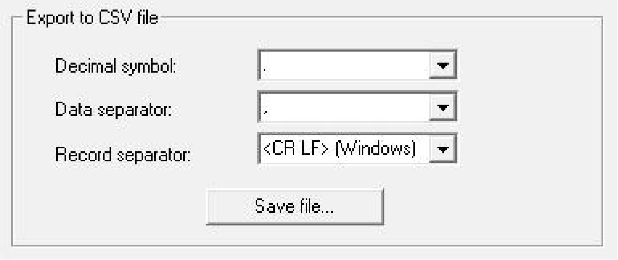

9.4.6. Time Based Analysis

Data from a number of files in the field list can be displayed as a list of values at one position or region. The values are calculated as the average value within the ROI in each field.

Select ***Field List*** in the ***View*** menu to open the **Field List** dialog. Select files.

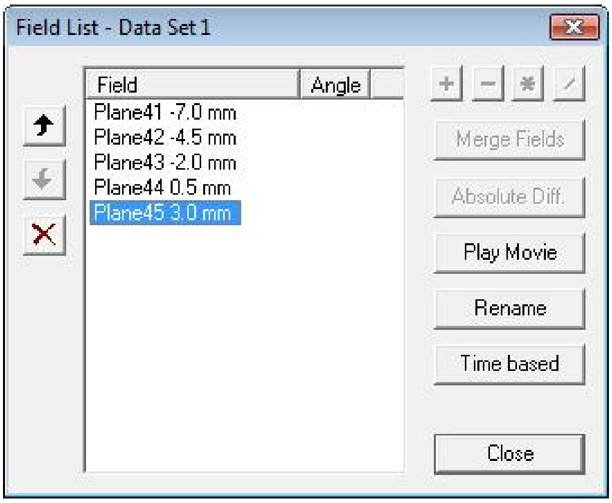

As an option, the buttons **+**, **-**, **·**, /, or **Absolute Diff.** can be selected. Select two or several files, and click the appropriate button.

Click **Time based** to perform the calculations.

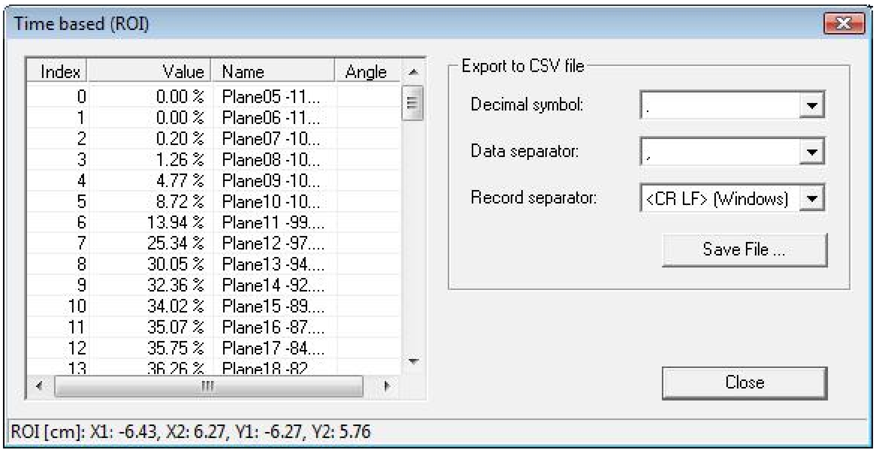

Data can be exported to a CSV file. See *9.4.5.4 Export to CSV File*.

9.4.7. Merge Fields

Two or more fields can be merged together to one large field.

In the following example two 13 × 13 cm measured fields will be merged with a resulting overlapping region of 1,5 cm.

In the first measurement the MatriXX was shifted 5 cm in – x direction.

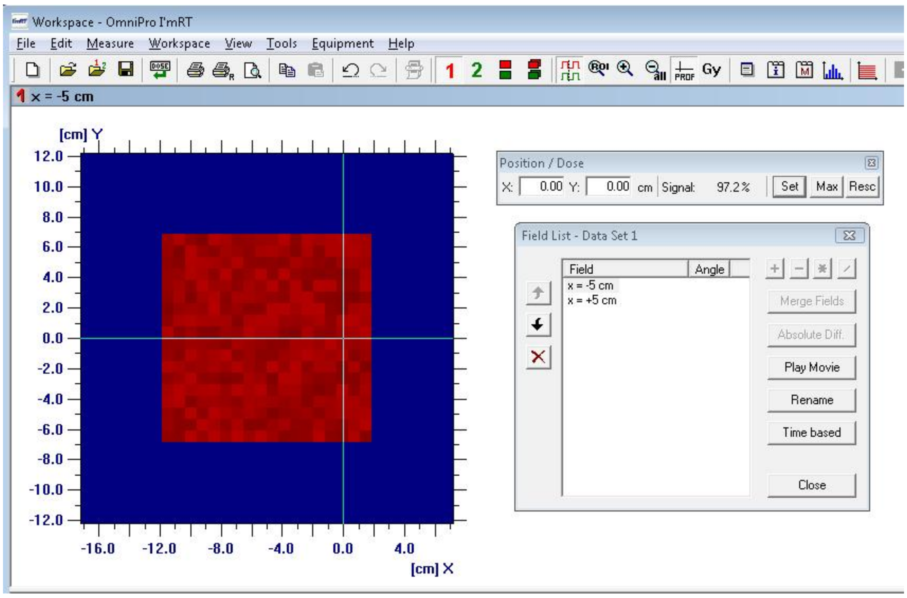

In the second measurement the MatriXX was shifted 5 cm in + x direction.

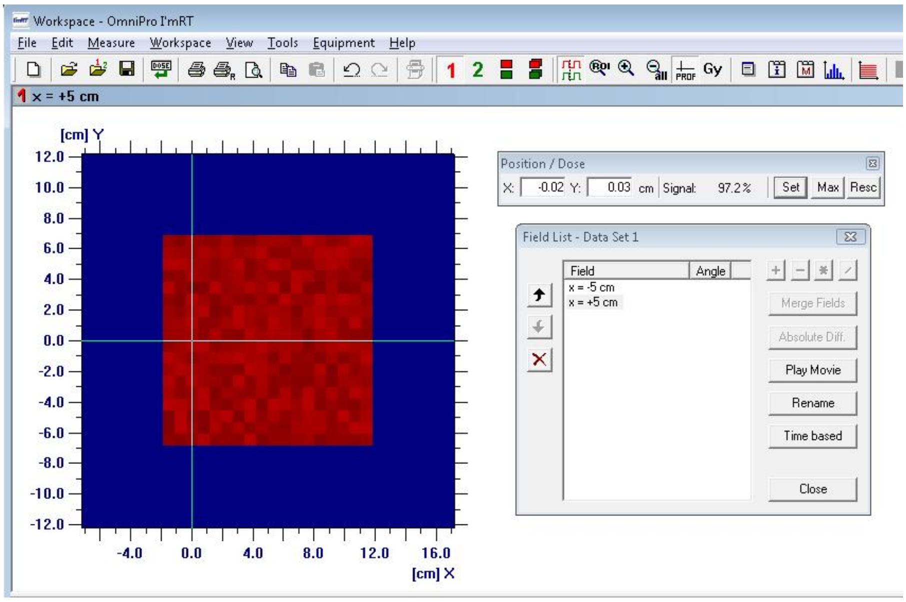

For merging these both measurements select the two fields in the field list, and click the button **Merge Fields**. The two images will be added in respect to their coordinates, and a large image will be created.

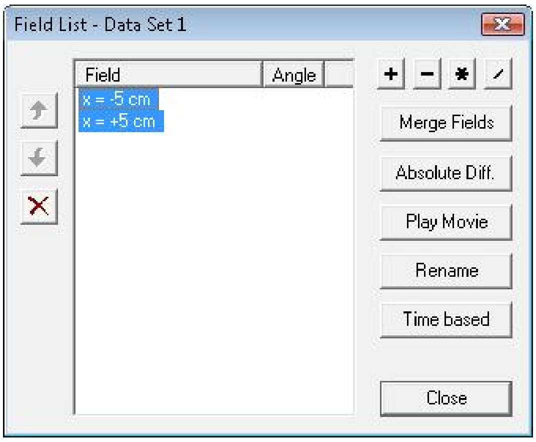

The large field will have a size of 23 × 13 cm.

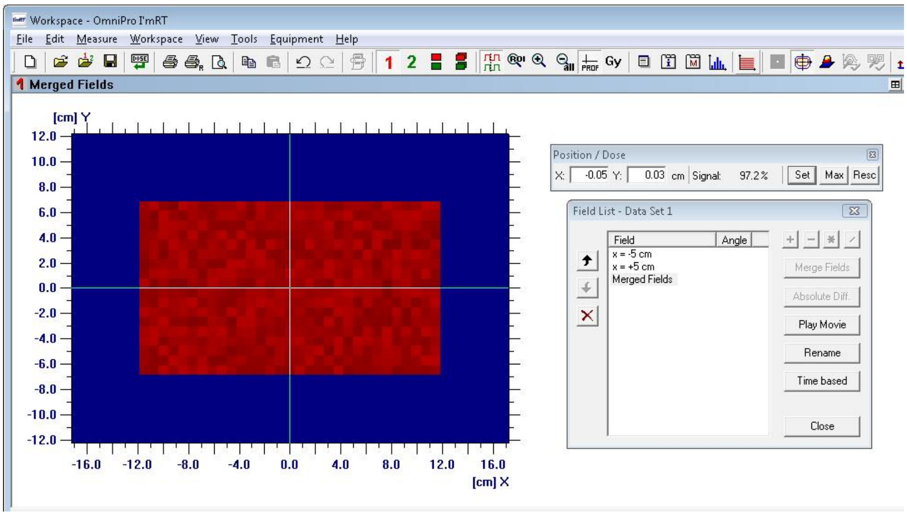

In the overlapping region the average of the pixels will be calculated and used for creating the large image.

NOTICE

IMPORTANT NOTICE

OVERLAP OF TWO FIELDS

The overlapping size of two fields should be at least 1.5 cm so two chamber rows will be covered.

With four measurements all 22 × 22 cm and an overlapping region of 2 cm it is possible to achieve a large field size of 40 × 40 cm.

9.5. Plan Verification 2D

NOTICE

IMPORTANT NOTICE

OPEN TWO DATA SET FILES

To perform the operations described below it is necessary to open two data set files. Normally this will be the measured data in one data set, and imported dose planes from TPS in the other data set.

NOTICE

IMPORTANT NOTICE

2D DATA SET AND 3D DOSE CUBE

It is not possible to compare a 2D data set with a 3D dose cube. The 2D data must first be converted to a 3D cube. See *9.4.3 Build a 3D Cube*.

Click the toolbar button 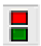 to switch to the 2D Plan verification view. The data set 1 file is displayed in the upper left pane and the data set 2 file is displayed the lower left pane. You can display both files in either isodoses view, 2D array view or 3D view. The upper right pane, the **Compare Profiles** pane, displays profiles or isodoses of both data set files. The lower right pane, the **Result pane**, will display the result of mathematical comparisons between the two data sets.

If you want to synchronize the views, click 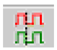 . The profile cursors will be synchronized.

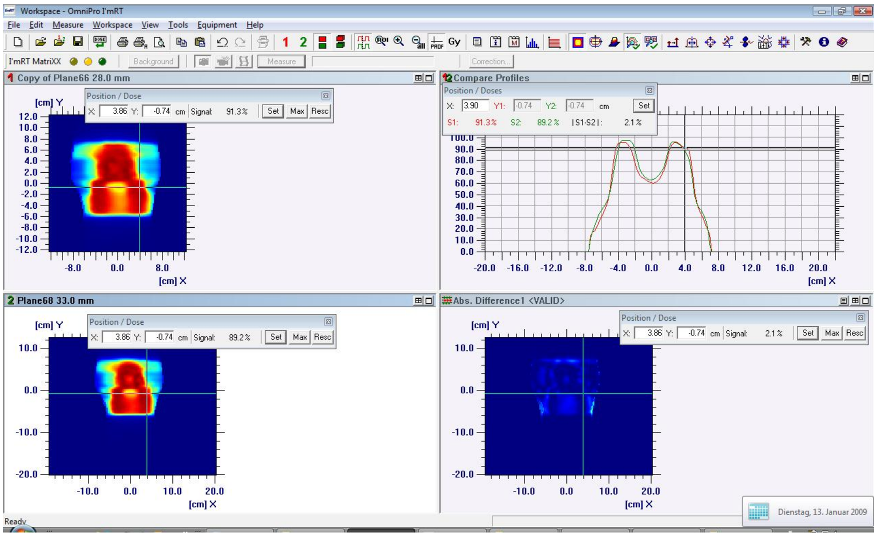

9.5.1. Compare Profiles

All instructions given in *9.4.2 Profile* can be followed in 2D Plan verification. The difference is that you see only one plane at a time. To switch planes, select the right-click command **X**, **Y**, or **Z**.

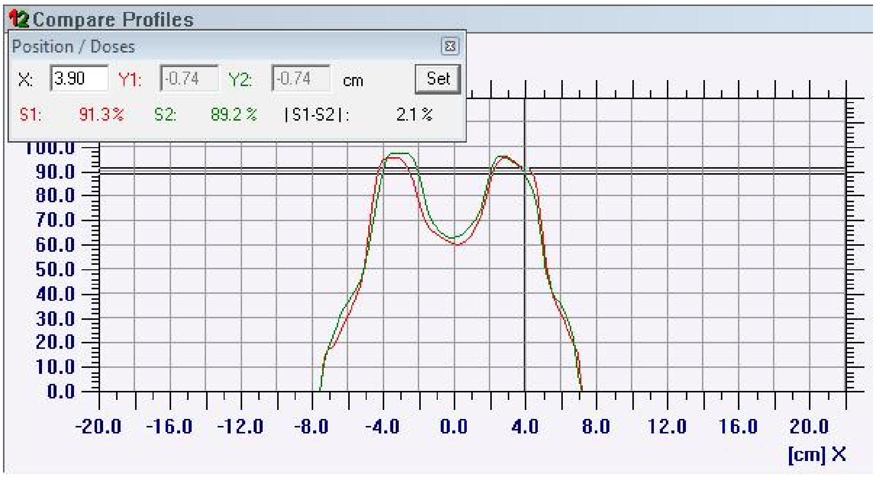

9.5.2. Compare Isodoses

The instructions given in *9.4.1 Isodoses* can be followed in 2D Plan verification. The difference is that the isodose levels for the Data set files can be set separately. Right-click and choose ***Isodose Levels Set 1*** or ***Isodose Levels Set 2***.

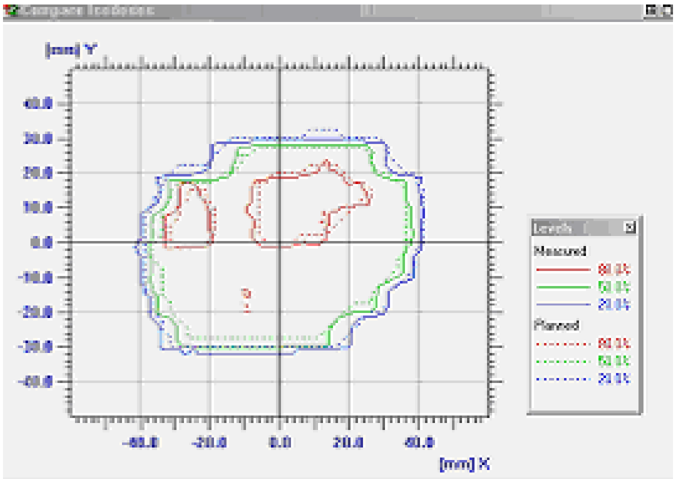

9.5.3. Synchronized Rescaling

Synchronized rescaling can be performed on data sets with absolute or relative dose values in both sets, and with identical measuring planes.

Select ***Synchronized Rescaling*** in the ***Edit*** menu.

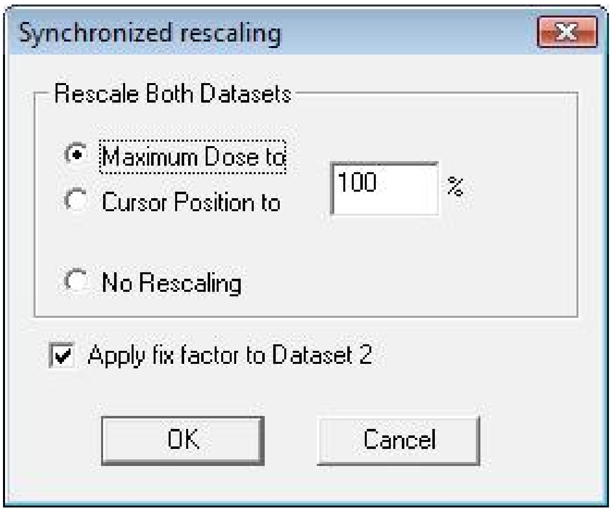

Select **Maximum Dose to** or **Cursor Position to**, and enter a value (default 100%).

NOTICE

IMPORTANT NOTICE

ENABLE CURSOR POSITION TO

To enable **Cursor Position to**, the cursor must be activated. The data sets must be synchronized. Click the synchronize button 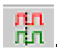.

Option for absolute data in both datasets: Mark **No Rescaling** and **Apply fix factor** to **Dataset 2**, to read the factor from dose to percentage (e.g. 100% = 100 cGy) from data set 1, and apply it to data set 2, by keeping the absolute value and changing the relative value.

9.5.4. Histogram

The Histogram function is available for 2D data.

NOTICE

IMPORTANT NOTICE

3D DATA

For 3D data the histogram displayed applies to the entire cube, regardless of which dimension or level that is displayed in the data set.

The data value (e.g. the dose) is displayed on the x axis, and the number of pixels in this range is displayed on the y axis.

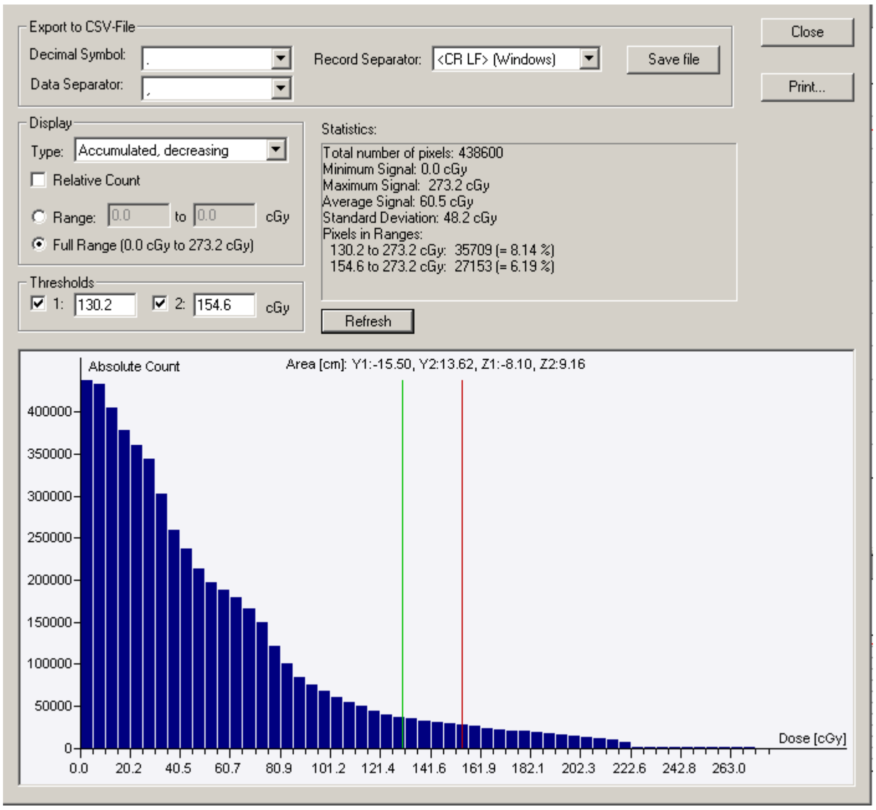

Type: Select the type of histogram to be displayed.

Relative Count: Check the checkbox to display the number of pixels as relative count (%).

Uncheck the checkbox to display the number of pixels as absolute count.

Range: Select **Range**, and define values, to display other than the full range of data.

Full Range: Select **Full Range** to display the full range of data.

Threshold: Check the checkbox and define threshold data. Vertical lines will appear on the selected threshold values in the graph.

Export to CSV: Define **Decimal Symbol**, **Data Separator**, and **Record Separator**. Click **Save file** to save data as a CSV file.

The pixel values for the selected ranges are displayed in the **Statistics** pane:

Total number of pixels: 438600
Minimum Signal: 0.0 cGy
Maximum Signal: 273.2 cGy
Average Signal: 60.5 cGy
Standard Deviation: 48.2 cGy
Pixels in Ranges:
0.0 to 130.2 cGy: 402891 (= 91.86 %)
130.2 to 154.6 cGy: 8556 (= 1.95 %)
154.6 to 273.2 cGy: 27153 (= 6.19 %)

Standard Histogram

Total number of pixels: 438600
Minimum Signal: 0.0 cGy
Maximum Signal: 273.2 cGy
Average Signal: 60.5 cGy
Standard Deviation: 48.2 cGy
Pixels in Ranges:
0.0 to 130.2 cGy: 402891 (= 91.86 %)
0.0 to 154.6 cGy: 411447 (= 93.81 %)

Accumulated increasing

Total number of pixels: 438600
Minimum Signal: 0.0 cGy
Maximum Signal: 273.2 cGy
Average Signal: 60.5 cGy
Standard Deviation: 48.2 cGy
Pixels in Ranges:
130.2 to 273.2 cGy: 35709 (= 8.14 %)
154.6 to 273.2 cGy: 27153 (= 6.19 %)

Accumulated decreasing

Refresh: Click **Refresh** to change the histogram display after changing the range or threshold data.

9.5.5. Palette

9.5.5.1. Change Palette

Right-click in the **Result** pane and select **Change Palette** in the context menu.

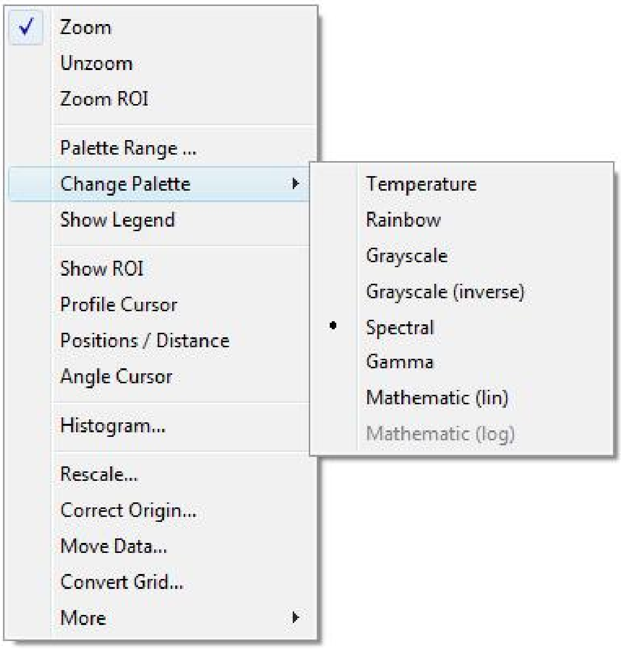

Select a palette, and define the range in **Palette Range**.

9.5.5.2. Palette Range

Right-click in the **Result Set** pane, and select ***Palette Range*** in the context menu.

If one of the options ***Temperature***, ***Rainbow***, ***Grayscale***, ***Inversed Grayscale***, and ***Spectral*** is selected, the minimum and maximum dose shall be defined.

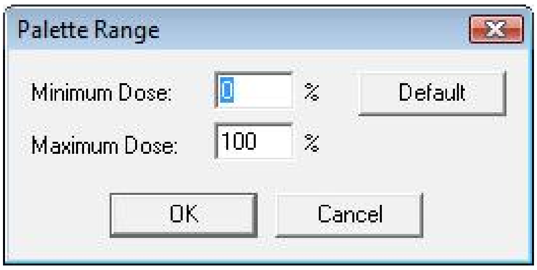

If Gamma is selected (mandatory for gamma calculations), the minimum dose and the upper threshold shall be defined. The palette range should be 0% to 125%. The color turns to red at the selected upper threshold value. 100% is equal to the gamma value 1.

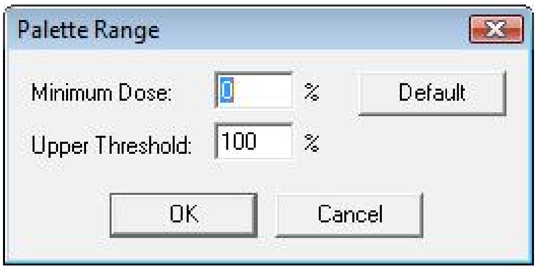

If ***Mathematics (lin)*** is selected, the lower and upper threshold shall be selected.

9.5.6. Calculations

You can perform a number of calculations on the data set files and view the results in the lower right pane.

To select type of calculation, click in the **Result pane**, or select ***Calculate Result*** in the ***Tools*** menu. The calculations menu is displayed.

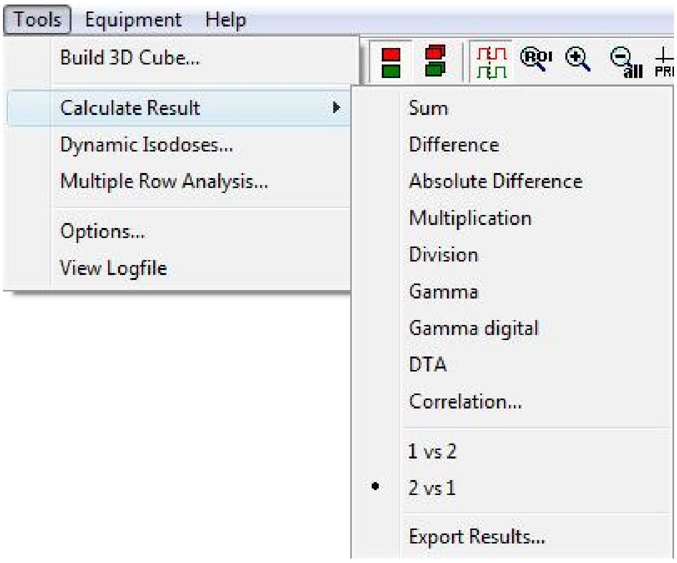

Select type of calculation. A detailed description of the algorithms used is found in *Algorithms / 2D Data*.

Select the order of calculation (dataset 1 vs. dataset 2, or vice versa).

NOTICE

IMPORTANT NOTICE

DISSIMILAR RESOLUTIONS

When the two datasets have dissimilar resolutions, the results may also be slightly dissimilar.

Use the ***Export Results*** command to export the **Result data** as an opd file (OmniPro I'mRT data).

NOTICE

IMPORTANT NOTICE

COMPARING HIGH AND LOW RESOLUTION DATA

When comparing two matrices with different resolution, the high resolution data (this is typically the scanned film data) should be in data set 2, the lower resolution data (typically the imported data from treatment planning system) in data set 1. This speeds up the calculation and prevents interference-like patterns in the gamma matrix.

NOTICE

IMPORTANT NOTICE

GAMMA CALCLULATION

To evaluate the result of a gamma calculation, the palette should be switched to ***Gamma***. (Use the right-click command ***Change Palette***). The palette range should be 0% to 125% (set the range in the **Palette Range** dialog). The color turns to red at the selected upper threshold value. 100% is equal to the gamma value 1.

The delta dose and delta distance values are displayed in the caption of the result view.

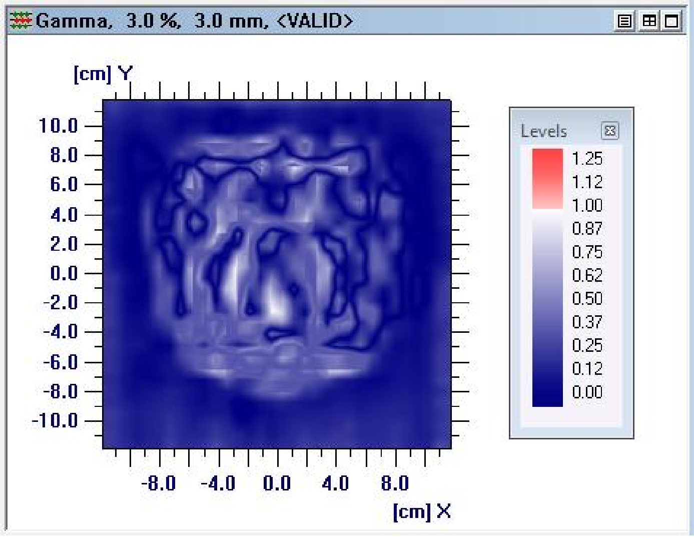

9.5.6.1. Practical usage of the Gamma Method

The most common method to verify calculations vs. measurements is the Gamma method. Therefore a workflow for using the Gamma method in a correct way is described:

1.) Import the plan, perform the measurements.

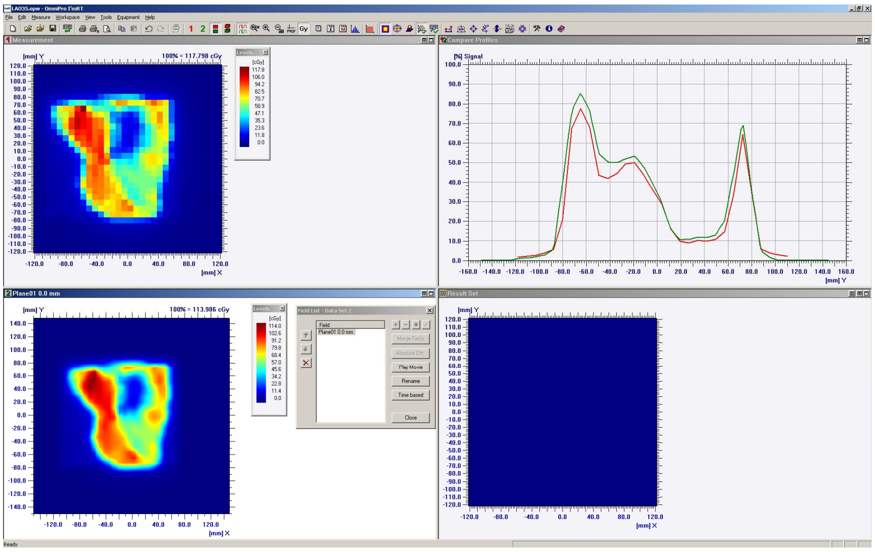

The screenshot shows the measured field in data set 1 and the imported dose plane in data set 2.

2.) Convert the grid of the imported TPS dose plane to the resolution of the MatriXX. With this step it is simulated what the MatriXX should “see”. This step is required as the MatriXX has a resolution of 7.62 mm while TPS calculates with a resolution of 1 mm (in this case).

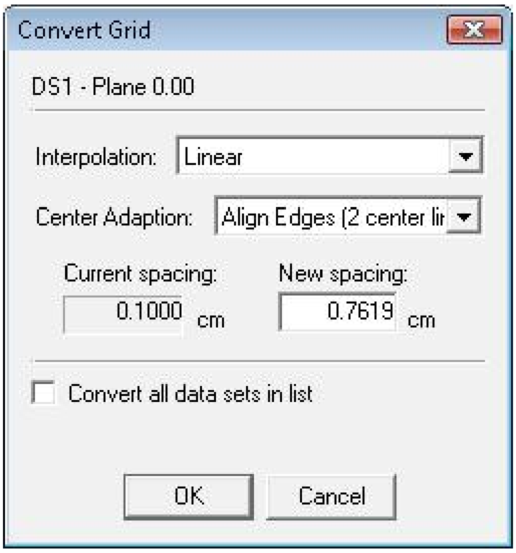

If the used linac has an even number of leaf pairs, the use of the centre adaption Align edges (2 centre lines) is recommended. For an odd number the **Centre Adaption** “Align centre (one centre line)” is recommended. Centre Adaption is required especially when the grid resolution is reduced. Please check the effect on a square field (plan import). A shift of the field can be seen for grid conversion to lower resolution with no centre alignment.

3.) Convert the grid of the TPS data and the measurements to a higher resolution (1mm for example).

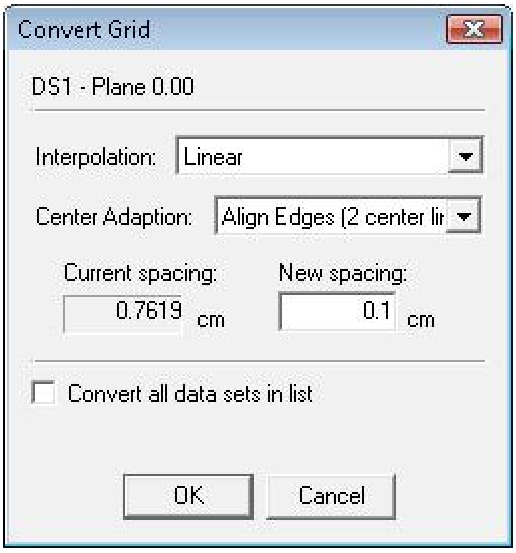

The reason for the convert grid to a higher resolution is the discrete Gamma calculation in OP I'mRT (the number of measurement points are evaluated).

4.) Rescaling

Use synchronize rescaling in data set 1 (**Edit:Synchronize Rescaling**).

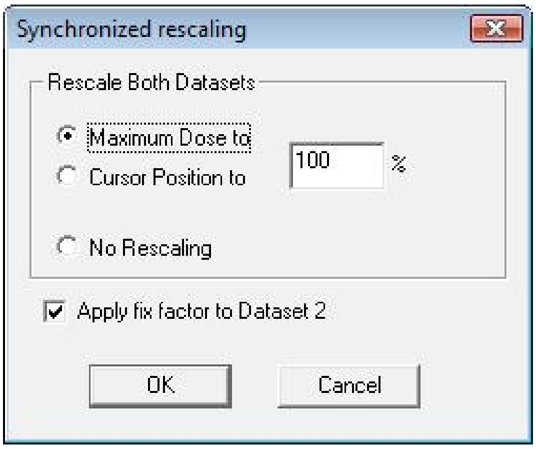

Please use the settings like in the screenshot above.

The reason for this normalization step is the fact that OmniPro I'mRT is using the normalization value to calculate the ΔGamma value. The ΔGamma, is entered as percentage value under **Tools:Options:2D options**. The Gamma value is therefore calculated in relative values in OP I'mRT.

Example:

ΔGamma = 3%, Normalized dose 100% = 1 Gy

→ Delta Gamma = 3 cGy

ΔGamma = 3%, Normalized dose 100% = 0,5 Gy

→ Delta Gamma = 1,5 cGy

OP I'mRT is using the global Gamma.

Difference between global and local Gamma:

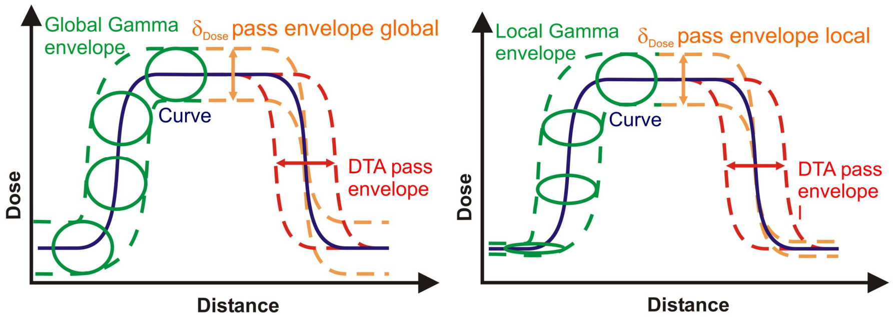

5.) Calculate Gamma

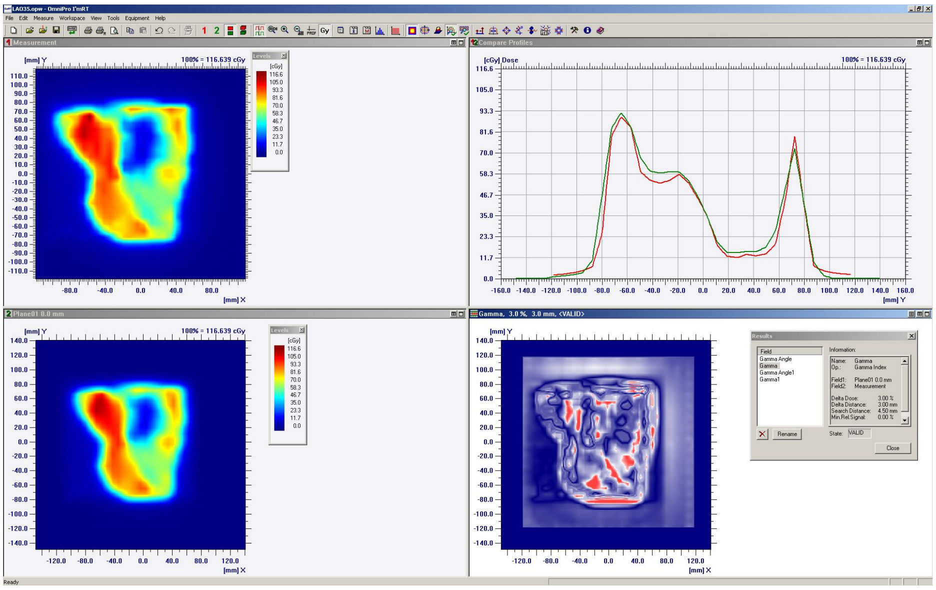

Compare 1 vs. 2 and 2 vs. 1 (Measurement vs. TPS and TPS vs. Measurement). Therefore select the referring command in ***Tools:Calculate Result***.

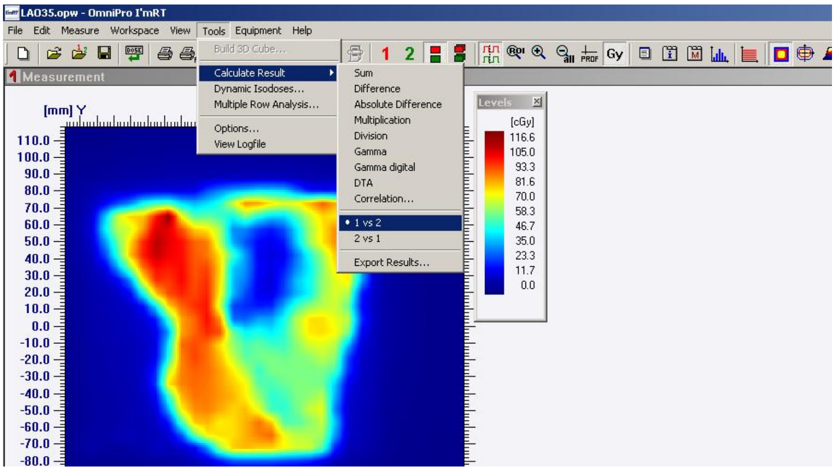

5.) Analyzing

For analyzing the profile cursor as well as the histogram is available. Please refer to chapter *9.4.2 Profile* and *9.5.4 Histogram.*

9.6. Plan Verification 3D

NOTICE

IMPORTANT NOTICE

OPEN TWO DATA SET FILES

To perform the operations described below, it is necessary to open two data set files. Normally this will be the measured data in one data set, and an imported dose cube from TPS in the other data set.

NOTICE

IMPORTANT NOTICE

2D DATA SET AND 3D CUBE

It is not possible to compare a 2D data set with a 3D dose cube. The 2D data must first be converted to a 3D cube. See *9.4.3 Build a 3D cub*e.

Click the toolbar button 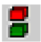 to switch to 3D Plan verification view.

9.6.1. Difference to 2D

In Plan 3D Verification you can perform the same operations as in 2D. The difference is that you can choose which plane to view, and that it is possible to move through the different planes, step by step.

To select a plane with another orientation, click **XY**, **XZ**, or **YZ**.

To select a plane with another offset, drag the slider.

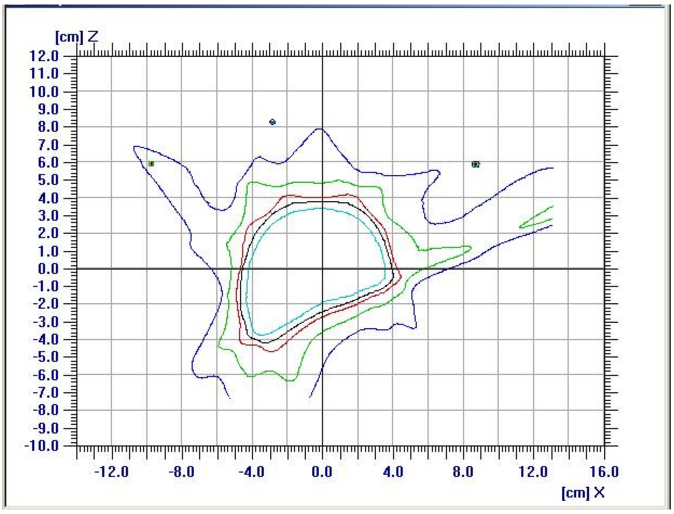

If you want to synchronize the views, click 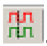. The profile cursors, the plane orientation, and the offset of the selected plane will be synchronized.

10. Data Output

10.1. Print Data

10.1.1. Preview and Print a Selected Pane

1. Select the pane you want to print.
2. To view the page setup, select the menu command ***File:Print preview***. The page setup is displayed in a preview window.

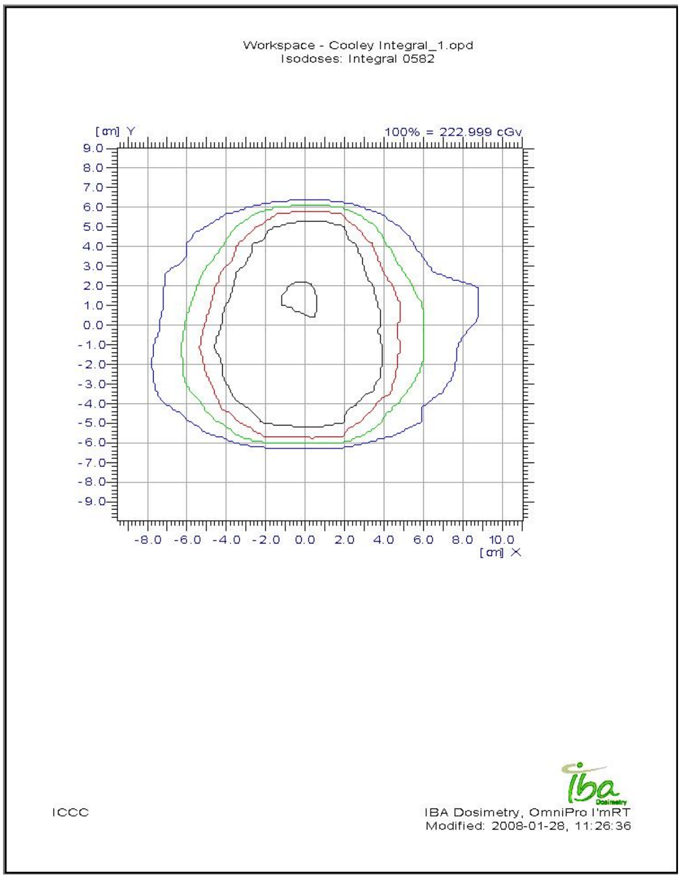

3. Click **Print** in the preview window. A standard **Print** dialog opens.

10.1.1.1. Print Directly

To print directly, select the menu command ***File:Print***, or click the **Print** toolbar button. A standard **Print** dialog opens.

10.1.2. Preview and Print All Panes in the Window

10.1.2.1. Print Report Preview

To view the page setup, select the menu command ***File:Print Report Preview***. A preview of the report is displayed.

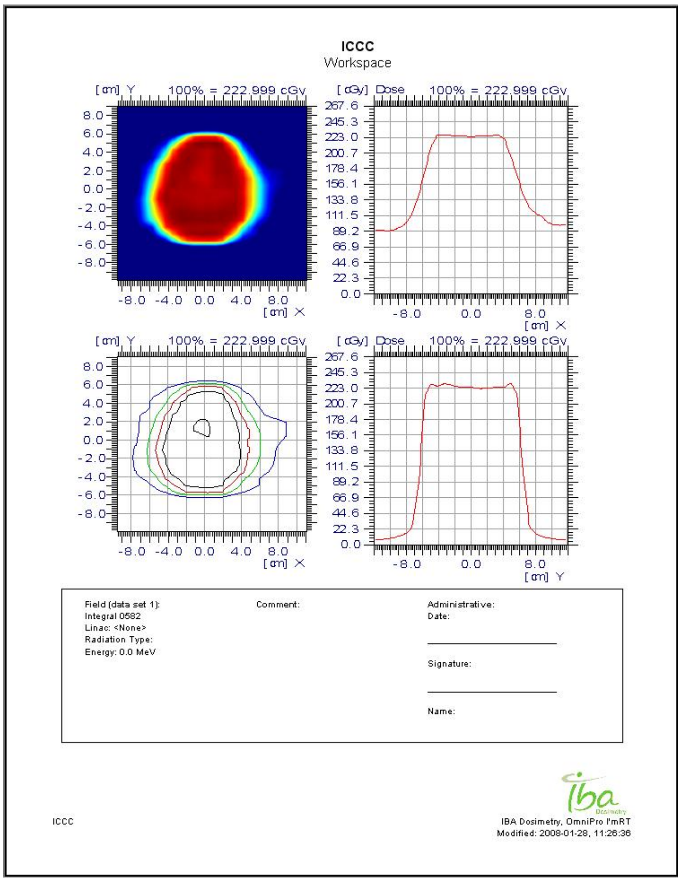

10.1.2.2. Report Setup

To define the report layout, select ***File:Report Setup***. The **Report Setup** dialog opens.

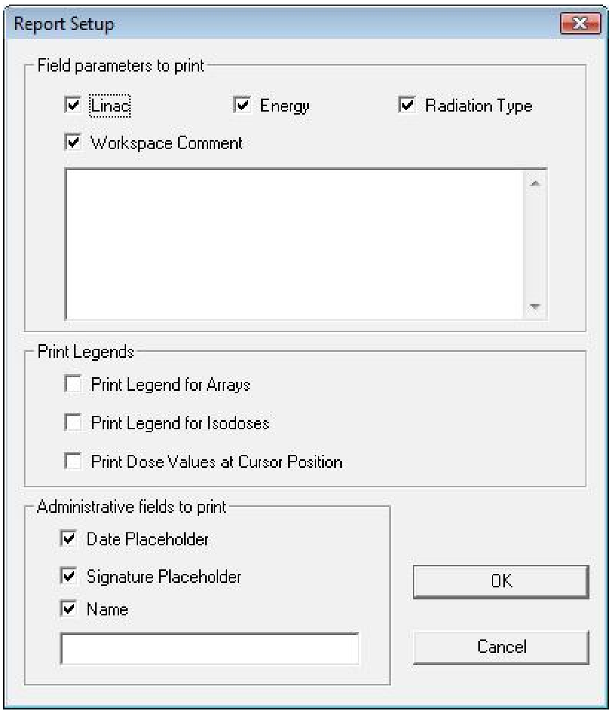

1. Mark which field parameters that shall be printed.
2. Mark **Workspace Comment** if a comment shall be printed. It can be used to store additional information about the complete workspace - not only one single data set.
3. Mark **Administrative Fields** to print lines for date, signature, and name. The name will be printed, if entered in the field below the **Name** checkbox.
4. Click **OK**, and select ***Print Report Preview*** to view the setup, or print directly.

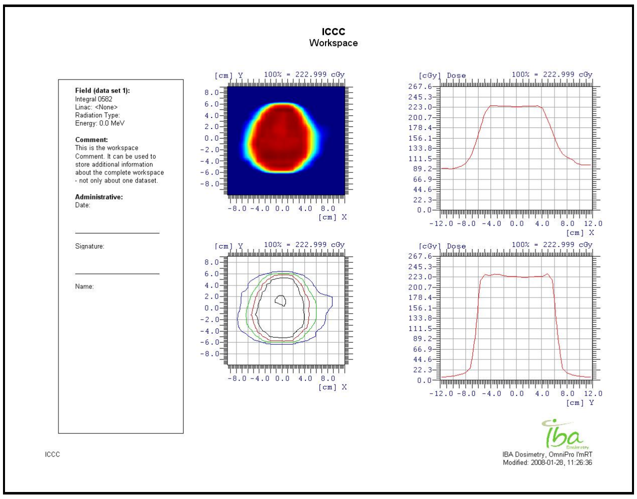

10.1.2.3. Print Directly

To print without previewing, select the menu command ***File:Print Report***, or click the button 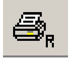 in the toolbar.

A standard **Print** dialog opens.

10.1.3. Print Setup

OmniPro I'mRT will use the default printer unless you select an alternative printer in the **Print Setup** dialog, which is opened by choosing the ***File:Print Setup***.

10.2. Copy Data

2D data can be copied and pasted between datasets within the OmniPro I'mRT application, or from OmniPro I'mRT to an external program in ASCII format (e.g. to Excel and Notepad).

To re-import copied data from an external file to OmniPro I'mRT, use generic ASCII import (see *8.4 Generic ASCII Import*).

Settings for the copy/paste function are made in **Options**. Select ***Tools:Options*** and click the **Copy/Paste** tab. (For further description, see the OmniPro I'mRT online help).

Select ***Copy and Paste*** in the ***Edit*** menu, or use the toolbar buttons **Copy** 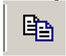 and **Paste** 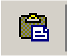, to copy and paste data.

10.3. Export Data

10.3.1. Export to ASCII

You can save your data file in ASCII based text format. The file is saved with the extension *.opg.

*.opg files can be exported to external software applications (e.g. MS Excel and RIT).

⚠ WARNING

WARNING

LIMITED NUMBER OF DATA ROWS AND/OR COLUMNS

Some Windows applications support fewer rows and/or columns than the number of columns exported by OmniPro I'mRT (e.g. Microsoft EXCEL supports 256 × 65536).

If importing OmniPro I'mRT ASCII data to those applications, rows and/or columns exceeding the limit will not be imported.

If necessary, reduce the number of data by selecting a small region of interest (ROI).

Select the menu command ***File:Export Data: Generic ASCII File…***. The **ASCII Output** dialog opens.

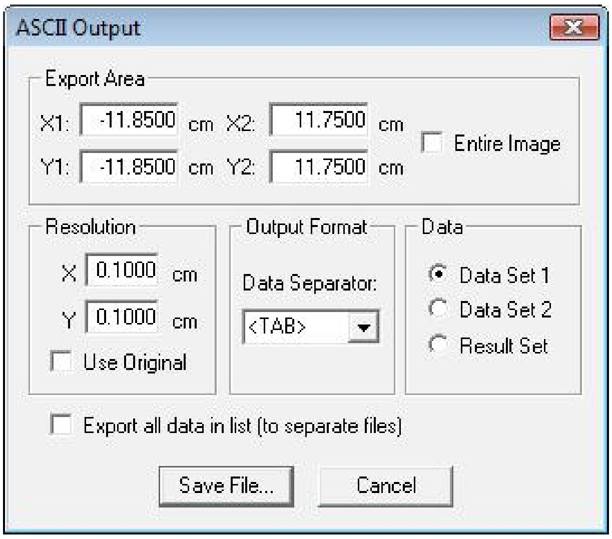

10.3.1.1. Manually Defined Export Data

1. Select which kind of data to export: **Data Set 1**, **Data Set 2**, or the **Result Set**.
2. If the **Entire Image** and/or **Use Original** checkboxes are unmarked, the export area and the resolution values can be entered manually.
3. Enter values in the **Export Area** and **Resolution** fields.
4. Click **Save File**. A standard **Save** dialog opens.
5. Define the file name and location.
6. Click **Save**.

The values defined for the export area and resolution are stored, and will be displayed the next time the dialog is opened.

10.3.1.2. Original Export Data

If the **Entire Image** and/or **Use Original** checkboxes are marked, the original values of the field are used, and cannot be changed.

1. Click Save File. A standard **Save** dialog opens.
2. Define the file name and the location.
3. Click **Save**.

The next time that the dialog is opened, the checkboxes will remain marked, and the values for the current image are displayed:

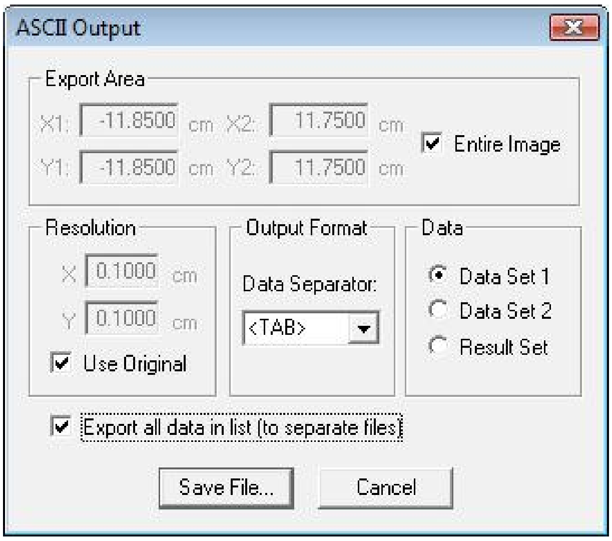

10.3.1.3. Export All Data in List (to Separate Files)

If the **Export all data in list (to separate files)** checkbox is marked, all images in the active dataset will be exported to separate files.

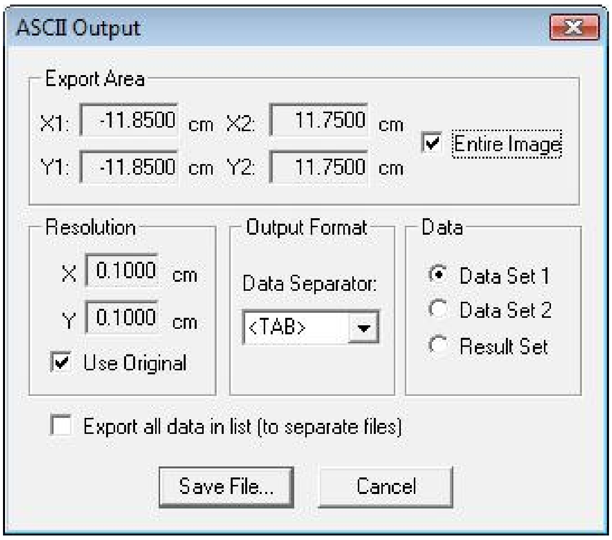

Entire Image and/or Use Original unmarked:

Manually defined data can be applied. Enter values in the **Export Area** and **Resolution** fields. Entered data will be applied on all images in the dataset.

NOTICE

IMPORTANT NOTICE

IMAGE ORIENTATIONS

If the images have different orientations (XY, XZ, YZ), the **Entire Image** must be activated.

Entire Image and/or Use Original marked:

The original, individual data for each image will be applied. The data displayed (dimmed) in the **Export Area** and **Resolution** fields refer to the current image, and are not valid for other images in the list.

1. Click **Save File**. A standard **Save** dialog opens.
2. Define the location and a file name for the list of images.
3. Click **Save**. The exported files will be named Filename_001.opg, Filename_002.opg, and so on.

10.3.2. Export to BRI

You can save your data file in BRI Image format (Beam Image System Image file). The file is saved as a *.bri file.

1. Select the menu command ***File:Export Data:BRI file***. The **Export to BRI Format** dialog opens.

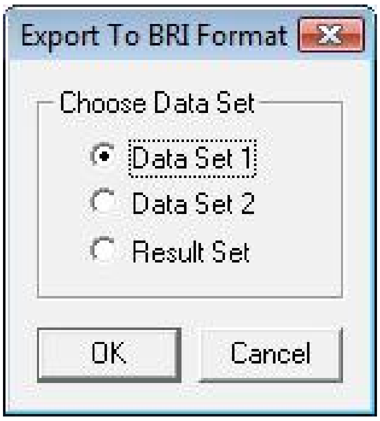

2. Select which kind of data to export: **Data Set 1**, **Data Set 2**, or the **Result Set**.
3. Click **OK**. A standard **Save** dialog opens.
4. Define the file name and the desired location.
5. Click **Save**.

10.3.3. Export to RT Image

Data can be exported to RT Image. Select ***File:Export Data:Export RT Image***.

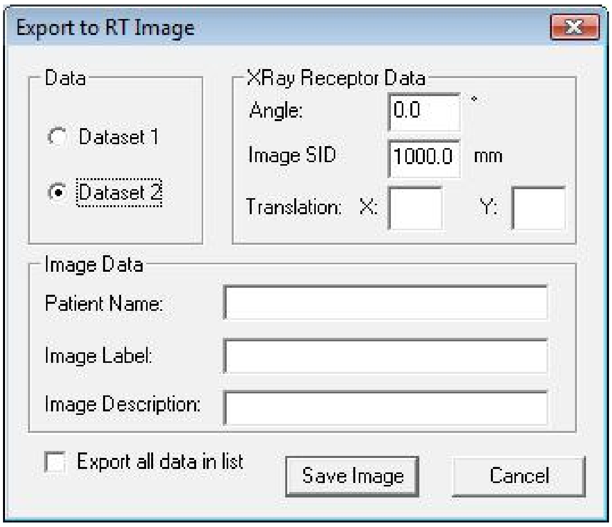

Data section:

Select dataset.

XRay Receptor data section:

Angle: The position of I'mRT MatriXX, as defined in MatriXX Setup in Measurement Parameters (see *7.4.5.2 Define the Measurement Parameters*).

Image SID: Default values are displayed:

- For a MatriXX mounted on a table: 100 cm
- For a MatriXX mounted on the gantry: 80 cm

The values are editable.

Translation: The position in (x, y, z) coordinates of origin of IEC X-RAY IMAGE RECEPTOR system in the IEC GANTRY coordinate system.

Image Data section:

Enter data.

Export all data in list: Mark the checkbox to export all data in the **Field List**.

10.3.4. Export to TomoTherapy

Data can be exported to the TomoTherapy treatment planning system (TomoTherapy DQA Analysis panel of the HiArt Planning Station software application, version 2.2.2, or later).

- The image is interpolated to 0.5 mm resolution.
- Fix markers are added to the MatriXX image at the center of the corner pixels (-110.5, -110.5) and (110.5, 110.5).

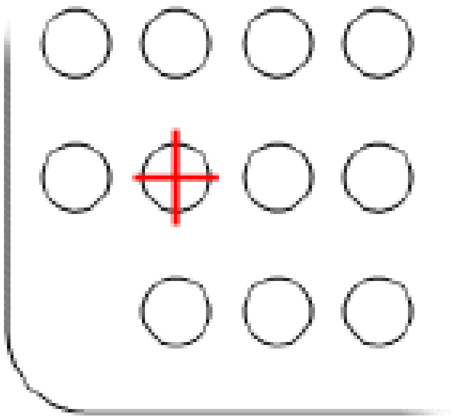

- Data and markers are exported as 16-bit .tif file.
- A calibration file (scanner value to cGy) is created.

10.3.4.1. Export Procedure:

1. Load the MatriXX image in Dataset 1 or 2.

NOTICE

IMPORTANT NOTICE

ABSOLUTE VALUES

The image must have absolute values.

2. Select the menu command ***File:Tomo Therapy:Export Image File***. The **Image Output** dialog will open.

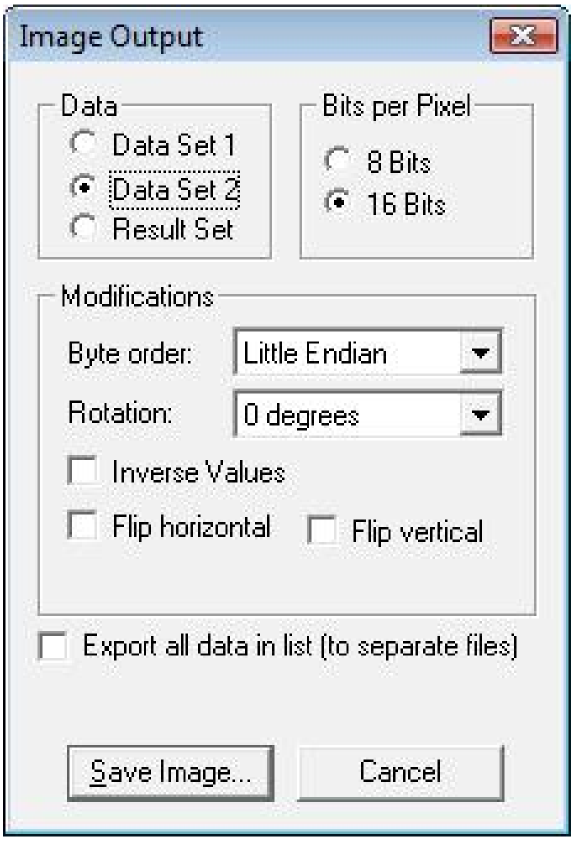

3. Select which dataset to save.
4. Rotate the image, if necessary.
5. Flip the image, if necessary.

NOTICE

IMPORTANT NOTICE

NO NEGATIVE VALUES

The image must not contain negative values. If it does, select **Clip negative values to zero**.

6. Click **Save Image**.

The image is saved as <filename>.tif, and the calibration file as <filename>.cal.

10.3.5. Export to OmniPro Accept

Data can be exported to the OmniPro Accept software. The file is saved in RFA 300 Beam Data *.asc format.

NOTICE

IMPORTANT NOTICE

SELECT ACCELERATOR WITH VALID SSD

Before measuring or importing data that shall be exported to OmniPro Accept for profile analysis, an accelerator with a valid SSD must be selected.

1. Select the menu command ***File:Export Data:OmniPro Accept***. The **OmniPro Accept ASCII Output** dialog opens.

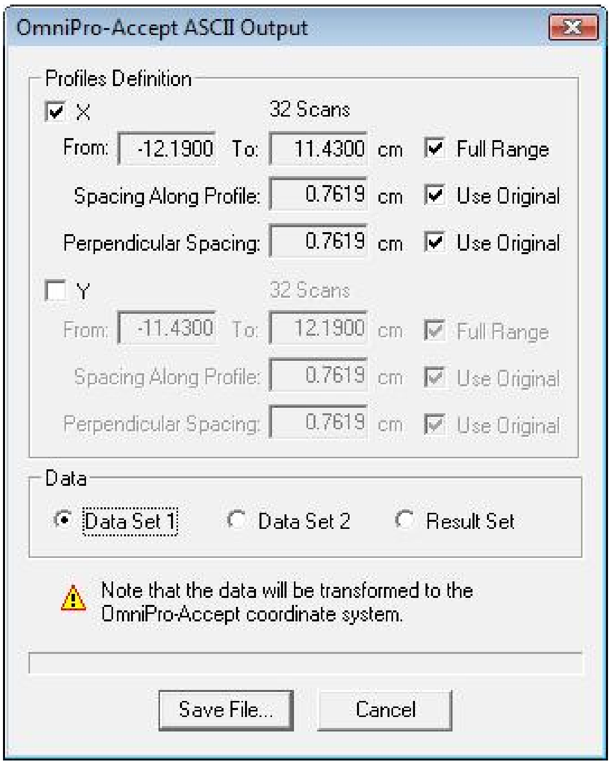

2. Select the profile direction (X and/or Y), and which data that shall be exported (**Data Set 1**, **Data Set 2**, or **Result Set**).
3. The ranges can be defined by the user: Un-mark the checkboxes **Full Range** and **Use Original** to enable the input fields.
4. Click **Save File**. A standard **Save** dialog opens.
5. Define the file name and the desired location.
6. Click **Save**.

10.3.6. Export of Data in Graphic Format

It is possible to export data from the main application in a graphic format. Supported file types are .tif and lossless .jpg (16 bit grey scale images). Lossless .jpg has a better compression, but is not recognized by most commercial imaging applications. (The Medical Imaging application supports lossless .jpg).

Saving in a graphic format does not preserve calibration and origin definition information. After saving, this information will be lost.

There is also an option to save in raw format, i.e. only the 16 bit buffer will be saved, without any header information.

1. Select ***File:Export Data:Image File***. The **Image Output** dialog opens.

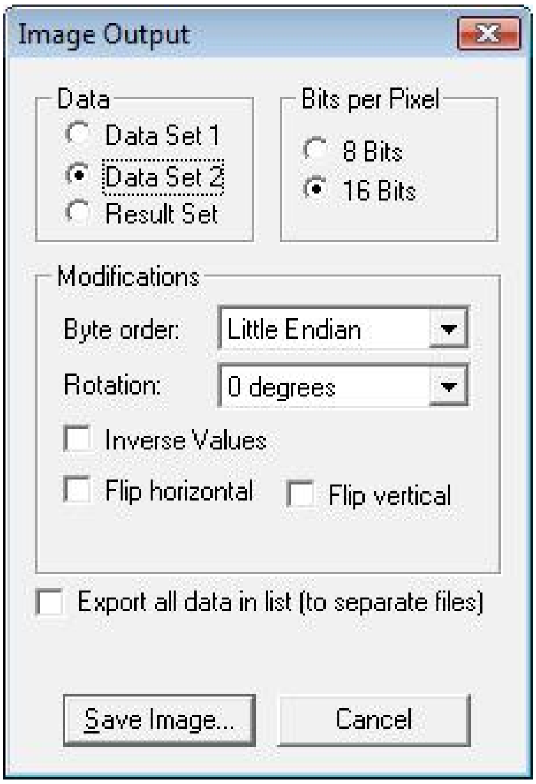

Data: Select which dataset image to export.

Bits per Pixel: Select the number of bits that shall be used for each pixel.

Modifications section:

Byte order: Select the byte order per pixel (only for 16 bit images). The options are **Little Endian** and **Big Endian**.

Rotation: Select the angle, if the image shall be rotated.

Inverse Values: Mark the checkbox to invert the values (i.e. 255-value for 8 bit, 65535-value for 16 bit).

Flip horizontal/Flip vertical: Mark the checkbox to flip the image horizontally and/or vertically.

Save Image: Click the button to save the image. The **Image Export** dialog opens.

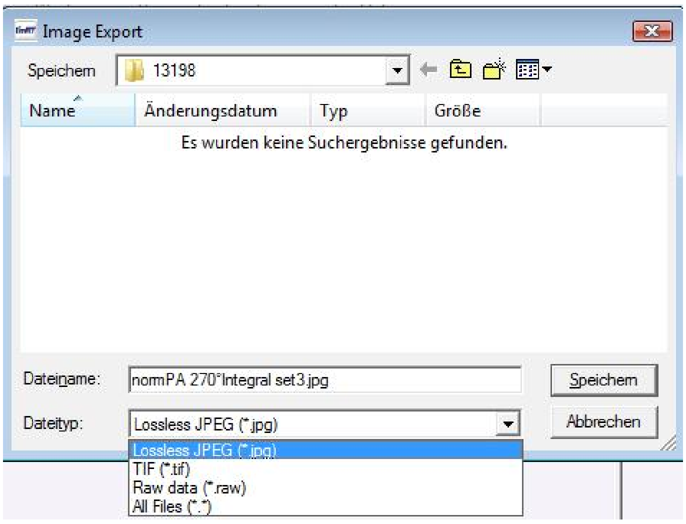

2. Select the format type, and click the **Save** button

10.4. Save Data

If you want to change the working directory, see *OmniPro I'mRT Software - Outline / Windows Environment*.

10.4.1. Save Measurement

1. Select the menu command ***File:Save*** or click the toolbar **Save** button. The **Save** dialog opens.

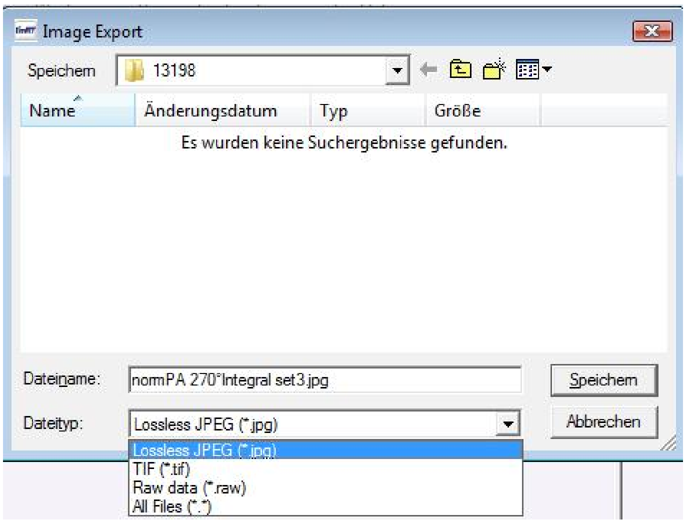

2. If you want to save the data in single files, (instead of binding them together to a workspace) clear the **Workspace** option.
3. Click the **Browse** button positioned to the right of one of the data sets. The **Save As** dialog opens.
4. Browse for the file, or enter the name of the file. Click **Save**. The **Save As** dialog closes.
5. Repeat steps 3 and 4 for the other data set.

10.4.2. Save Workspace

You can combine up to two data sets and one result set, and save them as a workspace.

1. Select the menu command ***File:Save***. Select the name and the location of the data sets as described above.
2. Select the **Workspace** option.
3. Click the **Browse** button. The **Save As** dialog opens.
4. Browse for the file, or enter the name of the file. Click **Save**. The **Save As** dialog closes.

10.4.3. Save Your Options

To save the above mentioned option(s), click **Save** to save the selections. The dialog closes.

NOTICE

IMPORTANT NOTICE

FILE FORMAT

The result file belonging to a workspace is saved as XXXX_res.opd, where XXXX is the name of the workspace file.

10.4.4. Save Measurement Parameters

The I'mRT QA/BIS, the MatriXX, or the film parameters setup from the Measurement parameters dialog, can be saved as an .opt file (OmniPro parameters file).

1. Select the menu command ***Measure:Save*** parameters. A standard **Save** dialog opens.
2. Enter the name of the file. Click **Save**. The dialog closes.

Open a saved parameters file with the menu command ***Measure:Load*** parameters.

NOTICE

IMPORTANT NOTICE

COPY PARAMETERS FROM THE ACTIVE FILE

It is also possible to copy the parameters from the active file to the measurement setup. This is done by selecting the menu command ***Measure:Copy*** parameters.

11. iViewGT Browser

11.1. Prerequisites

This interface reads images from the iView database, and prior to usage the iViewGT program must be set to save EPID images in lossless JPEG format. The iView database also contains the information (renormalization factors) needed for image pixel to intensity map conversion, and therefore you must assure that the iView program holds a database version higher or equal to 3.1. Another important factor is to setup the iViewGT in IMRT mode, with image capture of the complete radiation segment.

For a correct size of the image transferred to the I'mRT program, the following EPID and pixel information must be supplied from the iViewGT database:

- Size (w × h) (pixels),
- Horizontal scale (mm/pixels),
- Pixel aspect ratio (h/w),
- Centre (x,y) (pixels).

11.2. Browser

11.2.1. Installation

1. Select ***File:Import Data:iViewGT***. The **iViewGT Browser** dialog opens.
2. Create an ODBC source for the iViewGT program. The ODBC driver to be used is the dBase driver (*.dbf). This interface is tested with Microsoft dBase driver (*.dbf).
3. You can configure the ODBC driver via Windows control panel, ODBC datasources, or directly from the **I'mRT iView GT Browser**. Click the **Browse** button, and then the **New** button.

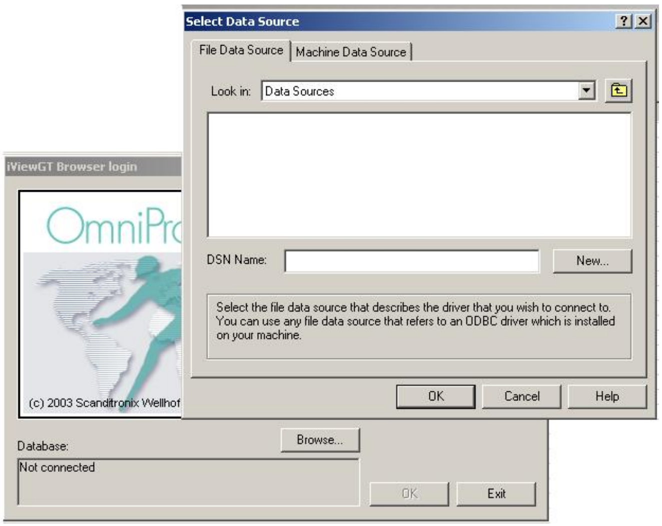

ODBC Setup Input:

ODBC Driver: Microsoft dBase driver (*.dbf).

Data Source Name: your logical name used for identifying the actual database

Database version: dBase IV

Database Directory: select the directory where the iView database is installed. Do not select single files.

4. Expand the dialog with the **Options** button.

The **Collating Sequence** checkbox must be set (marked) to **International**, otherwise the database becomes case sensitive.

The **Show Deleted Rows** checkbox must not be set (not marked).

11.2.2. Login

Before you can login to the I'mRT iViewGT Browser, you need to select the data source name. Type a name, or click the **Browse** button.

User name – Password: This is the username and password used with Elekta's iViewGT software. Observe that the user name and password depends on the currently selected database.

11.2.3. iViewGT Browser Dialog

11.2.3.1. Select images

1. Select ***File:Import Data:iViewGT***. Log in. The **iViewGT Browser** opens and displays the **Search for patients** dialog, with the options to search for patient's name or hospital's patient id.

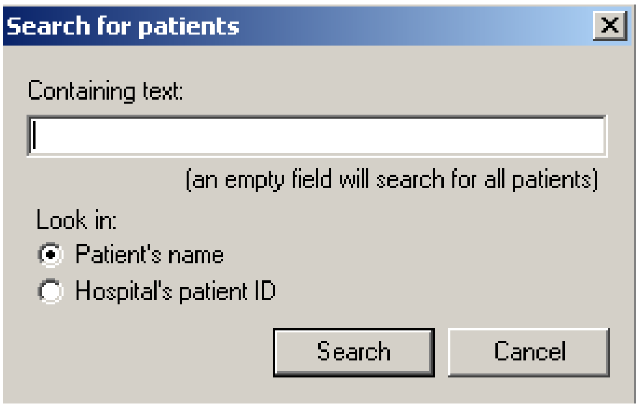

2. After selecting a patient, step through the buttons in the **iViewGT Browser** to make your selection of images.

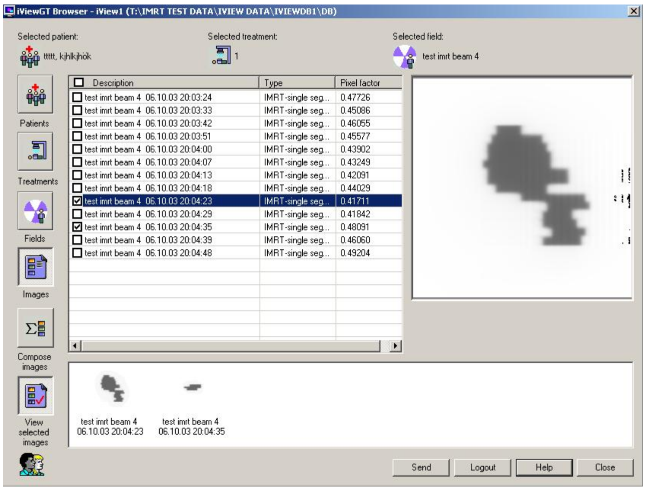

Patients: Activates a search for the Search for patients dialog.

Treatments: Shows the active treatments associated with a patient.

Fields: Shows the fields belonging to a treatment.

Images: Shows the images belonging to a field. Observe that an image can consist of several frames.

The image pixels are renormalized before they are saved in the database. The scaling factor is displayed in the column **Pixel factor**.

Example:

If 100 image frames were integrated at acquisition time during delivery of an I'mRT segment, a typical maximum brightness in this raw accumulated image might be 2 500 000 pixel units. Before saving into the database, the pixel values are renormalized in this accumulated image to give maximum brightness of, typically, 40 000, so all pixel values would be multiplied by 0.016. This is the value of the pixel factor.

NOTICE

IMPORTANT NOTICE

COMPOSED IMAGE

In the case of a composed image, the pixel factor is not available until the user wants to visualize this image.

A checkbox is placed in the table associated to the images. This checkbox is used for marking images to be composed or to be sent to the I'mRT application.

View selected images: Click this button to display the selected images in the pane beside the button. To remove an image, right-click in it and select ***Remove image*** in the context menu that appears. The corresponding checkbox will become unmarked. You can also remove an image by unmarking the corresponding checkbox.

You can add images from other patients, treatments, and fields, and send them simultaneously.

If an image is composed of several frames, an arrow (expand/collapse) button will be shown at the left side of the image. Each individual frame can be previewed. If you click on a frame in the preview window, the frame will be displayed in the right side of the browser (image holder). If you left click with the mouse on any image in the image holder, a new image dialog will be displayed. The left mouse button gives you a zoom function.

11.2.3.2. Compose Images

Compose images: This button allows you to compose images. All images with a marked checkbox will be composed to a single image.

In image composition, a max internal scale factor is used. Observe that an image does not hold an absolute dose.

During image composition, all images/frames are calculated with a renormalization factor and scaled relative to each other.

11.2.3.3. Send Images to the Main Application

Send: All images with a marked checkbox will be transferred to the OmniPro I'mRT application for further analysis. It is possible to select images from different treatments, fields, or patients, and send them simultaneously. If an image consists of frames, a dialog will be shown, asking if the frames shall be transferred to the I'mRT application.

The image data is normalized before it is sent to the main application. The pixel value, read from the image, is divided with pixel factor, resulting in a 32 bit pixel value. A median filter is applied so that the bad pixels are not taken into account for the calculation of the maximum value. Thus they have no influence on the rescaling factor, but they are not removed from the image.

Set origin: If the origin of the image was not set in the database, the **Set origin** dialog will open when you click the **Send** button. This dialog gives you the option to set the origin in the centre of the image, before it is sent.

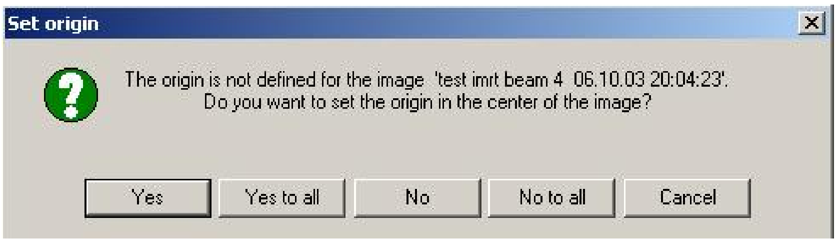

11.2.4. Close and Log Out

Close: The **iViewGT Browser** dialog will be closed, but the username and password are remembered for the next dialog usage.

Logout: **The iViewGT Browser** dialog will be closed and you will be logged out. You need to enter the username and password the next time you open the dialog.

⚠ WARNING

WARNING

CORRECT ELEKTA IVIEWGT SOFTWARE VERSION

You must ensure that you use the correct version of Elekta's iViewGT software. The version must contain an image renormalization factor.

⚠ WARNING

WARNING

CONNECT TO THE CORRECT DATABASE

You must ensure that you connect to the correct database. It is possible to set up/configure multiple ODBC drivers.

⚠ WARNING

WARNING

VERIFY PATIENT SELECTION

You must ensure that you select the correct patient, treatment, fields, and images. The current selection is displayed in the upper part of the **iViewGT Browser** dialog.

⚠ WARNING

WARNING

DOSIMETRY LIMITATIONS

You must be aware of the dosimetry limitations of the image information given from the iViewGT.

⚠ WARNING

WARNING

CORRECT IMAGE TYPE

For I'mRT verifications you must ensure that you are using the correct image type. The image type is displayed in the **Type** column in the image table. For I'mRT verifications you must ensure that you use a correct I'mRT image capturing mode.

Image Technology is used within the **iViewGT Browser**:

Image Technology is provided under License by AccuSoft Corporation.

ImageGear© 1996 – 2002. All Rights Reserved.

12. Maintenance

12.1. OmniPro I'mRT Software

12.1.1. ODBC Data Source

12.1.1.1. Redefine ODBC

If a problem with the data base connection occurs, you may have to define the Open Database Connectivity (ODBC) data source again:

1. Select ***Tools:Options***. The **Options** dialog opens. Click the **Environment** tab.

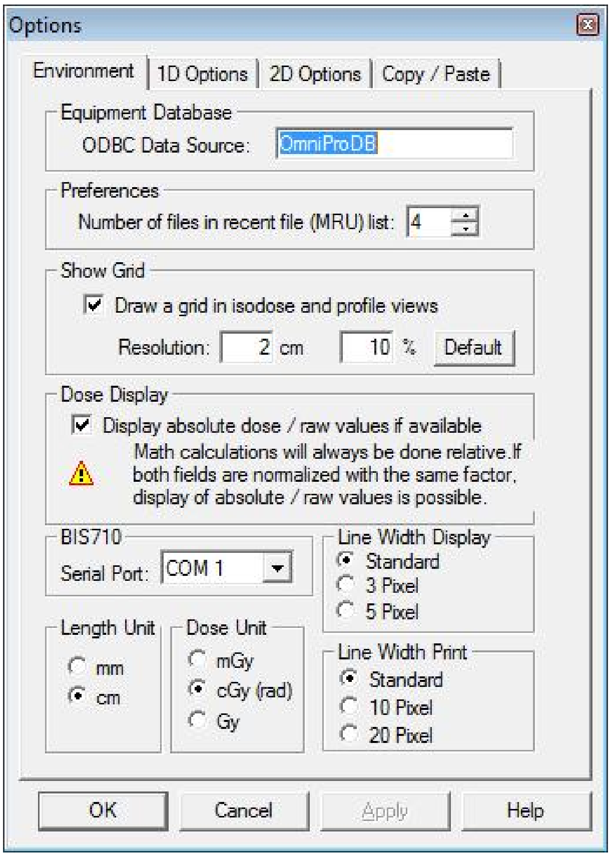

2. Enter OmniProDB in **ODBC Data Source**.
3. Click **OK**.

12.1.1.2. Moving the Database

NOTICE

IMPORTANT NOTICE

REINSTALLATION REQUIRED

Change of the location of the database requires reinstallation of the program. The existing database can be copied over the newly created database after installation, or copied there before installation (will not be overwritten).

To move the database to another location (after the initial installation), follow the steps below:

4. Move the file EquipDB.mdb to the new destination.
5. From the Windows Start menu select: Settings/Control Panel/32bit ODBC.
6. Select OmniPro DB in the **System DSN** tab.
7. Click **Configure**. Click **Select**.
8. Browse for EquipDB.mdb. Click **OK**.
9. Click **OK**.

12.2. MatriXX

12.2.1. General

Except for occasional cleaning, the MatriXX are maintenance-free.

12.2.2. Cleaning

To clean the housing of the device, use a soft, dry duster, moistened with a cleaning agent. Do not use organic solvents.

Use dry compressed air to clean the connectors on the rear side of the device, and the cable connectors.

12.2.3. Repair

Extremely low-current devices, like ion chamber detectors, require special knowledge and tools. Do not try to repair MatriXX yourself, but return it to IBA Dosimetry.

12.2.4. Recalibration

MatriXX needs recalibration, please return it to IBA Dosimetry.

13. Troubleshooting

13.1. Hardware

13.1.1. MatriXX

Problem	Possible reason	Solution
Device cannot be found by the computer / is not communicating	IP / subnet mask is not correctly entered.	Ping the device.<br>Check IP / subnet settings.
	Device and computer are on different gateways.	Check gateway settings.
	Cross-over cable not used for direct connection to the computer.	Use cross-over cable.
	Adapter is used for connection.	Do not use adapters (e.g. USB-Ethernet converter) for connection.
	Ethernet cable / pins are broken or defect.<br><br>Network socket is defect.<br><br>Network (cable) defect or is not compatible with CAT-6.<br><br>Ethernet hub / switch defect.	
	Ethernet port blocked by other application	Check if the Ethernet port is available.
	Recommended cable length exceeded.	Use an Ethernet minihub and ping the device.
	The Ethernet port can be deactivated if a battery-powered laptop is used.	Check power administration.
	Firewall is active and blocks communication.	Deactivate the firewall.
	The laptop has a WiFi interface.	Verify that the wireless internet connection is turned off.
Impossible to install / modify / set up the device on the computer.	Operator does not have administrator rights.	Log-on with administrator rights before the program is started.
XX Setup tool not accepted by the local network	Network firewall settings.	Add the XX Setup tool to the list of applications that are allowed access to the network.

Problem	Possible reason	Solution
IP address to MatriXX lost		Press the AUX button on the MatriXX.
Pixels are under- / over-responding on MatriXX.		Perform user calibration (requires OmniPro I'mRT version 1.6 or higher).
Cold spots in scan data.	Irradiation-induced change of the PLD memory.	Reboot MatriXX.
Linac interlock when measuring with MatriXX mounted on the gantry.	Gantry adapter not recognized as an accessory by the Linac software.	Add the gantry adapter as an accessory in the Linac software.

13.1.2. Scanners, I'mRT Phantom, I'mRT QA, BIS 710

Problem / Error message	Possible cause	Action
Vidar scanner problem		Refer to Vidar scanner manuals.
I'MRT QA problem		Refer to I'mRT QA manual.
BIS 710 problem		Refer to the BIS 710 Manual.
I'mRT Phantom problem		Refer to the I'mRT Phantom manual.

13.2. Software

13.2.1. Installation

Problem / Error message	Possible cause	Action
Error message during installation:<br>"1607: Unable to install InstallShield scripting runtime."	Problems with the isscript-file installation.	Right click the isscript.msi from the OmniPro I'mRT installation disk, and select Install. A wizard will guide you through the installation.
The administrator on the local computer has no access to the network location where the database shall be located.		Log in as local administrator, and install the program and the database locally on the computer.<br>Log in as user with network access.<br>Create a folder on the network. Map the folder to a driver letter (same letter for all users)<br>Move the database to the new location.<br>Configure the ODBC data source, see *12.1.1 ODBC Data Source*.

13.2.2. Database and Equipment Setup

Problem / Error message	Possible cause	Action
Corrupt database	It may happen that a database file gets corrupted. Most often the actual data is not affected, only the "index keys" used to find the data.	Recreate the indices:<br>From the Windows ***Start*** menu, select ***Settings/Control Panel/32bit ODBC***.<br>Select **OmniPro DB** under the **System DSN** tab.<br>Click **Configure**.<br>Click **Repair** in the dialog that opens.<br>Browse for EquipDB.mdb.<br>Click **OK**.
Broken database connection		Define the Open Database Connectivity. See *12.1.1 ODBC Data Source*.

Problem / Error message	Possible cause	Action
Error message: "Access to default.opt denied" when closing the measurement parameter dialog.	The user has no access to the application folder.	All OmniPro I'mRT users must have read and write access to the application folder (i.e. the folder where OPimrt.exe is located).
Windows 2000, network installation of database: A new (empty) database has been installed at C:\EquipDB.mdb<br>or<br>the user is asked to (re)insert a CD with the OmniPro I'mRT software.	Restricted access to network for some users.<br>The database directory is not mapped in the same way for the different users.	Restore network connection.<br>Give network access to all OmniPro I'mRT users (levels), alternatively log in as user with network access.<br>Map the database directory to the same letter for ALL users.<br>In all of the above cases: Check that the database connection is still correct (see *12.1.1 ODBC Data Source*). It may be necessary to delete the (empty) database at C:\EquipDB.mdb and restore the ODBC data source connection.
The equipment (e.g. radiation device) under the ***Equipment*** menu and in **Measure:Select Clinic and Device** the dialog has disappeared.	It is possible to define two or more clinics. Each clinic has its own equipment definition (e.g. accelerators). When one clinic is selected in ***Equipment:Select Clinic and Device*** it is only possible to see the devices defined for this clinic.	Select the correct clinic in the **Select Clinic and Device** dialog.
Error message: "No Clinic found" when trying to perform an Equipment setup.	No clinic is selected in the **Select Clinic and Device** dialog. After clinics have been defined in the equipment setup, one of them must be selected.	Select ***Equipment:Select Clinic and device.***<br>Select clinic in the **Clinic** list.<br>Click **OK**.

13.2.3. Display of Data

Problem / Error message	Possible cause	Action
No imported or measured data are displayed.	The wrong view is selected<br>or<br>The data level is outside the palette or dose range	Select another view (2D or 3D). See *6.1.2.1 Panes.*<br>or<br>Rescale the data or change the range. In the second case, edit the profile cursor first and check the data range.

Problem / Error message	Possible cause	Action
I'mRT QA/BIS: the measurement data are off axis by a large distance.	The correction image has not been defined correctly.	Define the correction image in the I'mRT QA/BIS tool bar. See *Equipment Setup and Calibration / Correction Image.*
I'mRT QA/BIS: The measurement data are very (!) noisy.	The correction image has not been defined correctly.	Define the correction image. See *7.8.2.1 I'mRT QA/BIS Correction Image.*
I'mRT QA: No movie, only one image is displayed after measuring in movie mode.	Only one single image can be displayed at the same time, all other images are found in the **Field list**.	Open the **Field list** dialog. Click the toolbar button, or select ***View:Field list***.<br><br>Select the movie image you want to edit, or click **Play movie**.
I'mRT QA/BIS measurement is selected but the I'mRT QA/BIS toolbar remains dimmed (non active).		Select Data set 1 or Data set 2.
2D dose distributions can not be compared with 3D dose data.	Measured film data are 2D distributions, 3D dose cubes are 3D data. 2D Data must be converted to a 3D cube first.	Select the film data.<br><br>Select ***Tools:Build 3D cube***.<br><br>Set the **Offset** for each film.<br><br>Select **Build 3D cube**.<br><br>Select ***Workspace:Plan Verification 3D***.
The result of a gamma-calculation shows an interference pattern	Data with high resolution (typically filmdata) in **Dataset 1**, data with low resolution (typically planned data) in **Dataset 2**.	Open the low resolution data in **Dataset 1**, and the high resolution data in **Dataset 2**.<br><br>or<br><br>Use data with similar resolution.
Lost "old" data set? If a new measurement /import is performed while another data set is already open, the "old" data set disappears from the pane.	All fields are found in the in the **Field list**. No data is lost.	Open the **Field list** dialog.<br><br>Select requested field from the list. This field is displayed in the data set pane(s).<br><br>If saving the data set the new data will be saved as new fields in the same field list as the old ones.

14. Appendix

14.1. Algorithms

14.1.1. 1D Data

14.1.1.1. User-defined Protocols

NOTICE

IMPORTANT NOTICE

SYMMETRY AND FLATNESS CALCULATION

Symmetry and flatness calculations are performed on CAX-corrected (i.e. centered) data.

14.1.1.1.1. Flatness

The flatness value is calculated according to one of the following methods:

- D_{max} / D_{min} (IEC)

Flatness = D_{max} / D_{min} within the flattened area defined as in point 4. in *14.1.1.1.3 Flattened area*.

- Geom.field – 80% & 90%

Flatness = max. distances from 80 and 90% levels to the geometric field at the respective depth.

- Geom.field – 90% level

Flatness = max. distances from profiles 90% level to geometric field on surface.

- Variation over CAX

Flatness = $100 \cdot D_{max} / D_{min}$

within flattened area, defined as in point 2. in *14.1.1.1.3 Flattened area*.

- Variation over Mean (80%)

Flatness = $100 \cdot |D_{max} - D_{min}| / (D_{max} + D_{min})$

within flattened area, defined as in point 1. in *14.1.1.1.3 Flattened area*.

- Variation over Mean (3 cm)

Flatness = $100 \cdot (D_{max} - D_{min}) / (D_{max} + D_{min})$

within flattened area defined as FW - 3 cm.

14.1.1.1.2. *Symmetry*

The symmetry value is calculated according to one of the following methods:

- Area

Compare areas (right – left) down to 50% of CAX

Symmetry = 100 · |AreaL – AreaR| / (AreaL + AreaR)

- Area / 2

Symmetry = (100 · |AreaL – AreaR| / (AreaL + AreaR)) / 2

- Largest Point Difference

Max difference in dose between points on equal distance from central axis within flattened area, defined as in *14.1.1.1.3 Flattened area*.

Symmetry = Max (|PointL – PointR|)

- Point Difference Quotient

Max quotient in dose between of points on equal distance from central axis within flattened area, defined as in 2 in *14.1.1.1.3 Flattened area*.

Symmetry = 100 · Max (|PointL / PointR| , |PointR / PointL|)

- Point Difference Quotient (IEC)

Max quotient in dose between points on equal distance from central axis within flattened area, defined as in 4 in *14.1.1.1.3 Flattened area*.

Symmetry = 100 · Max (|PointL / PointR|, |PointR / PointL|)

14.1.1.1.3. *Flattened Area*

The deviation value is calculated according to one of the following methods:

- D_{diff} / D_{CAX}

The largest value of 100 · |D_{min} - D_{CAX}| /D_{CAX} and 100 · |D_{max} - D_{CAX}| / D_{CAX} within flattened area, defined as in 2. in D_{max} / D_{CAX} 100 · (D_{max} - D_{CAX}) below.

- D_{max} / D_{CAX} 100 · (D_{max} - D_{CAX})

The Flattened Area can be defined in one of the following ways:

1. 80% of FW
2. FW – 2 cm if 2 cm ≤ FW < 10 cm

   80% of FW if 10 cm ≤ FW < 30 cm

   FW – 6 cm if 30 cm ≤ FW
3. FW – 3 cm
4. For main axes:

   FW - 2 · 1 cm if 5 ≤ FW ≤ 10

   FW -2 · (0.1 · FW) if 10< FW ≤ 30

   FW -2 · 3 cm if 30 < FW

For diagonals:

FW -2 · 2 cm if 5 ≤ FW ≤ 10

FW -2 · (0.2 · FW) if 10 < FW ≤ 30

FW -2 · 6 cm if 30 < FW

The Field Width, Offset and Penumbra values shall be displayed only if the point distances are < 10 mm.

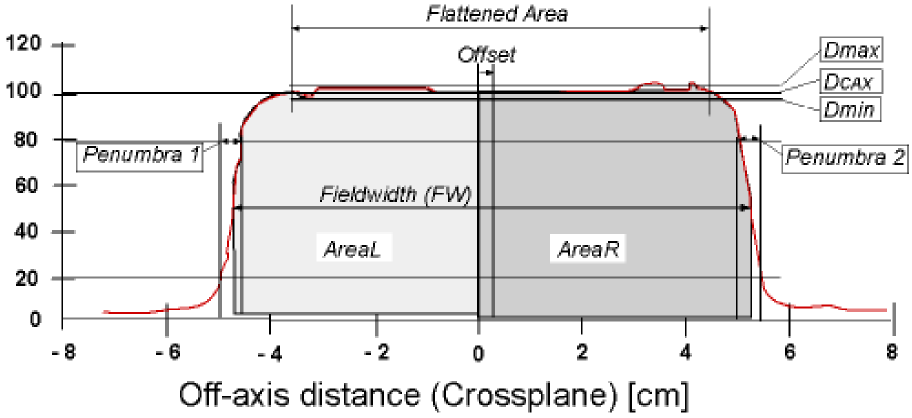

14.1.1.2. Predefined Protocols

The following pre-defined protocols for profiles are available:

- DIN
- IEC
- JARP86
- Elekta
- GE
- Siemens
- Varian

14.1.1.2.1. Photon Profile Protocols

DIN

Field width 50%

Penumbra 80%, 20%

Flatness Variation over CAX:

100 · D_{max} / D_{min} within flattened area defined as in item Flattened area below.

Symmetry Point Difference Quotient:

Max quotient in dose between two points on equal distance from central axis, within flattened area defined as in item **Flattened area**.

100 · Max (|Point L – Point R| , |Point R – Point L|)

Deviation Not defined

Flattened area FW-2 · 1 cm, if 2 cm = FW ≤ 10 cm

FW -2 · (0.1 · FW), if 10 cm < FW ≤ 30 cm

FW -2 · 3 cm, if 30 cm = FW

IEC

Field width Not defined

Penumbra 80%, 20%

Flatness: D_{max} / D_{min} (IEC):

100 · D_{max} / D_{min}

D_{max} anywhere in the radiation field

D_{min} within the flattened area defined as in item Flattened area

Symmetry Point Difference Quotient (IEC):

Max quotient in dose between two points on equal distance from central axis, within flattened area defined as in item **Flattened area**.

100 · Max (|Point L – Point R|,

|Point R - Point L|)

Deviation D_{diff} / D_{CAX}:

The largest of the values 100 · | D_{min} - D_{CAX}| / D_{CAX} and 100 · |D_{max} - D_{CAX}| / D_{CAX}, within flattened area defined as in item **Flattened area**.

Flattened area FW -2 · 1 cm, if 5 cm ≤ FW ≤ 10 cm

FW -2 (0.2 · FW), if 10 cm < FW ≥ 30 cm

FW -2 · 3 cm, if 30 cm < FW

JARP86

Field width Not defined

Penumbra Not defined

Flatness Variation over CAX:

100 · D_{max} / D_{min} within flattened area defined as in item **Flattened area**.

Symmetry Not defined

Deviation Not defined

Flattened area FW -2 · 1 cm, if 5 cm ≤ FW ≤ 10 cm

FW -2 (0.2 · FW), if 10 cm < FW ≥ 30 cm

FW -2 · 3 cm, if 30 cm < FW

Elekta

Field width Not defined

Penumbra 80%, 20%

Flatness D_{max} / D_{min} (IEC): 100 · D_{max} / D_{min}

D_{max} anywhere in the radiation field

D_{min} within the flattened area defined as in item **Flattened area**.

Symmetry Point Difference Quotient (IEC):

Max quotient in dose between two points on equal distance from central axis, within flattened area defined as in item **Flattened area**.

100 · Max (|Point L - Point R| , |Point R - Point L|)

Deviation D_{diff} / D_{CAX}:

The largest of the values 100 · |D_{min} - D_{CAX}| / D_{CAX} and 100 · |D_{max} - D_{CAX}| / D_{CAX}, within flattened area defined as in item **Flattened area**.

Flattened area FW -2 · 1 cm, if 5 cm ≤ FW ≤ 10 cm

FW -2 (0.2 · FW), if 10 cm < FW ≥ 30 cm

FW -2 · 3 cm, if 30 cm < FW

GE

Field width Not defined

Penumbra 80%, 20%

Flatness Variation over CAX:

100 · D_{max} / D_{min} within flattened area defined as in item **Flattened area**.

Symmetry Point Difference Quotient:

Max quotient in dose between two points on equal distance from central axis, within flattened area defined as in item Flattened area.

100 · Max (|Point L - Point R| , |Point R - Point L|

Deviation D_{diff} / D_{CAX}:

The largest of the values 100 · |D_{min} - D_{CAX}| / D_{CAX} and 100 · |D_{max} - D_{CAX}| / D_{CAX}, within flattened area defined as in item **Flattened area**.

Flattened area FW -2 · 1 cm, if 5 cm ≤ FW ≤ 10 cm

FW -2 (0.2 · FW), if 10 cm < FW ≥ 30 cm

FW -2 · 3 cm, if 30 cm < FW

Siemens

**Field width**	50%
**Penumbra**	80%, 20%
**Flatness**	Variation over mean (80%):
	100 · $\lvert D_{max} - D_{min}\rvert / (D_{max} + D_{min})$ within flattened area defined as in item **Flattened area**.
**Symmetry**	Area / 2:
	100 · |AreaL – Area R| / (AreaL + AreaR) / 2
**Deviation**	D_{max} / D_{CAX}:
	100 · (D_{max} / D_{CAX})
**Flattened area**	80% of FW

Varian

**Field width**	50%
**Penumbra**	80%, 20%
**Flatness**	Variation over mean (80%):
	100 · $\lvert D_{max} - D_{min}\rvert / (D_{max} + D_{min})$ within flattened area defined as in item **Flattened area**.
**Symmetry**	Point Difference:
	Max difference in dose between points on equal distance from central axis within flattened area, defined as 80% of FW
**Deviation**	D_{max} / D_{CAX}:
	100 · (D_{max} / D_{CAX})
**Flattened area**	80% of FW

14.1.1.2.2. Electron Profile Protocols

IEC

**Field width**	50%
**Penumbra**	80%, 20%
**Flatness**	Geometrical field 80% and 90% level:
	Max. distances from 80 and 90% levels to the geometrical field at the respective depth.
**Symmetry**	Point Difference Quotient IEC:
	Max quotient in dose between two points on equal distance from central axis, within flattened area defined as in item **Flattened area**.
	100 · Max (|Point L - Point R| , |Point R - Point L|)

Deviation D_{max} / D_{CAX}:

$100 \cdot (D_{max} / D_{CAX})$

Flattened area 1 cm inside the isodose contour (dose relative to dose at CAX)

JARP86

Field width Not defined

Penumbra Not defined

Flatness Geometrical field -90% level distance:

Max. distance from profiles 90% level to geometrical field on surface

Symmetry Not defined

Deviation Not defined

Flattened area Not defined

Elekta

Field width Not defined

Penumbra 80%, 20%

Flatness Geometric field 80% and 90%

Symmetry Point Difference Quotient IEC:

Max quotient in dose between two points on equal distance from central axis, within flattened area defined as in item **Flattened area**.

100 · Max (|Point L - Point R| , |Point R - Point L|)

Deviation D_{diff} / D_{CAX}:

The largest of the values $100 \cdot |D_{min} - D_{CAX}| / D_{CAX}$ and $100 \cdot |D_{max} - D_{CAX}| / D_{CAX}$, within flattened area defined as in item **Flattened area**.

Flattened area 1 cm inside the isodose contour (dose relative to dose at CAX)

GE

Field width Not defined

Penumbra 80%, 20%

Flatness Variation over CAX:

$100 \cdot D_{max} / D_{min}$ within flattened area defined as in item **Flattened area**.

**Symmetry**	Point Difference Quotient:<br>Max quotient in dose between two points on equal distance from central axis, within flattened area defined as in item **Flattened area**.<br>100 · Max (|Point L - Point R| , |Point R - Point L|)
**Deviation**	D_{diff} / D_{CAX}:<br>The largest of the values 100 · |D_{min} - D_{CAX}| / D_{CAX} and 100 · |D_{max} - D_{CAX} | / D_{CAX}, within flattened area defined as in item **Flattened area**.
**Flattened area**	FW-2 · 1 cm, if 2 cm = FW ≤ 10 cm<br>FW -2 · (0.1 · FW), if 10 cm < FW ≤ 30 cm<br>FW -2 · 3 cm, if 30 cm = FW

<u>Siemens</u>

**Field width**	50%
**Penumbra**	80%, 20%
**Flatness**	Variation over mean (FW -3 cm)
**Symmetry**	Area / 2: (100 · |AreaL - Area R| / (AreaL + AreaR)) / 2
**Deviation**	Not defined
**Flattened area**	FW -3 cm

<u>Varian</u>

**Field width**	50%
**Penumbra**	80%, 20%
**Flatness**	Variation over mean (80%)
**Symmetry**	Point Difference:<br>Max difference in dose between points on equal distance from central axis within flattened area, defined as 80% of FW
**Deviation**	Not defined
**Flattened area**	80% of FW

14.1.1.3. FermiFit Interpolation

The profiles are internally and individually normalized to 100%. The fit works on left and right penumbra zones (e.g. 10% - 90% area). The width shall be equal on both sides.

The function used is:

$f(x) = 1 / (1 + \exp((x-x0)/\sigma))$

A least-square fit is used for x0 (position) and σ (width).

14.1.1.4. Linear Interpolation

14.1.1.4.1. Converting to a Lower Resolution

An average over all source pixels within the area of a target pixel is calculated and set as value for the new pixel.

14.1.1.4.2. Converting to a Higher Resolution

All target pixels are calculated by the four surrounding source pixels, using a binlinear algorithm:

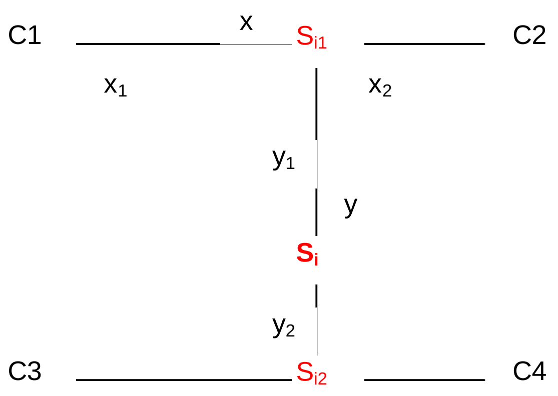

C1..C4: Signal of source pixels

Si: Interpolated signal

x: x-distance between C1 and C2 (equals x-distance between C3 and C4)

x1: x-distance between C1 and Si (equals x-distance between C3 and Si)

x2: x-distance between Si and C2 (equals x-distance between Si and C4)

y: y-distance between C1 and C3 (equals y-distance between C2 and C4)

y1: y-distance between C1 and Si (equals y-distance between C2 and Si)

y2: y-distance between Si and C3 (equals y-distance between Si and C4)

The interpolation is done in three steps:

1. Linear interpolation between C1 and C2:

   $S_{i1} = C1 + (x1 / x) \cdot (C2-C1)$

2. Linear interpolation between C3 and C4:

   $S_{i2} = C3 + (x1 / x) \cdot (C4-C3)$

3. Linear interpolation between S_{i1} and S_{i2}:

   $S_i = S_{i1} + (y1 / y) \cdot (S_{i2} - S_{i1})$

14.1.2. 2D Data

14.1.2.1. Gamma Method

For practical usage of the gamma method in OmniPro I'mRT please refer to chapter *9.5.6.1 Practical usage of the Gamma Method.*

If comparing two dose distributions, the simplest methods, e.g. comparing isodoses, or difference, may not be appropriate.

Misjudgment when checking the agreement of two dose distributions may occur in the following cases:

- The difference between two dose-distributions can be large in high-gradient regions, even if the isodoses are relatively close to each other.
- The isodose distance between two dose distributions can be large in regions with a flat dose distributions, though the difference in dose may be quite small.
- The gamma method combines both methods mentioned above.
- The user can define dose difference- and isodose distance pass/fail criteria.
- If both parameters (dose and isodose distance) are outside their pass/fail criteria, the agreement "fails" according to the gamma method.
- If only one parameter is outside the defined pass/fail criteria but the other well inside, the result of the comparison can still pass the calculation.

The gamma value for the measurement point rm is defined as:

$$\gamma(\mathbf{r}_m) = \min\{\Gamma(\mathbf{r}_m,\mathbf{r}_c)\}\forall\{\mathbf{r}_c\}$$

where

$$\Gamma(\mathbf{r}_m,\mathbf{r}_c) = \sqrt{\frac{r^2(\mathbf{r}_m,\mathbf{r}_c)}{\Delta d_M^2} + \frac{\delta^2(\mathbf{r}_m,\mathbf{r}_c)}{\Delta D_M^2}}$$

$$r(\mathbf{r}_m,\mathbf{r}_c) = |\mathbf{r}_c - \mathbf{r}_m|$$

and

$$\delta(\mathbf{r}_m,\mathbf{r}_c) = D_c(\mathbf{r}_c) - D_m(\mathbf{r}_m)$$

The pass-fail criteria are:

$\gamma(\mathbf{r}_m) \leq 1$	calculation passes
$\gamma(\mathbf{r}_m) > 1$	calculation fails

.

This gamma calculation is then performed for all r_m.

$\mathbf{r}_m$	position of a single measurement point (set into origin for this calculation)
$\mathbf{r}_c$	spatial location of the calculated distribution relative to the measurement point
Δd_M	passing criteria for isodose distance
$\Delta \mathrm{D}_M$	passing criteria for dose
$D_c(\mathbf{r}_c)$	calculated dose in r_c
$D_m(\mathbf{r}_m)$	measured dose in r_m

Ref: Low, Harms, Mutic and Purdy: A technique for the quantitative evaluation of dose distributions Med. Phys. 25 (5) May 1998, pp658

14.1.2.2. Gamma Digital

The digital Gamma is optimized for digital images, having a low resolution (in contrast to films), but it can also be used with high resolution images.

The calculations are based on the ones for the conventional gamma; the same parameter settings are used.

The result of each pixel can only be 'passed' (0) or 'failed' (2) No concrete value is calculated. The value 2 for 'failed' was selected to be able to use the same palette as for the conventional gamma. The latter point usually results in a noticeable increase of speed needed for the calculation.

Peculiarities of digital images are considered in the calculation, e.g. steep edges between two adjacent pixels in a coarse grid (for details se the reference below).

The calculation of the pass or fail state for a point consists of three successive filters:

The first level is directly based on the conventional gamma, with the difference that the calculation is stopped as soon as a value lower than or equal to 1 is found.

If level 1 wasn't successful, the point under investigation is passed to level 2. If there are any two points within the accepted distance having a delta dose of opposite sign, the distribution definitely intersects the ellipsoid of acceptance. As we are not interested in the actual value, the point can be set to 'passed'.

If level 2 still didn't succeed the point is passed to level 3. It checks if the direct neighbor points of the accepted distance have a delta dose of opposite sign compared to the points within the accepted distance. If this is the case, the intersection point (where delta dose = 0) is calculated by linear interpolation. If this point is situated inside the accepted distance, the point finally passes.

As only pass/fail states are calculated, not the actual gamma value, the search distance parameter is not taken into account in the algorithm.

NOTICE

IMPORTANT NOTICE

DIGITAL GAMMA MAPS

It was also found that there are slight deviations between digital gamma maps and normal gamma evaluation (not all point with gamma > 1.0 appear red in the digital gamma computation. This is a consequence of the approximative and fast computation of the digital gamma map.

Ref:: Depuydt, Van Esch and Huyskens: A quantitative evaluation of IMRT dose distributions: refinement and clinical assessment of the gamma evaluation. Radiotherapy and Oncology 62 (2002) 309 - 319.

14.1.2.3. DTA

The DTA is the distance between a dose point in the calculated distribution and the nearest point in the measured distribution containing the same dose value.

The user can define pass/fail criteria for the dose difference distance.

14.1.2.4. Correlation

The correlation coefficient r describes how well two data-distributions are correlated to each other.

r = 1 means that they are highly correlated,

r = 0 means that there is no statistical correlation between the two distributions.

Example:

Two identical fields have the correlation coefficient 1. When you move one of the fields, or if you rescale one of the fields, the correlation coefficient is still 1, since you just need to move the dose distribution or rescale the dose, and the fields are identical again. Therefore they are highly correlated.

$$r = \frac{\sum(X_i - \bar{X})(Y_i - \bar{Y})}{\sqrt{\sum(X_i - \bar{X})^2(Y_i - \bar{Y})^2}}$$

$$-1 \leq r \leq 1$$

X_i	measured Dose in position *i*
$\bar{X}$	average measured Dose
$\bar{X}$	calculated Dose in position *i*
$\bar{Y}$	average calculated Dose

14.2. References

1 Low, Harms, Mutic, Purdy: A technique for the quantitative evaluation of dose distributions Med. Phys. 25 (5) May 1998, p.658

2 DICOM Conformance Statement,
IBA Dosimetry document no P-07-002-510-002

3 DIN 6800, Teil 2, August 1980. Dosimessverfaren nach der Sondenmethode for Photonen- und Elektronenstrahlung IEC 1989. Medical electron accelerators – Functional performance characteristics. International Electrotechnical Commission, Geneva (IEC Publication 976)

4 IEC 1989a. Medical electron accelerators in the range 1 MeV to 50 MeV – Guidlines for functional performance characteristics. International Electrotechnical Commission, Geneva (IEC Publication 977)

5 JARP (Japanese Association of Radiological Physicists) Fax from Yoshitano at Toyo Medic co,ltd. Feb 10 and April 4, 1997 (based on the maintenance and management program for External Therapy Machine prepared by the Research and Development Committee from the Japan Radiation and Tumor Society 1992).

6 Elekta

7 GE

8 Siemens (Acceptance data for Siemens Mevatron (Siemens protocol) /GNn)

9 Varian: Equipment specifications for Clinac 2100C Linear Accelerator

10 I'mRT Phantom User's Guide,
IBA Dosimetry document no PW-02-003-510-001

14.3. Glossary of Terms

**ADC**	Analogue-to-digital converter. (ADC units are "house numbers" when scanning a film). With the help of a step film, a correlation between ADC-units and OD can be established.
**Applicator**	Extended collimator used for electron fields, also called Cone or Tube.
**Array**	Presents the Data set pane in a 2D array view.
**ASCII**	**A**merican **S**tandard **C**ode for **I**nformation **I**nterchange; a code for information exchange between computers made by different companies.
**Background**	The signal of a measurement device without any beam.
**BIS**	**B**eam **I**maging **S**ystem. IBA Dosimetry provides three beam imaging systems: I'mRT QA, BIS 710, and MatriXX.
**BIS2G**	A 2D detector array by IBA Dosimetry, sold until 2nd quarter 2003 under the product name BIS2G. The new product name is I'mRT QA.<br>Its predecessor was the BIS 710.<br>In this manual the term I'mRT QA/BIS is used in information that applies to both I'mRT QA and BIS 710.
**BIS 710**	A 2D detector array by IBA Dosimetry.<br>In this manual the term I'mRT QA/BIS is used in information that applies to both I'mRT QA and BIS 710.
**BQI**	**B**eam **Q**uality **I**ndex; Q(TPR20/10).
**CAX**	Central axis (beam axis).
**CCD**	**C**harge **C**oupled **D**evice: The digital camera in the BIS uses a CCD chip. The light from the Scintillator hits the CCD chip, where it converts to a signal which is sent to the computer.
**CCW**	Counter Clockwise
**cGy**	Gray [J/Kg] 1 Gy = 100 cGy = 100 rad.
**Correction image**	A correction, individually for every BIS, corrects among others for inhomogenities of the scintillator.
**Correlation coefficient**	Describes how well two data sets are correlated with each other. r = 1 means perfect correlation, r = 0 means no correlation.
**CW**	Clockwise

**D_{max}**	Dose maximum
**D_{min}**	Dose minimum
**DICOM**	**D**igital **I**maging and **CO**mmunications in **M**edicine. A standard for communications between medical imaging devices, developed by ACR-NEMA (American College of Radiology - National Electrical Manufacturer's Association).
**DTA**	**D**istance **T**o **A**greement. The DTA is the distance between a dose point in the calculated distribution and the nearest point containing the same dose value in the measured distribution.
**dpi**	Dots per Inch. A unit used to describe the resolution of scanners.
**Field**	Radiation Field. One single dose distribution. One or more fields can be saved in one Data set.
**Film calibration**	With the help of a number of films, irradiated with known doses, the optical density can be calibrated to dose.
**Fixture**	Attachment in-between the BIS and the treatment head, allowing the servo to rotate with the gantry.
**Fog**	The optical density of an unexposed but developed film.
**Frame grabber**	A PCI card mounted in the computer: It acts as an interface between the BIS and the OmniPro I'mRT software.
**FW**	Field Width.
**Gy**	Gray [J/Kg]. 1 Gy = 100 cGy = 100 rad.
**Head mounted**	Fixed to the gantry (head).
**I'mRT QA**	A 2D detector array by IBA Dosimetry, sold until 2nd quarter 2003 under the product name BIS2G. The new product name is I'mRT QA.<br>In this manual the term I'mRT QA/BIS is used in information that applies to both I'mRT QA and BIS 710.
**I'mRT MatriXX**	A 2D detector array by IBA Dosimetry.
**Isocenter**	Rotation centre for the gantry.
**Isodose**	Points with the same dose value.
**MatriXX ^{Evolution}**	A 2D detector array by IBA Dosimetry, especially intended for rotational techniques.
**MeV**	MegaelectronVolt, a unit used for the nominal or real energy of a beam consisting of charged particles (electrons, protons).

**MU**	Monitor Units, "kicks".
**MV**	MegaVolt, accelerating potential of Linac.
**OD**	**O**ptical **D**ensity
**ODBC**	Short for **O**pen **D**ata **B**ase **C**onnectivity; a standard database access method developed by the SQL Access group.
**OpenGL**	Standard for 3D graphics.
**Panes**	Data are displayed in four panes or views in the OmniPro I'mRT software.
**Patient coordinates**	Coordinate system used in the treatment planning system.
**Phantom coordinates**	Coordinate system used in the OmniPro I'mRT software (according to IEC standard).
**rad**	Gray [J/Kg]. 1 Gy = 100 cGy = 100 rad.
**Reduction**	BIS measurement: Only every second pixel is used (reduces the amount of data by four).
**Reference point**	Special point for each energy when Absolute Dosimetry is performed.
**Rescale**	Renormalize the dose, or change the geometric scale of a dose distribution
**ROI**	Region of interest
**RTOG**	Data format standard for exchange of treatment planning data, based on the AAPM Report #10.
**RTPS**	**R**adiation **T**reatment **P**lanning **S**ystem.
**SAD**	Source Axis Distance (isocentre distance).
**Scanner calibration**	With the help of a step film, the signal of the scanner (ADU units) is calibrated to optical density.
**Scintillator**	A material that converts ionizing radiation into light (used in the BIS).
**Smooth**	Reducing the noise and/or stochastic errors of measurement data.
**SSD**	**S**ource Skin (**S**urface) **D**istance.
**Step film**	Consists of a number of steps with well defined optical density values. It is used to perform a film scanner calibration (Signal to Optical density).
**Synchronize Views**	Profile cursor movements, and plane and orientation selections are performed in both data sets in the same way.
**Table mounted**	Positioned on the patient table.

**TCP**	(Internet) **T**ransmission **C**ontrol **P**rotocol.
**TPS**	**T**reatment **P**lanning **S**ystem.
**Water equivalent build up**	Free-in-air measurements are usually performed with a build-up. One reason is to reduce the contribution of low energy scattered radiation, which does not contribute at all to the dose inside the body.
**Workspace**	A workspace helps you to link data belonging to a special plan. The workspace file does not contain any 2D dose distribution data itself, but rather information about where data that belong together is saved. It can contain the link to two data set files (*.odp) and one result file (with the name *workspace name_res.opd*). Additionally it can contain Absolute Dose verification data and comments.

14.4. Complaint Reports

This section contains three copies of Complaint Report, OmniPro I'mRT, and three copies of Complaint Report, MatriXX.

Always keep one copy of the form. If needed, make more photocopies of the form. If there is no form, make a written report describing the fault with as much detail as possible. Please always include your name, address, email, phone, and the license number (found in the **About** box of OmniPro I'mRT).

The user shall report all complaints about the system to any representative of IBA Dosimetry, or directly to:

IBA Dosimetry GmbH

Service Department

Bahnhofstrasse 5

DE-90592 Schwarzenbruck

Germany

Phone: +49 9128 607-38

Fax: +49 9128 607-10

e-mail: service@iba-group.com

For customers in South and Middle America, USA, and Canada:

IBA Dosimetry America

3150 Stage Post Drive, Suite 110

Bartlett, TN 38133

USA

Phone: +1 901 386-2242

Fax: +1 901 382-9453

e-mail: USService@iba-group.com

For customers in China:

IBA China Dosimetry Department

No.6, Xing Guang Er Jie,

Beijing OPTO-Mechatronics Industrial Park (OIP)

Tongzhou District, Beijing

101111

P.R. China

Phone: +86 10 80809155

Fax: + 86 10 80809298

Complaint Report OmniPro I'mRT

Issuing person:	Date:

Customer information

Hospital name:		Contact person:
Address:		
Tel:	Fax:	E-mail:

Product information

Software version:	Serial /License number:	
**PC:**		
Type:	Processor:	Memory (RAM):
Operating system (including Windows version and service pack (if any)):		
**Film scanner:**		
Type:	Version:	Firmware:
**SCSI adapter:**		
Type:	Driver:	Version:
**I'mRT QA/BIS:**		
Type:	Serial number:	
**Frame grabber:**		
Type:		Driver version:

**Other hardware:**	
Unit:	Id /Serial number:

|

Description of the complaint

Appendices:

Complaint reported by:	Date:	Signature:

Complaint Report OmniPro I'mRT

Issuing person:	Date:

Customer information

Hospital name:		Contact person:
Address:		
Tel:	Fax:	E-mail:

Product information

Software version:	Serial /License number:	
**PC:**		
Type:	Processor:	Memory (RAM):
Operating system (including Windows version and service pack (if any)):		
**Film scanner:**		
Type:	Version:	Firmware:
**SCSI adapter:**		
Type:	Driver:	Version:
**I'mRT QA/BIS:**		
Type:	Serial number:	
**Frame grabber:**		
Type:		Driver version:

**Other hardware:**	
Unit:	Id /Serial number:

Description of the complaint

Appendices:

Complaint reported by:	Date:	Signature:

Complaint Report OmniPro I'mRT

Issuing person:	Date:

Customer information

Hospital name:		Contact person:
Address:		
Tel:	Fax:	E-mail:

Product information

Software version:	Serial /License number:	
**PC:**		
Type:	Processor:	Memory (RAM):
Operating system (including Windows version and service pack (if any)):		
**Film scanner:**		
Type:	Version:	Firmware:
**SCSI adapter:**		
Type:	Driver:	Version:
**I'mRT QA/BIS:**		
Type:	Serial number:	
**Frame grabber:**		
Type:		Driver version:

**Other hardware:**	
Unit:	Id /Serial number:

Description of the complaint

Appendices:

Complaint reported by:	Date:	Signature:
